# Identification of glioblastoma immune subtypes and immune landscape based on a large cohort

**DOI:** 10.1186/s41065-021-00193-x

**Published:** 2021-08-19

**Authors:** Huiyuan Zhang, Ying Chen

**Affiliations:** 1 Department of Medical Oncology, the First Hospital of China Medical University, Shenyang, China; 2 Key Laboratory of Anticancer Drugs and Biotherapy of Liaoning Province, the First Hospital of China Medical University, Shenyang, China; 3 Liaoning Province Clinical Research Center for Cancer, Shenyang, China; 4 Key Laboratory of Precision Diagnosis and Treatment of Gastrointestinal Tumors, Ministry of Education, Shenyang, China

**Keywords:** Glioblastoma, Prognosis, Immune subtype, Immune landscape, Co-expression modules, Riskscore

## Abstract

Glioblastomas (GBM) are the most common primary brain malignancy and also the most aggressive one. In addition, GBM have to date poor treatment options. Therefore, understanding the GBM microenvironment may help to design immunotherapy treatments and rational combination strategies. In this study, the gene expression profiles and clinical follow-up data were downloaded from TCGA-GBM, and the molecular subtypes were identified using ConsensusClusterPlus. Univariate and multivariate Cox regression were used to evaluate the prognostic value of immune subtypes. The Graph Structure Learning method was used for dimension reduction to reveal the internal structure of the immune system. A Weighted Correlation Network Analysis (WGCNA) was used to identify immune-related gene modules. Four immune subtypes (IS1, IS2, IS3, IS4) with significant prognosis differences were obtained. Interestingly, IS4 had the highest mutation rate. We also found significant differences in the distribution of the four subtypes at immune checkpoints, molecular markers, and immune characteristics. WGCNA identified 11 co-expressed module genes, and there were significant differences among the four subtypes. Finally, CD1A, CD1E, and IL23R genes with significant prognostic significance were selected as the final feature genes in the brown module. Overall, this study provided a conceptual framework for understanding the tumor immune microenvironment of GBM.

**Supplementary Information:**

The online version contains supplementary material available at 10.1186/s41065-021-00193-x.

## Introduction

Glioblastomas (GBM) are the most common primary malignant neoplasm accounting for 46.1% of all the tumors in the central nervous system (CNS) [1, 2]. Due to their diffusely infiltrative nature, aggressive GBM are challenging to treat. Surgery, radiotherapy, and temozolomide chemotherapy have become the standard treatments. Bevacizumab, an anti-vascular endothelial growth factor antibody (VEGF), can extend progression-free survival (PFS) but not overall survival (OS) [3, 4]. Nevertheless, the recent discovery of two novel prognostic biomarkers, IDH (isocitrate dehydrogenase) and MGMT (O6-methylguanine-DNA methyltransferase) promoter methylation, opened new diagnosis and treatment possibilities. IDH mutations are oncogenic, and studies have shown that IDH wild-type and IDH mutant are driven by different oncogenic processes and respond differently to GBM treatment [5]. On the other hand, as shown in a phase III study, MGMT promoter methylation predicted the value in patients with glioblastoma treated with alkylating drugs such as temozolomide. Therefore, MGMT promoter methylation status has become an important predictive biomarker of response to TMZ in GBM tumors [6, 7].

In recent years, immune checkpoint inhibitor (ICI) therapies, commonly targeting the PD-1/PD-L1 pathway, have changed the paradigm and improved the treatment of many cancers, including common melanoma and lung cancer. Nevertheless, immunotherapy treatment has a low response rate, and not all patients benefit from its use, suggesting that there may be a specific response pattern. The clinical efficacy, mechanism, and influencing factors of PD-1/PD-L1 checkpoint therapy in GBM remain unclear. Tumor-associated macrophages (TAM) are a significant component of the GBM microenvironment and contribute to the growth and aggressive behavior of GBM [8–10]. ERK1/2 and STAT3 signaling might play a vital role in anti-tumor immunosuppression [11–13]. Besides, many other factors can contribute to or suppress immunotherapy efficacy. For example, temozolomide treatment can contribute to the down-regulation of PD-L1 in GBM cells, thus impairing the efficacy of PD-1/PD-L1 inhibitors such as nivolumab [14]. On the other hand, CXCR and CD73 might improve or enhance anti-PD-L1 treatment [15, 16]. Targeting myeloid cells with anti-CXCR4 antibodies promoted anti-tumor immune responses and improved survival rates.

In this study, we conducted a multi-cohort retrospective study to identify four repeatable immune subtypes in glioblastoma patients. In addition, we used independent data to validate the subtypes and conduct comprehensive molecular identification. Each subtype was found to be associated with different gene expression profiling, and different subtypes had different patterns in tumor genetic aberrations, tumor-infiltrating immune cell composition, immune activation and suppression, and cytokine profiles, especially in relation to clinical prognosis. This study provided a novel conceptual framework for understanding the glioblastoma microenvironment. Our data may have clinical significance for the design of novel immunotherapy treatments and rational drug combination strategies.

## Materials and methods

### Dataset sources and data pre-processing

The processed Agilent gene expression microarray data (TCGA-GBM Agilent cohort) and clinical follow-up data of TCGA-GBM were downloaded from the UCSC Xena website. Four hundred forty-seven samples were included in the study after screening (Supplementary Table 1). TCGA-GBM samples were pre-processed as follows: 1) remove normal tissue samples data; 2) remove samples without survival state; 3) remove samples with overall survival time less than 30 days; 4) reserve the expression profile of immune-related genes. We also obtained RNA-Seq data from TCGA-GBM using the TCGA GDC Application Programming Interface (API). A total of 148 samples were included in the study after screening (Supplementary Table 2). These samples were pre-processed following these steps: 1) remove normal tissue samples data; 2) remove samples without survival state; 3) remove samples with overall survival time less than 30 days; 4) remove genes with 50% or more samples Fragment Per Kilobase of exon per Megabase (FPKM) equal to 0; 5) reserve the expression profile of immune-related genes and the FPKM values were log‐transformed using log2 (FPKM + 1).

A total of 2,006 immune-related genes were collected (Supplementary Table 3).The following categories of immune-related genes were collected for follow-up analysis from the literature [17]: immune cell-specific genes derived from single-cell RNA-seq data; genes of co-stimulatory or co-inhibitory molecules; cytokine and cytokine receptor genes; genes involved in antigen processing and presentation pathway; and other immune-related genes.

### Identification of GBM immune subtypes and immune gene modules

The consistent matrix was constructed using the ConsensusClusterPlus package in R [18]. The immune subtypes were obtained using the expression data of 1683 immune-related genes. Using the PAM algorithm and the “1-Pearson correlation coefficient” as the metric distance, we performed 500 bootstraps, each involving 80% of the patients in the training cohort. The number of clusters was set from 2 to 10, and the consistency matrix and the consistency cumulative distribution function were calculated to determine the best classification. The immune genes were grouped by consistent clustering, and the immune gene modules were obtained simultaneously using the same settings and parameters as described before.

### Functional analysis

Gene Ontology enrichment analysis of immune genes in each module was performed using DAVID (v6.8) to annotate the biological functions and the collected immune-related genes as background. The association of immune subtypes with 57 immune-related molecules and cell characteristics was evaluated using ANOVA [19].

### Assessment of the clinical, molecular, and cellular characteristics associated with immune subtypes

The prognostic value of immune subtypes with age and gender as covariates and overall survival (OS) as an endpoint in the training cohort was evaluated using the log-rank test and univariate and multivariate Cox regression. Variance analysis was then used to assess the correlation between immune subtypes and various immune-related molecular and cellular characteristics in the verification cohort.

### Immune landscape

Considering the dynamic characteristics of the immune system, the Graph Structure Learning method was used for dimension reduction to reveal the internal structure of the immune system and visualize the distribution of individual patients. Simply, this method projected high-dimension gene expression data into a lower-dimensional space preserving the local structure information [20]. This algorithm has been previously used to simulate the progression and definition of cancer using large and single-cell gene expression data [21, 22]. The obtained immune landscape reflected the relationship between patients in a non-linear manifold, which may complement the discrete immune subtypes defined in a linear Euclidean space.

## Results

### Construction of molecular subtypes based on immune-related genes

**Fig. 1:**
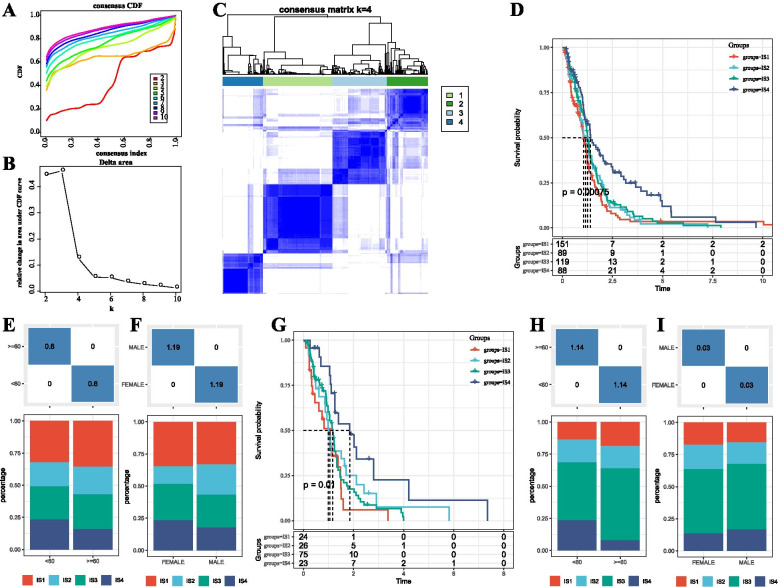
The immune subtypes in TCGA-GBM Agilent. **A** TCGA-GBM Agilent cohort CDF curves. **B** TCGA-GBM Agilent cohort CDF Delta area curve. Delta area curve of consensus clustering indicating the relative change in area under the cumulative distribution function (CDF) curve for each category number k compared with k – 1. The horizontal axis represents the category number k, and the vertical axis represents the relative change in area under the CDF curve. **C** Consensus k = 4 sample cluster heat map. **D** The KM curves of prognosis in four molecular subtypes. € Distribution of four immune subtypes in different age groups. **F** Distribution of four immune subtypes in different gender groups. The lower half was proportional, and the upper half was the distribution of the statistically significant difference between pairwise comparisons -log10 (*P-value*). **G** Prognostic differences of four immune subtypes in the TCGA-GBM HiSeq cohort. **H** The distribution of four immune subtypes in different age groups in TCGA-GBM HiSeq cohort. **F** Distribution of four immune subtypes in different gender groups in the TCGA-GBM HiSeq cohort

We extracted the immune-related gene expression profiles using the TCGA-GBM Agilent dataset. 1683 genes were obtained for follow-up analysis. 447 GBM samples were clustered using ConsistentClusterPlus, and the best cluster number was determined by the Cumulative Distribution Function (CDF). From the CDF Delta area curve, we obtained that Cluster = 4 represented the most stable clustering (Fig. 1A-B), and we chose k = 4 to obtain four Immune Subtype (IS) (Fig. 1C). Interestingly, further analysis of the prognostic features of the four immune subtypes suggested that there were significant differences in their prognosis, as shown in Fig. 1D. In general, IS4 showed a better prognosis, while IS1 was the poorest. Furthermore, we compared the four molecular subtypes with age and gender and found no significant differences between the four molecular subtypes in age and gender groups, as shown in Fig. 1E-F. In addition, molecular subtyping for RNASeq of TCGA-GBM was performed using the same methods, and, consistently with the training cohort, no significant differences were observed in the prognosis of these four immune subtypes (Fig. 1G). Similarly, no significant differences were found when comparing the four molecular subtypes with age and gender (Fig. 1H-I).

### Relationship between immune subtypes, TMB, and common gene mutations

**Fig. 2:**
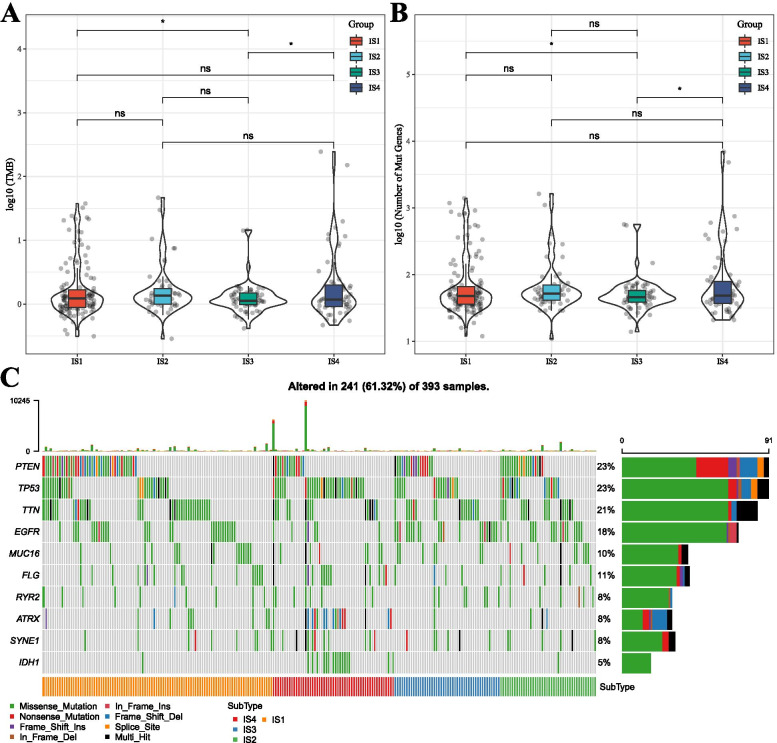
**A** Difference in the distribution of TMB in samples of four molecular subtypes. **B** Difference in the distribution of the number of mutations in samples of four molecular subtypes. A rank-sum test was used to determine the *P-value* (* < 0.05; ** < 0.01). **C** mutation characteristics of the top 10 significant mutation genes with the highest mutation frequency in each subtype

First, we downloaded the mutation dataset deal with mutect2 software from TCGA-GBM Agilent. We then calculated the tumor mutation burden (TMB) and analyzed the distribution of TMB in the four immune subtypes, as shown in Fig. 2A. TMB in IS1 was significantly lower than in IS3, and TMB in IS3 was lower than in IS4. In addition, we also quantified the number of gene mutations in samples from different immune subtypes and found that the number of mutations in IS3 subtypes was significantly lower than in IS4 (Fig. 2B). Furthermore, we screened 2,705 genes with a mutation frequency of more than 4 in each subtype. A Chi-square test was used to screen the genes with high mutation frequency in each subtype. The selection threshold was *P* < 0.05, and 987 genes were obtained (Supplementary Table 4). The top 10 highest mutation frequencies in all subtypes are shown in Fig. 2C. The mutation rate of IDH1 in IS4 was significantly higher than in the other IS, consistent with the good prognosis of patients carrying IDH1 mutations.

### Classical markers expression in chemotherapy-induced immune response in immune subtypes and immune checkpoint gene expression

**Fig. 3:**
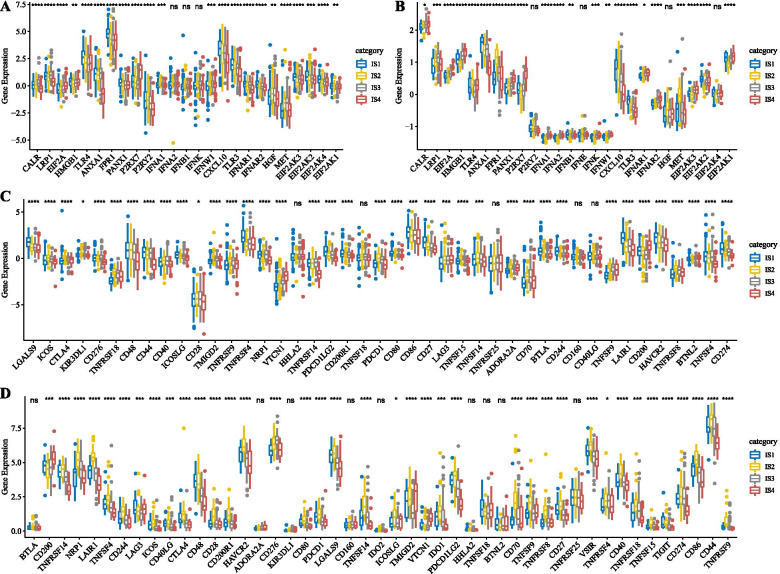
**A** The distribution difference of classical markers expression in chemotherapy-induced immune response in the TCGA-GBM Agilent. **B** The distribution difference of classical markers expression in chemotherapy-induced immune response in the TCGA-GBM HiSeq. **C** The distribution difference of immune checkpoint genes in the TCGA-GBM Agilent. **D** The distribution difference of immune checkpoint genes in the TCGA-GBM HiSeq. Significance was calculated using analysis of variance (* < 0.05; ** < 0.01; *** < 0.001; **** < 0.0001)

To observe differences in the distribution of classical marker expression in chemotherapy-induced immune responses among the four immune subtypes, we calculated the differences of these genes in the TCGA-GBM Agilent cohort and TCGA-GBM HiSeq, respectively. We found a total of 25 genes expressed in TCGA-GBM Agilent, and 22 (88%) of those genes were significantly different in each immune subtype (Fig. 3A). For the TCGA-GBM HiSeq dataset, we found 26 genes, and 22 (84.6%) of those were significantly different in each immune subtype (Fig. 3B). These data suggested that chemotherapy-induced markers of immune response had a significant difference in different immune subtypes, which might contribute to the different clinical progression of the disease. We also obtained 47 immune checkpoint-related genes from a previous study [23] and analyzed the differences between these genes in each IS. The results showed that 42 (89.3%) genes had significant differences in the TCGA-GBM Agilent cohort (Fig. 3C), and 38 (80.9%) genes had significant differences in the TCGA-GBM HiSeq cohort (Fig. 3D).

### Distribution differences of molecular marker-related genes in different immune subtypes

**Fig. 4:**
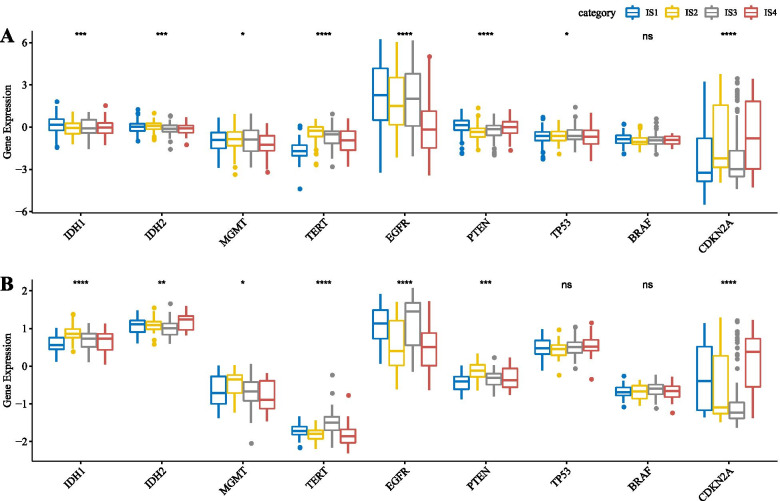
**A** The distribution difference of molecular marker-related genes in different immune subtypes in TCGA-GBM Agilent. **B** The distribution difference of molecular marker-related genes in different immune subtypes in TCGA-GBM HiSeq

We extracted the expression profiles of nine genes (IDH1, IDH2, MGMT, TERT, EGFR, PTEN, TP53, BRAF, and CDKN2A) from TCGA-GBM Agilent and TCGA-GBM HiSeq cohorts and analyzed their distribution in each subtype. We observed that all the genes except BRAF had significant differences among immune subtypes in the TCGA-GBM Agilent cohort (Fig. 4A). The expression of IDH1and EGFR in IS1 was significantly higher than in IS4, while expression of CDKN2A in IS4 was significantly higher than in IS1. For the TCGA-GBM HiSeq cohort, the expression difference of the nine genes in different immune subtypes is shown in Fig. 4B.

### Immune characteristics in different immune subtypes

**Fig. 5:**
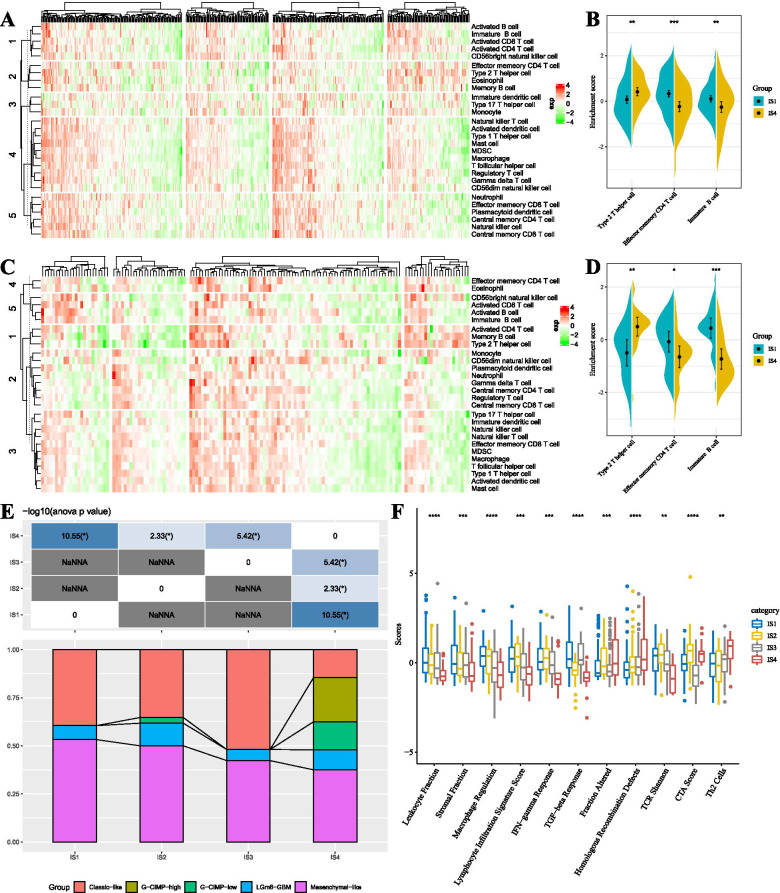
**A** The difference of enrichment scores of 28 immune cells in each subtype in the TCGA-GBM Agilent cohort. **B** The enrichment scores of immune cells with a significant difference in the good and poor prognosis subtypes in the TCGA-GBM Agilent cohort. **C** The difference of enrichment scores of 28 immune cells in each subtype in the TCGA-GBM HiSeq cohort. **D** The enrichment scores of immune cells with a significant difference in the good and poor prognosis subtypes in the TCGA-GBM HiSeq cohort. **E** The intersection of four immune molecular subtypes with the previous five molecular subtypes. **F** The distribution of four immune subtypes in 56 immune-related features; 11 immune features presented FDR < 0.05

Next, we aimed to compare the distribution of immune cell components in different immune subtypes. To do so, we obtained 28 immune cell marker genes from a previous study [24] and scored each type of immune cell using ssGSEA to determine the scores in each patient using the TCGA-GBM Agilent and the TCGA-GBM HiSeq cohorts. In the TCGA-GBM Agilent cohort, these immune cells were divided into five categories (Fig. 5A), and most of these immune cell components had differences in different subtypes. For example, “Type 2 T helper cell” was significantly lower in IS1 than in IS4, “Effector memory CD4 T cell” and “Immature B cell” were significantly higher in IS1 than in IS4 (Fig. 5B). Similar results were obtained in the TCGA-GBM HiSeq cohort (Fig. 5CD), suggesting that poor prognosis might be related to the activation of effector memory CD4 T cells, immature B cells, and the inhibition of type 2 T helper cells.

To study the relationship between the four immune molecular subtypes and the five molecular subtypes of previous pan-cancers, we extracted and compared the molecular subtypes of these samples from a previous study [19]. Based on multidimensional histologic data collected and analyzed from 1,122 glioma samples (WHO Grade II to WHO grade IV), the TCGA team classified the glioblastomas into IDH mutant and IDH wild-type [25]. The IDH mutant glioblastoma could be further divided into G-CIMP-low, G-CIMP-high, and combined deletion (codel), while the IDH wild-type glioblastoma can be subdivided into classic-like, mesenchymal-like, LGm6-GBM, and pilocytic astrocytoma-like (PA-like) GBM. Significant differences were found in the survival, grade, age, histological type, and other clinical features between the two types of glioblastoma. In IDH mutant glioblastoma, the prognosis of G-CIMP-low and G-CIMP-high was good, and the prognosis of classic-like, mesenchymal-like, and LGM6-GBM in IDH wild-type glioblastoma.

When studying the immune molecular subtypes, it can be observed that IS1, IS2, and IS3 subtypes were mainly composed of classic-like and mesenchymal-like GBM, while the proportion of G-CIMP-high and G-CIMP-low GBM in IS4 subtypes was higher than in IS1, IS2, and IS3 subtypes (Fig. 5E). These data were consistent with the poor prognosis of IS1, IS2, and IS3 subtypes in survival analysis. Besides, we evaluated the association of immune subtypes with 56 previously defined immune molecular characteristics (Supplementary Table 5). The 11 most significant immune-related features were identified by FDR < 0.05 (Fig. 5D). IS1 subtype had the highest “Leukocyte Fraction”, “Stromal Fraction”, “Macrophage Regulation”, “Lymphocyte Infiltration Signature Score”, “IFN-gamma Response”, “TGF-beta Response”, and “TCR Shannon”, while IS4 was significantly higher than IS1, IS2, and IS3 in “Fraction Altered”, “Homologous Recombination Defects”, and “Th2 Cells”.

### The immune landscape of GBM

**Fig. 6:**
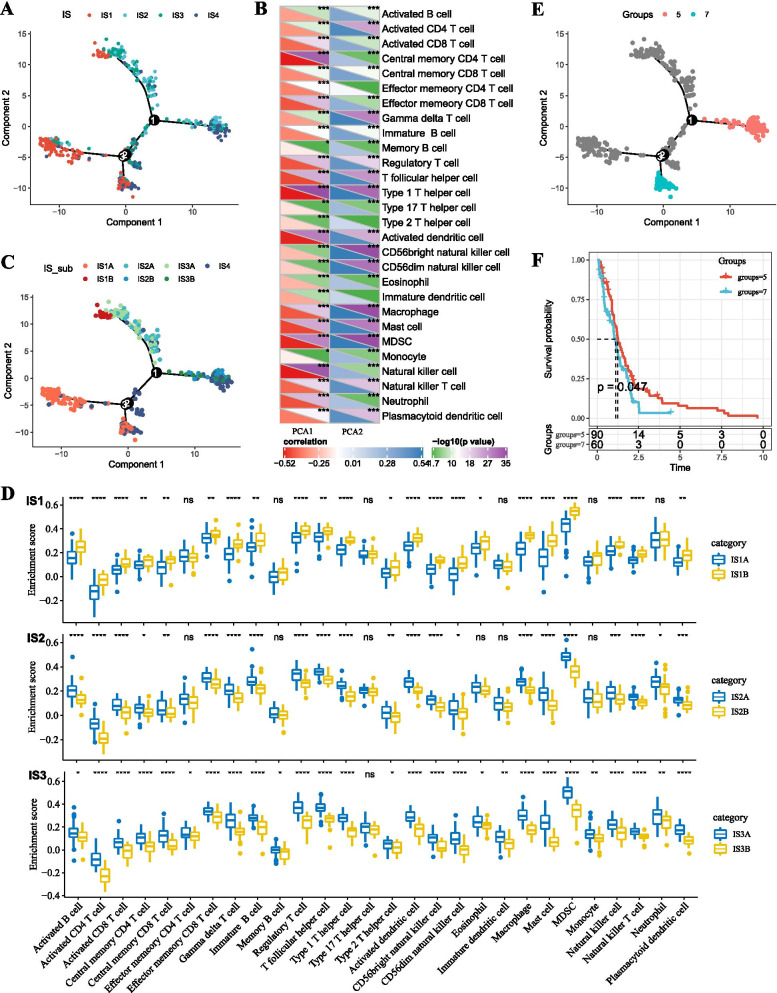
**A** The immune landscape in glioblastoma. Each point represents a sample, and different colors represent different molecular subtypes. The horizontal axis represents the first principal component, and the vertical axis represents the second principal component. **B** The correlation heatmaps between two principal components and 28 kinds of immune cells. **C** Immune landscape in glioblastoma and four immune molecular subtypes. **D** Immune landscape in glioblastoma and samples from two different locations. **E** Immune landscape in glioblastoma. **F** Prognostic differences in samples from different locations in the glioma immune landscape

To reveal the internal structure of the immune system and visualize the distribution of individual patients, we applied the Graph Structure Learning method for dimension reduction to the gene expression profiles. The analysis plotted individual patients into a graph with a sparse tree data structure and defined the GBM-associated immune landscape. The patient’s location represented the overall characteristics of the corresponding tumor microenvironment subtype (Fig. 6A). The horizontal coordinates were highly correlated with a variety of immune cells (Fig. 6B). The horizontal coordinates were most relevant to “Type 1 T helper cells”, “Natural killer cells”, “Central memory CD4 T cells”, and “Activated dendritic cells”. In contrast, the vertical coordinates were most related to “CD56^bright^ natural killer cell”, “MDSC”, and “Macrophage”, and IS1 was distributed at vertical opposite ends of the immune landscape, indicating significant intra-class heterogeneity in the subtypes. According to the position in the immune landscape, IS1 could be further divided into 2 subtypes (Fig. 6C), and, notably, these subtypes showed a specific immune expression pattern (Fig. 6D). Furthermore, different positions in the immune landscape had different prognostic features, and immune landscape analysis provided a further complement to the previously defined immune subtypes.

### Identification of co-expression modules of immune genes

**Fig. 7:**
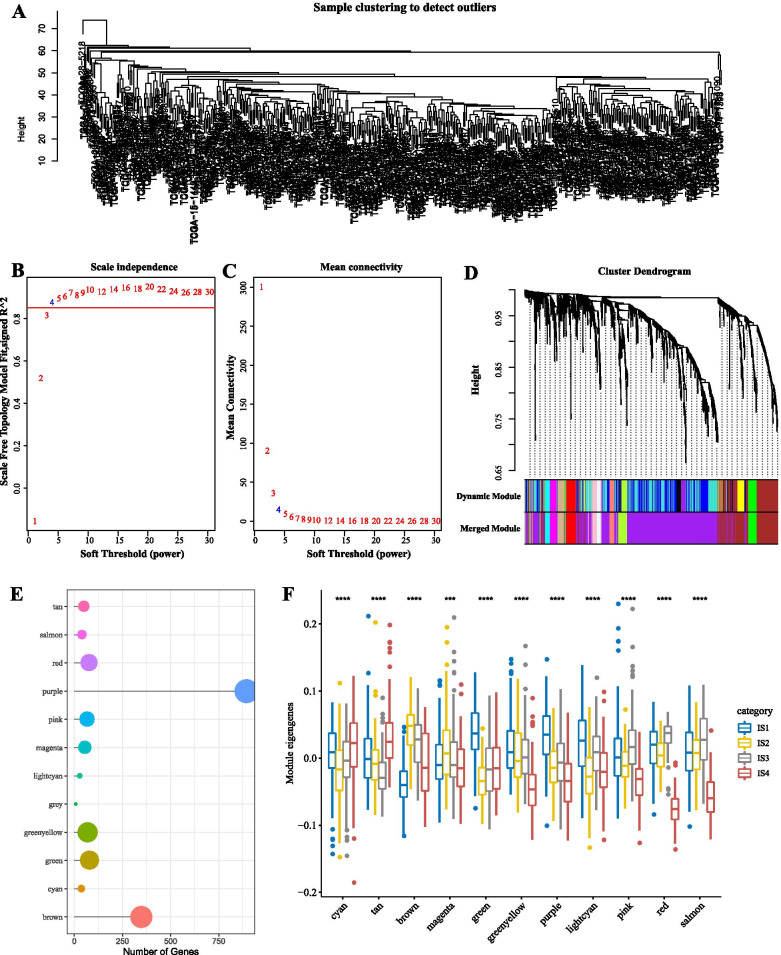
**A** Cluster analysis of samples. **B-C** Analysis of network topology for various soft-thresholding powers. **D** Gene dendrogram and module colors. **E** Genes statistics of modules. **F** Distribution of eigenvectors of modules in immune molecular subtypes. The grey module displayed a collection of the genes that could not be merged

Next, we used the R software package “WGCNA” (Weighted Correlation Network Analysis) to identify the co-expression modules of these immune genes. The samples were first clustered (Fig. 7A), and then the soft threshold was set to 4 to screen the co-expression modules. The co-expression network is a scale-free network, namely logarithm of the node with connectivity degree k (log (k)), that is negatively correlated with the logarithm of the probability of occurrence of the node (log(P(k))), and the correlation coefficient is greater than 0.85. To ensure that the network was a scale-free network, we chose β = 4 (Fig. 7B and C). Subsequently, we transformed the expression matrix into the adjacency matrix and then into the topological matrix. The average linkage hierarchical clustering was performed by the topological overlap matrix (TOM)-based dissimilarity measure. According to the criteria of the Dynamic Hybrid Tree Cut algorithm to cut the hierarchal clustering tree, the minimum number of genes per gene network module was set to 20. After the gene module was determined by the Dynamic Tree Cut, the eigenvectors of each module were calculated in turn. Then, we clustered the modules and merged the near modules into new modules (height = 0.25, deepSplit = 4, minModuleSize = 20). Finally, we gained 12 modules (Fig. 7D), and the gene statistics of each module are shown in Fig. 7E, in which 1683 genes were assigned to 12 co-expression modules. We calculated the distribution of the eigenvectors of the 11 modules in the four immune molecular subtypes, as shown in Fig. 7F. The eigenvectors of these 11 modules were significantly different in four molecular subtypes, and the eigenvectors of IS1 in cyan, tan, brown modules were significantly lower than IS4. The eigenvectors of IS1 in green, green-yellow, purple, light cyan, pink, red, and salmon modules were significantly higher than IS4.

### Function and prognostic analysis of co-expression modules of immune genes

**Fig. 8:**
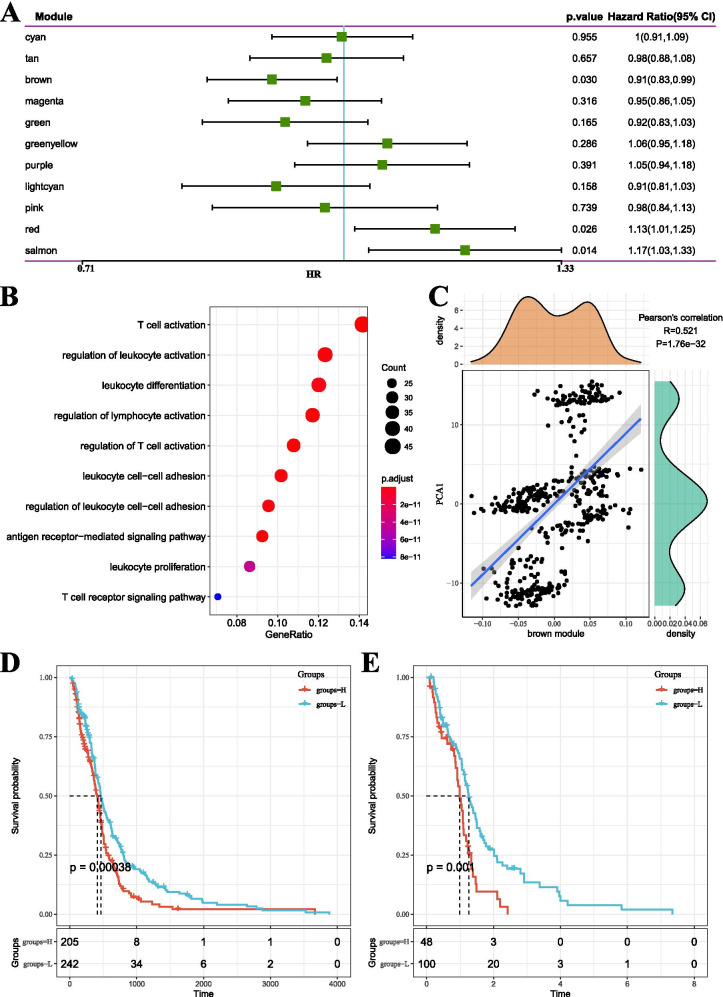
**A** The result of univariate analysis of module eigenvector genes. **B** The result of Gene Enrichment Analysis of Brown Module. **C** The correlation between the eigenvector of the brown module and the first principal component in the immune landscape. **D** The KM survival curve distribution of patients was grouped according to the module eigenvector genes expression from the brown module in the TCGA-GBM Agilent cohort. **E** The KM survival curve distribution of patients was grouped according to the module eigenvector genes expression from the brown module in the TCGA-GBM HiSeq cohort

We identified 11 immune-related gene modules (Supplementary Table 6) and found a significant correlation between the modules and GBM prognosis (Fig. 8A). High scores in the brown module predicted a better prognosis, while high scores in the red and salmon modules predicted a poor prognosis. Function Enrichment analysis showed that the brown module was associated with immune processes, such as T cell activation and T cell receptor signaling pathway (Fig. 8B), and this was highly positively correlated with the first principal component in the immune landscape (Fig. 8C). Then, the genes with a correlation coefficient greater than 0.8 with the brown module from the TCGA-GBM Agilent were extracted, and the univariate Cox proportional hazard regression analysis was carried out with *P* < 0.05.

We extracted 12 genes with a difference. The correlation between genes in the brown module and eigenvectors of the module, and the result of univariate analysis of module genes is shown in Supplementary Table 7. Furthermore, the stepAIC method in the MASS package was used for stepwise regression analysis, and the AIC information criterion was used to reduce 11 genes to 3 genes: CD1A, CD1E, and IL23R. The final 3-gene signature formula was as follows: RiskScore =—0.2363417*CD1A + 0.2073704*CD1E—0.2120154*IL23R.

We calculated a risk score for each sample according to its expression level. The Zscore method was applied to the pre-processing of Riskscore, and the samples were divided into two groups: high-risk (Riskscore > 0; 205 samples) low-risk (Riskscore < 0; 242 samples) groups. The KM curve was plot as shown in Fig. 8D (*P* = 0.00038). Similarly, there were also significant differences in the prognosis (Fig. 8E). Finally, three genes, CD1A, CD1E, and IL23R, were selected as module eigengenes, which had a significant prognostic significance and had a correlation coefficient greater than 0.8 with brown module genes.

## Discussion

Aggressive glioblastomas are a significant threat to public health. Due to its characteristics, treatment options are scarce, and thus new treatments are urgently needed. While checkpoint inhibitors have revolutionized cancer treatment, these therapies had little benefit for GBM patients. The results of several recently published clinical trials have been discouraging, possibly due to the poor immunogenicity of GBM tumors [26, 27]. In this study, we defined four GBM immune subtypes and identified and validated immune-related GBM molecular markers, which may help better predict the outcome of immunotherapy.

Glioblastomas typically have a low tumor mutation burden (TMB) and a highly immunosuppressed microenvironment, both associated with resistance to immunotherapy. However, in some gliomas, a high mutation burden was found [28, 29], but little was known about the mechanism leading to hypermutation and whether this could predict the response to immunotherapy. Mehdi and associates [30] performed a comprehensive analysis of the tumor mutation burden and determinants of the molecular signature of 10,294 glioblastomas, showing that chemotherapy could obtain mutants while it could not promote response to PD-1 inhibitors. Some studies demonstrated that some GBM patients with high TMB might benefit from PD-1 inhibitors [31], and a small fraction of pediatric GBM presenting a very high TMB may be sensitive to immune checkpoint suppression [32]. These studies supported TMB as a potential biomarker to identify GBM patients who may benefit from ICI therapy. Notably, in our study, TMB in IS1 with a poor prognosis was significantly lower than in IS3, and TMB in IS3 was lower than in IS4 with a good prognosis, indicating that the immune subtype can predict immunotherapy efficacy.

Recent advances in microarray and next-generation sequencing technologies have begun to reveal the complete picture of the GBM genome and improved understanding of the key molecules that drive GBM. Several studies of the Cancer Genome Atlas (TCGA) project defined the core of recurrent genomic changes of GBM [33, 34]. Mutations in the *TP53* gene (p53 protein) were among the most common mutations found in GBM, with a frequency of more than 65% in secondary GBM, and it may occur early with IDH1 mutation [35, 36]. The prevalence of p53 mutations was 54, 32, 21, and 0% among proneural, mesenchymal, neural, and classical subtypes of different GBM, respectively [37].

Besides, EGFR changes have been detected in more than half of GBM samples using genomic analysis. Nevertheless, the EGFR targeting strategy has failed in clinical trials [38, 39]. Instead, PTEN mutation enrichment was found in non-responding patients associated with immunosuppressive gene expression signatures. On the other hand, we found MAPK pathway alterations (PTPN11, BRAF) enriched in responder patients [40]. Our study extracted the expression profiles of IDH1, IDH2, MGMT, TERT, EGFR, PTEN, TP53, BRAF, CDKN2A genes and analyzed their differential distribution to each immune subtype. The results showed that the expression of all the genes except BRAF was significantly different in different immune subtypes in the TCGA-GBM Agilent cohort, indicating that our immune subtypes were closely related to the driving genes. The potential of these genes as predictive biomarkers for therapeutic responses in different immune subtypes needs further investigation.

A recent study showed that the expression of PD-L1 in GBM cells harmed the prognosis of patients [41]. In our study, significant differences were found in the expression of immune checkpoint genes in various subtypes, which may contribute to different clinical progression of the disease. Checkpoint inhibitors can also change the glioblastoma microenvironment, highlighting the value of immunotherapy for GBM. For instance, neoadjuvant nivolumab resulted in increased expression of chemokine transcripts, immune cell infiltration, and diversity of TCR clones between tumor-infiltrating T lymphocytes, supporting the local immunomodulatory effect of treatment [27]. In our study, we found differences in the composition of immune cells in different immune subtypes. For example, the “Type 2 T helper cell” in IS1 was significantly lower than in IS4, “Effector memory CD4 T cell” and “Immature B cell” were significantly higher than those in IS4, which indicated that the poor prognosis of GBM might be related to the activation of “Effector memory CD4 T cell”, “Immature B cell” and the inhibition of “Type 2 T helper cell”. Besides, we identified 11 of the most significant immune-related features by assessing the correlation between the immunophenotype. In addition, 56 previously defined immune molecular features, the highest levels of Leukocyte Fraction, Stromal Fraction, Macrophage Regulation, Lymphocyte Infiltration Signature Score, IFN-gamma Response, TGF-beta Response, and TCR Shannon were found in IS1 subtype. At the same time, Fraction Altered Homologous Recombination Defects and Th2 Cells were significantly higher in IS4 than those in IS1, IS2, and IS3. To understand the biological significance of these immune subtypes, Function Enrichment analysis was applied to demostrate that the brown module was associated with immune processes such as T cell activation and T cell receptor signaling pathway. Elena Anghileri and others had also demonstrated that abundant T-cell infiltration contributed to a durable clinical benefit in the treatment of GBM, sustained by a persistent and robust immune response during anti-PD1 therapy [42].

At last, CD1A, CD1E, and IL23R were selected as the final feature genes. CD1 molecules bind to T cells and present lipid-based antigens. In humans, there are three classes of CD1 molecules with nonredundant functions: Group 1 (CD1a, CD1b, CD1c), Group 2 (CD1d), and Group 3 (CD1e). These molecules have different expression patterns. For instance, CD1a is expressed in large quantities in Langerhans cells. Kim et al. demonstrated that CD1a was a lipid receptor for urushiol and mediated CD4 + T cell-driven skin inflammation, producing cytokines IL-17 and Il-22 in Langerhans cells. They further demonstrated that CD1 was a vital amplifier of an inflammatory response mediated by Th17 cells in psoriasis patients. Therefore, CD1a may be a potential therapeutic target for inflammatory dermatitis. A different study evaluated the relationship between inflammatory and metabolic signals in monocytes expressing CD1a in vitro and in vivo. This study suggested that monocytes expressing CD1a may be sensors and mediators of ulcerative colitis inflammation, and that CD1a could be a potential therapeutic target for the disease. Some species express CD1e, a special CD1 subtype with unknown functions. Unlike other CD1 proteins, CD1e is not transported across the surface of DC cells, and therefore cannot mediate antigen presentation to T cells. CD1E is an intracellular protein that exists in a soluble form inside the late endosomes or lysosomes [43, 44]. Studies have shown that CD1e helps expand the repertoire of glycolipid T cell antigens to optimize antimicrobial immune responses [45]. At present, there is no study on the relationship of CD1 with glioma. The interleukin 23 receptor (IL-23R), a proinflammatory cytokine receptor family member, is highly expressed in tumor tissue to induce local inflammation and promote tumor development. Many studies have shown that IL-23R plays a crucial role in tumorigenesis and cancer development in different types of cancer, such as esophageal cancer, colorectal cancer, bladder cancer, breast cancer, and laryngeal cancer. However, the role of IL-23R in glioblastoma remains unknown [46–50].

In this study, we conducted a multi-cohort retrospective study to identify four replicable immune subtypes of GBM and applied independent data for subtype verification and comprehensive molecular identification. Although four immune subtypes were explored and validated in two independent datasets, our study had some limitations. These conclusions were from a retrospective study, so functional experiments are needed in the future to explore the molecular function of these biomarkers in four immune subtypes. In addition, this study only focused on microarray expression datasets. Therefore, it is necessary to validate these findings in tissue samples or in clinical patients in order to elucidate the mechanisms of these targets. Future studies should further elucidate the complex relationships among immune subtypes, immunotherapeutic sensitivity, and molecular immune markers. Immune landscape analysis provided a further complement to the previously defined immune subtypes. Overall, this study provided some potential molecular targets for developing new immunotherapies that may ultimately lead to individualized therapies for GBM patient populations.

## Supplementary Information

**Additional file 1.**

**Additional file 2.**

**Additional file 3.**

**Additional file 4.**

**Additional file 5.**

**Additional file 6.**

**Additional file 7.**
