# Supplementary material for: Identification of glioblastoma immune subtypes and immune landscape based on a large cohort
Source: Hereditas. 2021 Aug 19;158:30. doi: 10.1186/s41065-021-00193-x (PMC8377979; doi:10.1186/s41065-021-00193-x)
Supplement: Supplementary file 1 — Additional file 1. [file 41065_2021_193_MOESM1_ESM.docx]

Samples OS.time OS DSS.time DSS DFI.time DFI PFI.time PFI Age Gender histological_type Stage Grade

TCGA-06-2567 133 1 133 1 NA NA 133 1 65 MALE Untreated primary (de novo) GBM

TCGA-12-0703 620 1 620 1 NA NA 208 1 46 MALE Untreated primary (de novo) GBM

TCGA-14-1821 541 1 541 1 NA NA 331 1 31 MALE Untreated primary (de novo) GBM

TCGA-08-0373 134 1 134 1 NA NA 134 1 69 MALE Untreated primary (de novo) GBM

TCGA-26-1438 305 0 305 0 NA NA 158 1 36 MALE Untreated primary (de novo) GBM

TCGA-14-1402 975 1 975 1 NA NA 333 1 58 FEMALE Untreated primary (de novo) GBM

TCGA-16-1045 883 1 883 1 NA NA 654 1 49 FEMALE Untreated primary (de novo) GBM

TCGA-19-1388 394 1 394 1 NA NA 148 1 58 MALE Untreated primary (de novo) GBM

TCGA-19-0963 434 1 434 1 NA NA 310 1 61 MALE Untreated primary (de novo) GBM

TCGA-14-0736 460 1 460 1 NA NA 128 1 49 MALE Untreated primary (de novo) GBM

TCGA-19-1387 181 0 181 0 NA NA 79 1 81 MALE Untreated primary (de novo) GBM

TCGA-14-1452 216 1 216 1 NA NA 172 1 60 MALE Untreated primary (de novo) GBM

TCGA-14-1823 543 1 543 1 NA NA 140 1 58 FEMALE Untreated primary (de novo) GBM

TCGA-06-0749 82 1 82 1 NA NA 82 1 50 MALE Untreated primary (de novo) GBM

TCGA-06-1086 208 1 208 1 NA NA 208 1 42 MALE Untreated primary (de novo) GBM

TCGA-12-0618 395 1 395 NA NA NA 395 0 49 MALE Untreated primary (de novo) GBM

TCGA-08-0525 486 1 486 1 NA NA 343 1 52 MALE Untreated primary (de novo) GBM

TCGA-12-1598 476 1 476 1 NA NA 476 1 75 FEMALE Untreated primary (de novo) GBM

TCGA-12-0692 111 1 111 1 NA NA 35 1 75 FEMALE Untreated primary (de novo) GBM

TCGA-02-0422 441 1 441 1 NA NA 125 1 50 MALE Untreated primary (de novo) GBM

TCGA-06-2564 181 0 181 0 NA NA 181 0 50 MALE Untreated primary (de novo) GBM

TCGA-02-0004 345 1 345 1 NA NA 314 1 59 MALE Untreated primary (de novo) GBM

TCGA-06-0179 616 1 616 1 NA NA 250 1 64 MALE Untreated primary (de novo) GBM

TCGA-06-1801 360 1 360 1 NA NA 232 1 56 FEMALE Untreated primary (de novo) GBM

TCGA-14-0871 880 1 880 NA NA NA 880 0 74 FEMALE Untreated primary (de novo) GBM

TCGA-12-3649 463 1 463 1 NA NA 351 1 76 MALE Untreated primary (de novo) GBM

TCGA-28-1749 280 0 280 0 NA NA 280 1 73 MALE Untreated primary (de novo) GBM

TCGA-08-0358 678 1 678 1 NA NA 264 1 50 MALE Untreated primary (de novo) GBM

TCGA-14-0787 68 1 68 NA NA NA 68 0 69 MALE Untreated primary (de novo) GBM

TCGA-06-0162 104 1 104 1 NA NA 104 1 47 FEMALE Untreated primary (de novo) GBM

TCGA-12-1092 661 1 661 1 NA NA 637 1 58 MALE Untreated primary (de novo) GBM

TCGA-12-1094 372 1 372 1 NA NA 186 1 56 MALE Untreated primary (de novo) GBM

TCGA-28-1747 77 1 77 NA NA NA 77 0 44 MALE Untreated primary (de novo) GBM

TCGA-08-0345 53 1 53 1 NA NA 53 1 71 FEMALE Untreated primary (de novo) GBM

TCGA-02-0260 515 1 515 1 NA NA 515 1 54 MALE Untreated primary (de novo) GBM

TCGA-27-2519 550 1 550 1 NA NA 256 1 48 MALE Untreated primary (de novo) GBM

TCGA-06-0412 291 1 291 1 NA NA 130 1 56 FEMALE Untreated primary (de novo) GBM

TCGA-27-1835 648 1 648 1 NA NA 157 1 53 FEMALE Untreated primary (de novo) GBM

TCGA-02-0440 345 1 345 1 NA NA 212 1 62 MALE Untreated primary (de novo) GBM

TCGA-27-2521 510 1 510 1 NA NA 510 1 34 MALE Untreated primary (de novo) GBM

TCGA-12-1599 781 1 781 1 NA NA 89 1 47 FEMALE Untreated primary (de novo) GBM

TCGA-06-1087 123 1 123 1 NA NA 109 1 75 MALE Untreated primary (de novo) GBM

TCGA-06-0397 274 1 274 1 NA NA 274 1 57 FEMALE Untreated primary (de novo) GBM

TCGA-08-0356 946 1 946 1 NA NA 447 1 59 FEMALE Untreated primary (de novo) GBM

TCGA-06-0152 375 1 375 1 NA NA 299 1 68 MALE Untreated primary (de novo) GBM

TCGA-06-0645 175 1 175 1 NA NA 175 1 55 FEMALE Untreated primary (de novo) GBM

TCGA-19-0957 666 1 666 1 NA NA 50 1 48 FEMALE Untreated primary (de novo) GBM

TCGA-12-0776 296 1 296 1 NA NA 127 1 52 MALE Untreated primary (de novo) GBM

TCGA-15-1449 404 1 404 1 NA NA 74 1 65 MALE Untreated primary (de novo) GBM

TCGA-28-1751 232 0 232 0 NA NA 219 1 61 FEMALE Untreated primary (de novo) GBM

TCGA-12-3651 386 1 386 1 NA NA 358 1 77 MALE Untreated primary (de novo) GBM

TCGA-12-3650 333 1 333 1 NA NA 239 1 46 MALE Untreated primary (de novo) GBM

TCGA-19-0955 358 0 358 0 NA NA 358 0 71 MALE Untreated primary (de novo) GBM

TCGA-28-1745 345 0 345 0 NA NA 317 1 76 MALE Untreated primary (de novo) GBM

TCGA-06-0409 2201 1 2201 1 NA NA 334 1 43 MALE Untreated primary (de novo) GBM

TCGA-19-1787 385 1 385 1 NA NA 308 1 48 MALE Untreated primary (de novo) GBM

TCGA-15-1446 581 0 581 0 NA NA 443 1 55 MALE Untreated primary (de novo) GBM

TCGA-14-1453 35 1 35 1 NA NA 35 1 69 MALE Untreated primary (de novo) GBM

TCGA-19-2620 148 1 148 0 NA NA 148 0 70 MALE Untreated primary (de novo) GBM

TCGA-06-0649 64 1 64 1 NA NA 64 1 73 FEMALE Untreated primary (de novo) GBM

TCGA-12-1597 675 1 675 1 NA NA 180 1 62 FEMALE Untreated primary (de novo) GBM

TCGA-02-0337 764 1 764 1 NA NA 691 1 48 MALE Untreated primary (de novo) GBM

TCGA-02-0285 422 1 422 1 NA NA 422 1 50 FEMALE Untreated primary (de novo) GBM

TCGA-27-2527 438 1 438 1 NA NA 438 1 81 MALE Untreated primary (de novo) GBM

TCGA-02-0332 782 1 782 1 NA NA 438 1 46 FEMALE Untreated primary (de novo) GBM

TCGA-14-0865 502 1 502 1 NA NA 215 1 67 MALE Untreated primary (de novo) GBM

TCGA-12-1088 3881 1 3881 NA NA NA 3881 0 53 FEMALE Untreated primary (de novo) GBM

TCGA-12-3646 1339 1 1339 1 NA NA 1315 1 59 FEMALE Untreated primary (de novo) GBM

TCGA-27-1836 914 1 914 1 NA NA 172 1 33 FEMALE Untreated primary (de novo) GBM

TCGA-08-0524 221 1 221 1 NA NA 61 1 17 FEMALE Untreated primary (de novo) GBM

TCGA-08-0244 690 1 690 1 NA NA 484 1 62 MALE Untreated primary (de novo) GBM

TCGA-12-0691 369 1 369 1 NA NA 98 1 63 MALE Untreated primary (de novo) GBM

TCGA-14-0790 419 1 419 1 NA NA 419 1 64 FEMALE Untreated primary (de novo) GBM

TCGA-32-2616 224 1 224 1 NA NA 182 1 48 FEMALE Untreated primary (de novo) GBM

TCGA-41-2573 272 0 272 0 NA NA 167 1 59 MALE Untreated primary (de novo) GBM

TCGA-14-3477 115 0 115 0 NA NA 115 0 38 FEMALE Untreated primary (de novo) GBM

TCGA-12-1095 482 1 482 1 NA NA 96 1 65 FEMALE Untreated primary (de novo) GBM

TCGA-08-0517 1785 1 1785 1 NA NA 1024 1 34 FEMALE Untreated primary (de novo) GBM

TCGA-08-0522 635 1 635 1 NA NA 266 1 61 MALE Untreated primary (de novo) GBM

TCGA-19-2621 33 1 33 NA NA NA 33 0 83 MALE Untreated primary (de novo) GBM

TCGA-14-0812 99 1 99 1 NA NA 99 1 65 MALE Untreated primary (de novo) GBM

TCGA-14-1458 203 1 203 NA NA NA 81 1 54 MALE Untreated primary (de novo) GBM

TCGA-08-0360 468 1 468 1 NA NA 112 1 76 MALE Untreated primary (de novo) GBM

TCGA-02-0451 493 1 493 1 NA NA 428 1 62 FEMALE Untreated primary (de novo) GBM

TCGA-02-2470 393 1 393 1 NA NA 183 1 57 MALE Treated primary GBM

TCGA-02-0317 372 1 372 1 NA NA 238 1 40 MALE Untreated primary (de novo) GBM

TCGA-08-0510 130 1 130 1 NA NA 90 1 75 MALE Untreated primary (de novo) GBM

TCGA-02-0048 98 1 98 NA NA NA 23 1 80 MALE Untreated primary (de novo) GBM

TCGA-14-0817 164 1 164 NA NA NA 164 0 69 FEMALE Untreated primary (de novo) GBM

TCGA-19-2619 294 0 294 0 NA NA 146 1 55 FEMALE Untreated primary (de novo) GBM

TCGA-28-1753 37 0 37 0 NA NA 37 0 53 MALE Untreated primary (de novo) GBM

TCGA-08-0349 298 1 298 1 NA NA 93 1 46 MALE Untreated primary (de novo) GBM

TCGA-32-1982 142 1 142 1 NA NA 142 1 76 FEMALE Untreated primary (de novo) GBM

TCGA-12-0615 467 1 467 1 NA NA 160 1 78 FEMALE Untreated primary (de novo) GBM

TCGA-06-0875 1322 0 1322 0 NA NA 279 1 61 FEMALE Untreated primary (de novo) GBM

TCGA-12-0656 2883 1 2883 1 NA NA 1467 1 48 FEMALE Untreated primary (de novo) GBM

TCGA-08-0516 596 1 596 1 NA NA 40 1 14 MALE Untreated primary (de novo) GBM

TCGA-08-0390 425 1 425 1 NA NA 425 1 69 MALE Untreated primary (de novo) GBM

TCGA-14-1454 918 1 918 1 NA NA 291 1 54 FEMALE Untreated primary (de novo) GBM

TCGA-12-0820 562 1 562 1 NA NA 272 1 40 MALE Untreated primary (de novo) GBM

TCGA-02-0111 705 1 705 1 NA NA 74 1 56 MALE Untreated primary (de novo) GBM

TCGA-06-0745 239 1 239 1 NA NA 92 1 59 MALE Untreated primary (de novo) GBM

TCGA-06-0877 204 0 204 0 NA NA 204 0 78 MALE Untreated primary (de novo) GBM

TCGA-02-0333 133 1 133 1 NA NA 4 1 77 FEMALE Untreated primary (de novo) GBM

TCGA-14-1827 179 1 179 NA NA NA 179 0 51 MALE Untreated primary (de novo) GBM

TCGA-06-2570 958 0 958 0 NA NA 958 0 21 FEMALE Untreated primary (de novo) GBM

TCGA-14-1825 232 1 232 1 NA NA 82 1 70 MALE Untreated primary (de novo) GBM

TCGA-12-0616 448 1 448 1 NA NA 398 1 36 FEMALE Untreated primary (de novo) GBM

TCGA-02-0432 1433 1 1433 1 NA NA 1053 1 36 MALE Untreated primary (de novo) GBM

TCGA-02-0290 485 1 485 1 NA NA 374 1 49 MALE Untreated primary (de novo) GBM

TCGA-15-1447 1618 0 1618 0 NA NA 340 1 36 FEMALE Untreated primary (de novo) GBM

TCGA-12-0818 2791 1 2791 NA NA NA 2791 0 45 FEMALE Untreated primary (de novo) GBM

TCGA-27-1832 300 1 300 1 NA NA 176 1 59 FEMALE Untreated primary (de novo) GBM

TCGA-12-0775 232 1 232 1 NA NA 94 1 75 FEMALE Untreated primary (de novo) GBM

TCGA-08-0380 454 1 454 1 NA NA 151 1 74 FEMALE Untreated primary (de novo) GBM

TCGA-06-0876 1405 0 1405 0 NA NA 424 1 72 FEMALE Untreated primary (de novo) GBM

TCGA-19-1385 327 1 327 1 NA NA 177 1 69 MALE Untreated primary (de novo) GBM

TCGA-02-0023 612 1 612 1 NA NA 435 1 38 FEMALE Untreated primary (de novo) GBM

TCGA-06-0182 111 1 111 1 NA NA 111 1 76 MALE Untreated primary (de novo) GBM

TCGA-19-1788 112 1 112 NA NA NA 112 0 39 MALE Untreated primary (de novo) GBM

TCGA-28-2506 205 0 205 0 NA NA 84 1 63 FEMALE Untreated primary (de novo) GBM

TCGA-12-0828 272 1 272 1 NA NA 13 1 57 MALE Untreated primary (de novo) GBM

TCGA-06-0747 82 1 82 1 NA NA 82 1 53 MALE Untreated primary (de novo) GBM

TCGA-02-0456 102 1 102 1 NA NA 12 1 67 FEMALE Untreated primary (de novo) GBM

TCGA-12-0821 323 1 323 1 NA NA 259 1 62 MALE Untreated primary (de novo) GBM

TCGA-08-0347 782 1 782 1 NA NA 59 1 50 MALE Untreated primary (de novo) GBM

TCGA-16-1055 313 1 313 1 NA NA 288 1 48 MALE Untreated primary (de novo) GBM

TCGA-02-0015 627 1 627 1 NA NA 456 1 50 MALE Untreated primary (de novo) GBM

TCGA-08-0359 103 1 103 1 NA NA 103 1 59 FEMALE Untreated primary (de novo) GBM

TCGA-02-2486 618 1 618 1 NA NA 618 1 64 MALE Untreated primary (de novo) GBM

TCGA-12-0819 754 1 754 1 NA NA 428 1 49 FEMALE Untreated primary (de novo) GBM

TCGA-06-1802 466 1 466 1 NA NA 81 1 61 MALE Untreated primary (de novo) GBM

TCGA-16-1063 425 1 425 1 NA NA 369 1 72 MALE Untreated primary (de novo) GBM

TCGA-27-1830 154 1 154 1 NA NA 124 1 57 MALE Untreated primary (de novo) GBM

TCGA-08-0511 235 1 235 1 NA NA 235 1 69 MALE Untreated primary (de novo) GBM

TCGA-08-0512 1282 1 1282 1 NA NA 231 1 48 MALE Untreated primary (de novo) GBM

TCGA-06-0413 96 1 96 1 NA NA 96 1 77 FEMALE Untreated primary (de novo) GBM

TCGA-06-0744 1426 1 1426 1 NA NA 1277 1 66 MALE Untreated primary (de novo) GBM

TCGA-14-2555 242 0 242 0 NA NA 242 0 44 FEMALE Untreated primary (de novo) GBM

TCGA-19-0960 262 0 262 0 NA NA 262 0 83 FEMALE Untreated primary (de novo) GBM

TCGA-06-2559 150 1 150 1 NA NA 150 1 83 MALE Untreated primary (de novo) GBM

TCGA-08-0350 889 1 889 1 NA NA 439 1 32 MALE Untreated primary (de novo) GBM

TCGA-02-0325 323 1 323 1 NA NA 284 1 61 MALE Untreated primary (de novo) GBM

TCGA-19-2625 124 1 124 0 NA NA 124 0 76 FEMALE Untreated primary (de novo) GBM

TCGA-02-0266 539 1 539 1 NA NA 293 1 14 MALE Untreated primary (de novo) GBM

TCGA-27-1837 427 1 427 1 NA NA 136 1 36 MALE Untreated primary (de novo) GBM

TCGA-16-0861 131 0 131 NA NA NA 94 1 65 MALE Untreated primary (de novo) GBM

TCGA-12-0827 1179 1 1179 1 NA NA 1179 1 38 FEMALE Untreated primary (de novo) GBM

TCGA-12-1097 442 1 442 1 NA NA 166 1 61 MALE Untreated primary (de novo) GBM

TCGA-02-0070 762 0 762 0 NA NA 762 1 70 MALE Untreated primary (de novo) GBM

TCGA-28-1760 143 0 143 0 NA NA 105 1 48 MALE Untreated primary (de novo) GBM

TCGA-06-1800 815 1 815 1 NA NA 750 1 61 MALE Untreated primary (de novo) GBM

TCGA-12-1093 486 1 486 1 NA NA 322 1 66 FEMALE Untreated primary (de novo) GBM

TCGA-12-1091 1010 1 1010 1 NA NA 56 1 10 FEMALE Untreated primary (de novo) GBM

TCGA-28-1752 258 0 258 0 NA NA 258 0 42 FEMALE Untreated primary (de novo) GBM

TCGA-16-1060 278 1 278 1 NA NA 111 1 70 FEMALE Untreated primary (de novo) GBM

TCGA-14-0786 701 1 701 1 NA NA 127 1 50 FEMALE Untreated primary (de novo) GBM

TCGA-27-2528 480 1 480 1 NA NA 72 1 62 MALE Untreated primary (de novo) GBM

TCGA-27-1834 1233 1 1233 1 NA NA 335 1 56 MALE Untreated primary (de novo) GBM

TCGA-06-2565 506 1 506 1 NA NA 178 1 59 MALE Untreated primary (de novo) GBM

TCGA-12-1090 231 1 231 1 NA NA 231 1 58 MALE Untreated primary (de novo) GBM

TCGA-02-0068 804 1 804 1 NA NA 175 1 57 MALE Untreated primary (de novo) GBM

TCGA-32-2638 766 1 766 1 NA NA 766 1 67 MALE Untreated primary (de novo) GBM

TCGA-27-1838 350 1 350 1 NA NA 119 1 59 FEMALE Untreated primary (de novo) GBM

TCGA-02-0324 235 1 235 1 NA NA 164 1 69 FEMALE Untreated primary (de novo) GBM

TCGA-14-1795 60 1 60 1 NA NA 60 1 66 MALE Untreated primary (de novo) GBM

TCGA-02-0016 2648 1 2648 1 NA NA 1699 1 50 MALE Untreated primary (de novo) GBM

TCGA-28-1755 47 1 47 1 NA NA 47 1 52 FEMALE Untreated primary (de novo) GBM

TCGA-27-2524 231 1 231 1 NA NA 231 1 56 MALE Untreated primary (de novo) GBM

TCGA-02-0446 282 1 282 1 NA NA 15 1 61 MALE Untreated primary (de novo) GBM

TCGA-02-0087 1757 0 1757 0 NA NA 1757 1 27 FEMALE Untreated primary (de novo) GBM

TCGA-14-1401 114 1 114 1 NA NA 114 1 54 MALE Untreated primary (de novo) GBM

TCGA-12-3648 819 1 819 1 NA NA 86 1 61 FEMALE Untreated primary (de novo) GBM

TCGA-08-0357 1143 1 1143 1 NA NA 155 1 49 MALE Untreated primary (de novo) GBM

TCGA-12-3653 442 1 442 1 NA NA 224 1 34 FEMALE Untreated primary (de novo) GBM

TCGA-14-0867 62 1 62 NA NA NA 62 0 67 MALE Untreated primary (de novo) GBM

TCGA-06-2561 537 1 537 1 NA NA 78 1 53 FEMALE Untreated primary (de novo) GBM

TCGA-08-0348 370 1 370 1 NA NA 198 1 63 MALE Untreated primary (de novo) GBM

TCGA-02-0106 355 1 355 1 NA NA 196 1 54 MALE Untreated primary (de novo) GBM

TCGA-12-0822 715 1 715 1 NA NA 91 1 46 MALE Untreated primary (de novo) GBM

TCGA-14-1456 1246 0 1246 0 NA NA 98 1 23 MALE Untreated primary (de novo) GBM

TCGA-12-0662 1161 1 1161 1 NA NA 600 1 51 MALE Untreated primary (de novo) GBM

TCGA-14-1451 703 1 703 1 NA NA 19 1 39 MALE Untreated primary (de novo) GBM

TCGA-06-0644 384 1 384 1 NA NA 85 1 71 MALE Untreated primary (de novo) GBM

TCGA-02-0258 503 1 503 1 NA NA 503 1 36 FEMALE Untreated primary (de novo) GBM

TCGA-06-0192 1185 1 1185 1 NA NA 648 1 58 MALE Untreated primary (de novo) GBM

TCGA-12-1089 177 1 177 1 NA NA 177 1 64 MALE Untreated primary (de novo) GBM

TCGA-27-1833 737 1 737 1 NA NA 469 1 67 FEMALE Untreated primary (de novo) GBM

TCGA-12-0772 1638 1 1638 1 NA NA 607 1 46 MALE Untreated primary (de novo) GBM

TCGA-08-0514 337 1 337 1 NA NA 337 1 69 FEMALE Untreated primary (de novo) GBM

TCGA-06-0879 1229 1 1229 1 NA NA 699 1 52 MALE Untreated primary (de novo) GBM

TCGA-12-1602 206 1 206 1 NA NA 140 1 58 MALE Untreated primary (de novo) GBM

TCGA-08-0389 467 1 467 1 NA NA 467 1 59 MALE Untreated primary (de novo) GBM

TCGA-06-1084 728 1 728 1 NA NA 208 1 54 MALE Untreated primary (de novo) GBM

TCGA-06-2558 380 1 380 NA NA NA 380 0 75 FEMALE Untreated primary (de novo) GBM

TCGA-02-0039 320 1 320 1 NA NA 102 1 54 MALE Untreated primary (de novo) GBM

TCGA-19-1789 99 1 99 NA NA NA 99 0 69 FEMALE Untreated primary (de novo) GBM

TCGA-32-2632 269 1 269 1 NA NA 269 1 80 MALE Untreated primary (de novo) GBM

TCGA-02-0104 1977 1 1977 1 NA NA 1457 1 29 FEMALE Untreated primary (de novo) GBM

TCGA-26-1799 285 1 285 1 NA NA 213 1 68 MALE Untreated primary (de novo) GBM

TCGA-02-0026 748 1 748 1 NA NA 462 1 27 MALE Untreated primary (de novo) GBM

TCGA-32-2615 485 1 485 1 NA NA 131 1 62 MALE Untreated primary (de novo) GBM

TCGA-28-2514 160 0 160 0 NA NA 160 0 45 MALE Untreated primary (de novo) GBM

TCGA-06-0686 432 1 432 1 NA NA 160 1 53 MALE Untreated primary (de novo) GBM

TCGA-12-0688 811 1 811 1 NA NA 228 1 74 MALE Untreated primary (de novo) GBM

TCGA-02-0084 384 1 384 NA NA NA 384 0 36 FEMALE Untreated primary (de novo) GBM

TCGA-08-0520 327 1 327 1 NA NA 105 1 70 MALE Untreated primary (de novo) GBM

TCGA-12-0773 1315 1 1315 1 NA NA 725 1 24 MALE Untreated primary (de novo) GBM

TCGA-02-0338 322 1 322 1 NA NA 168 1 41 MALE Untreated primary (de novo) GBM

TCGA-02-2483 466 0 466 0 NA NA 466 0 43 MALE Untreated primary (de novo) GBM

TCGA-12-0653 320 1 320 1 NA NA 267 1 65 MALE Untreated primary (de novo) GBM

TCGA-27-2523 489 1 489 1 NA NA 402 1 63 MALE Untreated primary (de novo) GBM

TCGA-06-0155 318 1 318 1 NA NA 256 1 61 MALE Untreated primary (de novo) GBM

TCGA-12-0769 458 1 458 1 NA NA 378 1 52 MALE Untreated primary (de novo) GBM

TCGA-19-1786 218 0 218 0 NA NA 218 0 64 FEMALE Untreated primary (de novo) GBM

TCGA-02-0271 440 1 440 NA NA NA 440 0 26 MALE Untreated primary (de novo) GBM

TCGA-06-0881 504 1 504 1 NA NA 141 1 50 MALE Untreated primary (de novo) GBM

TCGA-06-0194 142 1 142 1 NA NA 125 1 37 FEMALE Untreated primary (de novo) GBM

TCGA-02-0330 484 1 484 1 NA NA 120 1 51 FEMALE Untreated primary (de novo) GBM

TCGA-12-0670 790 1 790 NA NA NA 379 1 60 MALE Untreated primary (de novo) GBM

TCGA-06-0146 611 1 611 1 NA NA 530 1 33 FEMALE Untreated primary (de novo) GBM

TCGA-14-1459 378 1 378 1 NA NA 275 1 63 FEMALE Untreated primary (de novo) GBM

TCGA-06-0743 803 1 803 1 NA NA 176 1 69 MALE Untreated primary (de novo) GBM

TCGA-12-0619 1062 1 1062 1 NA NA 203 1 60 MALE Untreated primary (de novo) GBM

TCGA-28-2513 222 0 222 0 NA NA 75 1 69 FEMALE Untreated primary (de novo) GBM

TCGA-27-1831 505 1 505 1 NA NA 144 1 66 MALE Untreated primary (de novo) GBM

TCGA-14-2554 532 1 532 1 NA NA 532 1 52 FEMALE Untreated primary (de novo) GBM

TCGA-12-0780 452 1 452 1 NA NA 91 1 51 FEMALE Untreated primary (de novo) GBM

TCGA-06-0878 218 0 218 0 NA NA 66 1 74 MALE Untreated primary (de novo) GBM

TCGA-08-0352 39 1 39 1 NA NA 39 1 79 MALE Untreated primary (de novo) GBM

TCGA-12-1099 126 1 126 1 NA NA 62 1 68 FEMALE Untreated primary (de novo) GBM

TCGA-06-0648 298 1 298 1 NA NA 202 1 77 MALE Untreated primary (de novo) GBM

TCGA-02-0289 432 1 432 1 NA NA 245 1 57 MALE Untreated primary (de novo) GBM

TCGA-14-1037 587 1 587 1 NA NA 326 1 66 FEMALE Untreated primary (de novo) GBM

TCGA-14-0866 801 1 801 NA NA NA 97 1 55 MALE Untreated primary (de novo) GBM

TCGA-08-0518 588 1 588 1 NA NA 134 1 60 FEMALE Untreated primary (de novo) GBM

TCGA-14-1396 34 1 34 1 NA NA 34 1 78 FEMALE Untreated primary (de novo) GBM

TCGA-06-0177 127 1 127 1 NA NA 127 1 64 MALE Untreated primary (de novo) GBM

TCGA-06-2557 33 1 33 1 NA NA 33 1 76 MALE Untreated primary (de novo) GBM

TCGA-02-0269 327 1 327 1 NA NA 99 1 68 MALE Untreated primary (de novo) GBM

TCGA-08-0344 3524 1 3524 1 NA NA 1880 1 66 MALE Untreated primary (de novo) GBM

TCGA-27-2526 87 1 87 1 NA NA 87 1 79 FEMALE Untreated primary (de novo) GBM

TCGA-08-0351 1987 1 1987 1 NA NA 1542 1 40 MALE Untreated primary (de novo) GBM

TCGA-02-0430 321 1 321 1 NA NA 321 1 67 FEMALE Untreated primary (de novo) GBM

TCGA-12-1096 277 1 277 1 NA NA 270 1 55 MALE Untreated primary (de novo) GBM

TCGA-06-0646 175 1 175 1 NA NA 90 1 60 MALE Untreated primary (de novo) GBM

TCGA-19-2629 737 1 737 1 NA NA 145 1 60 MALE Untreated primary (de novo) GBM

TCGA-28-2509 145 0 145 0 NA NA 145 0 77 FEMALE Untreated primary (de novo) GBM

TCGA-02-2466 511 1 511 1 NA NA 128 1 61 MALE Treated primary GBM

TCGA-12-0654 231 1 231 1 NA NA 216 1 48 FEMALE Untreated primary (de novo) GBM

TCGA-12-1600 448 1 448 1 NA NA 404 1 86 MALE Untreated primary (de novo) GBM

TCGA-02-0321 301 1 301 1 NA NA 73 1 74 MALE Untreated primary (de novo) GBM

TCGA-16-0849 793 0 793 0 NA NA 793 0 54 MALE Untreated primary (de novo) GBM

TCGA-32-1986 386 1 386 1 NA NA 233 1 68 MALE Untreated primary (de novo) GBM

TCGA-28-1756 86 0 86 0 NA NA 86 0 78 MALE Untreated primary (de novo) GBM

TCGA-16-1460 195 0 195 0 NA NA 195 0 36 FEMALE Untreated primary (de novo) GBM

TCGA-08-0353 256 1 256 1 NA NA 164 1 58 MALE Untreated primary (de novo) GBM

TCGA-14-1034 485 1 485 1 NA NA 282 1 60 FEMALE Untreated primary (de novo) GBM

TCGA-14-0789 342 1 342 NA NA NA 105 1 54 MALE Untreated primary (de novo) GBM

TCGA-27-2518 753 1 753 1 NA NA 539 1 52 MALE Untreated primary (de novo) GBM

TCGA-02-0059 291 1 291 1 NA NA 21 1 68 FEMALE Untreated primary (de novo) GBM

TCGA-06-0414 1068 1 1068 0 NA NA 1013 1 63 MALE Untreated primary (de novo) GBM

TCGA-14-0783 189 1 189 1 NA NA 189 1 36 FEMALE Untreated primary (de novo) GBM

TCGA-02-2485 470 0 470 0 NA NA 186 1 53 MALE Untreated primary (de novo) GBM

TCGA-14-1829 218 0 218 0 NA NA 218 0 57 MALE Untreated primary (de novo) GBM

TCGA-08-0355 747 1 747 1 NA NA 519 1 30 FEMALE Untreated primary (de novo) GBM

TCGA-14-0813 41 1 41 NA NA NA 41 0 78 MALE Untreated primary (de novo) GBM

TCGA-06-0127 121 1 121 1 NA NA 91 1 67 MALE Untreated primary (de novo) GBM

TCGA-15-0742 419 1 419 NA NA NA 232 1 65 MALE Untreated primary (de novo) GBM

TCGA-12-0707 864 1 864 NA NA NA 864 0 55 MALE Untreated primary (de novo) GBM

TCGA-06-0216 735 1 735 1 NA NA 175 1 61 FEMALE Untreated primary (de novo) GBM

TCGA-08-0509 382 1 382 1 NA NA 382 1 63 MALE Untreated primary (de novo) GBM

TCGA-08-0529 560 1 560 1 NA NA 328 1 56 FEMALE Untreated primary (de novo) GBM

TCGA-12-0826 845 1 845 NA NA NA 845 0 38 FEMALE Untreated primary (de novo) GBM

TCGA-02-0326 223 1 223 1 NA NA 5 1 82 FEMALE Untreated primary (de novo) GBM

TCGA-02-0051 459 1 459 1 NA NA 89 1 43 MALE Untreated primary (de novo) GBM

TCGA-19-0964 103 0 103 0 NA NA 103 0 70 MALE Untreated primary (de novo) GBM

TCGA-16-1056 426 1 426 1 NA NA 239 1 55 MALE Untreated primary (de novo) GBM

TCGA-19-2623 229 0 229 0 NA NA 229 0 65 MALE Untreated primary (de novo) GBM

TCGA-02-0281 121 1 121 1 NA NA 121 1 78 FEMALE Untreated primary (de novo) GBM

TCGA-19-1386 276 1 276 1 NA NA 237 1 51 MALE Untreated primary (de novo) GBM

TCGA-08-0245 1151 1 1151 1 NA NA 463 1 31 FEMALE Untreated primary (de novo) GBM

TCGA-08-0521 146 1 146 1 NA NA 125 1 17 MALE Untreated primary (de novo) GBM

TCGA-08-0531 230 1 230 1 NA NA 230 1 64 MALE Untreated primary (de novo) GBM

TCGA-06-0164 1731 1 1731 1 NA NA 1428 1 47 MALE Untreated primary (de novo) GBM

TCGA-06-0238 405 1 405 1 NA NA 311 1 46 MALE Untreated primary (de novo) GBM

TCGA-08-0346 256 1 256 1 NA NA 75 1 69 MALE Untreated primary (de novo) GBM

TCGA-28-1750 254 0 254 0 NA NA 116 1 40 FEMALE Untreated primary (de novo) GBM

TCGA-06-0175 123 1 123 1 NA NA 39 1 69 MALE Untreated primary (de novo) GBM

TCGA-26-1443 217 0 217 0 NA NA 217 1 60 FEMALE Untreated primary (de novo) GBM

TCGA-06-0410 143 1 143 1 NA NA 143 1 76 FEMALE Untreated primary (de novo) GBM

TCGA-02-0025 1300 1 1300 1 NA NA 656 1 47 MALE Untreated primary (de novo) GBM

TCGA-26-1440 296 0 296 0 NA NA 233 1 48 MALE Untreated primary (de novo) GBM

TCGA-32-1970 468 1 468 1 NA NA 408 1 59 MALE Untreated primary (de novo) GBM

TCGA-08-0375 372 1 372 1 NA NA 161 1 51 FEMALE Untreated primary (de novo) GBM

TCGA-16-0848 535 1 535 1 NA NA 129 1 57 MALE Untreated primary (de novo) GBM

TCGA-19-1389 141 1 141 1 NA NA 81 1 51 MALE Untreated primary (de novo) GBM

TCGA-06-2566 182 1 182 1 NA NA 182 1 23 FEMALE Untreated primary (de novo) GBM

TCGA-12-0620 318 1 318 1 NA NA 318 1 57 MALE Untreated primary (de novo) GBM

TCGA-06-0394 329 1 329 1 NA NA 87 1 51 MALE Untreated primary (de novo) GBM

TCGA-08-0246 127 1 127 1 NA NA 103 1 57 FEMALE Untreated primary (de novo) GBM

TCGA-19-1392 111 1 111 NA NA NA 111 0 72 FEMALE Untreated primary (de novo) GBM

TCGA-06-0149 262 1 262 1 NA NA 204 1 74 FEMALE Untreated primary (de novo) GBM

TCGA-08-0354 546 1 546 1 NA NA 253 1 52 FEMALE Untreated primary (de novo) GBM

TCGA-02-0339 377 1 377 1 NA NA 148 1 67 MALE Untreated primary (de novo) GBM

TCGA-06-2563 932 0 932 0 NA NA 554 1 72 FEMALE Untreated primary (de novo) GBM

TCGA-02-0079 829 1 829 1 NA NA 798 1 57 MALE Untreated primary (de novo) GBM

TCGA-32-2634 693 0 693 0 NA NA 693 0 82 MALE Untreated primary (de novo) GBM

TCGA-16-1062 646 1 646 1 NA NA 518 1 57 FEMALE Untreated primary (de novo) GBM

TCGA-16-0846 119 1 119 1 NA NA 119 1 85 MALE Untreated primary (de novo) GBM

TCGA-12-1098 121 1 121 1 NA NA 93 1 75 FEMALE Untreated primary (de novo) GBM

TCGA-12-0829 626 1 626 1 NA NA 473 1 75 MALE Untreated primary (de novo) GBM

TCGA-06-2562 382 1 382 1 NA NA 151 1 81 MALE Untreated primary (de novo) GBM

TCGA-12-3644 1818 1 1818 1 NA NA 1142 1 47 FEMALE Untreated primary (de novo) GBM

TCGA-16-1047 139 1 139 1 NA NA 25 1 66 FEMALE Untreated primary (de novo) GBM

TCGA-19-1390 772 1 772 NA NA NA 772 0 63 FEMALE Untreated primary (de novo) GBM

TCGA-06-1805 1031 0 1031 0 NA NA 1031 0 28 FEMALE Untreated primary (de novo) GBM

TCGA-08-0386 548 1 548 1 NA NA 427 1 74 MALE Untreated primary (de novo) GBM

TCGA-41-2575 290 1 290 1 NA NA 131 1 75 MALE Untreated primary (de novo) GBM

TCGA-12-0778 454 1 454 1 NA NA 232 1 53 MALE Untreated primary (de novo) GBM

TCGA-12-3652 1062 1 1062 1 NA NA 203 1 60 MALE Untreated primary (de novo) GBM

TCGA-06-0882 632 1 632 1 NA NA 213 1 30 MALE Untreated primary (de novo) GBM

TCGA-16-0850 498 1 498 1 NA NA 83 1 52 FEMALE Untreated primary (de novo) GBM

TCGA-08-0385 82 1 82 1 NA NA 82 1 71 MALE Untreated primary (de novo) GBM

TCGA-26-5136 577 1 577 1 NA NA 577 1 78 FEMALE Untreated primary (de novo) GBM

TCGA-28-5207 343 1 343 1 NA NA 343 1 71 MALE Untreated primary (de novo) GBM

TCGA-06-5418 83 1 83 1 NA NA 83 1 75 FEMALE Untreated primary (de novo) GBM

TCGA-76-4932 1458 1 1458 1 NA NA 1458 1 50 FEMALE Treated primary GBM

TCGA-12-5295 454 1 454 1 NA NA 399 1 60 FEMALE Untreated primary (de novo) GBM

TCGA-76-4934 77 1 77 0 NA NA 77 0 66 FEMALE Untreated primary (de novo) GBM

TCGA-28-5216 415 0 415 0 NA NA 415 0 52 MALE Untreated primary (de novo) GBM

TCGA-76-4929 111 1 111 1 NA NA 111 1 76 FEMALE Untreated primary (de novo) GBM

TCGA-12-5299 98 1 98 1 NA NA 98 1 56 FEMALE Untreated primary (de novo) GBM

TCGA-26-5133 452 0 452 0 NA NA 370 1 59 MALE Untreated primary (de novo) GBM

TCGA-26-5139 48 0 48 0 NA NA 48 0 65 FEMALE Untreated primary (de novo) GBM

TCGA-76-4935 1121 1 1121 0 NA NA 1121 0 52 FEMALE Untreated primary (de novo) GBM

TCGA-06-5414 273 0 273 0 NA NA 167 1 61 MALE Untreated primary (de novo) GBM

TCGA-06-5416 204 0 204 0 NA NA 204 0 23 FEMALE Untreated primary (de novo) GBM

TCGA-28-5204 454 1 454 1 NA NA 454 1 72 MALE Untreated primary (de novo) GBM

TCGA-28-5220 388 1 388 1 NA NA 262 1 67 MALE Untreated primary (de novo) GBM

TCGA-76-4926 138 1 138 1 NA NA 34 1 68 MALE Untreated primary (de novo) GBM

TCGA-26-5132 286 0 286 0 NA NA 286 0 74 MALE Untreated primary (de novo) GBM

TCGA-76-4925 146 1 146 NA NA NA 88 1 76 MALE Untreated primary (de novo) GBM

TCGA-28-5208 544 1 544 1 NA NA 148 1 52 MALE Untreated primary (de novo) GBM

TCGA-32-5222 585 1 585 1 NA NA 118 1 66 MALE Untreated primary (de novo) GBM

TCGA-06-5415 260 0 260 0 NA NA 260 0 60 MALE Untreated primary (de novo) GBM

TCGA-28-5215 335 1 335 1 NA NA 164 1 62 FEMALE Untreated primary (de novo) GBM

TCGA-28-5213 951 0 951 0 NA NA 951 0 72 MALE Untreated primary (de novo) GBM

TCGA-28-5209 442 0 442 0 NA NA 442 0 66 FEMALE Untreated primary (de novo) GBM

TCGA-76-4931 279 1 279 1 NA NA 112 1 70 FEMALE Untreated primary (de novo) GBM

TCGA-28-5214 713 1 713 1 NA NA 247 1 53 MALE Untreated primary (de novo) GBM

TCGA-26-5135 270 1 270 NA NA NA 270 0 72 FEMALE Untreated primary (de novo) GBM

TCGA-28-5219 690 0 690 0 NA NA 258 1 47 FEMALE Untreated primary (de novo) GBM

TCGA-76-4927 535 1 535 1 NA NA 416 1 58 MALE Untreated primary (de novo) GBM

TCGA-06-5417 155 0 155 0 NA NA 155 0 45 FEMALE Untreated primary (de novo) GBM

TCGA-28-5218 157 1 157 NA NA NA 157 0 63 MALE Untreated primary (de novo) GBM

TCGA-76-4928 94 1 94 1 NA NA 94 1 85 FEMALE Untreated primary (de novo) GBM

TCGA-12-5301 62 1 62 1 NA NA 62 1 59 MALE Untreated primary (de novo) GBM

TCGA-26-5134 167 0 167 0 NA NA 167 0 74 MALE Untreated primary (de novo) GBM

TCGA-32-4208 643 0 643 0 NA NA 643 0 25 MALE Untreated primary (de novo) GBM

TCGA-32-1978 482 1 482 1 NA NA 482 1 57 MALE Untreated primary (de novo) GBM

TCGA-32-2494 632 1 632 1 NA NA 488 1 58 FEMALE Untreated primary (de novo) GBM

TCGA-41-2572 406 1 406 1 NA NA 122 1 67 MALE Untreated primary (de novo) GBM

TCGA-26-1439 422 1 422 1 NA NA 284 1 63 MALE Untreated primary (de novo) GBM

TCGA-32-1991 515 1 515 1 NA NA 345 1 60 MALE Untreated primary (de novo) GBM

TCGA-32-4213 604 0 604 0 NA NA 353 1 47 FEMALE Untreated primary (de novo) GBM

TCGA-32-4210 113 1 113 1 NA NA 113 1 73 MALE Untreated primary (de novo) GBM

TCGA-32-2495 457 1 457 1 NA NA 252 1 59 FEMALE Treated primary GBM

TCGA-19-1790 154 1 154 1 NA NA 88 1 56 MALE Untreated primary (de novo) GBM

TCGA-41-3393 135 1 135 1 NA NA 135 1 81 FEMALE Untreated primary (de novo) GBM

TCGA-32-4719 330 1 330 1 NA NA 330 1 73 MALE Untreated primary (de novo) GBM

TCGA-32-1973 641 1 641 1 NA NA 507 1 21 MALE Untreated primary (de novo) GBM

TCGA-19-4068 137 0 137 0 NA NA 137 0 68 FEMALE Untreated primary (de novo) GBM

TCGA-32-2491 372 1 372 1 NA NA 70 1 63 MALE Untreated primary (de novo) GBM

TCGA-19-2631 213 1 213 0 NA NA 213 0 74 FEMALE Untreated primary (de novo) GBM

TCGA-32-1987 452 1 452 1 NA NA 68 1 49 FEMALE Untreated primary (de novo) GBM

TCGA-32-4209 618 1 618 1 NA NA 94 1 40 MALE Treated primary GBM

TCGA-32-4211 383 1 383 1 NA NA 383 1 56 MALE Untreated primary (de novo) GBM

TCGA-06-0939 814 1 814 1 NA NA 329 1 79 FEMALE Untreated primary (de novo) GBM

TCGA-41-3915 360 1 360 1 NA NA 288 1 48 MALE Untreated primary (de novo) GBM

TCGA-14-4157 104 0 104 0 NA NA 96 1 40 MALE Untreated primary (de novo) GBM

TCGA-06-5410 108 1 108 1 NA NA 108 1 72 FEMALE Untreated primary (de novo) GBM

TCGA-81-5911 539 0 539 0 NA NA 277 1 33 MALE Untreated primary (de novo) GBM

TCGA-19-5953 144 1 144 1 NA NA 119 1 58 MALE Untreated primary (de novo) GBM

TCGA-76-6286 638 1 638 1 NA NA 180 1 60 MALE Untreated primary (de novo) GBM

TCGA-06-6389 237 0 237 0 NA NA 237 0 49 FEMALE Untreated primary (de novo) GBM

TCGA-41-6646 379 1 379 1 NA NA 200 1 73 FEMALE Untreated primary (de novo) GBM

TCGA-87-5896 800 0 800 0 800 0 800 0 50 FEMALE Untreated primary (de novo) GBM

TCGA-06-5408 357 1 357 1 NA NA 158 1 54 FEMALE Untreated primary (de novo) GBM

TCGA-76-6664 1101 0 1101 0 NA NA 1101 0 49 FEMALE Untreated primary (de novo) GBM

TCGA-15-1444 1537 1 1537 1 1550 1 1550 1 21 MALE Glioblastoma Multiforme (GBM)

TCGA-76-6656 147 1 147 NA NA NA 147 0 66 MALE Untreated primary (de novo) GBM

TCGA-19-5955 54 1 54 NA NA NA 54 0 83 MALE Untreated primary (de novo) GBM

TCGA-06-5859 139 0 139 0 NA NA 139 0 63 MALE Untreated primary (de novo) GBM

TCGA-76-6282 519 1 519 1 NA NA 458 1 63 MALE Untreated primary (de novo) GBM

TCGA-06-6695 253 0 253 0 NA NA 133 1 64 MALE Untreated primary (de novo) GBM

TCGA-06-1804 414 1 414 1 NA NA 414 1 81 FEMALE Untreated primary (de novo) GBM

TCGA-28-6450 165 1 165 1 NA NA 165 1 60 MALE Untreated primary (de novo) GBM

TCGA-06-6694 224 1 224 1 NA NA 224 1 76 FEMALE Untreated primary (de novo) GBM

TCGA-41-5651 460 1 460 1 NA NA 210 1 59 FEMALE Untreated primary (de novo) GBM

TCGA-06-6698 145 0 145 0 NA NA 145 0 53 FEMALE Untreated primary (de novo) GBM

TCGA-74-6584 228 0 228 0 NA NA 91 1 55 FEMALE Untreated primary (de novo) GBM

TCGA-74-6573 105 1 105 1 NA NA 105 1 67 MALE Untreated primary (de novo) GBM

TCGA-06-6700 145 0 145 0 NA NA 145 0 76 MALE Untreated primary (de novo) GBM

TCGA-32-1980 36 1 36 1 NA NA 36 1 72 MALE Untreated primary (de novo) GBM

TCGA-76-6660 114 1 114 1 NA NA 114 1 73 MALE Glioblastoma Multiforme (GBM)

TCGA-76-6191 508 1 508 1 NA NA 323 1 57 MALE Untreated primary (de novo) GBM

TCGA-06-5856 114 1 114 1 NA NA 114 1 58 MALE Untreated primary (de novo) GBM

TCGA-26-1442 953 0 953 0 NA NA 953 0 43 MALE Untreated primary (de novo) GBM

TCGA-26-6173 241 0 241 0 NA NA 241 0 57 MALE Untreated primary (de novo) GBM

TCGA-19-5958 428 1 428 1 NA NA 356 1 56 MALE Untreated primary (de novo) GBM

TCGA-19-5951 244 1 244 1 NA NA 244 1 76 FEMALE Untreated primary (de novo) GBM

TCGA-76-6285 254 1 254 1 NA NA 254 1 64 FEMALE Untreated primary (de novo) GBM

TCGA-32-1979 593 1 593 1 NA NA 265 1 69 FEMALE Untreated primary (de novo) GBM

TCGA-14-0740 364 1 364 1 NA NA 364 1 70 MALE Untreated primary (de novo) GBM

TCGA-19-5947 202 1 202 1 NA NA 43 1 47 FEMALE Untreated primary (de novo) GBM

TCGA-14-1395 42 1 42 1 NA NA 42 1 52 MALE Untreated primary (de novo) GBM

TCGA-19-5954 368 1 368 1 NA NA 368 1 72 FEMALE Untreated primary (de novo) GBM

TCGA-28-5211 358 0 358 0 NA NA 225 1 42 MALE Untreated primary (de novo) GBM

TCGA-76-6662 1048 1 1048 1 NA NA 205 1 58 MALE Untreated primary (de novo) GBM

TCGA-19-5952 575 1 575 NA NA NA 575 0 62 MALE Untreated primary (de novo) GBM

TCGA-76-6280 346 1 346 1 NA NA 108 1 57 MALE Untreated primary (de novo) GBM

TCGA-06-5411 254 1 254 1 NA NA 214 1 51 MALE Untreated primary (de novo) GBM

TCGA-76-6661 727 1 727 1 NA NA 727 1 54 MALE Untreated primary (de novo) GBM

TCGA-06-0650 717 1 717 1 NA NA 351 1 39 FEMALE Untreated primary (de novo) GBM

TCGA-81-5910 49 1 49 1 NA NA 49 1 64 MALE Untreated primary (de novo) GBM

TCGA-74-6575 636 0 636 0 NA NA 93 1 73 FEMALE Untreated primary (de novo) GBM

TCGA-26-6174 71 0 71 0 NA NA 71 0 65 FEMALE Untreated primary (de novo) GBM

TCGA-06-6391 45 1 45 1 NA NA 45 1 44 FEMALE Untreated primary (de novo) GBM

TCGA-06-6697 391 0 391 0 NA NA 306 1 65 MALE Untreated primary (de novo) GBM

TCGA-06-6701 151 0 151 0 NA NA 151 0 60 MALE Untreated primary (de novo) GBM

TCGA-06-6693 3667 1 3667 1 NA NA 3667 1 64 FEMALE Untreated primary (de novo) GBM

TCGA-06-5412 138 1 138 1 NA NA 88 1 78 FEMALE Untreated primary (de novo) GBM

TCGA-19-5959 511 1 511 1 NA NA 119 1 77 FEMALE Untreated primary (de novo) GBM

TCGA-74-6581 250 0 250 0 NA NA 250 0 77 MALE Untreated primary (de novo) GBM

TCGA-74-6577 132 0 132 0 NA NA 132 1 51 MALE Untreated primary (de novo) GBM

TCGA-14-0862 88 1 88 1 NA NA 88 1 60 MALE Untreated primary (de novo) GBM

TCGA-76-6193 82 1 82 NA NA NA 64 1 78 MALE Untreated primary (de novo) GBM

TCGA-06-6388 159 1 159 1 NA NA 85 1 64 FEMALE Untreated primary (de novo) GBM

TCGA-06-6390 164 1 164 1 NA NA 164 1 58 MALE Untreated primary (de novo) GBM

TCGA-76-6283 165 1 165 1 NA NA 165 1 71 FEMALE Glioblastoma Multiforme (GBM)

TCGA-76-6657 153 1 153 1 NA NA 135 1 74 MALE Untreated primary (de novo) GBM

TCGA-06-5413 268 0 268 0 NA NA 195 1 67 MALE Untreated primary (de novo) GBM

TCGA-14-1450 1788 0 1788 0 NA NA 826 1 57 FEMALE Untreated primary (de novo) GBM

TCGA-74-6578 436 0 436 0 NA NA 222 1 58 MALE Untreated primary (de novo) GBM

TCGA-19-5956 684 1 684 1 NA NA 647 1 53 FEMALE Untreated primary (de novo) GBM

TCGA-06-6699 47 0 47 0 NA NA 47 0 58 FEMALE Untreated primary (de novo) GBM
