# Supplementary material for: Identification of glioblastoma immune subtypes and immune landscape based on a large cohort
Source: Hereditas. 2021 Aug 19;158:30. doi: 10.1186/s41065-021-00193-x (PMC8377979; doi:10.1186/s41065-021-00193-x)
Supplement: Supplementary file 2 — Additional file 2. [file 41065_2021_193_MOESM2_ESM.docx]

Samples OS.time OS DSS.time DSS DFI.time DFI PFI.time PFI Age Gender histological_type Stage Grade

TCGA-06-0125 1448 1 1448 1 NA NA 797 1 63 FEMALE Untreated primary (de novo) GBM

TCGA-06-0152 375 1 375 1 NA NA 299 1 68 MALE Untreated primary (de novo) GBM

TCGA-06-0171 399 1 399 1 NA NA 117 1 65 MALE Untreated primary (de novo) GBM

TCGA-06-0190 317 1 317 1 NA NA 88 1 62 MALE Untreated primary (de novo) GBM

TCGA-02-0047 448 1 448 1 NA NA 57 1 78 MALE Untreated primary (de novo) GBM

TCGA-02-0055 76 1 76 1 NA NA 6 1 62 FEMALE Untreated primary (de novo) GBM

TCGA-02-2483 466 0 466 0 NA NA 466 0 43 MALE Untreated primary (de novo) GBM

TCGA-02-2485 470 0 470 0 NA NA 186 1 53 MALE Untreated primary (de novo) GBM

TCGA-02-2486 618 1 618 1 NA NA 618 1 64 MALE Untreated primary (de novo) GBM

TCGA-06-0129 1024 1 1024 1 NA NA 148 1 30 MALE Untreated primary (de novo) GBM

TCGA-06-0130 394 1 394 1 NA NA 244 1 54 MALE Untreated primary (de novo) GBM

TCGA-06-0132 771 1 771 1 NA NA 482 1 49 MALE Untreated primary (de novo) GBM

TCGA-06-0138 737 1 737 1 NA NA 394 1 43 MALE Untreated primary (de novo) GBM

TCGA-06-0139 362 1 362 1 NA NA 152 1 40 MALE Untreated primary (de novo) GBM

TCGA-06-0141 313 1 313 1 NA NA 145 1 62 MALE Untreated primary (de novo) GBM

TCGA-06-0156 178 1 178 1 NA NA 178 1 57 MALE Untreated primary (de novo) GBM

TCGA-06-0157 97 1 97 1 NA NA 97 1 63 FEMALE Untreated primary (de novo) GBM

TCGA-06-0158 329 1 329 1 NA NA 90 1 73 MALE Untreated primary (de novo) GBM

TCGA-06-0168 598 1 598 1 NA NA 461 1 59 FEMALE Untreated primary (de novo) GBM

TCGA-06-0174 98 1 98 1 NA NA 47 1 54 MALE Untreated primary (de novo) GBM

TCGA-06-0178 2681 1 2681 1 NA NA 192 1 38 MALE Untreated primary (de novo) GBM

TCGA-06-0184 2126 1 2126 1 NA NA 1276 1 63 MALE Untreated primary (de novo) GBM

TCGA-06-0187 828 1 828 1 NA NA 531 1 69 MALE Untreated primary (de novo) GBM

TCGA-06-0210 225 1 225 1 NA NA 67 1 72 FEMALE Untreated primary (de novo) GBM

TCGA-06-0211 360 1 360 1 NA NA 53 1 47 MALE Untreated primary (de novo) GBM

TCGA-06-0238 405 1 405 1 NA NA 311 1 46 MALE Untreated primary (de novo) GBM

TCGA-06-0644 384 1 384 1 NA NA 85 1 71 MALE Untreated primary (de novo) GBM

TCGA-06-0645 175 1 175 1 NA NA 175 1 55 FEMALE Untreated primary (de novo) GBM

TCGA-06-0646 175 1 175 1 NA NA 90 1 60 MALE Untreated primary (de novo) GBM

TCGA-06-0649 64 1 64 1 NA NA 64 1 73 FEMALE Untreated primary (de novo) GBM

TCGA-06-0686 432 1 432 1 NA NA 160 1 53 MALE Untreated primary (de novo) GBM

TCGA-06-0743 803 1 803 1 NA NA 176 1 69 MALE Untreated primary (de novo) GBM

TCGA-06-0744 1426 1 1426 1 NA NA 1277 1 66 MALE Untreated primary (de novo) GBM

TCGA-06-0745 239 1 239 1 NA NA 92 1 59 MALE Untreated primary (de novo) GBM

TCGA-06-0747 82 1 82 1 NA NA 82 1 53 MALE Untreated primary (de novo) GBM

TCGA-06-0749 82 1 82 1 NA NA 82 1 50 MALE Untreated primary (de novo) GBM

TCGA-06-0878 218 0 218 0 NA NA 66 1 74 MALE Untreated primary (de novo) GBM

TCGA-06-0882 632 1 632 1 NA NA 213 1 30 MALE Untreated primary (de novo) GBM

TCGA-06-1804 414 1 414 1 NA NA 414 1 81 FEMALE Untreated primary (de novo) GBM

TCGA-06-2557 33 1 33 1 NA NA 33 1 76 MALE Untreated primary (de novo) GBM

TCGA-06-2558 380 1 380 NA NA NA 380 0 75 FEMALE Untreated primary (de novo) GBM

TCGA-06-2559 150 1 150 1 NA NA 150 1 83 MALE Untreated primary (de novo) GBM

TCGA-06-2561 537 1 537 1 NA NA 78 1 53 FEMALE Untreated primary (de novo) GBM

TCGA-06-2562 382 1 382 1 NA NA 151 1 81 MALE Untreated primary (de novo) GBM

TCGA-06-2563 932 0 932 0 NA NA 554 1 72 FEMALE Untreated primary (de novo) GBM

TCGA-06-2564 181 0 181 0 NA NA 181 0 50 MALE Untreated primary (de novo) GBM

TCGA-06-2565 506 1 506 1 NA NA 178 1 59 MALE Untreated primary (de novo) GBM

TCGA-06-2567 133 1 133 1 NA NA 133 1 65 MALE Untreated primary (de novo) GBM

TCGA-06-2570 958 0 958 0 NA NA 958 0 21 FEMALE Untreated primary (de novo) GBM

TCGA-06-5408 357 1 357 1 NA NA 158 1 54 FEMALE Untreated primary (de novo) GBM

TCGA-06-5410 108 1 108 1 NA NA 108 1 72 FEMALE Untreated primary (de novo) GBM

TCGA-06-5411 254 1 254 1 NA NA 214 1 51 MALE Untreated primary (de novo) GBM

TCGA-06-5412 138 1 138 1 NA NA 88 1 78 FEMALE Untreated primary (de novo) GBM

TCGA-06-5413 268 0 268 0 NA NA 195 1 67 MALE Untreated primary (de novo) GBM

TCGA-06-5414 273 0 273 0 NA NA 167 1 61 MALE Untreated primary (de novo) GBM

TCGA-06-5416 204 0 204 0 NA NA 204 0 23 FEMALE Untreated primary (de novo) GBM

TCGA-06-5417 155 0 155 0 NA NA 155 0 45 FEMALE Untreated primary (de novo) GBM

TCGA-06-5418 83 1 83 1 NA NA 83 1 75 FEMALE Untreated primary (de novo) GBM

TCGA-06-5856 114 1 114 1 NA NA 114 1 58 MALE Untreated primary (de novo) GBM

TCGA-06-5858 187 0 187 0 NA NA 97 1 45 FEMALE Untreated primary (de novo) GBM

TCGA-06-5859 139 0 139 0 NA NA 139 0 63 MALE Untreated primary (de novo) GBM

TCGA-08-0386 548 1 548 1 NA NA 427 1 74 MALE Untreated primary (de novo) GBM

TCGA-12-0616 448 1 448 1 NA NA 398 1 36 FEMALE Untreated primary (de novo) GBM

TCGA-12-0618 395 1 395 NA NA NA 395 0 49 MALE Untreated primary (de novo) GBM

TCGA-12-0619 1062 1 1062 1 NA NA 203 1 60 MALE Untreated primary (de novo) GBM

TCGA-12-0821 323 1 323 1 NA NA 259 1 62 MALE Untreated primary (de novo) GBM

TCGA-12-1597 675 1 675 1 NA NA 180 1 62 FEMALE Untreated primary (de novo) GBM

TCGA-12-3650 333 1 333 1 NA NA 239 1 46 MALE Untreated primary (de novo) GBM

TCGA-12-3652 1062 1 1062 1 NA NA 203 1 60 MALE Untreated primary (de novo) GBM

TCGA-12-3653 442 1 442 1 NA NA 224 1 34 FEMALE Untreated primary (de novo) GBM

TCGA-12-5295 454 1 454 1 NA NA 399 1 60 FEMALE Untreated primary (de novo) GBM

TCGA-12-5299 98 1 98 1 NA NA 98 1 56 FEMALE Untreated primary (de novo) GBM

TCGA-14-0787 68 1 68 NA NA NA 68 0 69 MALE Untreated primary (de novo) GBM

TCGA-14-0789 342 1 342 NA NA NA 105 1 54 MALE Untreated primary (de novo) GBM

TCGA-14-0790 419 1 419 1 NA NA 419 1 64 FEMALE Untreated primary (de novo) GBM

TCGA-14-0817 164 1 164 NA NA NA 164 0 69 FEMALE Untreated primary (de novo) GBM

TCGA-14-0871 880 1 880 NA NA NA 880 0 74 FEMALE Untreated primary (de novo) GBM

TCGA-14-1034 485 1 485 1 NA NA 282 1 60 FEMALE Untreated primary (de novo) GBM

TCGA-14-1823 543 1 543 1 NA NA 140 1 58 FEMALE Untreated primary (de novo) GBM

TCGA-14-1825 232 1 232 1 NA NA 82 1 70 MALE Untreated primary (de novo) GBM

TCGA-14-1829 218 0 218 0 NA NA 218 0 57 MALE Untreated primary (de novo) GBM

TCGA-14-2554 532 1 532 1 NA NA 532 1 52 FEMALE Untreated primary (de novo) GBM

TCGA-15-0742 419 1 419 NA NA NA 232 1 65 MALE Untreated primary (de novo) GBM

TCGA-15-1444 1537 1 1537 1 1550 1 1550 1 21 MALE Glioblastoma Multiforme (GBM)

TCGA-16-0846 119 1 119 1 NA NA 119 1 85 MALE Untreated primary (de novo) GBM

TCGA-16-1045 883 1 883 1 NA NA 654 1 49 FEMALE Untreated primary (de novo) GBM

TCGA-19-1390 772 1 772 NA NA NA 772 0 63 FEMALE Untreated primary (de novo) GBM

TCGA-19-1787 385 1 385 1 NA NA 308 1 48 MALE Untreated primary (de novo) GBM

TCGA-19-2619 294 0 294 0 NA NA 146 1 55 FEMALE Untreated primary (de novo) GBM

TCGA-19-2620 148 1 148 0 NA NA 148 0 70 MALE Untreated primary (de novo) GBM

TCGA-19-2625 124 1 124 0 NA NA 124 0 76 FEMALE Untreated primary (de novo) GBM

TCGA-19-2629 737 1 737 1 NA NA 145 1 60 MALE Untreated primary (de novo) GBM

TCGA-19-4065 214 0 214 0 NA NA 70 1 36 MALE Untreated primary (de novo) GBM

TCGA-19-5960 455 1 455 1 NA NA 382 1 56 MALE Untreated primary (de novo) GBM

TCGA-26-1442 953 0 953 0 NA NA 953 0 43 MALE Untreated primary (de novo) GBM

TCGA-26-5132 286 0 286 0 NA NA 286 0 74 MALE Untreated primary (de novo) GBM

TCGA-26-5133 452 0 452 0 NA NA 370 1 59 MALE Untreated primary (de novo) GBM

TCGA-26-5134 167 0 167 0 NA NA 167 0 74 MALE Untreated primary (de novo) GBM

TCGA-26-5135 270 1 270 NA NA NA 270 0 72 FEMALE Untreated primary (de novo) GBM

TCGA-26-5136 577 1 577 1 NA NA 577 1 78 FEMALE Untreated primary (de novo) GBM

TCGA-26-5139 48 0 48 0 NA NA 48 0 65 FEMALE Untreated primary (de novo) GBM

TCGA-27-1830 154 1 154 1 NA NA 124 1 57 MALE Untreated primary (de novo) GBM

TCGA-27-1831 505 1 505 1 NA NA 144 1 66 MALE Untreated primary (de novo) GBM

TCGA-27-1832 300 1 300 1 NA NA 176 1 59 FEMALE Untreated primary (de novo) GBM

TCGA-27-1834 1233 1 1233 1 NA NA 335 1 56 MALE Untreated primary (de novo) GBM

TCGA-27-1835 648 1 648 1 NA NA 157 1 53 FEMALE Untreated primary (de novo) GBM

TCGA-27-1837 427 1 427 1 NA NA 136 1 36 MALE Untreated primary (de novo) GBM

TCGA-27-2519 550 1 550 1 NA NA 256 1 48 MALE Untreated primary (de novo) GBM

TCGA-27-2521 510 1 510 1 NA NA 510 1 34 MALE Untreated primary (de novo) GBM

TCGA-27-2523 489 1 489 1 NA NA 402 1 63 MALE Untreated primary (de novo) GBM

TCGA-27-2524 231 1 231 1 NA NA 231 1 56 MALE Untreated primary (de novo) GBM

TCGA-27-2526 87 1 87 1 NA NA 87 1 79 FEMALE Untreated primary (de novo) GBM

TCGA-27-2528 480 1 480 1 NA NA 72 1 62 MALE Untreated primary (de novo) GBM

TCGA-28-1747 77 1 77 NA NA NA 77 0 44 MALE Untreated primary (de novo) GBM

TCGA-28-1753 37 0 37 0 NA NA 37 0 53 MALE Untreated primary (de novo) GBM

TCGA-28-2499 95 0 95 NA NA NA 95 0 59 MALE Untreated primary (de novo) GBM

TCGA-28-2509 145 0 145 0 NA NA 145 0 77 FEMALE Untreated primary (de novo) GBM

TCGA-28-2513 222 0 222 0 NA NA 75 1 69 FEMALE Untreated primary (de novo) GBM

TCGA-28-2514 160 0 160 0 NA NA 160 0 45 MALE Untreated primary (de novo) GBM

TCGA-28-5204 454 1 454 1 NA NA 454 1 72 MALE Untreated primary (de novo) GBM

TCGA-28-5207 343 1 343 1 NA NA 343 1 71 MALE Untreated primary (de novo) GBM

TCGA-28-5208 544 1 544 1 NA NA 148 1 52 MALE Untreated primary (de novo) GBM

TCGA-28-5209 442 0 442 0 NA NA 442 0 66 FEMALE Untreated primary (de novo) GBM

TCGA-28-5213 951 0 951 0 NA NA 951 0 72 MALE Untreated primary (de novo) GBM

TCGA-28-5215 335 1 335 1 NA NA 164 1 62 FEMALE Untreated primary (de novo) GBM

TCGA-28-5216 415 0 415 0 NA NA 415 0 52 MALE Untreated primary (de novo) GBM

TCGA-28-5218 157 1 157 NA NA NA 157 0 63 MALE Untreated primary (de novo) GBM

TCGA-28-5220 388 1 388 1 NA NA 262 1 67 MALE Untreated primary (de novo) GBM

TCGA-32-1970 468 1 468 1 NA NA 408 1 59 MALE Untreated primary (de novo) GBM

TCGA-32-1980 36 1 36 1 NA NA 36 1 72 MALE Untreated primary (de novo) GBM

TCGA-32-1982 142 1 142 1 NA NA 142 1 76 FEMALE Untreated primary (de novo) GBM

TCGA-32-2615 485 1 485 1 NA NA 131 1 62 MALE Untreated primary (de novo) GBM

TCGA-32-2616 224 1 224 1 NA NA 182 1 48 FEMALE Untreated primary (de novo) GBM

TCGA-32-2632 269 1 269 1 NA NA 269 1 80 MALE Untreated primary (de novo) GBM

TCGA-32-2634 693 0 693 0 NA NA 693 0 82 MALE Untreated primary (de novo) GBM

TCGA-32-2638 766 1 766 1 NA NA 766 1 67 MALE Untreated primary (de novo) GBM

TCGA-32-4213 604 0 604 0 NA NA 353 1 47 FEMALE Untreated primary (de novo) GBM

TCGA-32-5222 585 1 585 1 NA NA 118 1 66 MALE Untreated primary (de novo) GBM

TCGA-41-2572 406 1 406 1 NA NA 122 1 67 MALE Untreated primary (de novo) GBM

TCGA-41-3915 360 1 360 1 NA NA 288 1 48 MALE Untreated primary (de novo) GBM

TCGA-41-5651 460 1 460 1 NA NA 210 1 59 FEMALE Untreated primary (de novo) GBM

TCGA-76-4925 146 1 146 NA NA NA 88 1 76 MALE Untreated primary (de novo) GBM

TCGA-76-4926 138 1 138 1 NA NA 34 1 68 MALE Untreated primary (de novo) GBM

TCGA-76-4927 535 1 535 1 NA NA 416 1 58 MALE Untreated primary (de novo) GBM

TCGA-76-4928 94 1 94 1 NA NA 94 1 85 FEMALE Untreated primary (de novo) GBM

TCGA-76-4929 111 1 111 1 NA NA 111 1 76 FEMALE Untreated primary (de novo) GBM

TCGA-76-4931 279 1 279 1 NA NA 112 1 70 FEMALE Untreated primary (de novo) GBM

TCGA-76-4932 1458 1 1458 1 NA NA 1458 1 50 FEMALE Treated primary GBM
