# Supplementary material for: Identification of glioblastoma immune subtypes and immune landscape based on a large cohort
Source: Hereditas. 2021 Aug 19;158:30. doi: 10.1186/s41065-021-00193-x (PMC8377979; doi:10.1186/s41065-021-00193-x)
Supplement: Supplementary file 3 — Additional file 3. [file 41065_2021_193_MOESM3_ESM.docx]

GeneSymbol GeneID

A2M 2

ABCA6 23460

ABCA8 10351

ABCA9 10350

ABCB1 5243

ABCC9 10060

ABCD2 225

ABI3 51225

ABI3BP 25890

ACAP1 9744

ACE 1636

ACHE 43

ACOXL 55289

ACP5 54

ACSL5 51703

ACSM5 54988

ACSS3 79611

ACTA2 59

ACTN1 87

ACVR1 90

ACVR1B 91

ACVR2A 92

ACVR2B 93

ACVRL1 94

ADAM12 8038

ADAM28 10863

ADAM6 8755

ADAM8 101

ADAMDEC1 27299

ADAMTS10 81794

ADAMTS12 81792

ADAMTS14 140766

ADAMTS16 170690

ADAMTS2 9509

ADAMTS4 9507

ADAMTS5 11096

ADAMTS9 56999

ADAMTSL2 9719

ADAP2 55803

ADAT1 23536

ADCY4 196883

ADCYAP1 116

ADORA2A 135

ADORA3 140

ADPRH 141

ADRA2A 150

ADRM1 11047

AEBP1 165

AFF3 3899

AGAP2 116986

AGTR1 185

AHCYL2 23382

AIF1 199

CRYBG2 55057

AIM2 9447

AKAP12 9590

AKAP2 11217

AKAP5 9495

AKNA 80709

ALDH1A1 216

ALDH3B1 221

ALOX5 240

ALOX5AP 241

ALOXE3 59344

ALPK2 115701

AMH 268

AMHR2 269

JAML 120425

AMIGO3 386724

AMPD1 270

AMPH 273

ANGPTL1 9068

ANGPTL2 23452

ANK2 287

ANKRD17 26057

ANKRD22 118932

ANKRD36BP1 84832

ANKRD44 91526

ANKRD55 79722

SOWAHD 347454

ANO6 196527

ANTXR1 84168

ANTXR2 118429

ANXA6 309

AOAH 313

AOC3 8639

AP1S2 8905

AP3B1 8546

APBB1IP 54518

APBB2 323

APLNR 187

APOBR 55911

APOBEC3A 200315

APOBEC3D 140564

APOBEC3G 60489

APOBEC3H 164668

APOC1 341

APOC2 344

APOE 348

APOL3 80833

APOL6 80830

AQP10 89872

AQP1 358

AQP9 366

AREG 374

ARHGAP15 55843

ARHGAP18 93663

ARHGAP22 58504

ARHGAP25 9938

ARHGAP30 257106

ARHGAP31 57514

ARHGAP4 393

ARHGAP6 395

ARHGAP9 64333

ARHGDIB 397

ARHGEF15 22899

ARHGEF37 389337

ARHGEF6 9459

ARID5A 10865

ARL6IP5 10550

ARRB1 408

ARRB2 409

ARRDC5 645432

ARSB 411

ART4 420

ASAH1 427

CLMP 79827

ASGR2 433

ASPN 54829

ASRGL1 80150

ASXL2 55252

ASXL3 80816

ATE1 11101

ATP10A 57194

ATP2A3 489

ATP8A1 10396

ATP8B4 79895

AVPR1A 552

AZGP1 563

B2M 567

B3GAT1 27087

BANK1 55024

BATF 10538

BATF2 116071

BATF3 55509

BCL2A1 597

BCL2L14 79370

BCL6B 255877

BDKRB2 624

BEND5 79656

BEX5 340542

BFSP2 8419

BGN 633

BHLHA15 168620

BHLHE22 27319

BHLHE41 79365

BICC1 80114

BIN2 51411

BIRC6 57448

BLK 640

BMP2 650

BMP2K 55589

BMP7 655

BMPR1A 657

BMPR1B 658

BMPR2 659

BNC2 54796

BST1 683

BST2 684

BTK 695

BTLA 151888

BTN2A2 10385

BTN3A1 11119

BTN3A2 11118

BTN3A3 10384

C10orf128 170371

VSIR 64115

VSTM4 196740

C10orf99 387695

C11orf21 29125

RUBCNL 80183

MEDAG 84935

CEP128 145508

SLIRP 81892

RTRAF 51637

C14orf2 9556

C15orf48 84419

C15orf53 400359

C16orf54 283897

MILR1 284021

SCIMP 388325

LDLRAD4 753

PEAK3 374872

C19orf38 255809

TRIR 79002

MCEMP1 199675

C19orf66 55337

C1orf116 79098

C1orf127 148345

GCSAML 148823

C1orf162 128346

PIK3CD-AS1 644997

C1orf228 339541

THEMIS2 9473

C1orf54 79630

C1QA 712

C1QB 713

C1QC 714

C1QTNF7 114905

LAMP5 24141

C2 717

RTP5 285093

TRABD2A 129293

C3 718

C3AR1 719

C4A 720

NDNF 79625

C5AR1 728

DCANP1 140947

CREBRF 153222

C5orf56 441108

ADTRP 84830

UQCC2 84300

C6orf132 647024

CCDC170 80129

C7 730

CPED1 79974

NUGGC 389643

ERCC6L2 375748

C9orf139 401563

CYSRT1 375791

CACNA1C 775

CACNA2D2 9254

CACNA2D4 93589

CALB2 794

CALD1 800

CALR 811

CAMK1 8536

CAMK4 814

CANX 821

CARD11 84433

CARD8 22900

CARD9 64170

CASP5 838

CASS4 57091

CAV1 857

CCDC102B 79839

CCDC141 285025

CCDC69 26112

CCDC80 151887

CCL11 6356

CCL13 6357

CCL14 6358

CCL15 6359

CCL1 6346

CCL16 6360

CCL17 6361

CCL18 6362

CCL19 6363

CCL20 6364

CCL21 6366

CCL22 6367

CCL23 6368

CCL24 6369

CCL25 6370

CCL26 10344

CCL2 6347

CCL28 56477

CCL3 6348

CCL3L1 6349

CCL3L3 414062

CCL4 6351

CCL4L1 388372

CCL5 6352

CCL7 6354

CCL8 6355

CCNT1 904

CCR10 2826

CCR1 1230

CCR2 729230

CCR3 1232

CCR4 1233

CCR5 1234

CCR6 1235

CCR7 1236

CCR8 1237

CCR9 10803

CCRL2 9034

CD14 929

CD160 11126

CD163 9332

CD163L1 283316

CD180 4064

CD19 930

CD1A 909

CD1B 910

CD1C 911

CD1D 912

CD1E 913

CD200 4345

CD200R1 131450

CD207 50489

CD209 30835

CD226 10666

CD22 933

CD244 51744

CD247 919

CD248 57124

CD274 29126

CD27 939

CD28 940

CD2 914

CD300A 11314

CD300C 10871

CD300E 342510

CD300LB 124599

CD300LF 146722

CD302 9936

CD33 945

CD34 947

CD36 948

CD37 951

CD3D 915

CD3E 916

CD3G 917

CD40 958

CD40LG 959

CD48 962

CD4 920

CD52 1043

CD53 963

CD58 965

CD5 921

CD68 968

CD6 923

CD69 969

CD70 970

CD72 971

CD74 972

CD7 924

CD79A 973

CD79B 974

CD80 941

CD84 8832

CD86 942

CD8A 925

CD8B 926

CD93 22918

CD96 10225

ADGRE5 976

CDC42SE2 56990

CDH11 1009

CDH20 28316

CDH3 1001

CDH5 1003

CDH6 1004

CDK15 65061

CDKL5 6792

CDSN 1041

CEACAM19 56971

CEACAM21 90273

CEACAM4 1089

ADA2 51816

CELF2 10659

CERKL 375298

CETP 1071

CFP 5199

CHAC1 79094

CHIT1 1118

CHN1 1123

CHRD 8646

CHRDL1 91851

CHRNA6 8973

CHST13 166012

CHST2 9435

CIITA 4261

CILP 8483

CISH 1154

CLCF1 23529

CLEC10A 10462

CLEC11A 6320

CLEC12A 160364

CLEC14A 161198

CLEC1A 51267

CLEC3B 7123

CLEC4A 50856

CLEC4D 338339

CLEC4E 26253

CLEC4G 339390

CLEC4M 10332

CLEC5A 23601

CLEC6A 93978

CLEC9A 283420

CLECL1 160365

CLIC2 1193

CLIC3 9022

CLIC5 53405

CLIP3 25999

CLNK 116449

CLOCK 9575

CLTB 1212

CMA1 1215

CMAHP 8418

CMKLR1 1240

CMPK2 129607

CNFN 84518

CNR2 1269

CNRIP1 25927

CNTF 1270

CNTFR 1271

COL10A1 1300

COL11A1 1301

COL12A1 1303

COL14A1 7373

COL15A1 1306

COL18A1 80781

COL1A1 1277

COL1A2 1278

COL6A5 256076

COL3A1 1281

COL4A1 1282

COL4A2 1284

COL5A1 1289

COL5A2 1290

COL5A3 50509

COL6A1 1291

COL6A2 1292

COL6A3 1293

COL6A6 131873

COL8A1 1295

COLEC12 81035

CORIN 10699

CORO1A 11151

COTL1 23406

CPA3 1359

CPNE5 57699

CPVL 54504

CPXM1 56265

CPZ 8532

CR1 1378

CR1L 1379

CR2 1380

CRCT1 54544

CREB1 1385

CREB3L1 90993

CREBL2 1389

CRISPLD2 83716

CRLF2 64109

CRTAM 56253

CRYBB1 1414

CSF1 1435

CSF1R 1436

CSF2 1437

CSF2RA 1438

CSF2RB 1439

CSF3 1440

CSF3R 1441

CSGALNACT2 55454

CSMD2 114784

CST7 8530

CTF1 1489

CTGF 1490

CTHRC1 115908

CTLA4 1493

CTSB 1508

CTSE 1510

CTSG 1511

CTSK 1513

CTSL 1514

CTSO 1519

CTSS 1520

CTSW 1521

CTSZ 1522

CTTNBP2 83992

CX3CL1 6376

CX3CR1 1524

CXCL10 3627

CXCL11 6373

CXCL12 6387

CXCL1 2919

CXCL13 10563

CXCL16 58191

CXCL2 2920

CXCL3 2921

CXCL5 6374

CXCL6 6372

CXCL9 4283

CXCR1 3577

CXCR2 3579

CXCR2P1 3580

CXCR3 2833

CXCR4 7852

CXCR5 643

CXCR6 10663

ACKR3 57007

CXorf21 80231

CXorf36 79742

CXorf65 158830

CYBA 1535

CYBB 1536

CYFIP2 26999

CYP1B1 1545

CYP27A1 1593

CYSLTR1 10800

CYSLTR2 57105

CYTH4 27128

CYTIP 9595

DAAM2 23500

DAB2 1601

DACT1 51339

DACT3 147906

ACKR1 2532

DBH 1621

DCBLD1 285761

DCHS1 8642

DCN 1634

DDI2 84301

DDR2 4921

DDX58 23586

DDX60 55601

DENND1C 79958

DENND2A 27147

DERL3 91319

DHRS1 115817

DHRS9 10170

DIXDC1 85458

DKK2 27123

DLC1 10395

DLL4 54567

DMKN 93099

DMXL2 23312

DNAH8 1769

DNAJC5B 85479

DOCK10 55619

DOCK11 139818

DOCK2 1794

DOCK4 9732

DOCK8 81704

DOK1 1796

DOK2 9046

DOK3 79930

DOK5 55816

DOK6 220164

DPEP1 1800

DPEP2 64174

DPP8 54878

DPT 1805

DSC1 1823

DSG1 1828

DSP 1832

DUOXA1 90527

DUSP16 80824

DUSP4 1846

DYSF 8291

E2F5 1875

EBF1 1879

EBF2 64641

EBI3 10148

ECM2 1842

ECSCR 641700

EDA 1896

EDA2R 60401

EDAR 10913

EDNRA 1909

EDNRB 1910

EFEMP2 30008

EFTUD2 9343

EGF 1950

EGFR 1956

EHD2 30846

ADGRL4 64123

EMCN 51705

EMILIN1 11117

EMILIN2 84034

ADGRE2 30817

ADGRE4P 326342

ENG 2022

ENO3 2027

ENOX1 55068

ENPEP 2028

ENPP2 5168

ENPP3 5169

ENPP4 22875

ENTPD1 953

EOMES 8320

EP300 2033

EPCAM 4072

EPO 2056

EPOR 2057

EPS8 2059

EPS8L1 54869

EPSTI1 94240

ERAP1 51752

ERAP2 64167

ERN1 2081

ERP27 121506

ESAM 90952

ETS1 2113

ETV3 2117

ETV7 51513

EVI2A 2123

EVI2B 2124

EVPL 2125

F13A1 2162

F2R 2149

F5 2153

FABP3 2170

FCMR 9214

FAM105A 54491

FAM107A 11170

PCED1B 91523

DENND6B 414918

FAM129C 199786

FAM13C 220965

FAM155A 728215

FAM168A 23201

FAM171B 165215

FAM177B 400823

TVP23A 780776

CCSER1 401145

FAM198B 51313

FAM19A5 25817

FAM25A 643161

FAM25BP 100132929

CALHM5 254228

CALHM6 441168

PIEZO2 63895

STRIP2 57464

FAM46B 115572

FAM46C 54855

FAM49A 81553

NXPE4 54827

MINDY2 54629

RIPOR2 9750

FAM78A 286336

FAM83A 84985

FAM92B 339145

FAP 2191

FAS 355

FASLG 356

FAT4 79633

FBLN2 2199

FBLN5 10516

FBN1 2200

FBP1 2203

FBXL7 23194

FBXO6 26270

FCAR 2204

FCER1A 2205

FCER1G 2207

FCER2 2208

FCGBP 8857

FCGR1A 2209

FCGR1B 2210

FCGR1CP 100132417

FCGR2A 2212

FCGR2B 2213

FCGR2C 9103

FCGR3A 2214

FCGR3B 2215

FCGRT 2217

FCN1 2219

FCRL1 115350

FCRL2 79368

FCRL3 115352

FCRL4 83417

FCRL5 83416

FCRL6 343413

FCRLA 84824

FERMT2 10979

FERMT3 83706

FGD2 221472

FGD3 89846

FGD5 152273

FGF14 2259

FGF7 2252

FGL2 10875

FGR 2268

FHL5 9457

FIBIN 387758

FICD 11153

VEGFD 2277

FILIP1L 11259

FKBP11 51303

FKBP7 51661

FLI1 2313

ANKRD36BP2 645784

FLT1 2321

FLT3 2322

FLT3LG 2323

FLT4 2324

FLVCR2 55640

FMNL1 752

FMNL3 91010

FMOD 2331

FN1 2335

FNBP1 23048

FNDC1 84624

FNIP2 57600

FOLR2 2350

FOXP3 50943

FPR1 2357

FPR2 2358

FPR3 2359

FRZB 2487

FSCN1 6624

FSTL1 11167

FSTL3 10272

FUCA1 2517

FUT7 2529

FYB1 2533

FYN 2534

FZD4 8322

GAB3 139716

GALM 130589

GALNT15 117248

GAPT 202309

GAS7 8522

GATA1 2623

GATA2 2624

GATA3 2625

GATM 2628

GBGT1 26301

GBP1 2633

GBP2 2634

GBP4 115361

GBP5 115362

GCSAM 257144

GDF5 8200

GFI1 2672

GFRA3 2676

GGT1 2678

GGT5 2687

GGTA1P 2681

GHR 2690

GHRL 51738

GIMAP1 170575

GIMAP2 26157

GIMAP4 55303

GIMAP5 55340

GIMAP6 474344

GIMAP7 168537

GIMAP8 155038

GIPC3 126326

GIT2 9815

GJA4 2701

GJA5 2702

GJB2 2706

GJB3 2707

GJB5 2709

GJD3 125111

GLIPR2 152007

GLIS3 169792

GLRX 2745

COLGALT2 23127

GLT8D2 83468

GMFG 9535

GMIP 51291

GMPR 2766

GNA15 2769

GNAI2 2771

GNG11 2791

GNG2 54331

GNG7 2788

GNGT2 2793

GNLY 10578

GNS 2799

GPBAR1 151306

GPC5 2262

GPC6 10082

GPIHBP1 338328

ADGRG5 221188

ADGRF4 221393

ADGRF5 221395

ADGRA2 25960

GPR132 29933

ADGRD1 283383

GPR137B 7107

GPR141 353345

GPR15 2838

GPR157 80045

GPR171 29909

GPR174 84636

GPR18 2841

GPR183 1880

GPR25 2848

GPR34 2857

GPR35 2859

GPR4 2828

GPR55 9290

GPR65 8477

GPR78 27201

GPR82 27197

GPR84 53831

GPRIN3 285513

GPSM3 63940

GRAP 10750

GRAP2 9402

GREM1 26585

GRIN3A 116443

GSDMA 284110

GTF2A1 2957

GUCY1A2 2977

GUCY1A3 2982

GVINP1 387751

GYPC 2995

GZMA 3001

GZMB 3002

GZMH 2999

GZMK 3003

GZMM 3004

HAMP 57817

HAPLN3 145864

HAVCR1 26762

HAVCR2 84868

HCG26 352961

HCK 3055

HCLS1 3059

HCP5 10866

HCST 10870

HDC 3067

HECW2 57520

HEPH 9843

HEPHL1 341208

HERC6 55008

HERPUD1 9709

HEYL 26508

HFE 3077

HGF 3082

HIC1 3090

HIPK3 10114

HIST1H2AE 3012

HIST1H2AG 8969

HIST1H2AM 8336

HIST1H3H 8357

HK3 3101

HLA-A 3105

HLA-B 3106

HLA-C 3107

HLA-DMA 3108

HLA-DMB 3109

HLA-DOA 3111

HLA-DOB 3112

HLA-DPA1 3113

HLA-DPB1 3115

HLA-DPB2 3116

HLA-DQA1 3117

HLA-DQA2 3118

HLA-DQB1 3119

HLA-DQB2 3120

HLA-DRA 3122

HLA-DRB1 3123

HLA-DRB5 3127

HLA-DRB6 3128

HLA-E 3133

HLA-F 3134

HLA-G 3135

HLA-H 3136

HLX 3142

HMCN1 83872

ARHGAP45 23526

HMSD 284293

HNMT 3176

HPGD 3248

HPGDS 27306

HRH2 3274

HS3ST1 9957

HS3ST2 9956

HSD11B1 3290

HSD17B14 51171

HSH2D 84941

HSP90AA1 3320

HSP90AB1 3326

HSPA12B 116835

HSPA1A 3303

HSPA1B 3304

HSPA1L 3305

HSPA2 3306

HSPA4 3308

HSPA5 3309

HSPA6 3310

HSPA8 3312

HTR2A 3356

HTRA3 94031

HTRA4 203100

HVCN1 84329

HYDIN 54768

ICAM1 3383

ICAM2 3384

ICAM3 3385

ICK 22858

ICOS 29851

ICOSLG 23308

IDO1 3620

IDO2 169355

IFFO1 25900

IFI27 3429

IFI30 10437

IFI35 3430

IFI44 10561

IFI44L 10964

IFI6 2537

IFIH1 64135

IFIT2 3433

IFIT3 3437

IFIT5 24138

IFITM1 8519

IFITM3 10410

IFNA13 3447

IFNA21 3452

IFNAR1 3454

IFNAR2 3455

IFNB1 3456

IFNE 338376

IFNG 3458

IFNGR1 3459

IFNGR2 3460

IFNK 56832

IFNW1 3467

IGDCC4 57722

IGF1 3479

JCHAIN 3512

IGLL1 3543

IGSF10 285313

IGSF21 84966

IGSF6 10261

IKZF1 10320

IKZF3 22806

IL10 3586

IL10RA 3587

IL10RB 3588

IL11 3589

IL11RA 3590

IL12A 3592

IL12B 3593

IL12RB1 3594

IL12RB2 3595

IL13 3596

IL13RA1 3597

IL15 3600

IL15RA 3601

IL16 3603

IL17A 3605

IL17B 27190

IL17RA 23765

IL17RB 55540

IL18 3606

IL18BP 10068

IL18R1 8809

IL18RAP 8807

IL19 29949

IL1A 3552

IL1B 3553

IL36G 56300

IL1R1 3554

IL1R2 7850

IL1RAP 3556

IL1RL1 9173

IL1RN 3557

IL20 50604

IL20RA 53832

IL20RB 53833

IL21 59067

IL21R 50615

IL22 50616

IL22RA1 58985

IL22RA2 116379

IL2 3558

IL23A 51561

IL23R 149233

IL24 11009

IL27 246778

IFNL2 282616

IFNLR1 163702

IFNL1 282618

IL2RA 3559

IL2RB 3560

IL2RG 3561

IL32 9235

IL3RA 3563

IL4 3565

IL4I1 259307

IL4R 3566

IL5 3567

IL5RA 3568

IL6 3569

IL6R 3570

IL6ST 3572

IL7 3574

IL7R 3575

CXCL8 3576

IL9 3578

IL9R 3581

INHBA 3624

INHBB 3625

INHBC 3626

INHBE 83729

INMT 11185

INPP5D 3635

IPCEF1 26034

IQGAP2 10788

IRF1 3659

IRF4 3662

IRF7 3665

IRF8 3394

IRF9 10379

ISG15 9636

ISG20 3669

ISLR 3671

ITGA11 22801

ITGA1 3672

ITGA2B 3674

ITGA4 3676

ITGA5 3678

ITGA8 8516

ITGA9 3680

ITGAD 3681

ITGAL 3683

ITGAM 3684

ITGAX 3687

ITGB1 3688

ITGB2 3689

ITGB3 3690

ITGB7 3695

ITGBL1 9358

ITK 3702

ITM2A 9452

IVL 3713

JAK2 3717

JAK3 3718

JAKMIP1 152789

JAM2 58494

JAM3 83700

JMY 133746

JSRP1 126306

JUP 3728

KCNA3 3738

KCNAB2 8514

KCND2 3751

KCNE4 23704

KCNH2 3757

KCNJ10 3766

KCNJ8 3764

KCNK13 56659

KCNK6 9424

KCNMB1 3779

KCNN3 3782

KCNN4 3783

KCNT2 343450

KCTD12 115207

KDR 3791

FAM30A 9834

KIAA0368 23392

TESPA1 9840

KIAA0754 643314

JCAD 57608

KIAA1549 57670

TLDC1 57707

SHISAL1 85352

KIAA1755 85449

KIF21B 23046

KIR2DL1 3802

KIR2DL3 3804

KIR2DL4 3805

KIR2DS4 3809

KIR3DL1 3811

KIR3DL2 3812

KIR3DL3 115653

KIRREL1 55243

KIT 3815

KITLG 4254

KL 9365

KLHDC10 23008

KLHL11 55175

KLHL23 151230

KLHL6 89857

KLK7 5650

KLK9 284366

KLRB1 3820

KLRC1 3821

KLRC2 3822

KLRC3 3823

KLRC4 8302

KLRD1 3824

KLRG1 10219

KLRK1 22914

KMO 8564

KRT1 3848

KRT14 3861

KRT16 3868

KRT6A 3853

KRT6B 3854

KRT6C 286887

KRT78 196374

LAD1 3898

LAG3 3902

LAIR1 3903

LAIR2 3904

LAMA2 3908

LAMA4 3910

LAMC2 3918

LAP3 51056

LAPTM5 7805

LAT 27040

LAT2 7462

LATS1 9113

LATS2 26524

LAX1 54900

LCE3D 84648

LCK 3932

LCN10 414332

LCOR 84458

LCP1 3936

LCP2 3937

LDB2 9079

LEP 3952

LEPR 3953

LGALS2 3957

LGALS9 3965

LGI2 55203

LGMN 5641

LHFPL6 10186

LHFPL2 10184

LIF 3976

LIFR 3977

LIG3 3980

LILRA1 11024

LILRA2 11027

LILRA3 11026

LILRA4 23547

LILRA5 353514

LILRA6 79168

LILRB1 10859

LILRB2 10288

LILRB3 11025

LILRB4 11006

LILRB5 10990

LILRP2 79166

LIMD2 80774

LIME1 54923

LIMS1 3987

LIPA 3988

LMOD1 25802

LMTK2 22853

UNQ6494 100129066

FAM83A-AS1 100131726

LINC00426 100188949

PCED1B-AS1 100233209

TNFRSF14-AS1 115110

LINC00654 149837

LINC00926 283663

SMIM1 388588

LGALS17A 400696

MIR31HG 554202

LOC606724 606724

LOC653786 653786

LOC730101 730101

BMS1P20 96610

LOXL2 4017

LOXL3 84695

LPAR4 2846

LPL 4023

PLPPR4 9890

LPXN 9404

LRCH2 57631

LRMP 4033

LRP6 4040

LRRC15 131578

LRRC17 10234

LRRC25 126364

LRRC32 2615

NRROS 375387

LSAMP 4045

LSP1 4046

LST1 7940

LTA 4049

LTB 4050

LTBP2 4053

LTBR 4055

LTC4S 4056

LUM 4060

LY86 9450

LY9 4063

LY96 23643

LYL1 4066

LYN 4067

LYPD3 27076

LYPD5 284348

LYVE1 10894

LYZ 4069

MAGEL2 54551

MAN1A1 4121

MAN1A2 10905

MAN1C1 57134

MAOB 4129

MAP1LC3C 440738

MAP3K2 10746

MAP4K1 11184

MAP7D1 55700

43160 55016

43167 220972

MARCO 8685

MBNL3 55796

SLC25A53 401612

MCOLN2 255231

MED13 9969

MED13L 23389

MEF2B 100271849

MEF2C 4208

MEI1 150365

MEOX2 4223

MET 4233

MFAP3 4238

MFAP4 4239

MFNG 4242

MFRP 83552

MFSD7 84179

MGAT4A 11320

MGAT5 4249

MZB1 51237

MGP 4256

NA 4276

MICAL2 9645

MICB 4277

MIR155HG 114614

MITF 4286

MLPH 79083

MMP12 4321

MMP1 4312

MMP14 4323

MMP16 4325

MMP2 4313

MMP25 64386

MMP3 4314

MMP9 4318

MMRN1 22915

MMRN2 79812

MNDA 4332

MPEG1 219972

MPL 4352

MPP1 4354

MR1 3140

MRC1 4360

MRGPRF 116535

MRO 83876

MRPL27 51264

MRPL55 128308

MRPS12 6183

MRPS21 54460

MRVI1 10335

MS4A14 84689

MS4A1 931

MS4A2 2206

MS4A4A 51338

MS4A6A 64231

MS4A7 58475

MSR1 4481

MSRB3 253827

MVP 9961

MX1 4599

MXD1 4084

MXRA8 54587

MYCT1 80177

MYEF2 50804

MYO1F 4542

MYO1G 64005

MYO7A 4647

MYO9A 4649

N4BP2 55728

N4BP2L1 90634

NAALADL1 10004

NAIP 4671

NAP1L3 4675

NAPSB 256236

NBEA 26960

NBEAL1 65065

NCCRP1 342897

NCF1 653361

NCF1B 654816

NCF1C 654817

NCF2 4688

NCF4 4689

NCKAP1L 3071

NCOA2 10499

NCR1 9437

NCR3 259197

NEGR1 257194

NFAM1 150372

NFATC2 4773

NFKB2 4791

NFKBID 84807

NFKBIE 4794

NFYA 4800

NFYB 4801

NFYC 4802

NHLRC2 374354

NHSL2 340527

NID2 22795

NIPAL4 348938

NKG7 4818

NLRC3 197358

NLRC4 58484

NLRC5 84166

NLRP12 91662

NLRP3 114548

NMI 9111

NOD2 64127

NOTCH4 4855

NOVA2 4858

NOX4 50507

NR1H3 10062

NR5A2 2494

NRP1 8829

NRXN3 9369

NT5E 4907

NTM 50863

NTNG2 84628

NTRK1 4914

OAS1 4938

OAS2 4939

OAS3 4940

OASL 8638

TENM3 55714

OGFRL1 79627

OGN 4969

OLFML1 283298

OLFML2B 25903

OLFML3 56944

OLR1 4973

OMD 4958

OSCAR 126014

OSM 5008

OSMR 9180

OTOA 146183

OVOL1 5017

P2RX1 5023

P2RX4 5025

P2RX5 5026

P2RX7 5027

P2RY10 27334

P2RY11 5032

P2RY12 64805

P2RY13 53829

P2RY14 9934

P2RY8 286530

P4HA3 283208

PABPC5 140886

PADI2 11240

PAFAH1B2 5049

PAG1 55824

PAK5 57144

PALM2-AKAP2 445815

PARM1 25849

PARP12 64761

PARP14 54625

PARP15 165631

PARP9 83666

PARVG 64098

PATL2 197135

PATZ1 23598

PBX4 80714

PCDH12 51294

PCDH17 27253

PCDH18 54510

PCDHGA12 26025

PCOLCE 5118

PCYOX1L 78991

PDCD1 5133

PDCD1LG2 80380

PDE1A 5136

PDE1B 5153

PDE3A 5139

PDE3B 5140

PDE4B 5142

PDE6G 5148

PDGFA 5154

PDGFB 5155

PDGFC 56034

PDGFRA 5156

PDGFRB 5159

PDGFRL 5157

PDIA2 64714

PDIA3 2923

PDZK1IP1 10158

PDZRN3 23024

PECAM1 5175

PEG3 5178

PFDN2 5202

PGLYRP4 57115

PGM5 5239

PHACTR1 221692

PIK3AP1 118788

PIK3CG 5294

PIK3R5 23533

PIK3R6 146850

PILRA 29992

PIM2 11040

PIP4K2A 5305

PKD2L1 9033

PKHD1L1 93035

PKIB 5570

PKP3 11187

PLA1A 51365

PLA2G2D 26279

PLA2G4E 123745

PLA2G7 7941

PLAC9 219348

PLCB2 5330

PLCB4 5332

PLCL1 5334

PLCL2 23228

PLD4 122618

PLEK2 26499

PLEK 5341

PLEKHM3 389072

PLEKHN1 84069

PLEKHO1 51177

PLEKHO2 80301

PLIN3 10226

PLVAP 83483

PLXDC1 57125

PLXNA4 91584

PLXNC1 10154

PLXND1 23129

PML 5371

PMP22 5376

PNLIPRP3 119548

PNMA2 10687

PNOC 5368

PODN 127435

POSTN 10631

POU2AF1 5450

POU2F2 5452

PLPP3 8613

PLPP4 196051

PLPP7 84814

PPBP 5473

PPFIA2 8499

PPL 5493

PPM1H 57460

PPM1M 132160

PPP1R13L 10848

PPP1R16B 26051

PPP1R9A 55607

PRAM1 84106

PRELP 5549

PREX1 57580

PREX2 80243

PRF1 5551

PRG2 5553

PRKAR2A 5576

PRKAR2B 5577

PRKCB 5579

PRKCQ 5588

PRKG1 5592

PRL 5617

PRLR 5618

PROCR 10544

PROM1 8842

PRRX1 5396

PRSS27 83886

PRTG 283659

PSAP 5660

PSMB10 5699

PSMB8 5696

PSMB9 5698

PSMC1 5700

PSMC2 5701

PSMC3 5702

PSMC4 5704

PSMC5 5705

PSMC6 5706

PSMD10 5716

PSMD11 5717

PSMD13 5719

PSMD14 10213

PSMD1 5707

PSMD2 5708

PSMD3 5709

PSMD4 5710

PSMD5 5711

PSMD6 9861

PSMD7 5713

PSMD8 5714

PSME1 5720

PSME2 5721

PSME3 10197

PSTPIP1 9051

PTAFR 5724

PTCRA 171558

PTGDR 5729

PTGDS 5730

PTGER2 5732

PTGFR 5737

PTGIR 5739

PTGIS 5740

PTH1R 5745

PTPN22 26191

PTPN6 5777

PTPN7 5778

PTPRB 5787

PTPRC 5788

PTPRCAP 5790

PTPRJ 5795

PTPRM 5797

PTPRO 5800

CAVIN1 284119

PUS10 150962

PVR 5817

PVRIG 79037

NECTIN2 5819

NECTIN3 25945

PYHIN1 149628

QPRT 23475

RAB20 55647

RAB33A 9363

RAB37 326624

RAB39A 54734

RAB39B 116442

RAB42 115273

RAB8B 51762

RAD23B 5887

RAD54L2 23132

RAET1E 135250

RAET1G 353091

RAET1L 154064

RAI2 10742

RAMP3 10268

RAPGEF2 9693

RAPGEF6 51735

RARRES2 5919

RARRES3 5920

RASAL3 64926

RASGRF2 5924

RASGRP2 10235

RASGRP3 25780

RASGRP4 115727

RASL12 51285

RASSF2 9770

RASSF3 283349

RASSF4 83937

RASSF5 83593

RASSF6 166824

RBM38 55544

RBP5 83758

RC3H2 54542

RCAN2 10231

RCN3 57333

RCSD1 92241

RDH12 145226

RECK 8434

REL 5966

RELB 5971

RELN 5649

RENBP 5973

REST 5978

RFTN1 23180

RFX5 5993

RFXANK 8625

RFXAP 5994

RGL1 23179

RGL4 266747

RGPD1 400966

RGS13 6003

RGS1 5996

RGS18 64407

RGS5 8490

RHOD 29984

RHOH 399

RHOJ 57381

RIF1 55183

RIMKLA 284716

RIN1 9610

RIN3 79890

CARMIL2 146206

RNASE1 6035

RNASE2 6036

RNASE6 6039

RNASE7 84659

RNF125 54941

RNF166 115992

RNF180 285671

RNF222 643904

LAMTOR2 28956

ROBO4 54538

ROCK2 9475

ROR1 4919

RPS6KA4 8986

RRN3P2 653390

RSAD2 91543

RTKN2 219790

RTN1 6252

RTP4 64108

RUFY4 285180

NA 84127

RUNX1T1 862

RUNX3 864

S100A12 6283

S100A16 140576

S100A2 6273

S100A7 6278

S100A7A 338324

S100A8 6279

S100A9 6280

S100B 6285

S1PR1 1901

S1PR4 8698

SALL2 6297

SAMD14 201191

SAMD3 154075

SAMD9 54809

SAMD9L 219285

SAMHD1 25939

SAMSN1 64092

SARDH 1757

SASH3 54440

SBNO1 55206

SBSN 374897

SCARF1 8578

SCARF2 91179

SCEL 8796

SCML4 256380

SCN7A 6332

SCUBE3 222663

SDC2 6383

SDCBP2 27111

SDR9C7 121214

SDS 10993

SDSL 113675

SEC24A 10802

SEC24D 9871

SECISBP2L 9728

SECTM1 6398

SELENBP1 8991

SELL 6402

SELP 6403

SELPLG 6404

43344 1731

43349 23157

SERINC5 256987

SERPINA1 5265

SERPINE1 5054

SERPINF1 5176

SERPING1 710

SFMBT2 57713

SFN 2810

SFRP2 6423

SFTPB 6439

SGCD 6444

SGIP1 84251

POMK 84197

PEAK1 79834

SH2B3 10019

SH2D1A 4068

SH2D2A 9047

SH2D3C 10044

SH2D5 400745

SHE 126669

SEM1 7979

SIGLEC10 89790

SIGLEC11 114132

SIGLEC12 89858

SIGLEC14 100049587

SIGLEC1 6614

SIGLEC5 8778

SIGLEC6 946

SIGLEC7 27036

SIGLEC8 27181

SIGLEC9 27180

SIGLEC17P 284367

SIRPB1 10326

SIRPB2 284759

SIRPG 55423

SIT1 27240

SKAP1 8631

SLA2 84174

SLA 6503

SLAMF1 6504

SLAMF6 114836

SLAMF7 57823

SLAMF8 56833

SLC10A2 6555

SLC11A1 6556

SLC12A3 6559

SLC15A3 51296

SLC17A9 63910

SLC18A2 6571

SLC1A7 6512

SLC24A4 123041

SLC25A45 283130

SLC29A3 55315

SLC2A5 6518

SLC34A2 10568

SLC39A2 29986

SLC45A3 85414

SLC6A12 6539

SLC7A7 9056

SLC8A1 6546

SLCO2B1 11309

SLCO5A1 81796

NA 342615

SLIT2 9353

SLIT3 6586

SLURP1 57152

SMAP2 64744

SMPDL3B 27293

SNAI3 333929

SNED1 25992

SNRPF 6636

SNTB1 6641

SNX20 124460

SOD3 6649

SON 6651

SOX17 64321

SOX5 6660

SP100 6672

SP110 3431

SP140 11262

SPAG4 6676

SPARC 6678

SPARCL1 8404

SPATA13 221178

SPI1 6688

SPIB 6689

SPN 6693

SPNS3 201305

SPOCK2 9806

SPON1 10418

SPRR1A 6698

SPRR1B 6699

SPRR2D 6703

SPRR2E 6704

SPRR2G 6706

SPRY1 10252

SRGN 5552

SSC5D 284297

SSTR3 6753

ST3GAL2 6483

ST3GAL5 8869

ST3GAL6 10402

ST6GAL1 6480

ST6GALNAC3 256435

ST8SIA4 7903

STAB1 23166

STAC3 246329

STAP1 26228

STARD13 90627

STARD8 9754

STAT1 6772

STAT4 6775

STAT5A 6776

STK17B 9262

STK33 65975

STRN 6801

STXBP6 29091

SUCNR1 56670

SULF1 23213

SULT1C2 6819

SULT1C4 27233

SULT2B1 6820

SUSD3 203328

SVOPL 136306

SYNE1 23345

SYT11 23208

SYTL3 94120

TAGAP 117289

TAOK1 57551

TAP1 6890

TAP2 6891

TAPBP 6892

TAPBPL 55080

TARP 445347

TBC1D10C 374403

TBCEL 219899

TBX21 30009

TBXA2R 6915

TBXAS1 6916

TCEAL7 56849

TCIRG1 10312

TCL1A 8115

TCN2 6948

TEK 7010

TESC 54997

TFEC 22797

TGFB1 7040

TGFB2 7042

TGFB3 7043

TGFBI 7045

TGFBR1 7046

TGFBR2 7048

TGFBRAP1 9392

TGM1 7051

TGM2 7052

THBS1 7057

THBS2 7058

THEMIS 387357

THPO 7066

THSD7A 221981

THY1 7070

TIE1 7075

TIFAB 497189

TIGIT 201633

TIMD4 91937

PAM16 51025

TIMM50 92609

TIMP2 7077

TIMP3 7078

TLR10 81793

TLR1 7096

TLR4 7099

TLR5 7100

TLR7 51284

TLR8 51311

TLR9 54106

TM4SF18 116441

TM6SF1 53346

DCSTAMP 81501

TMC8 147138

TMEM106A 113277

TMEM119 338773

TMEM140 55281

IGFLR1 79713

TMEM150B 284417

TMEM156 80008

TMEM170B 100113407

TMEM176A 55365

TMEM176B 28959

TMEM200A 114801

TMEM204 79652

TMEM229B 161145

TMEM233 387890

TMEM26 219623

TMEM47 83604

TMEM79 84283

SYNDIG1 79953

TMIGD2 126259

TNF 7124

TNFAIP6 7130

TNFAIP8L2 79626

TNFRSF10A 8797

TNFRSF10B 8795

TNFRSF10C 8794

TNFRSF10D 8793

TNFRSF11A 8792

TNFRSF11B 4982

TNFRSF12A 51330

TNFRSF13B 23495

TNFRSF13C 115650

TNFRSF14 8764

TNFRSF17 608

TNFRSF18 8784

TNFRSF1A 7132

TNFRSF1B 7133

TNFRSF25 8718

TNFRSF4 7293

TNFRSF6B 8771

TNFRSF8 943

TNFRSF9 3604

TNFSF10 8743

TNFSF11 8600

TNFSF12 8742

TNFSF12-TNFSF13 407977

TNFSF13 8741

TNFSF13B 10673

TNFSF14 8740

TNFSF15 9966

TNFSF18 8995

TNFSF4 7292

TNFSF8 944

TNFSF9 8744

TNIK 23043

TNIP3 79931

TNN 63923

TNNT2 7139

TNS3 64759

TOX 9760

TPK1 27010

TPSAB1 7177

TPSB2 64499

TPSD1 23430

TPSG1 25823

TRAF1 7185

TRAF3IP3 80342

TRANK1 9881

TRAT1 50852

TREM1 54210

TREM2 54209

TREML1 340205

TRIM21 6737

TRIM22 10346

TRIM61 391712

TRPC4AP 26133

TRPV2 51393

TRPV3 162514

TSHR 7253

TSHZ3 57616

TSLP 85480

TSPAN11 441631

TSPAN32 10077

TSPAN4 7106

TTBK2 146057

TTC16 158248

TTC21B 79809

TTC24 164118

TTC37 9652

TUBA4A 7277

TUBB6 84617

TXK 7294

NME8 51314

TYMP 1890

TYROBP 7305

UBA7 7318

UBASH3A 53347

UBD 10537

UBE2L6 9246

UBR1 197131

UBXN11 91544

UBXN1 51035

UCP2 7351

UHMK1 127933

ULBP1 80329

ULBP2 80328

ULBP3 79465

UNC13D 201294

UNC5C 8633

UNC93B1 81622

USHBP1 83878

USP12 219333

USP51 158880

UTS2 10911

VAMP5 10791

VASH1 22846

VAV1 7409

VCAM1 7412

VCAN 1462

VEGFA 7422

VEGFB 7423

VEGFC 7424

VENTX 27287

VGLL3 389136

VIM 7431

VMO1 284013

VNN2 8875

VPREB3 29802

VPS37D 155382

VSIG4 11326

VWF 7450

WARS 7453

WAS 7454

WDFY4 57705

WFDC12 128488

WIPF1 7456

WISP1 8840

WNT2 7472

XAF1 54739

XBP1 7494

XCL1 6375

XCL2 6846

XCR1 2829

XKR8 55113

XPNPEP2 7512

ZAP70 7535

ZBP1 81030

ZBTB10 65986

ZBTB32 27033

ZC3H12D 340152

ZCCHC24 219654

ZDHHC20 253832

ZEB1 6935

ZEB2 9839

ZFPM2 23414

ZKSCAN1 7586

ZMYND15 84225

ZNF185 7739

ZKSCAN8 7745

ZNF215 7762

ZNF366 167465

ZNF423 23090

ZNF469 84627

ZNF521 25925

ZNF620 253639

ZNF660 285349

ZNF671 79891

ZNF683 257101

ZNF804A 91752

ZNF80 7634

ZNF827 152485

ZNF831 128611

ZNF835 90485

LRP1 4035

EIF2A 83939

EIF2AK3 9451

EIF2AK2 5610

EIF2AK4 440275

EIF2AK1 27102

HMGB1 3146

ANXA1 301

PANX1 24145

P2RY2 5029

IFNA1 3439

IFNA2 3440

TLR3 7098

IFNAR1 3454

IFNAR2 3455

VTCN1 79679

HHLA2 11148
