# Supplementary material for: Identification of glioblastoma immune subtypes and immune landscape based on a large cohort
Source: Hereditas. 2021 Aug 19;158:30. doi: 10.1186/s41065-021-00193-x (PMC8377979; doi:10.1186/s41065-021-00193-x)
Supplement: Supplementary file 4 — Additional file 4. [file 41065_2021_193_MOESM4_ESM.docx]

Tag IS2 IS3 IS4 IS1 IS2.pvalue IS3.pvalue IS4.pvalue IS1.pvalue

RYR2 6 7 8 10 0.999173831789961 0.261528040209772 0.316252914030606 0.0417517572946482

TTN 16 17 15 36 0.999999987568453 0.226559556591874 0.843690832425201 2.43970444231737e-10

PIK3CA 3 4 6 14 0.999972559289176 0.711547086013052 0.52122352530057 4.28778302794659e-05

PPEF2 1 0 2 6 0.994347358298145 1 0.598825208550144 0.00179923593433847

FAT2 2 4 5 8 0.999763161405716 0.411705624246847 0.369423451190061 0.0129424141521671

CTNNA3 0 0 4 1 1 1 0.00766526196931799 0.648204567890215

TTC17 0 1 1 3 1 0.610163927365809 0.696413818386389 0.0471593735603865

OR4D9 0 0 1 4 1 1 0.696413818386389 0.00483325080615788

ZNF605 0 0 2 3 1 1 0.284217063178254 0.0471593735603865

TEP1 1 0 4 2 0.981810335882373 1 0.0379766110990049 0.388631796595168

CHD8 0 0 5 4 1 1 0.0225016097501159 0.0667950494494009

NCR1 0 1 0 4 1 0.610163927365809 1 0.00483325080615788

LZTR1 1 3 3 8 0.999841330058873 0.487086329378885 0.646816596830816 0.00208435206666125

DEPDC5 1 0 1 4 0.967502612277654 1 0.761468000160734 0.0124634702142331

HRNR 3 3 5 7 0.996845067244519 0.621069187249463 0.321811444046566 0.032614103169732

GCKR 0 1 1 4 1 0.677788552210515 0.761468000160734 0.0124634702142331

VIT 1 0 2 5 0.989846180646667 1 0.529967391499114 0.00696117912301087

PSME4 1 0 1 4 0.967502612277654 1 0.761468000160734 0.0124634702142331

DYSF 1 2 3 5 0.998262593363086 0.587247231048392 0.418699801734448 0.0361232787589351

ADAMTS9 0 1 2 5 1 0.780354200079733 0.529967391499114 0.00696117912301087

ABTB1 0 0 1 5 1 1 0.761468000160734 0.00101814110212027

OTOP1 0 0 1 4 1 1 0.696413818386389 0.00483325080615788

LRRC66 0 1 2 4 1 0.733873138685818 0.453737297296426 0.0250155717660838

ANK2 7 3 4 9 0.935411402556304 0.788511206605815 0.755628889856866 0.0143635386974884

FAT4 5 2 5 9 0.984023054182207 0.903620452934011 0.4645842631583 0.00711075428178304

ENC1 3 1 4 6 0.978816625781951 0.931639069301714 0.33743772632839 0.029299598312284

GABRG2 0 0 2 3 1 1 0.284217063178254 0.0471593735603865

SLC44A4 0 0 2 3 1 1 0.284217063178254 0.0471593735603865

ZNF318 2 1 5 9 0.999281817740843 0.962249901033029 0.275148355829698 0.00111272204598869

AIM1 3 1 3 6 0.966795114671163 0.916803292852299 0.540452432560751 0.0195839258529907

LAMA4 0 1 2 5 1 0.780354200079733 0.529967391499114 0.00696117912301087

HIVEP2 1 0 7 6 0.999710122222941 1 0.0133172950819115 0.029299598312284

AHR 1 0 3 5 0.994347358298145 1 0.288965031820512 0.0134136081088514

PIK3CG 2 2 2 7 0.993867179062387 0.683076950314477 0.800045007743114 0.00388515566452808

HTR5A 1 0 1 3 0.942095563694729 1 0.696413818386389 0.0471593735603865

ST18 1 0 1 6 0.989846180646667 1 0.853154887657977 0.000702323006745593

TG 1 1 3 10 0.999841330058873 0.943872077952986 0.646816596830816 3.61050763417572e-05

DENND3 0 0 2 5 1 1 0.453737297296426 0.00304939405034507

ZBTB5 2 0 3 9 0.996375604609126 1 0.595820079715031 0.000147601388816014

RGS3 1 0 2 5 0.989846180646667 1 0.529967391499114 0.00696117912301087

C10orf12 3 2 2 7 0.978816625781951 0.723938199009901 0.834082495747794 0.0066296959072663

OR52M1 2 1 1 5 0.953836759434847 0.818848824808028 0.884946097546456 0.0134136081088514

MRGPRX1 0 1 1 4 1 0.677788552210515 0.761468000160734 0.0124634702142331

NXF1 0 1 1 3 1 0.610163927365809 0.696413818386389 0.0471593735603865

FGF23 1 0 0 4 0.942095563694729 1 1 0.00483325080615788

TMEM132D 0 2 3 8 1 0.683076950314477 0.540452432560751 0.000573178831519092

NRXN3 1 1 2 5 0.994347358298145 0.818848824808028 0.598825208550144 0.0134136081088514

THBS1 1 1 4 6 0.999040806752537 0.898824343426312 0.231304350998932 0.0123383396121618

RASGRF1 0 0 2 4 1 1 0.371142909514663 0.0124634702142331

IFT140 1 0 1 4 0.967502612277654 1 0.761468000160734 0.0124634702142331

PLCG2 4 2 6 1 0.888004127265872 0.683076950314477 0.0346989276250299 0.936582941917419

SETBP1 1 1 2 6 0.996861809262073 0.850706445272823 0.659995881370045 0.00384176345468385

ZBTB7C 1 1 0 3 0.942095563694729 0.610163927365809 1 0.0471593735603865

ZNFX1 3 0 3 7 0.966795114671163 1 0.540452432560751 0.00388515566452808

FRMPD4 1 1 2 11 0.999841330058873 0.943872077952986 0.862892634042714 3.07780731051722e-06

ITIH6 0 1 1 7 1 0.818848824808028 0.884946097546456 0.00015386654294915

SLITRK4 1 0 1 5 0.981810335882373 1 0.812756450638119 0.00304939405034507

PCDHB7 2 1 0 5 0.925559422532313 0.780354200079733 1 0.00696117912301087

PCLO 4 3 4 8 0.990237224261604 0.660324855312454 0.596347119134989 0.0129424141521671

GRM3 2 3 2 7 0.996375604609126 0.437909458141937 0.834082495747794 0.0066296959072663

CUBN 0 2 3 5 1 0.532130240624871 0.354142517270538 0.0229854527630189

LRRK1 1 2 4 6 0.999471942393383 0.683076950314477 0.28354001670918 0.0195839258529907

MYO15A 1 0 2 6 0.994347358298145 1 0.598825208550144 0.00179923593433847

NF1 3 8 4 19 0.99999951001391 0.201120489090241 0.95689325616054 2.01182972841599e-07

SLFN11 1 0 2 5 0.989846180646667 1 0.529967391499114 0.00696117912301087

HNF4A 1 0 2 4 0.981810335882373 1 0.453737297296426 0.0250155717660838

KCNB1 2 0 0 3 0.71710543467726 1 1 0.0471593735603865

CSMD2 5 1 5 9 0.976112959885622 0.979299543008922 0.417239509792742 0.00476636840424171

PCDHA2 2 0 2 4 0.925559422532313 1 0.529967391499114 0.0430699644091567

SYNE1 6 6 12 9 0.999674217396857 0.507955426571899 0.0239974111762199 0.137214545409923

EGFR 16 21 7 28 0.999993823346937 0.00223467866730241 0.998886490610246 2.0583026050048e-06

DLGAP2 1 0 0 5 0.967502612277654 1 1 0.00101814110212027

PTEN 19 17 14 41 0.999999970059545 0.367143976340208 0.962360135921959 1.58073472528006e-13

TP53 16 16 32 27 0.99999999979946 0.497097450442218 0.000108431446246039 0.00148324705182313

FCRL5 1 0 1 4 0.967502612277654 1 0.761468000160734 0.0124634702142331

MAP4K3 0 0 1 4 1 1 0.696413818386389 0.00483325080615788

PIKFYVE 1 1 6 7 0.999841330058873 0.943872077952986 0.0706913424059458 0.0106068717677957

SETD2 2 0 6 7 0.997873212519892 1 0.0706913424059458 0.0106068717677957

ANKRD17 1 1 2 4 0.989846180646667 0.780354200079733 0.529967391499114 0.0430699644091567

NIPBL 0 0 3 4 1 1 0.164656940060257 0.0250155717660838

ELOVL7 0 0 2 3 1 1 0.284217063178254 0.0471593735603865

TMEM2 2 0 5 3 0.971717299622785 1 0.0377011024044698 0.282576009947826

ARNTL2 0 1 2 4 1 0.733873138685818 0.453737297296426 0.0250155717660838

AXIN1 1 1 1 4 0.981810335882373 0.733873138685818 0.812756450638119 0.0250155717660838

KIAA0100 1 0 4 2 0.981810335882373 1 0.0379766110990049 0.388631796595168

NETO1 0 1 2 4 1 0.733873138685818 0.453737297296426 0.0250155717660838

OR7D2 1 0 1 3 0.942095563694729 1 0.696413818386389 0.0471593735603865

BCOR 1 0 3 7 0.998262593363086 1 0.418699801734448 0.00102651502698752

UBAP2L 1 0 1 3 0.942095563694729 1 0.696413818386389 0.0471593735603865

CACNA1S 1 2 1 8 0.999040806752537 0.637560622653752 0.944978827634011 0.000258466100780682

SCN1A 4 0 3 6 0.888004127265872 1 0.540452432560751 0.0195839258529907

COL6A3 3 6 4 12 0.999917560141795 0.237807796440428 0.815320451514976 0.000455950095276326

TLR2 0 0 5 4 1 1 0.0225016097501159 0.0667950494494009

PIK3R1 2 6 7 16 0.999999799376563 0.439937714707292 0.491359027832953 1.08466519502635e-05

PKD1L1 3 0 3 7 0.966795114671163 1 0.540452432560751 0.00388515566452808

OR52N1 0 0 1 4 1 1 0.696413818386389 0.00483325080615788

SIK3 1 1 2 4 0.989846180646667 0.780354200079733 0.529967391499114 0.0430699644091567

BRCA1 1 0 1 3 0.942095563694729 1 0.696413818386389 0.0471593735603865

HCN1 0 2 1 6 1 0.47239720225338 0.884946097546456 0.00179923593433847

NOX4 2 3 0 0 0.71710543467726 0.0364632165930925 1 1

HYDIN 3 5 3 7 0.996845067244519 0.174348583853654 0.772312489716845 0.032614103169732

GSG2 0 2 2 4 1 0.408505708928411 0.529967391499114 0.0430699644091567

HNF1B 0 1 2 4 1 0.733873138685818 0.453737297296426 0.0250155717660838

MUC16 9 7 9 16 0.999367257468474 0.573381066673456 0.511070768793198 0.000821118473251273

COL6A2 0 4 2 1 1 0.0178288923408584 0.453737297296426 0.769643459130453

ZCCHC12 1 1 1 5 0.989846180646667 0.780354200079733 0.853154887657977 0.00696117912301087

CACNA2D1 0 1 3 4 1 0.780354200079733 0.225047014688361 0.0430699644091567

KCNU1 1 0 6 1 0.989846180646667 1 0.00140026354602069 0.813821425872558

RB1 4 8 4 12 0.999874671874777 0.0805022378363908 0.882463364392552 0.00166368802691029

ZFP28 2 0 4 1 0.88165627064934 1 0.0379766110990049 0.769643459130453

SLC6A14 0 1 1 3 1 0.610163927365809 0.696413818386389 0.0471593735603865

FLG 7 4 14 17 0.999979668027461 0.955561171184043 0.032757570571476 0.000306488789398221

RBM15B 1 0 1 3 0.942095563694729 1 0.696413818386389 0.0471593735603865

PEX1 1 1 6 0 0.989846180646667 0.780354200079733 0.00140026354602069 1

PALM2-AKAP2 0 0 1 9 1 1 0.909940566010502 1.38829185638483e-06

DIP2C 1 0 2 6 0.994347358298145 1 0.598825208550144 0.00179923593433847

TLR7 0 0 6 4 1 1 0.00730211709576205 0.0960014472403224

UBR4 1 0 4 9 0.999710122222941 1 0.33743772632839 0.000147601388816014

TMCO4 0 0 2 3 1 1 0.284217063178254 0.0471593735603865

MYOM3 2 0 4 1 0.88165627064934 1 0.0379766110990049 0.769643459130453

CLCA1 0 1 2 5 1 0.780354200079733 0.529967391499114 0.00696117912301087

SLC6A17 1 0 1 3 0.942095563694729 1 0.696413818386389 0.0471593735603865

POGZ 0 1 1 4 1 0.677788552210515 0.761468000160734 0.0124634702142331

NUP210L 1 3 1 5 0.996861809262073 0.233465048767417 0.909940566010502 0.0229854527630189

DCST1 0 0 2 3 1 1 0.284217063178254 0.0471593735603865

INSRR 1 0 3 4 0.989846180646667 1 0.225047014688361 0.0430699644091567

MTR 0 0 3 4 1 1 0.164656940060257 0.0250155717660838

AGBL5 0 0 2 3 1 1 0.284217063178254 0.0471593735603865

SLC4A5 0 1 1 4 1 0.677788552210515 0.761468000160734 0.0124634702142331

PTPN4 0 0 4 1 1 1 0.00766526196931799 0.648204567890215

LY75 2 2 2 6 0.989702246079122 0.637560622653752 0.760107688484289 0.0123383396121618

XIRP2 2 2 3 7 0.996375604609126 0.723938199009901 0.595820079715031 0.0066296959072663

MYO1B 2 2 2 5 0.982853968251951 0.587247231048392 0.713617232400177 0.0361232787589351

AOX1 0 0 4 8 1 1 0.231304350998932 0.000258466100780682

TTLL4 1 0 1 3 0.942095563694729 1 0.696413818386389 0.0471593735603865

SCG2 0 0 1 4 1 1 0.696413818386389 0.00483325080615788

XYLB 1 0 2 4 0.981810335882373 1 0.453737297296426 0.0250155717660838

CCR9 0 1 3 6 1 0.850706445272823 0.354142517270538 0.00384176345468385

FBXW12 1 0 0 4 0.942095563694729 1 1 0.00483325080615788

TMF1 2 0 1 4 0.88165627064934 1 0.812756450638119 0.0250155717660838

CCDC80 2 0 1 4 0.88165627064934 1 0.812756450638119 0.0250155717660838

ARHGAP31 2 0 1 7 0.971717299622785 1 0.909940566010502 0.000437550920774896

SEMA5B 0 0 3 4 1 1 0.164656940060257 0.0250155717660838

KALRN 0 1 2 6 1 0.818848824808028 0.598825208550144 0.00179923593433847

CLSTN2 0 0 4 4 1 1 0.0640068156800842 0.0430699644091567

TRIM42 0 2 0 3 1 0.202753277686633 1 0.0471593735603865

IGSF10 1 0 4 5 0.996861809262073 1 0.136884232437118 0.0229854527630189

SI 1 0 1 5 0.981810335882373 1 0.812756450638119 0.00304939405034507

BCHE 0 0 2 5 1 1 0.453737297296426 0.00304939405034507

TNIK 2 0 2 4 0.925559422532313 1 0.529967391499114 0.0430699644091567

UGT2A1 0 2 1 4 1 0.341281033358699 0.812756450638119 0.0250155717660838

FRAS1 2 4 2 6 0.996375604609126 0.201362482571807 0.834082495747794 0.029299598312284

THAP9 0 0 1 4 1 1 0.696413818386389 0.00483325080615788

HPSE 0 2 0 3 1 0.202753277686633 1 0.0471593735603865

GPRIN3 2 0 3 8 0.993867179062387 1 0.540452432560751 0.000573178831519092

EGF 0 2 2 4 1 0.408505708928411 0.529967391499114 0.0430699644091567

DDX60 0 0 1 4 1 1 0.696413818386389 0.00483325080615788

ADAM29 3 3 4 9 0.998084037803816 0.660324855312454 0.596347119134989 0.00307477273225714

TRIO 2 1 3 9 0.997873212519892 0.943872077952986 0.646816596830816 0.0003148458575274

PDZD2 4 2 7 8 0.99606128710228 0.903620452934011 0.126572716279669 0.025336100262477

NUP155 0 0 2 5 1 1 0.453737297296426 0.00304939405034507

DAB2 0 1 2 5 1 0.780354200079733 0.529967391499114 0.00696117912301087

VCAN 2 2 5 6 0.997873212519892 0.760376948183903 0.187847942788757 0.0417614160086095

ADGRV1 3 2 4 6 0.986641153189149 0.760376948183903 0.391834011251894 0.0417614160086095

CHSY3 1 2 2 5 0.996861809262073 0.532130240624871 0.659995881370045 0.0229854527630189

KDM3B 2 0 4 9 0.997873212519892 1 0.391834011251894 0.0003148458575274

PCDHGA8 0 0 2 3 1 1 0.284217063178254 0.0471593735603865

PCDHGA9 0 0 4 2 1 1 0.0193357288484222 0.313161299214229

PCDHGA11 0 0 2 5 1 1 0.453737297296426 0.00304939405034507

PCDHGC3 0 0 1 4 1 1 0.696413818386389 0.00483325080615788

GCM2 1 0 1 3 0.942095563694729 1 0.696413818386389 0.0471593735603865

NEDD9 1 0 2 4 0.981810335882373 1 0.453737297296426 0.0250155717660838

HIVEP1 1 1 5 3 0.996861809262073 0.850706445272823 0.0377011024044698 0.282576009947826

HIST1H3F 0 0 2 3 1 1 0.284217063178254 0.0471593735603865

CCHCR1 1 0 1 3 0.942095563694729 1 0.696413818386389 0.0471593735603865

SKIV2L 1 0 1 6 0.989846180646667 1 0.853154887657977 0.000702323006745593

ITPR3 1 1 3 6 0.998262593363086 0.877052366695266 0.418699801734448 0.00722006156791945

GCLC 1 0 3 4 0.989846180646667 1 0.225047014688361 0.0430699644091567

MDN1 1 3 5 6 0.999841330058873 0.487086329378885 0.187847942788757 0.0417614160086095

ROS1 1 1 3 6 0.998262593363086 0.877052366695266 0.418699801734448 0.00722006156791945

PTPRK 1 0 1 3 0.942095563694729 1 0.696413818386389 0.0471593735603865

LAMA2 1 0 4 5 0.996861809262073 1 0.136884232437118 0.0229854527630189

ARID1B 0 1 3 5 1 0.818848824808028 0.288965031820512 0.0134136081088514

IGF2R 1 0 2 7 0.996861809262073 1 0.659995881370045 0.000437550920774896

DNAH11 2 2 4 8 0.9987602514107 0.792682428777051 0.445703571585469 0.00355478680382881

BBS9 0 0 2 3 1 1 0.284217063178254 0.0471593735603865

DDC 0 2 0 3 1 0.202753277686633 1 0.0471593735603865

GTF2IRD1 1 0 1 3 0.942095563694729 1 0.696413818386389 0.0471593735603865

TRRAP 4 3 5 10 0.997533588970202 0.760701308008371 0.51086711558083 0.00256534425302484

SMURF1 1 1 3 5 0.996861809262073 0.850706445272823 0.354142517270538 0.0229854527630189

GJC3 1 0 1 3 0.942095563694729 1 0.696413818386389 0.0471593735603865

RELN 4 3 4 9 0.993768290355599 0.696677583216164 0.64112402147055 0.00476636840424171

PTPRZ1 1 1 4 7 0.999471942393383 0.916803292852299 0.28354001670918 0.00388515566452808

PRSS58 0 2 1 4 1 0.341281033358699 0.812756450638119 0.0250155717660838

CPA6 1 0 0 4 0.942095563694729 1 1 0.00483325080615788

PREX2 1 1 2 6 0.996861809262073 0.850706445272823 0.659995881370045 0.00384176345468385

SLC7A13 0 1 3 4 1 0.780354200079733 0.225047014688361 0.0430699644091567

FER1L6 2 0 7 4 0.993867179062387 1 0.00799522933973795 0.210859904455178

TONSL 0 0 2 4 1 1 0.371142909514663 0.0124634702142331

PRUNE2 1 2 4 6 0.999471942393383 0.683076950314477 0.28354001670918 0.0195839258529907

C9orf64 0 1 2 4 1 0.733873138685818 0.453737297296426 0.0250155717660838

NFIL3 2 0 1 5 0.925559422532313 1 0.853154887657977 0.00696117912301087

ANKS6 0 0 4 3 1 1 0.0379766110990049 0.124485055761882

SVEP1 0 0 2 4 1 1 0.371142909514663 0.0124634702142331

ITIH5 2 0 3 6 0.982853968251951 1 0.418699801734448 0.00722006156791945

FAM171A1 2 0 0 5 0.88165627064934 1 1 0.00304939405034507

SVIL 1 0 2 5 0.989846180646667 1 0.529967391499114 0.00696117912301087

KIF20B 0 1 2 4 1 0.733873138685818 0.453737297296426 0.0250155717660838

PLCE1 2 1 3 6 0.989702246079122 0.898824343426312 0.481164951979619 0.0123383396121618

TLL2 1 2 1 6 0.996861809262073 0.532130240624871 0.909940566010502 0.00384176345468385

R3HCC1L 0 0 2 3 1 1 0.284217063178254 0.0471593735603865

PKD2L1 0 0 2 3 1 1 0.284217063178254 0.0471593735603865

SEC31B 0 0 4 5 1 1 0.0972109804240587 0.0134136081088514

SORCS3 0 0 2 3 1 1 0.284217063178254 0.0471593735603865

KCNA4 0 1 3 6 1 0.850706445272823 0.354142517270538 0.00384176345468385

AHNAK 4 3 4 12 0.998469058749099 0.788511206605815 0.755628889856866 0.000162138156178482

SCYL1 1 0 1 4 0.967502612277654 1 0.761468000160734 0.0124634702142331

SPTBN2 0 0 3 5 1 1 0.225047014688361 0.00696117912301087

ARHGAP20 0 0 2 3 1 1 0.284217063178254 0.0471593735603865

NLRX1 0 0 2 3 1 1 0.284217063178254 0.0471593735603865

PUS3 1 0 1 3 0.942095563694729 1 0.696413818386389 0.0471593735603865

CCNT1 0 0 1 5 1 1 0.761468000160734 0.00101814110212027

GALNT6 1 3 2 5 0.998262593363086 0.284103783719027 0.713617232400177 0.0361232787589351

MAP3K12 2 1 1 4 0.925559422532313 0.780354200079733 0.853154887657977 0.0430699644091567

TMCC3 1 0 2 4 0.981810335882373 1 0.453737297296426 0.0250155717660838

STAB2 2 1 3 8 0.996375604609126 0.931639069301714 0.595820079715031 0.00114061542178986

POLR3B 0 0 2 3 1 1 0.284217063178254 0.0471593735603865

POLE 2 1 2 5 0.971717299622785 0.850706445272823 0.659995881370045 0.0229854527630189

PARP4 2 2 3 7 0.996375604609126 0.723938199009901 0.595820079715031 0.0066296959072663

AKAP11 0 1 5 8 1 0.931639069301714 0.148795742661153 0.00114061542178986

PCDH20 1 0 2 5 0.989846180646667 1 0.529967391499114 0.00696117912301087

TM9SF2 1 0 1 3 0.942095563694729 1 0.696413818386389 0.0471593735603865

EML1 1 0 1 5 0.981810335882373 1 0.812756450638119 0.00304939405034507

HERC2 1 0 3 9 0.999471942393383 1 0.540452432560751 6.17706440691746e-05

GJD2 0 0 0 6 1 1 1 3.44103354593332e-05

MAPKBP1 2 2 4 6 0.996375604609126 0.723938199009901 0.33743772632839 0.029299598312284

DUOX2 2 1 2 8 0.993867179062387 0.916803292852299 0.800045007743114 0.000573178831519092

SLC30A4 0 1 1 3 1 0.610163927365809 0.696413818386389 0.0471593735603865

IGDCC3 0 0 2 3 1 1 0.284217063178254 0.0471593735603865

ALPK3 1 0 2 4 0.981810335882373 1 0.453737297296426 0.0250155717660838

IGF1R 1 1 2 5 0.994347358298145 0.818848824808028 0.598825208550144 0.0134136081088514

TSC2 0 0 1 4 1 1 0.696413818386389 0.00483325080615788

SRRM2 2 2 3 7 0.996375604609126 0.723938199009901 0.595820079715031 0.0066296959072663

CREBBP 2 1 2 5 0.971717299622785 0.850706445272823 0.659995881370045 0.0229854527630189

GRIN2A 3 7 3 7 0.998845640294677 0.0360654135240176 0.834279286447483 0.0579256402085188

TXNDC11 0 0 2 3 1 1 0.284217063178254 0.0471593735603865

DNAH3 1 6 4 12 0.999998851710787 0.178336933652811 0.755628889856866 0.000162138156178482

KCTD19 1 0 1 3 0.942095563694729 1 0.696413818386389 0.0471593735603865

VAC14 1 0 1 4 0.967502612277654 1 0.761468000160734 0.0124634702142331

CDYL2 0 1 1 3 1 0.610163927365809 0.696413818386389 0.0471593735603865

ZZEF1 1 1 3 7 0.999040806752537 0.898824343426312 0.481164951979619 0.00210178352367709

MYH2 3 2 2 6 0.966795114671163 0.683076950314477 0.800045007743114 0.0195839258529907

MAP2K3 1 0 0 5 0.967502612277654 1 1 0.00101814110212027

ACACA 1 1 3 5 0.996861809262073 0.850706445272823 0.354142517270538 0.0229854527630189

MLLT6 0 0 2 3 1 1 0.284217063178254 0.0471593735603865

NAGLU 2 0 2 5 0.953836759434847 1 0.598825208550144 0.0134136081088514

KIF2B 1 2 6 8 0.999952875982995 0.821175177738358 0.122410068862699 0.00572551103302004

ABCA8 0 0 2 4 1 1 0.371142909514663 0.0124634702142331

ACOX1 1 0 0 5 0.967502612277654 1 1 0.00101814110212027

RNF213 2 1 6 7 0.9987602514107 0.953951387898049 0.0946176903714496 0.01609182673977

ANKRD12 0 0 2 4 1 1 0.371142909514663 0.0124634702142331

GAREM 1 1 1 4 0.981810335882373 0.733873138685818 0.812756450638119 0.0250155717660838

MC4R 0 0 2 3 1 1 0.284217063178254 0.0471593735603865

ZNF57 0 0 2 3 1 1 0.284217063178254 0.0471593735603865

ADGRE1 2 2 1 5 0.971717299622785 0.532130240624871 0.909940566010502 0.0229854527630189

OR2Z1 0 1 2 4 1 0.733873138685818 0.453737297296426 0.0250155717660838

BRD4 1 0 3 5 0.994347358298145 1 0.288965031820512 0.0134136081088514

CPAMD8 1 0 3 4 0.989846180646667 1 0.225047014688361 0.0430699644091567

WDR62 0 1 1 3 1 0.610163927365809 0.696413818386389 0.0471593735603865

CYP2A13 0 0 1 6 1 1 0.812756450638119 0.00020563992283035

ZNF229 3 0 1 4 0.749946815000421 1 0.853154887657977 0.0430699644091567

DHX34 0 0 2 4 1 1 0.371142909514663 0.0124634702142331

ZNF665 1 0 3 4 0.989846180646667 1 0.225047014688361 0.0430699644091567

PDYN 0 0 1 4 1 1 0.696413818386389 0.00483325080615788

JAG1 1 0 3 4 0.989846180646667 1 0.225047014688361 0.0430699644091567

ASXL1 1 0 2 4 0.981810335882373 1 0.453737297296426 0.0250155717660838

PPP1R16B 1 0 1 4 0.967502612277654 1 0.761468000160734 0.0124634702142331

CHD6 3 0 5 8 0.991663940284235 1 0.230225330251169 0.00355478680382881

PABPC1L 0 0 1 4 1 1 0.696413818386389 0.00483325080615788

TSHZ2 1 1 4 10 0.999913401968751 0.953951387898049 0.445703571585469 8.22347126647331e-05

ZNF217 1 1 3 5 0.996861809262073 0.850706445272823 0.354142517270538 0.0229854527630189

BCAS1 1 0 2 4 0.981810335882373 1 0.453737297296426 0.0250155717660838

SON 2 2 2 8 0.996375604609126 0.723938199009901 0.834082495747794 0.00114061542178986

BRWD1 1 0 2 5 0.989846180646667 1 0.529967391499114 0.00696117912301087

PTCHD1 1 0 1 3 0.942095563694729 1 0.696413818386389 0.0471593735603865

USP11 1 0 0 4 0.942095563694729 1 1 0.00483325080615788

WNK3 1 1 1 7 0.996861809262073 0.850706445272823 0.909940566010502 0.000437550920774896

MED12 0 1 3 6 1 0.850706445272823 0.354142517270538 0.00384176345468385

ATRX 3 1 17 10 0.99999715988815 0.997852589756841 1.14260488269764e-05 0.0417517572946482

NRK 0 3 1 1 1 0.0364632165930925 0.696413818386389 0.648204567890215

TENM1 3 1 3 7 0.978816625781951 0.931639069301714 0.595820079715031 0.0066296959072663

ZNF75D 0 0 2 3 1 1 0.284217063178254 0.0471593735603865

L1CAM 4 1 1 5 0.77754741972724 0.877052366695266 0.929572899371534 0.0361232787589351

CANX 1 0 4 1 0.967502612277654 1 0.0193357288484222 0.715213221625412

SLC29A4 1 1 1 4 0.981810335882373 0.733873138685818 0.812756450638119 0.0250155717660838

CCDC60 2 0 2 4 0.925559422532313 1 0.529967391499114 0.0430699644091567

FBXO22 0 0 5 0 1 1 0.000365012474729428 1

DNAH2 4 3 4 11 0.997533588970202 0.760701308008371 0.720839604254795 0.000530732144914017

PLCG1 0 1 4 0 1 0.610163927365809 0.00766526196931799 1

SPTA1 3 5 6 14 0.999984305778904 0.538711345282089 0.561949154483104 7.35777179413931e-05

ADAMTS19 1 1 0 3 0.942095563694729 0.610163927365809 1 0.0471593735603865

GABRA6 3 1 1 8 0.966795114671163 0.916803292852299 0.957056645958253 0.000573178831519092

ADGRF5 1 3 2 5 0.998262593363086 0.284103783719027 0.713617232400177 0.0361232787589351

FBXW10 2 1 1 4 0.925559422532313 0.780354200079733 0.853154887657977 0.0430699644091567

IPO9 0 0 2 4 1 1 0.371142909514663 0.0124634702142331

TERT 1 1 5 2 0.994347358298145 0.818848824808028 0.0225016097501159 0.52719599216433

ZNF366 0 0 3 4 1 1 0.164656940060257 0.0250155717660838

GABRB2 2 0 1 7 0.971717299622785 1 0.909940566010502 0.000437550920774896

ADCY1 1 2 1 5 0.994347358298145 0.47239720225338 0.884946097546456 0.0134136081088514

MUC17 4 2 5 10 0.99606128710228 0.903620452934011 0.4645842631583 0.00164052056751992

ZFHX3 3 1 4 10 0.996845067244519 0.969077046590886 0.548573715357109 0.000327566473728032

ITGB2 0 0 2 3 1 1 0.284217063178254 0.0471593735603865

FGG 0 2 0 5 1 0.341281033358699 1 0.00304939405034507

SDK1 3 4 2 11 0.998845640294677 0.454326063858094 0.94985579745827 0.000173165849983171

OR8K3 2 4 3 0 0.953836759434847 0.0490500553666507 0.288965031820512 1

DSG4 1 2 4 7 0.999710122222941 0.723938199009901 0.33743772632839 0.0066296959072663

USH2A 3 2 3 8 0.991663940284235 0.792682428777051 0.693227294964357 0.00355478680382881

APOB 2 2 7 13 0.999986507601746 0.940832991511349 0.220165132163987 4.6952534698628e-05

GPR83 1 1 4 0 0.967502612277654 0.677788552210515 0.0193357288484222 1

CEP126 0 1 4 0 1 0.610163927365809 0.00766526196931799 1

SSTR4 1 1 0 3 0.942095563694729 0.610163927365809 1 0.0471593735603865

LRP2 3 4 7 13 0.999972559289176 0.711547086013052 0.332307118301899 0.000237072468513051

STXBP5L 1 0 1 4 0.967502612277654 1 0.761468000160734 0.0124634702142331

KDR 1 1 2 4 0.989846180646667 0.780354200079733 0.529967391499114 0.0430699644091567

CARD6 0 1 3 6 1 0.850706445272823 0.354142517270538 0.00384176345468385

TSC22D1 0 1 1 5 1 0.733873138685818 0.812756450638119 0.00304939405034507

LRFN5 2 0 1 4 0.88165627064934 1 0.812756450638119 0.0250155717660838

MYH9 0 1 3 4 1 0.780354200079733 0.225047014688361 0.0430699644091567

CELSR1 1 0 2 4 0.981810335882373 1 0.453737297296426 0.0250155717660838

HYOU1 2 0 2 4 0.925559422532313 1 0.529967391499114 0.0430699644091567

ELMO2 1 0 1 3 0.942095563694729 1 0.696413818386389 0.0471593735603865

VPS13D 1 1 3 5 0.996861809262073 0.850706445272823 0.354142517270538 0.0229854527630189

AXDND1 0 0 3 6 1 1 0.288965031820512 0.00179923593433847

COL3A1 1 0 1 4 0.967502612277654 1 0.761468000160734 0.0124634702142331

PRR16 0 1 1 3 1 0.610163927365809 0.696413818386389 0.0471593735603865

DSP 2 2 4 9 0.999281817740843 0.821175177738358 0.498182099510322 0.00111272204598869

FAT3 0 1 4 1 1 0.677788552210515 0.0193357288484222 0.715213221625412

SSH1 1 0 1 4 0.967502612277654 1 0.761468000160734 0.0124634702142331

ZSCAN29 0 0 0 5 1 1 1 0.000198365463236156

WDR90 0 0 2 3 1 1 0.284217063178254 0.0471593735603865

MED13 1 0 3 5 0.994347358298145 1 0.288965031820512 0.0134136081088514

SIGLEC8 0 0 2 3 1 1 0.284217063178254 0.0471593735603865

NLRP9 0 0 2 3 1 1 0.284217063178254 0.0471593735603865

PCDH11X 2 2 2 6 0.989702246079122 0.637560622653752 0.760107688484289 0.0123383396121618

HSPG2 3 1 4 10 0.996845067244519 0.969077046590886 0.548573715357109 0.000327566473728032

GRHL3 1 1 3 5 0.996861809262073 0.850706445272823 0.354142517270538 0.0229854527630189

AMY2B 0 1 1 4 1 0.677788552210515 0.761468000160734 0.0124634702142331

SARS 2 0 1 4 0.88165627064934 1 0.812756450638119 0.0250155717660838

FAM46C 0 0 2 4 1 1 0.371142909514663 0.0124634702142331

OR2T1 0 1 1 3 1 0.610163927365809 0.696413818386389 0.0471593735603865

CAD 4 0 1 7 0.840571425817871 1 0.944978827634011 0.00210178352367709

CFLAR 1 1 1 4 0.981810335882373 0.733873138685818 0.812756450638119 0.0250155717660838

PTPRG 1 1 0 3 0.942095563694729 0.610163927365809 1 0.0471593735603865

PRICKLE2 2 0 1 5 0.925559422532313 1 0.853154887657977 0.00696117912301087

IGSF11 0 0 2 3 1 1 0.284217063178254 0.0471593735603865

NOA1 0 0 1 4 1 1 0.696413818386389 0.00483325080615788

PLCXD3 0 0 2 4 1 1 0.371142909514663 0.0124634702142331

THBS4 0 1 0 4 1 0.610163927365809 1 0.00483325080615788

MCC 0 0 4 3 1 1 0.0379766110990049 0.124485055761882

MEGF10 0 0 4 3 1 1 0.0379766110990049 0.124485055761882

UHRF1BP1 1 1 4 5 0.998262593363086 0.877052366695266 0.181989758700111 0.0361232787589351

TMEM181 1 0 1 4 0.967502612277654 1 0.761468000160734 0.0124634702142331

BAZ1B 1 0 5 2 0.989846180646667 1 0.0119464065179257 0.460397691935718

ANK1 2 3 4 7 0.9987602514107 0.534238584031864 0.445703571585469 0.01609182673977

TRPS1 0 1 2 4 1 0.733873138685818 0.453737297296426 0.0250155717660838

KANK1 2 0 4 10 0.9987602514107 1 0.445703571585469 8.22347126647331e-05

TAF1L 4 2 4 12 0.997533588970202 0.917912367426575 0.720839604254795 9.06436325013316e-05

UBAP1 1 0 2 5 0.989846180646667 1 0.529967391499114 0.00696117912301087

DNM1 1 1 3 7 0.999040806752537 0.898824343426312 0.481164951979619 0.00210178352367709

FAM208B 1 1 4 5 0.998262593363086 0.877052366695266 0.181989758700111 0.0361232787589351

TET1 2 2 2 9 0.997873212519892 0.760376948183903 0.862892634042714 0.0003148458575274

C10orf2 0 0 2 6 1 1 0.529967391499114 0.000702323006745593

PTPN11 0 1 2 7 1 0.850706445272823 0.659995881370045 0.000437550920774896

RBM19 2 0 1 4 0.88165627064934 1 0.812756450638119 0.0250155717660838

NALCN 2 0 3 5 0.971717299622785 1 0.354142517270538 0.0229854527630189

MYO1E 1 0 2 5 0.989846180646667 1 0.529967391499114 0.00696117912301087

AKAP13 2 0 7 6 0.997873212519892 1 0.020800866472003 0.0417614160086095

CACNG3 1 0 1 4 0.967502612277654 1 0.761468000160734 0.0124634702142331

SMCR8 0 0 2 5 1 1 0.453737297296426 0.00304939405034507

TOB1 1 1 2 6 0.996861809262073 0.850706445272823 0.659995881370045 0.00384176345468385

MC5R 0 0 1 4 1 1 0.696413818386389 0.00483325080615788

SIPA1L3 2 0 2 4 0.925559422532313 1 0.529967391499114 0.0430699644091567

MX1 2 0 1 5 0.925559422532313 1 0.853154887657977 0.00696117912301087

PPIL2 0 0 2 3 1 1 0.284217063178254 0.0471593735603865

PKDREJ 4 2 4 9 0.990237224261604 0.868054729047943 0.596347119134989 0.00307477273225714

HCCS 0 1 1 3 1 0.610163927365809 0.696413818386389 0.0471593735603865

PADI2 2 0 0 3 0.71710543467726 1 1 0.0471593735603865

SERINC2 1 1 0 3 0.942095563694729 0.610163927365809 1 0.0471593735603865

ZNF644 1 0 3 4 0.989846180646667 1 0.225047014688361 0.0430699644091567

INTS3 0 0 2 3 1 1 0.284217063178254 0.0471593735603865

ASH1L 2 2 2 6 0.989702246079122 0.637560622653752 0.760107688484289 0.0123383396121618

SMG5 3 0 1 4 0.749946815000421 1 0.853154887657977 0.0430699644091567

LAMC1 1 0 2 4 0.981810335882373 1 0.453737297296426 0.0250155717660838

HMCN1 5 3 5 9 0.989435928696628 0.760701308008371 0.51086711558083 0.0102575766093849

KIF21B 0 1 1 3 1 0.610163927365809 0.696413818386389 0.0471593735603865

RNPEP 1 0 1 3 0.942095563694729 1 0.696413818386389 0.0471593735603865

URB2 1 2 5 2 0.996861809262073 0.532130240624871 0.0377011024044698 0.588350987718456

SLC8A1 0 0 2 3 1 1 0.284217063178254 0.0471593735603865

PROKR1 0 0 1 6 1 1 0.812756450638119 0.00020563992283035

MAP3K19 1 2 3 6 0.999040806752537 0.637560622653752 0.481164951979619 0.0123383396121618

TANC1 1 0 4 2 0.981810335882373 1 0.0379766110990049 0.388631796595168

pk 0 1 1 3 1 0.610163927365809 0.696413818386389 0.0471593735603865

BTD 1 0 1 5 0.981810335882373 1 0.812756450638119 0.00304939405034507

PLCL2 0 0 4 3 1 1 0.0379766110990049 0.124485055761882

CELSR3 1 0 3 8 0.999040806752537 1 0.481164951979619 0.000258466100780682

QARS 1 0 1 4 0.967502612277654 1 0.761468000160734 0.0124634702142331

CACNA1D 1 1 3 5 0.996861809262073 0.850706445272823 0.354142517270538 0.0229854527630189

IMPG2 1 1 1 5 0.989846180646667 0.780354200079733 0.853154887657977 0.00696117912301087

ZIC4 0 1 1 5 1 0.733873138685818 0.812756450638119 0.00304939405034507

UGT2B15 1 1 0 4 0.967502612277654 0.677788552210515 1 0.0124634702142331

WDFY3 1 2 2 7 0.999040806752537 0.637560622653752 0.760107688484289 0.00210178352367709

SORBS2 1 1 1 5 0.989846180646667 0.780354200079733 0.853154887657977 0.00696117912301087

HTR1A 0 0 2 3 1 1 0.284217063178254 0.0471593735603865

MAP1B 1 1 6 3 0.998262593363086 0.877052366695266 0.0134029515175788 0.339710434859985

FER 0 0 1 4 1 1 0.696413818386389 0.00483325080615788

HSP90AB1 1 1 0 3 0.942095563694729 0.610163927365809 1 0.0471593735603865

PKHD1 3 4 8 13 0.999984305778904 0.740353042801546 0.213539818313764 0.000378767919008489

NCOA7 1 0 3 4 0.989846180646667 1 0.225047014688361 0.0430699644091567

GRM1 1 1 0 3 0.942095563694729 0.610163927365809 1 0.0471593735603865

INHBA 0 0 3 4 1 1 0.164656940060257 0.0250155717660838

AEBP1 1 0 1 3 0.942095563694729 1 0.696413818386389 0.0471593735603865

MLXIPL 1 0 1 3 0.942095563694729 1 0.696413818386389 0.0471593735603865

FOXP2 0 1 5 1 1 0.733873138685818 0.00535506716048509 0.769643459130453

KMT2C 5 4 5 9 0.993090107520443 0.57530198402541 0.55559049764496 0.0143635386974884

KIAA1429 0 0 1 4 1 1 0.696413818386389 0.00483325080615788

VPS13B 1 1 2 4 0.989846180646667 0.780354200079733 0.529967391499114 0.0430699644091567

SYBU 1 0 2 4 0.981810335882373 1 0.453737297296426 0.0250155717660838

VLDLR 1 0 4 2 0.981810335882373 1 0.0379766110990049 0.388631796595168

ADAMTSL1 0 0 1 4 1 1 0.696413818386389 0.00483325080615788

NPR2 1 1 1 5 0.989846180646667 0.780354200079733 0.853154887657977 0.00696117912301087

TRPM6 0 0 4 5 1 1 0.0972109804240587 0.0134136081088514

CCDC180 0 0 2 3 1 1 0.284217063178254 0.0471593735603865

TBC1D2 2 1 1 4 0.925559422532313 0.780354200079733 0.853154887657977 0.0430699644091567

EPC1 0 0 0 5 1 1 1 0.000198365463236156

ZNF33B 0 0 2 4 1 1 0.371142909514663 0.0124634702142331

CFAP70 0 0 2 3 1 1 0.284217063178254 0.0471593735603865

PDCD11 0 1 3 5 1 0.818848824808028 0.288965031820512 0.0134136081088514

TDRD1 0 1 1 4 1 0.677788552210515 0.761468000160734 0.0124634702142331

KCNK18 0 1 2 5 1 0.780354200079733 0.529967391499114 0.00696117912301087

CFAP46 0 0 3 4 1 1 0.164656940060257 0.0250155717660838

DDB1 0 0 3 4 1 1 0.164656940060257 0.0250155717660838

NRXN2 1 0 3 4 0.989846180646667 1 0.225047014688361 0.0430699644091567

OR6T1 0 0 2 3 1 1 0.284217063178254 0.0471593735603865

VWF 1 2 7 4 0.999710122222941 0.723938199009901 0.0133172950819115 0.255628199120549

DDX11 0 0 3 4 1 1 0.164656940060257 0.0250155717660838

KRT85 1 0 0 4 0.942095563694729 1 1 0.00483325080615788

ESPL1 3 2 5 7 0.994848503934627 0.821175177738358 0.275148355829698 0.0233482477344949

AMHR2 0 1 1 3 1 0.610163927365809 0.696413818386389 0.0471593735603865

ERBB3 1 0 4 5 0.996861809262073 1 0.136884232437118 0.0229854527630189

LRP1 2 1 4 8 0.997873212519892 0.943872077952986 0.391834011251894 0.00208435206666125

NOS1 1 1 2 10 0.999710122222941 0.931639069301714 0.834082495747794 1.40868969875972e-05

GCN1L1 2 0 5 6 0.993867179062387 1 0.113774103150873 0.0195839258529907

EP400 3 4 6 9 0.99959013761111 0.536440990352126 0.307222564921697 0.0102575766093849

OR4K1 0 0 1 4 1 1 0.696413818386389 0.00483325080615788

SUPT16H 0 0 1 4 1 1 0.696413818386389 0.00483325080615788

SYNE2 3 1 6 10 0.998845640294677 0.979299543008922 0.225975275382017 0.00100726691094266

ZFYVE26 1 0 5 3 0.994347358298145 1 0.0225016097501159 0.226603641135575

ACOT4 0 0 2 3 1 1 0.284217063178254 0.0471593735603865

DYNC1H1 4 1 3 7 0.947518516153163 0.943872077952986 0.646816596830816 0.0106068717677957

NUTM1 0 0 3 4 1 1 0.164656940060257 0.0250155717660838

VPS18 1 1 0 5 0.981810335882373 0.733873138685818 1 0.00304939405034507

CAPN3 1 1 0 4 0.967502612277654 0.677788552210515 1 0.0124634702142331

MYO5C 2 0 2 4 0.925559422532313 1 0.529967391499114 0.0430699644091567

MYH11 1 2 4 8 0.999841330058873 0.760376948183903 0.391834011251894 0.00208435206666125

TMC5 0 0 4 3 1 1 0.0379766110990049 0.124485055761882

EARS2 1 2 1 4 0.989846180646667 0.408505708928411 0.853154887657977 0.0430699644091567

ZNF646 2 0 5 3 0.971717299622785 1 0.0377011024044698 0.282576009947826

ITGAX 1 1 2 5 0.994347358298145 0.818848824808028 0.598825208550144 0.0134136081088514

NFATC3 0 0 2 3 1 1 0.284217063178254 0.0471593735603865

WRAP53 0 1 1 4 1 0.677788552210515 0.761468000160734 0.0124634702142331

MYH4 3 0 3 6 0.948612579116096 1 0.481164951979619 0.0123383396121618

NCOR1 1 0 4 5 0.996861809262073 1 0.136884232437118 0.0229854527630189

NOS2 0 0 2 4 1 1 0.371142909514663 0.0124634702142331

SPAG5 2 0 2 4 0.925559422532313 1 0.529967391499114 0.0430699644091567

SSH2 0 1 4 6 1 0.877052366695266 0.181989758700111 0.00722006156791945

MED1 0 0 2 5 1 1 0.453737297296426 0.00304939405034507

PSMD3 0 1 1 3 1 0.610163927365809 0.696413818386389 0.0471593735603865

STAT3 0 0 2 3 1 1 0.284217063178254 0.0471593735603865

C17orf53 0 1 1 3 1 0.610163927365809 0.696413818386389 0.0471593735603865

IGF2BP1 1 2 1 5 0.994347358298145 0.47239720225338 0.884946097546456 0.0134136081088514

GPR142 0 0 1 5 1 1 0.761468000160734 0.00101814110212027

DSG3 0 0 3 5 1 1 0.225047014688361 0.00696117912301087

PNPLA6 0 0 3 5 1 1 0.225047014688361 0.00696117912301087

FKBP8 1 0 1 3 0.942095563694729 1 0.696413818386389 0.0471593735603865

UPF1 0 1 3 5 1 0.818848824808028 0.288965031820512 0.0134136081088514

GPI 2 1 3 5 0.982853968251951 0.877052366695266 0.418699801734448 0.0361232787589351

TRPM4 0 0 2 4 1 1 0.371142909514663 0.0124634702142331

PCSK2 2 1 1 4 0.925559422532313 0.780354200079733 0.853154887657977 0.0430699644091567

RBL1 0 0 2 3 1 1 0.284217063178254 0.0471593735603865

PRAME 1 0 0 5 0.967502612277654 1 1 0.00101814110212027

ARSE 0 0 2 5 1 1 0.453737297296426 0.00304939405034507

MXRA5 5 1 3 7 0.895446837281322 0.953951387898049 0.693227294964357 0.01609182673977

SYTL5 1 0 2 4 0.981810335882373 1 0.453737297296426 0.0250155717660838

HUWE1 3 0 3 7 0.966795114671163 1 0.540452432560751 0.00388515566452808

CAPN6 1 1 1 4 0.981810335882373 0.733873138685818 0.812756450638119 0.0250155717660838

SAGE1 1 1 1 7 0.996861809262073 0.850706445272823 0.909940566010502 0.000437550920774896

MAP7D3 0 1 4 2 1 0.733873138685818 0.0379766110990049 0.388631796595168

F9 3 0 0 5 0.749946815000421 1 1 0.00696117912301087

MAGEC3 1 1 1 4 0.981810335882373 0.733873138685818 0.812756450638119 0.0250155717660838

SLAMF6 0 1 2 5 1 0.780354200079733 0.529967391499114 0.00696117912301087

JMY 0 1 4 0 1 0.610163927365809 0.00766526196931799 1

ABCC9 1 6 3 4 0.999710122222941 0.0185690003074586 0.595820079715031 0.255628199120549

SPAG17 1 2 2 6 0.998262593363086 0.587247231048392 0.713617232400177 0.00722006156791945

SCN10A 0 3 5 1 1 0.184885482291017 0.0225016097501159 0.849649605086086

EPHA3 0 1 6 0 1 0.733873138685818 0.000420013077478238 1

PCDHA3 1 0 6 3 0.996861809262073 1 0.00730211709576205 0.282576009947826

ADAMTS2 0 0 3 4 1 1 0.164656940060257 0.0250155717660838

COL11A2 1 0 6 2 0.994347358298145 1 0.00350224393217359 0.52719599216433

DYNC1I1 1 0 1 3 0.942095563694729 1 0.696413818386389 0.0471593735603865

SLC12A9 0 0 5 1 1 1 0.00183002852976591 0.715213221625412

ARHGEF5 1 0 3 4 0.989846180646667 1 0.225047014688361 0.0430699644091567

ANO4 0 1 4 2 1 0.733873138685818 0.0379766110990049 0.388631796595168

OR4M1 0 0 2 3 1 1 0.284217063178254 0.0471593735603865

PTPRS 0 0 2 5 1 1 0.453737297296426 0.00304939405034507

NEXN 1 0 4 2 0.981810335882373 1 0.0379766110990049 0.388631796595168

ABCC5 1 0 5 2 0.989846180646667 1 0.0119464065179257 0.460397691935718

CNTNAP2 3 2 4 6 0.986641153189149 0.760376948183903 0.391834011251894 0.0417614160086095

PDP1 1 0 2 4 0.981810335882373 1 0.453737297296426 0.0250155717660838

NOTCH2 1 2 3 6 0.999040806752537 0.637560622653752 0.481164951979619 0.0123383396121618

SLC5A7 0 3 1 5 1 0.184885482291017 0.884946097546456 0.0134136081088514

SPHKAP 2 3 4 7 0.9987602514107 0.534238584031864 0.445703571585469 0.01609182673977

STAB1 2 1 3 6 0.989702246079122 0.898824343426312 0.481164951979619 0.0123383396121618

MMRN1 0 1 3 4 1 0.780354200079733 0.225047014688361 0.0430699644091567

RAPGEF6 1 1 4 0 0.967502612277654 0.677788552210515 0.0193357288484222 1

ZAN 0 2 2 4 1 0.408505708928411 0.529967391499114 0.0430699644091567

DCSTAMP 0 1 1 5 1 0.733873138685818 0.812756450638119 0.00304939405034507

TRHDE 1 4 1 2 0.989846180646667 0.0311622625206435 0.853154887657977 0.460397691935718

EPHA10 0 4 2 3 1 0.0490500553666507 0.598825208550144 0.226603641135575

MACF1 1 4 5 7 0.999952875982995 0.325413522200063 0.275148355829698 0.0233482477344949

MAP1A 0 1 2 4 1 0.733873138685818 0.453737297296426 0.0250155717660838

TEKT5 0 1 1 3 1 0.610163927365809 0.696413818386389 0.0471593735603865

DCAF12L2 0 1 1 4 1 0.677788552210515 0.761468000160734 0.0124634702142331

MCOLN3 1 1 4 1 0.981810335882373 0.733873138685818 0.0379766110990049 0.769643459130453

PPP1R3A 2 0 5 3 0.971717299622785 1 0.0377011024044698 0.282576009947826

PEX14 1 0 1 3 0.942095563694729 1 0.696413818386389 0.0471593735603865

PTPRF 1 0 2 5 0.989846180646667 1 0.529967391499114 0.00696117912301087

TMPRSS7 0 0 1 4 1 1 0.696413818386389 0.00483325080615788

FBN2 2 2 3 7 0.996375604609126 0.723938199009901 0.595820079715031 0.0066296959072663

DNAH8 3 2 5 9 0.998084037803816 0.868054729047943 0.369423451190061 0.00307477273225714

FBP1 0 0 1 4 1 1 0.696413818386389 0.00483325080615788

GLI1 1 1 1 5 0.989846180646667 0.780354200079733 0.853154887657977 0.00696117912301087

PUM1 0 3 2 5 1 0.233465048767417 0.659995881370045 0.0229854527630189

WDR63 1 0 1 5 0.981810335882373 1 0.812756450638119 0.00304939405034507

HIPK1 0 0 4 2 1 1 0.0193357288484222 0.313161299214229

PDE4DIP 1 0 2 5 0.989846180646667 1 0.529967391499114 0.00696117912301087

ARHGEF11 0 1 2 5 1 0.780354200079733 0.529967391499114 0.00696117912301087

RXRG 1 0 0 5 0.967502612277654 1 1 0.00101814110212027

NAV1 2 0 4 5 0.982853968251951 1 0.181989758700111 0.0361232787589351

PIGR 0 1 1 3 1 0.610163927365809 0.696413818386389 0.0471593735603865

GALNT2 2 0 5 3 0.971717299622785 1 0.0377011024044698 0.282576009947826

CHRM3 1 1 1 5 0.989846180646667 0.780354200079733 0.853154887657977 0.00696117912301087

RGPD3 1 0 1 4 0.967502612277654 1 0.761468000160734 0.0124634702142331

TRIP12 0 1 1 3 1 0.610163927365809 0.696413818386389 0.0471593735603865

TRPM8 1 0 1 5 0.981810335882373 1 0.812756450638119 0.00304939405034507

FOXP1 1 0 1 3 0.942095563694729 1 0.696413818386389 0.0471593735603865

MUC4 0 0 3 4 1 1 0.164656940060257 0.0250155717660838

KIAA0232 0 0 2 3 1 1 0.284217063178254 0.0471593735603865

PRKG2 1 1 2 6 0.996861809262073 0.850706445272823 0.659995881370045 0.00384176345468385

RASGRF2 2 2 2 5 0.982853968251951 0.587247231048392 0.713617232400177 0.0361232787589351

FSTL4 1 0 1 4 0.967502612277654 1 0.761468000160734 0.0124634702142331

PCDHGA1 0 1 1 4 1 0.677788552210515 0.761468000160734 0.0124634702142331

LARP1 0 0 3 4 1 1 0.164656940060257 0.0250155717660838

FBXW11 1 0 4 1 0.967502612277654 1 0.0193357288484222 0.715213221625412

NOTCH4 0 0 2 3 1 1 0.284217063178254 0.0471593735603865

TRERF1 1 0 1 4 0.967502612277654 1 0.761468000160734 0.0124634702142331

CNR1 1 0 2 5 0.989846180646667 1 0.529967391499114 0.00696117912301087

THEMIS 1 0 1 4 0.967502612277654 1 0.761468000160734 0.0124634702142331

T 0 0 1 4 1 1 0.696413818386389 0.00483325080615788

IQCE 0 0 2 3 1 1 0.284217063178254 0.0471593735603865

BMPER 0 0 4 3 1 1 0.0379766110990049 0.124485055761882

HECW1 0 1 2 5 1 0.780354200079733 0.529967391499114 0.00696117912301087

ABCB4 0 2 3 5 1 0.532130240624871 0.354142517270538 0.0229854527630189

PPP1R9A 0 0 2 4 1 1 0.371142909514663 0.0124634702142331

CFTR 0 0 3 5 1 1 0.225047014688361 0.00696117912301087

FLNC 2 0 6 2 0.971717299622785 1 0.00730211709576205 0.588350987718456

TBXAS1 0 1 0 4 1 0.610163927365809 1 0.00483325080615788

PTPRN2 0 0 0 6 1 1 1 3.44103354593332e-05

NSMAF 1 0 1 3 0.942095563694729 1 0.696413818386389 0.0471593735603865

WISP1 0 0 1 4 1 1 0.696413818386389 0.00483325080615788

CYP11B2 0 0 2 3 1 1 0.284217063178254 0.0471593735603865

EPPK1 0 1 2 4 1 0.733873138685818 0.453737297296426 0.0250155717660838

PTCH1 1 1 3 5 0.996861809262073 0.850706445272823 0.354142517270538 0.0229854527630189

PTPN3 3 0 2 5 0.882314598683094 1 0.659995881370045 0.0229854527630189

TNC 1 0 4 5 0.996861809262073 1 0.136884232437118 0.0229854527630189

BTAF1 1 0 3 4 0.989846180646667 1 0.225047014688361 0.0430699644091567

DOCK1 1 0 2 4 0.981810335882373 1 0.453737297296426 0.0250155717660838

LRP5 1 0 3 5 0.994347358298145 1 0.288965031820512 0.0134136081088514

LRRC32 1 0 1 3 0.942095563694729 1 0.696413818386389 0.0471593735603865

KIAA1551 2 1 2 5 0.971717299622785 0.850706445272823 0.659995881370045 0.0229854527630189

COL2A1 1 2 1 4 0.989846180646667 0.408505708928411 0.853154887657977 0.0430699644091567

ACACB 0 1 1 7 1 0.818848824808028 0.884946097546456 0.00015386654294915

CUX2 1 1 2 6 0.996861809262073 0.850706445272823 0.659995881370045 0.00384176345468385

DNAH10 0 0 2 6 1 1 0.529967391499114 0.000702323006745593

TRPC4 0 0 1 4 1 1 0.696413818386389 0.00483325080615788

ATP11A 0 0 4 3 1 1 0.0379766110990049 0.124485055761882

MMP14 0 0 1 4 1 1 0.696413818386389 0.00483325080615788

LRRC16B 0 0 1 4 1 1 0.696413818386389 0.00483325080615788

VIPAS39 1 0 1 3 0.942095563694729 1 0.696413818386389 0.0471593735603865

TP53BP1 1 0 2 4 0.981810335882373 1 0.453737297296426 0.0250155717660838

DMXL2 0 1 2 4 1 0.733873138685818 0.453737297296426 0.0250155717660838

BNC1 0 1 2 6 1 0.818848824808028 0.598825208550144 0.00179923593433847

ACAN 1 2 5 1 0.994347358298145 0.47239720225338 0.0225016097501159 0.849649605086086

ZNF263 0 0 1 4 1 1 0.696413818386389 0.00483325080615788

DHX38 2 0 3 5 0.971717299622785 1 0.354142517270538 0.0229854527630189

PKD1L2 1 0 4 5 0.996861809262073 1 0.136884232437118 0.0229854527630189

SYNRG 0 0 4 2 1 1 0.0193357288484222 0.313161299214229

GRB7 1 0 0 4 0.942095563694729 1 1 0.00483325080615788

DCC 2 1 6 4 0.993867179062387 0.916803292852299 0.0346989276250299 0.210859904455178

MAN2B1 1 1 1 6 0.994347358298145 0.818848824808028 0.884946097546456 0.00179923593433847

SLC1A6 0 1 2 4 1 0.733873138685818 0.453737297296426 0.0250155717660838

NWD1 3 2 4 7 0.991663940284235 0.792682428777051 0.445703571585469 0.01609182673977

RYR1 2 0 6 5 0.993867179062387 1 0.0346989276250299 0.074067892977655

SPTBN4 0 0 2 4 1 1 0.371142909514663 0.0124634702142331

LILRB3 0 0 2 3 1 1 0.284217063178254 0.0471593735603865

SIRPB1 1 0 2 4 0.981810335882373 1 0.453737297296426 0.0250155717660838

BPI 0 0 1 4 1 1 0.696413818386389 0.00483325080615788

CDH4 1 0 1 3 0.942095563694729 1 0.696413818386389 0.0471593735603865

PRPF6 0 0 2 3 1 1 0.284217063178254 0.0471593735603865

PCNT 1 0 1 6 0.989846180646667 1 0.853154887657977 0.000702323006745593

CCT8L2 0 2 1 4 1 0.341281033358699 0.812756450638119 0.0250155717660838

KDM6A 1 0 1 4 0.967502612277654 1 0.761468000160734 0.0124634702142331

DRP2 1 0 1 3 0.942095563694729 1 0.696413818386389 0.0471593735603865

CRNN 0 1 2 4 1 0.733873138685818 0.453737297296426 0.0250155717660838

CDH18 0 4 1 5 1 0.0715331605127528 0.909940566010502 0.0229854527630189

PCDHA7 0 2 2 4 1 0.408505708928411 0.529967391499114 0.0430699644091567

EEF1A1 1 2 0 4 0.981810335882373 0.341281033358699 1 0.0250155717660838

ZNF71 0 0 1 4 1 1 0.696413818386389 0.00483325080615788

CNTRL 0 0 5 1 1 1 0.00183002852976591 0.715213221625412

PLXNC1 1 0 4 1 0.967502612277654 1 0.0193357288484222 0.715213221625412

CYP4F11 0 0 3 4 1 1 0.164656940060257 0.0250155717660838

NPAP1 4 0 3 7 0.922716240598273 1 0.595820079715031 0.0066296959072663

DNAH17 0 0 4 4 1 1 0.0640068156800842 0.0430699644091567

NLRP4 1 0 2 7 0.996861809262073 1 0.659995881370045 0.000437550920774896

YY2 1 0 1 3 0.942095563694729 1 0.696413818386389 0.0471593735603865

SPEN 3 0 8 5 0.991663940284235 1 0.00792671448301265 0.159512077371233

JAK1 2 0 2 8 0.989702246079122 1 0.760107688484289 0.000258466100780682

FLG2 3 2 8 12 0.999917560141795 0.949920188749583 0.127995469138109 0.000455950095276326

C2orf16 1 0 3 5 0.994347358298145 1 0.288965031820512 0.0134136081088514

ADD2 1 1 1 5 0.989846180646667 0.780354200079733 0.853154887657977 0.00696117912301087

SCN3A 0 0 4 3 1 1 0.0379766110990049 0.124485055761882

FAM171B 0 0 2 3 1 1 0.284217063178254 0.0471593735603865

VIL1 0 0 1 5 1 1 0.761468000160734 0.00101814110212027

FNDC3B 3 0 1 4 0.749946815000421 1 0.853154887657977 0.0430699644091567

UGT2B28 0 0 2 3 1 1 0.284217063178254 0.0471593735603865

KIAA0922 0 1 2 4 1 0.733873138685818 0.453737297296426 0.0250155717660838

PLK2 1 0 0 5 0.967502612277654 1 1 0.00101814110212027

STK10 0 0 4 3 1 1 0.0379766110990049 0.124485055761882

DDR1 0 0 2 3 1 1 0.284217063178254 0.0471593735603865

MTMR7 1 0 1 3 0.942095563694729 1 0.696413818386389 0.0471593735603865

FAM135B 2 0 2 6 0.971717299622785 1 0.659995881370045 0.00384176345468385

FRMPD1 3 0 3 5 0.92160229079154 1 0.418699801734448 0.0361232787589351

RRP12 0 0 1 4 1 1 0.696413818386389 0.00483325080615788

BTBD16 0 0 4 2 1 1 0.0193357288484222 0.313161299214229

OR5P2 0 0 2 3 1 1 0.284217063178254 0.0471593735603865

NAV2 3 0 4 7 0.978816625781951 1 0.33743772632839 0.0066296959072663

KAT5 2 0 2 5 0.953836759434847 1 0.598825208550144 0.0134136081088514

SCN2B 1 0 1 4 0.967502612277654 1 0.761468000160734 0.0124634702142331

KMT2A 1 1 4 7 0.999471942393383 0.916803292852299 0.28354001670918 0.00388515566452808

CD163L1 1 2 5 6 0.999710122222941 0.723938199009901 0.148795742661153 0.029299598312284

RFX4 2 1 0 4 0.88165627064934 0.733873138685818 1 0.0250155717660838

FREM2 2 0 4 9 0.997873212519892 1 0.391834011251894 0.0003148458575274

DUOX1 0 1 1 4 1 0.677788552210515 0.761468000160734 0.0124634702142331

UBN1 0 1 1 6 1 0.780354200079733 0.853154887657977 0.000702323006745593

ABCC1 1 0 4 5 0.996861809262073 1 0.136884232437118 0.0229854527630189

OR7G2 0 1 1 4 1 0.677788552210515 0.761468000160734 0.0124634702142331

SIGLEC1 0 1 0 4 1 0.610163927365809 1 0.00483325080615788

ZHX3 0 0 4 8 1 1 0.231304350998932 0.000258466100780682

ASCC2 0 0 2 4 1 1 0.371142909514663 0.0124634702142331

TLR8 0 2 1 4 1 0.341281033358699 0.812756450638119 0.0250155717660838

ZFX 0 0 2 3 1 1 0.284217063178254 0.0471593735603865

DOCK11 1 2 2 5 0.996861809262073 0.532130240624871 0.659995881370045 0.0229854527630189

MROH2B 2 1 5 2 0.971717299622785 0.850706445272823 0.0377011024044698 0.588350987718456

TDRD6 1 1 8 4 0.999710122222941 0.931639069301714 0.00267316359756436 0.255628199120549

PXDNL 0 1 4 0 1 0.610163927365809 0.00766526196931799 1

PKHD1L1 0 1 4 0 1 0.610163927365809 0.00766526196931799 1

UNC79 4 2 7 4 0.976802643249069 0.821175177738358 0.0436649964708246 0.397561865421127

ATP1A3 0 0 4 2 1 1 0.0193357288484222 0.313161299214229

KIF16B 1 1 5 1 0.989846180646667 0.780354200079733 0.0119464065179257 0.813821425872558

FRMD7 1 1 3 5 0.996861809262073 0.850706445272823 0.354142517270538 0.0229854527630189

UGT2A3 0 1 3 4 1 0.780354200079733 0.225047014688361 0.0430699644091567

GPR107 1 0 1 5 0.981810335882373 1 0.812756450638119 0.00304939405034507

OR10A3 1 0 1 3 0.942095563694729 1 0.696413818386389 0.0471593735603865

HOOK1 0 0 5 1 1 1 0.00183002852976591 0.715213221625412

NFASC 0 1 5 1 1 0.733873138685818 0.00535506716048509 0.769643459130453

NID1 0 0 4 2 1 1 0.0193357288484222 0.313161299214229

OR2L13 1 0 1 4 0.967502612277654 1 0.761468000160734 0.0124634702142331

PLB1 0 1 4 0 1 0.610163927365809 0.00766526196931799 1

EPAS1 0 2 5 2 1 0.47239720225338 0.0225016097501159 0.52719599216433

RETSAT 1 0 4 0 0.942095563694729 1 0.00766526196931799 1

SLC9A2 2 0 4 1 0.88165627064934 1 0.0379766110990049 0.769643459130453

HECW2 0 0 3 4 1 1 0.164656940060257 0.0250155717660838

IDH1 1 0 16 1 0.999974432714178 1 3.07656199327008e-10 0.978851782128671

ACOX3 0 0 2 3 1 1 0.284217063178254 0.0471593735603865

PDGFRA 3 1 7 5 0.991663940284235 0.953951387898049 0.0308140957967732 0.159512077371233

DHX16 0 1 4 0 1 0.610163927365809 0.00766526196931799 1

SGK223 1 1 4 1 0.981810335882373 0.733873138685818 0.0379766110990049 0.769643459130453

FGFR1 0 1 4 0 1 0.610163927365809 0.00766526196931799 1

KAT6B 0 2 5 2 1 0.47239720225338 0.0225016097501159 0.52719599216433

CFAP43 0 0 5 1 1 1 0.00183002852976591 0.715213221625412

C10orf90 1 0 5 0 0.967502612277654 1 0.00183002852976591 1

ABCC8 1 0 2 5 0.989846180646667 1 0.529967391499114 0.00696117912301087

ANO3 0 0 4 3 1 1 0.0379766110990049 0.124485055761882

ZBTB16 1 0 4 0 0.942095563694729 1 0.00766526196931799 1

SORL1 0 2 5 3 1 0.532130240624871 0.0377011024044698 0.282576009947826

PANX3 0 1 4 1 1 0.677788552210515 0.0193357288484222 0.715213221625412

DNAJC14 1 0 4 0 0.942095563694729 1 0.00766526196931799 1

TSHR 1 0 4 0 0.942095563694729 1 0.00766526196931799 1

IQCH 0 1 4 2 1 0.733873138685818 0.0379766110990049 0.388631796595168

ACSM2B 1 0 6 3 0.996861809262073 1 0.00730211709576205 0.282576009947826

EDC4 3 0 5 0 0.749946815000421 1 0.0119464065179257 1

TBX4 0 1 4 1 1 0.677788552210515 0.0193357288484222 0.715213221625412

NACA2 0 0 4 1 1 1 0.00766526196931799 0.648204567890215

ACE 1 0 3 4 0.989846180646667 1 0.225047014688361 0.0430699644091567

ACSBG2 0 0 2 3 1 1 0.284217063178254 0.0471593735603865

CYP4F12 0 1 4 1 1 0.677788552210515 0.0193357288484222 0.715213221625412

TSHZ3 4 1 3 6 0.922716240598273 0.931639069301714 0.595820079715031 0.029299598312284

LILRA5 1 0 1 3 0.942095563694729 1 0.696413818386389 0.0471593735603865

CSF2RA 0 0 5 0 1 1 0.000365012474729428 1

FAM47B 2 1 5 2 0.971717299622785 0.850706445272823 0.0377011024044698 0.588350987718456

PCDH19 2 0 2 4 0.925559422532313 1 0.529967391499114 0.0430699644091567

MAGEC1 0 0 4 1 1 1 0.00766526196931799 0.648204567890215

TPO 1 1 2 5 0.994347358298145 0.818848824808028 0.598825208550144 0.0134136081088514

JARID2 1 0 1 3 0.942095563694729 1 0.696413818386389 0.0471593735603865

PCDHGB2 0 0 3 4 1 1 0.164656940060257 0.0250155717660838

ABI2 2 0 4 0 0.815060320780103 1 0.0193357288484222 1

ARHGEF28 0 0 6 1 1 1 0.000420013077478238 0.769643459130453

ARFGEF2 3 0 3 6 0.948612579116096 1 0.481164951979619 0.0123383396121618

EDC3 0 1 0 4 1 0.610163927365809 1 0.00483325080615788

ENPP7 0 0 2 3 1 1 0.284217063178254 0.0471593735603865

BSN 0 1 5 2 1 0.780354200079733 0.0119464065179257 0.460397691935718

ADAM28 0 1 1 3 1 0.610163927365809 0.696413818386389 0.0471593735603865

CLEC14A 0 2 0 3 1 0.202753277686633 1 0.0471593735603865

OR10G8 0 0 2 4 1 1 0.371142909514663 0.0124634702142331

SACS 2 3 7 5 0.999281817740843 0.578986811567249 0.0436649964708246 0.194472246113733

LRRC55 1 0 1 3 0.942095563694729 1 0.696413818386389 0.0471593735603865

AMPD1 0 1 2 4 1 0.733873138685818 0.453737297296426 0.0250155717660838

MSL3 1 0 2 4 0.981810335882373 1 0.453737297296426 0.0250155717660838

XK 0 2 0 3 1 0.202753277686633 1 0.0471593735603865

CXorf57 1 0 1 3 0.942095563694729 1 0.696413818386389 0.0471593735603865

TRIM71 0 0 2 3 1 1 0.284217063178254 0.0471593735603865

KIF4B 0 1 5 4 1 0.850706445272823 0.0377011024044698 0.0960014472403224

CDK13 2 0 2 4 0.925559422532313 1 0.529967391499114 0.0430699644091567

ADNP2 0 0 2 4 1 1 0.371142909514663 0.0124634702142331

STK36 0 1 4 2 1 0.733873138685818 0.0379766110990049 0.388631796595168

HTT 1 1 5 2 0.994347358298145 0.818848824808028 0.0225016097501159 0.52719599216433

DPYSL2 0 0 2 3 1 1 0.284217063178254 0.0471593735603865

PTPDC1 0 0 2 3 1 1 0.284217063178254 0.0471593735603865

KIDINS220 3 0 5 1 0.826588743373446 1 0.0225016097501159 0.849649605086086

GLI2 1 0 2 4 0.981810335882373 1 0.453737297296426 0.0250155717660838

PIK3R5 1 1 0 3 0.942095563694729 0.610163927365809 1 0.0471593735603865

TMEM214 1 0 4 0 0.942095563694729 1 0.00766526196931799 1

CNGA4 0 0 4 3 1 1 0.0379766110990049 0.124485055761882

CNOT1 1 0 5 3 0.994347358298145 1 0.0225016097501159 0.226603641135575

POLR2A 1 0 1 6 0.989846180646667 1 0.853154887657977 0.000702323006745593

CDH9 1 1 2 4 0.989846180646667 0.780354200079733 0.529967391499114 0.0430699644091567

ZNF208 1 0 3 5 0.994347358298145 1 0.288965031820512 0.0134136081088514

NLRP11 2 0 4 1 0.88165627064934 1 0.0379766110990049 0.769643459130453

PKD2 0 1 1 3 1 0.610163927365809 0.696413818386389 0.0471593735603865

ADAMTS16 0 2 2 4 1 0.408505708928411 0.529967391499114 0.0430699644091567

SEMA3E 0 4 1 2 1 0.0178288923408584 0.812756450638119 0.388631796595168

BMP3 1 1 0 3 0.942095563694729 0.610163927365809 1 0.0471593735603865

KIAA1598 0 1 1 4 1 0.677788552210515 0.761468000160734 0.0124634702142331

SYNJ2 1 1 4 1 0.981810335882373 0.733873138685818 0.0379766110990049 0.769643459130453

ZDBF2 1 2 5 2 0.996861809262073 0.532130240624871 0.0377011024044698 0.588350987718456

ANKRD26 1 1 4 0 0.967502612277654 0.677788552210515 0.0193357288484222 1

HHIPL2 1 0 4 2 0.981810335882373 1 0.0379766110990049 0.388631796595168

XIRP1 1 1 4 0 0.967502612277654 0.677788552210515 0.0193357288484222 1

DAXX 1 1 4 1 0.981810335882373 0.733873138685818 0.0379766110990049 0.769643459130453

RTTN 0 1 4 2 1 0.733873138685818 0.0379766110990049 0.388631796595168

CAST 1 0 4 0 0.942095563694729 1 0.00766526196931799 1

OR2H1 1 0 4 0 0.942095563694729 1 0.00766526196931799 1

ENPEP 0 0 3 5 1 1 0.225047014688361 0.00696117912301087

CD163 2 1 5 1 0.953836759434847 0.818848824808028 0.0225016097501159 0.849649605086086

SOCS6 0 0 4 1 1 1 0.00766526196931799 0.648204567890215

C3orf20 1 0 0 4 0.942095563694729 1 1 0.00483325080615788

MYO3A 1 0 2 4 0.981810335882373 1 0.453737297296426 0.0250155717660838

CCNB3 1 1 4 1 0.981810335882373 0.733873138685818 0.0379766110990049 0.769643459130453

TKTL1 0 0 2 3 1 1 0.284217063178254 0.0471593735603865

CYP4B1 0 1 1 4 1 0.677788552210515 0.761468000160734 0.0124634702142331

ZPBP 0 3 1 1 1 0.0364632165930925 0.696413818386389 0.648204567890215

TJP3 0 0 3 4 1 1 0.164656940060257 0.0250155717660838

KIAA1324 2 0 2 4 0.925559422532313 1 0.529967391499114 0.0430699644091567

THBS3 2 0 1 5 0.925559422532313 1 0.853154887657977 0.00696117912301087

CXCR2 1 0 4 2 0.981810335882373 1 0.0379766110990049 0.388631796595168

ZBTB20 1 0 1 3 0.942095563694729 1 0.696413818386389 0.0471593735603865

SLC7A14 1 0 4 1 0.967502612277654 1 0.0193357288484222 0.715213221625412

UBR5 2 1 2 5 0.971717299622785 0.850706445272823 0.659995881370045 0.0229854527630189

ABCA1 1 0 5 3 0.994347358298145 1 0.0225016097501159 0.226603641135575

GRID1 2 1 1 4 0.925559422532313 0.780354200079733 0.853154887657977 0.0430699644091567

MYF5 2 0 0 4 0.815060320780103 1 1 0.0124634702142331

TPTE2 1 2 4 0 0.981810335882373 0.341281033358699 0.0379766110990049 1

CATSPERB 2 0 0 3 0.71710543467726 1 1 0.0471593735603865

LARP6 1 1 1 4 0.981810335882373 0.733873138685818 0.812756450638119 0.0250155717660838

CHD2 2 0 4 1 0.88165627064934 1 0.0379766110990049 0.769643459130453

SMYD4 2 0 0 3 0.71710543467726 1 1 0.0471593735603865

DHX58 1 1 0 3 0.942095563694729 0.610163927365809 1 0.0471593735603865

ZNF226 1 0 4 1 0.967502612277654 1 0.0193357288484222 0.715213221625412

PTPRH 2 1 2 5 0.971717299622785 0.850706445272823 0.659995881370045 0.0229854527630189

GSPT2 1 0 1 3 0.942095563694729 1 0.696413818386389 0.0471593735603865

IQSEC2 1 0 1 3 0.942095563694729 1 0.696413818386389 0.0471593735603865

MGAT4C 1 1 4 1 0.981810335882373 0.733873138685818 0.0379766110990049 0.769643459130453

CFAP47 1 4 7 4 0.999913401968751 0.282759892549312 0.0308140957967732 0.349776072642917

SRPX2 0 1 1 3 1 0.610163927365809 0.696413818386389 0.0471593735603865

COL11A1 1 0 4 1 0.967502612277654 1 0.0193357288484222 0.715213221625412

ALS2 0 0 1 4 1 1 0.696413818386389 0.00483325080615788

AKAP9 0 1 2 4 1 0.733873138685818 0.453737297296426 0.0250155717660838

WDR81 1 0 1 3 0.942095563694729 1 0.696413818386389 0.0471593735603865

ITIH4 0 0 4 2 1 1 0.0193357288484222 0.313161299214229

NEDD4 0 0 4 3 1 1 0.0379766110990049 0.124485055761882

MORC1 0 0 4 2 1 1 0.0193357288484222 0.313161299214229

COL12A1 1 0 2 4 0.981810335882373 1 0.453737297296426 0.0250155717660838

SULT1B1 0 3 0 2 1 0.0364632165930925 1 0.236059291778332

ZBTB2 0 1 1 4 1 0.677788552210515 0.761468000160734 0.0124634702142331

NCOA2 0 0 2 5 1 1 0.453737297296426 0.00304939405034507

PHLDB2 1 1 1 5 0.989846180646667 0.780354200079733 0.853154887657977 0.00696117912301087

HIST1H2BN 1 1 0 3 0.942095563694729 0.610163927365809 1 0.0471593735603865

FANCA 2 0 4 1 0.88165627064934 1 0.0379766110990049 0.769643459130453

NLRP13 1 1 1 6 0.994347358298145 0.818848824808028 0.884946097546456 0.00179923593433847

POU2F1 1 1 0 3 0.942095563694729 0.610163927365809 1 0.0471593735603865

DDX26B 2 0 1 5 0.925559422532313 1 0.853154887657977 0.00696117912301087

MFSD9 1 3 1 0 0.942095563694729 0.0364632165930925 0.696413818386389 1

PHF19 1 0 1 3 0.942095563694729 1 0.696413818386389 0.0471593735603865

CCDC105 1 0 3 4 0.989846180646667 1 0.225047014688361 0.0430699644091567

PRAMEF19 0 0 3 4 1 1 0.164656940060257 0.0250155717660838

CNNM4 1 0 1 3 0.942095563694729 1 0.696413818386389 0.0471593735603865

TNS1 1 0 2 5 0.989846180646667 1 0.529967391499114 0.00696117912301087

CTNND2 2 1 1 5 0.953836759434847 0.818848824808028 0.884946097546456 0.0134136081088514

ANKHD1-EIF4EBP3 0 1 3 5 1 0.818848824808028 0.288965031820512 0.0134136081088514

MAP3K4 1 0 2 5 0.989846180646667 1 0.529967391499114 0.00696117912301087

FAM120B 1 0 2 4 0.981810335882373 1 0.453737297296426 0.0250155717660838

OXA1L 0 0 2 3 1 1 0.284217063178254 0.0471593735603865

ZFYVE1 0 0 3 4 1 1 0.164656940060257 0.0250155717660838

MLH3 0 0 4 5 1 1 0.0972109804240587 0.0134136081088514

EIF5 0 0 2 4 1 1 0.371142909514663 0.0124634702142331

ZFYVE19 0 1 1 4 1 0.677788552210515 0.761468000160734 0.0124634702142331

CYP19A1 1 0 1 3 0.942095563694729 1 0.696413818386389 0.0471593735603865

SCNN1G 0 1 0 4 1 0.610163927365809 1 0.00483325080615788

ABCC12 0 0 2 3 1 1 0.284217063178254 0.0471593735603865

MYH13 1 1 3 5 0.996861809262073 0.850706445272823 0.354142517270538 0.0229854527630189

ZNF556 0 0 1 4 1 1 0.696413818386389 0.00483325080615788

ZNF439 0 0 1 4 1 1 0.696413818386389 0.00483325080615788

CEACAM1 0 1 3 4 1 0.780354200079733 0.225047014688361 0.0430699644091567

CSE1L 0 0 3 4 1 1 0.164656940060257 0.0250155717660838

POLA1 0 0 2 3 1 1 0.284217063178254 0.0471593735603865

NSDHL 0 1 0 4 1 0.610163927365809 1 0.00483325080615788

DLC1 1 0 4 2 0.981810335882373 1 0.0379766110990049 0.388631796595168

TUBA3C 0 3 1 6 1 0.233465048767417 0.909940566010502 0.00384176345468385

BLM 0 1 4 1 1 0.677788552210515 0.0193357288484222 0.715213221625412

CEP104 1 0 4 2 0.981810335882373 1 0.0379766110990049 0.388631796595168

ROR1 1 0 4 0 0.942095563694729 1 0.00766526196931799 1

NCOA1 0 0 5 1 1 1 0.00183002852976591 0.715213221625412

MSH6 1 0 2 4 0.981810335882373 1 0.453737297296426 0.0250155717660838

DGKD 1 0 4 1 0.967502612277654 1 0.0193357288484222 0.715213221625412

LTF 0 1 4 2 1 0.733873138685818 0.0379766110990049 0.388631796595168

SLITRK3 1 0 4 2 0.981810335882373 1 0.0379766110990049 0.388631796595168

AGPAT9 0 0 2 3 1 1 0.284217063178254 0.0471593735603865

GPRC6A 0 1 4 1 1 0.677788552210515 0.0193357288484222 0.715213221625412

TLE4 1 0 4 0 0.942095563694729 1 0.00766526196931799 1

UPF2 0 1 5 1 1 0.733873138685818 0.00535506716048509 0.769643459130453

GUCY2C 1 0 4 1 0.967502612277654 1 0.0193357288484222 0.715213221625412

SLC4A8 0 0 2 3 1 1 0.284217063178254 0.0471593735603865

LEMD3 0 2 4 1 1 0.341281033358699 0.0379766110990049 0.769643459130453

MALT1 1 0 4 2 0.981810335882373 1 0.0379766110990049 0.388631796595168

PLAGL2 0 0 4 1 1 1 0.00766526196931799 0.648204567890215

SCAF4 2 0 4 1 0.88165627064934 1 0.0379766110990049 0.769643459130453

TRMU 0 3 2 0 1 0.0364632165930925 0.284217063178254 1

LNPEP 0 0 1 5 1 1 0.761468000160734 0.00101814110212027

SLTM 1 0 0 4 0.942095563694729 1 1 0.00483325080615788

OR4D5 2 0 0 3 0.71710543467726 1 1 0.0471593735603865

FAM83H 0 2 4 1 1 0.341281033358699 0.0379766110990049 0.769643459130453

MAP3K15 1 0 4 1 0.967502612277654 1 0.0193357288484222 0.715213221625412

SHROOM3 0 0 2 3 1 1 0.284217063178254 0.0471593735603865

CYLC2 0 1 1 3 1 0.610163927365809 0.696413818386389 0.0471593735603865

SGSM2 0 0 2 3 1 1 0.284217063178254 0.0471593735603865

SETDB1 0 0 2 3 1 1 0.284217063178254 0.0471593735603865

BRDT 1 0 1 3 0.942095563694729 1 0.696413818386389 0.0471593735603865

ABCG5 0 0 1 4 1 1 0.696413818386389 0.00483325080615788

SLC6A6 1 0 1 3 0.942095563694729 1 0.696413818386389 0.0471593735603865

ALDH1L1 0 0 2 3 1 1 0.284217063178254 0.0471593735603865

DNAJC13 0 0 1 4 1 1 0.696413818386389 0.00483325080615788

LRRC15 0 1 1 3 1 0.610163927365809 0.696413818386389 0.0471593735603865

UGT3A1 0 0 3 4 1 1 0.164656940060257 0.0250155717660838

PDE6A 0 0 4 4 1 1 0.0640068156800842 0.0430699644091567

LGSN 0 0 2 5 1 1 0.453737297296426 0.00304939405034507

CTTNBP2 0 0 3 5 1 1 0.225047014688361 0.00696117912301087

KIAA1033 0 0 2 4 1 1 0.371142909514663 0.0124634702142331

AKAP6 2 0 2 4 0.925559422532313 1 0.529967391499114 0.0430699644091567

SNX1 0 0 2 3 1 1 0.284217063178254 0.0471593735603865

ADCY9 1 2 3 5 0.998262593363086 0.587247231048392 0.418699801734448 0.0361232787589351

ABCC11 1 0 2 4 0.981810335882373 1 0.453737297296426 0.0250155717660838

COG4 0 0 2 3 1 1 0.284217063178254 0.0471593735603865

BZRAP1 0 0 2 4 1 1 0.371142909514663 0.0124634702142331

C17orf47 0 1 1 4 1 0.677788552210515 0.761468000160734 0.0124634702142331

PAK7 2 0 2 5 0.953836759434847 1 0.598825208550144 0.0134136081088514

SLX4IP 1 0 3 4 0.989846180646667 1 0.225047014688361 0.0430699644091567

ASPHD2 1 0 1 5 0.981810335882373 1 0.812756450638119 0.00304939405034507

BRD1 1 0 2 4 0.981810335882373 1 0.453737297296426 0.0250155717660838

USP9X 0 0 3 7 1 1 0.354142517270538 0.000437550920774896

KCNK10 0 0 1 4 1 1 0.696413818386389 0.00483325080615788

BDP1 1 0 4 1 0.967502612277654 1 0.0193357288484222 0.715213221625412

TMEM57 0 1 1 3 1 0.610163927365809 0.696413818386389 0.0471593735603865

PPP1R15B 0 0 2 3 1 1 0.284217063178254 0.0471593735603865

STEAP3 0 0 4 1 1 1 0.00766526196931799 0.648204567890215

ZNF35 1 0 1 3 0.942095563694729 1 0.696413818386389 0.0471593735603865

TACR3 0 0 2 3 1 1 0.284217063178254 0.0471593735603865

CAMK2D 0 0 2 3 1 1 0.284217063178254 0.0471593735603865

XXbac-BPG116M5.17 0 1 4 2 1 0.733873138685818 0.0379766110990049 0.388631796595168

EPHA7 0 0 2 3 1 1 0.284217063178254 0.0471593735603865

TAF6 0 0 2 3 1 1 0.284217063178254 0.0471593735603865

AKNA 0 1 1 3 1 0.610163927365809 0.696413818386389 0.0471593735603865

NRAP 0 0 1 4 1 1 0.696413818386389 0.00483325080615788

HSPA12A 1 0 1 3 0.942095563694729 1 0.696413818386389 0.0471593735603865

LRP4 0 1 3 5 1 0.818848824808028 0.288965031820512 0.0134136081088514

ART4 1 0 4 1 0.967502612277654 1 0.0193357288484222 0.715213221625412

ACIN1 0 1 1 3 1 0.610163927365809 0.696413818386389 0.0471593735603865

TECPR2 1 0 1 4 0.967502612277654 1 0.761468000160734 0.0124634702142331

FAN1 0 0 2 5 1 1 0.453737297296426 0.00304939405034507

CA5A 0 0 2 3 1 1 0.284217063178254 0.0471593735603865

CEP192 0 0 2 4 1 1 0.371142909514663 0.0124634702142331

ZNF699 0 1 1 4 1 0.677788552210515 0.761468000160734 0.0124634702142331

PIK3R2 0 1 1 3 1 0.610163927365809 0.696413818386389 0.0471593735603865

ATP4A 0 0 4 3 1 1 0.0379766110990049 0.124485055761882

TULP2 0 0 2 4 1 1 0.371142909514663 0.0124634702142331

SYCP2 0 0 2 3 1 1 0.284217063178254 0.0471593735603865

ZNF81 0 0 4 2 1 1 0.0193357288484222 0.313161299214229

FAM120C 0 1 1 4 1 0.677788552210515 0.761468000160734 0.0124634702142331

IL12RB2 1 0 4 2 0.981810335882373 1 0.0379766110990049 0.388631796595168

KIF21A 0 0 2 4 1 1 0.371142909514663 0.0124634702142331

MBD5 1 1 2 4 0.989846180646667 0.780354200079733 0.529967391499114 0.0430699644091567

C11orf70 0 0 2 3 1 1 0.284217063178254 0.0471593735603865

ERRFI1 0 0 2 3 1 1 0.284217063178254 0.0471593735603865

KPRP 1 2 5 2 0.996861809262073 0.532130240624871 0.0377011024044698 0.588350987718456

CACNA1E 0 1 3 4 1 0.780354200079733 0.225047014688361 0.0430699644091567

LAMC2 0 0 4 1 1 1 0.00766526196931799 0.648204567890215

PLA2G4A 0 1 4 1 1 0.677788552210515 0.0193357288484222 0.715213221625412

INTS7 0 1 4 0 1 0.610163927365809 0.00766526196931799 1

PROX1 1 0 3 5 0.994347358298145 1 0.288965031820512 0.0134136081088514

MIA3 1 0 4 1 0.967502612277654 1 0.0193357288484222 0.715213221625412

CCR1 1 0 4 1 0.967502612277654 1 0.0193357288484222 0.715213221625412

TIPARP 0 0 5 2 1 1 0.00535506716048509 0.388631796595168

ARAP3 1 0 1 4 0.967502612277654 1 0.761468000160734 0.0124634702142331

LARS 0 0 2 3 1 1 0.284217063178254 0.0471593735603865

EHMT2 1 0 5 1 0.981810335882373 1 0.00535506716048509 0.769643459130453

TULP4 0 0 3 5 1 1 0.225047014688361 0.00696117912301087

QKI 0 1 1 4 1 0.677788552210515 0.761468000160734 0.0124634702142331

ANLN 0 1 1 3 1 0.610163927365809 0.696413818386389 0.0471593735603865

HAS2 0 0 2 3 1 1 0.284217063178254 0.0471593735603865

GABBR2 1 0 4 0 0.942095563694729 1 0.00766526196931799 1

OR13C8 0 0 2 3 1 1 0.284217063178254 0.0471593735603865

TSC1 2 1 4 0 0.88165627064934 0.733873138685818 0.0379766110990049 1

OGDHL 1 1 4 1 0.981810335882373 0.733873138685818 0.0379766110990049 0.769643459130453

ZNF195 0 0 2 3 1 1 0.284217063178254 0.0471593735603865

RAG2 1 0 1 4 0.967502612277654 1 0.761468000160734 0.0124634702142331

HTR3A 1 0 5 1 0.981810335882373 1 0.00535506716048509 0.769643459130453

KDM5A 0 0 3 6 1 1 0.288965031820512 0.00179923593433847

RTN1 1 0 1 3 0.942095563694729 1 0.696413818386389 0.0471593735603865

MAP3K9 0 1 4 1 1 0.677788552210515 0.0193357288484222 0.715213221625412

BDKRB2 0 0 2 3 1 1 0.284217063178254 0.0471593735603865

CILP 0 0 2 3 1 1 0.284217063178254 0.0471593735603865

ATF7IP2 1 0 1 3 0.942095563694729 1 0.696413818386389 0.0471593735603865

FBXO31 0 0 4 2 1 1 0.0193357288484222 0.313161299214229

CTAGE1 0 0 2 3 1 1 0.284217063178254 0.0471593735603865

ZNF317 0 0 4 2 1 1 0.0193357288484222 0.313161299214229

NOTCH3 2 0 6 1 0.953836759434847 1 0.00350224393217359 0.849649605086086

CALR3 0 0 4 2 1 1 0.0193357288484222 0.313161299214229

PAF1 0 0 3 4 1 1 0.164656940060257 0.0250155717660838

SLC6A16 0 0 1 4 1 1 0.696413818386389 0.00483325080615788

ZNF831 0 0 3 4 1 1 0.164656940060257 0.0250155717660838

HIRA 0 0 2 3 1 1 0.284217063178254 0.0471593735603865

ENTHD1 0 2 2 4 1 0.408505708928411 0.529967391499114 0.0430699644091567

POF1B 0 3 1 1 1 0.0364632165930925 0.696413818386389 0.648204567890215

OLFM3 0 0 2 3 1 1 0.284217063178254 0.0471593735603865

SENP5 1 0 1 5 0.981810335882373 1 0.812756450638119 0.00304939405034507

SDHA 1 1 0 3 0.942095563694729 0.610163927365809 1 0.0471593735603865

PRKCQ 0 0 0 5 1 1 1 0.000198365463236156

LDLR 0 1 1 3 1 0.610163927365809 0.696413818386389 0.0471593735603865

STPG1 0 1 1 3 1 0.610163927365809 0.696413818386389 0.0471593735603865

GLP1R 0 0 2 3 1 1 0.284217063178254 0.0471593735603865

ULBP1 1 0 1 3 0.942095563694729 1 0.696413818386389 0.0471593735603865

ADPGK 0 1 1 3 1 0.610163927365809 0.696413818386389 0.0471593735603865

SRL 1 0 1 3 0.942095563694729 1 0.696413818386389 0.0471593735603865

MPL 1 1 0 3 0.942095563694729 0.610163927365809 1 0.0471593735603865

PROKR2 2 3 0 0 0.71710543467726 0.0364632165930925 1 1

CAMKK2 0 0 2 3 1 1 0.284217063178254 0.0471593735603865

KRTAP10-11 0 3 1 1 1 0.0364632165930925 0.696413818386389 0.648204567890215

PLEKHM3 0 0 1 4 1 1 0.696413818386389 0.00483325080615788

KDELC2 1 0 0 5 0.967502612277654 1 1 0.00101814110212027
