# Supplementary material for: Identification of glioblastoma immune subtypes and immune landscape based on a large cohort
Source: Hereditas. 2021 Aug 19;158:30. doi: 10.1186/s41065-021-00193-x (PMC8377979; doi:10.1186/s41065-021-00193-x)
Supplement: Supplementary file 5 — Additional file 5. [file 41065_2021_193_MOESM5_ESM.docx]

Tag pvalue FDR

Leukocyte Fraction 1.89153073391767e-08 1.04034190365472e-06

Stromal Fraction 0.000912464048398591 0.00557616918465806

Intratumor Heterogeneity 0.309876425760894 0.532600106776537

TIL Regional Fraction NA NA

Proliferation 0.0574649829264692 0.197535878809738

Wound Healing 0.10946654521883 0.278620752804434

Macrophage Regulation 4.22246531087333e-05 0.000464471184196066

Lymphocyte Infiltration Signature Score 0.000711721053327165 0.00489308224162426

IFN-gamma Response 0.00063483365858803 0.00489308224162426

TGF-beta Response 5.98638469762044e-08 1.64625579184562e-06

SNV Neoantigens 0.171536442153956 0.362865550710292

Indel Neoantigens 0.376632757160548 0.591851475538005

Silent Mutation Rate 0.0657687612886708 0.21278128652217

Nonsilent Mutation Rate 0.0477502451870663 0.191173324284651

Number of Segments 0.356857993318512 0.591851475538005

Fraction Altered 0.000176411945347547 0.00161710949901918

Aneuploidy Score 0.080630738393653 0.23588643948056

Homologous Recombination Defects 3.75042076648792e-06 5.15682855392089e-05

BCR Evenness 0.610804622933964 0.80912201787143

BCR Shannon 0.640227567521156 0.80912201787143

BCR Richness 0.688942417108863 0.80912201787143

TCR Shannon 0.00101586650691211 0.00558726578801659

TCR Richness 0.0124350226614873 0.0569938538651501

TCR Evenness 0.910505663747419 0.927366879742742

CTA Score 1.06147199757648e-06 1.94603199555687e-05

Th1 Cells 0.67418355034797 0.80912201787143

Th2 Cells 0.00630316099374592 0.0315158049687296

Th17 Cells 0.120718925012137 0.278620752804434

B Cells Memory 0.528328259814395 0.764685639205045

B Cells Naive 0.255087352330331 0.480807392012349

Dendritic Cells Activated 0.691431542544677 0.80912201787143

Dendritic Cells Resting 0.745486813530039 0.832574962839865

Eosinophils 0.625537103799997 0.80912201787143

Macrophages M0 0.112233655542622 0.278620752804434

Macrophages M1 0.0831935272073915 0.23588643948056

Macrophages M2 0.187729610475459 0.37539801490945

Mast Cells Activated 0.756886329854423 0.832574962839865

Mast Cells Resting 0.15270176625262 0.335943885755765

Monocytes 0.570728502054101 0.804873528537835

Neutrophils 0.0521381793503593 0.191173324284651

NK Cells Activated 0.738605738195917 0.832574962839865

NK Cells Resting 0.830303749957904 0.861635966937448

Plasma Cells 0.191111716681174 0.37539801490945

T Cells CD4 Memory Activated 0.413136970262129 0.63118148234492

T Cells CD4 Memory Resting 0.772171927832826 0.832734431976577

T Cells CD4 Naive 0.262258577461281 0.480807392012349

T Cells CD8 0.372508726598383 0.591851475538005

T Cells Follicular Helper 0.671854499393335 0.80912201787143

T Cells gamma delta 0.0857768870838398 0.23588643948056

T Cells Regulatory Tregs 0.121579964860117 0.278620752804434

Lymphocytes 0.97126565707181 0.97126565707181

Neutrophils.1 0.0521381793503593 0.191173324284651

Eosinophils.1 0.625537103799997 0.80912201787143

Mast Cells 0.278133704303643 0.493463023764528

Dendritic Cells 0.446671864760146 0.663971690859677

Macrophages 0.805773612746295 0.852260551943196
