# Supplementary material for: Identification of glioblastoma immune subtypes and immune landscape based on a large cohort
Source: Hereditas. 2021 Aug 19;158:30. doi: 10.1186/s41065-021-00193-x (PMC8377979; doi:10.1186/s41065-021-00193-x)
Supplement: Supplementary file 6 — Additional file 6. [file 41065_2021_193_MOESM6_ESM.docx]

Tag cyan tan brown magenta green greenyellow purple lightcyan pink red salmon grey

TCGA-06-2567 0.0337765131923396 -0.00360993192540624 -0.0136996394109289 -0.0249454848724127 -0.0128298524730872 0.0670340235885003 0.0421786978935219 -0.0108561796720623 -0.0274389307001601 -0.0122876015984425 -0.0123375975412455 0.00300919415718265

TCGA-12-0703 0.0249670649822308 -0.00285213503234529 0.00785638314886572 -0.0395573323336024 -0.000429794839825146 -0.0218559276166884 -0.051897253809267 0.0184714507930153 0.00269742575664262 0.0426049932616009 0.0107406866791361 -0.00437654858496427

TCGA-14-1821 0.03314948854847 -0.00458305718929598 -0.0504262431145787 -0.0371771695610963 0.0241908678690063 -0.0697637862201473 -0.0763339328123683 0.00782078853251333 -0.0546418175821427 -0.127178652819474 -0.0991646260561111 -0.0397255104719099

TCGA-08-0373 -0.0193285505904266 0.0237469306527493 0.0509263363626006 -0.00717174264159112 -0.0335464623777633 -0.00749078365915634 -0.0110036849499971 0.0224039452931508 0.0503458980790437 -0.00417838098198952 0.0369573534794447 0.0305304583798457

TCGA-26-1438 0.0794851431188637 -0.00214399828543595 -0.0110518610154454 -0.0324707519930353 0.0230660571330069 0.0398793281858884 0.106878123389716 0.0469904102464646 0.0161224803845397 -0.0247708496428264 0.0588565777013312 -0.00674069599288155

TCGA-14-1402 -0.0650253313820264 -0.0560253529076616 -0.0156380203642125 0.0537588106338787 0.0208032467537128 0.00688193909796216 0.00853619612837879 0.0115298394604107 0.014168161028582 0.0566218684816087 0.0107630102937814 -0.047329607699967

TCGA-16-1045 0.0176009270347084 -0.00101676041740787 -0.0258746474416721 0.0200883617677384 0.0765132990049064 0.0314520711157014 0.105148622843467 0.0617329097106439 0.0240753414173617 0.0337931428959633 0.0138966217488536 -0.0325738647123414

TCGA-19-1388 0.0581302343289589 0.0629471814334039 -0.0560086435313496 -0.0664104932401545 0.0420255089648052 0.125560870307737 0.0529115255501044 -0.0633586223842909 -0.0603655706772167 -0.00781107944668391 -0.0105570407862309 0.0288729333221499

TCGA-19-0963 0.00564338746472874 0.108922713197681 -0.045860246347824 -0.0236951874863628 0.0523895209628055 -0.0266963622154156 -0.00106195801309659 0.0281354770408794 0.00801709781985226 -0.0321325553607594 -0.013759556364202 0.0272770987056442

TCGA-14-0736 -0.0339012362454722 -0.0405432925865424 0.00950252546852156 0.0249599448372591 0.019735956221847 0.0407240377965172 0.0689273098936078 0.0175106264394805 0.0210451534487139 0.0342130021218926 0.0376629477973373 0.0160667966658753

TCGA-19-1387 0.0179383167504504 0.0629180427314282 -0.0627629751796884 -0.063873723227891 0.0442466921683001 -0.0166751206897496 0.0178783784129115 0.00034651059501282 -0.0211397674650602 -0.0333066065754846 -0.00253238298584513 0.0636427554080371

TCGA-14-1452 -0.00995815656987673 -0.0483655267057582 -0.0238918996212367 -0.00380427681167092 0.0133750432354486 0.0630210747234588 0.0633924714888643 0.0537554784849848 0.0124361419954814 0.0543858961185377 0.0537024663341232 0.021349414614494

TCGA-14-1823 -0.0738773281687664 -0.0310711295815636 0.0075489649438693 0.00915360758641064 -0.0239367664673665 0.0293914632328672 0.0656499401333672 -0.02080594461709 -0.0341561260962415 0.0309311613637754 0.0465292747815752 0.0792096969601374

TCGA-06-0749 0.0069150744672304 0.0932219914063958 0.0431309678045706 -0.0121470726435847 -0.0532575267102678 -0.0187384178164586 0.0056571873743238 -0.0998626088908365 -0.065084792962783 -0.0309540189414054 -0.0204935130530199 -0.0189752480750071

TCGA-06-1086 0.0811317801834837 -0.00920756580598057 -0.018443566381915 -0.0453220146778792 0.0638909974163895 -0.0257743799631245 -0.0066463257265028 0.091697579748911 0.1655183841194 0.0267594432884839 -0.00182172582984645 0.25359839084764

TCGA-12-0618 -0.025388025002474 0.0409985232713998 0.0423472510545602 -0.0189461489948374 -0.0721612133770874 -0.077101652310466 -0.0774832845247259 -0.0550559844923963 -0.0310504154569619 -0.0552748495336037 -0.0532744033809355 0.0101850995987825

TCGA-08-0525 -0.0191005366330359 -0.0455256310514855 0.0118393703720637 -0.0300398365848561 -0.0233477807137822 0.0137449703837409 0.0121955956908213 0.0131561081781243 0.0414361245710367 0.0433483625867331 0.0399931575245224 -0.00855039339935633

TCGA-12-1598 -0.0469404942900569 0.0118984659673708 -0.0332716655194676 0.000134861100593615 -0.00934787480169594 0.0601028104385179 0.0148700945754391 -0.0176399002228869 -0.0401168478886513 -0.00082250780386998 -0.0317094768976381 0.0367047958372325

TCGA-12-0692 0.0573035071847294 -0.0462801489769594 0.0193573878138335 -0.056920767023909 -0.0493234744149577 -0.00416903655658767 -0.0488214200455175 0.0267413447759144 0.0681746818796582 -0.0060112162582718 0.00266391416830918 -0.0145975815216381

TCGA-02-0422 0.0255112496594001 -0.0313907672212087 0.0224607442189488 -0.0244032327019582 -0.0178361540938105 0.00594348353455434 -0.0249419449542413 0.00473133403923727 -0.00213306869432924 0.0276880226189022 0.0132877898007308 -0.0222805694557792

TCGA-06-2564 0.00458335647629712 -0.0038064306805608 -0.0405786223018232 -0.0374905765388523 0.0397813432322241 -0.0168381527274206 0.0166314626493742 0.0259982712698803 -0.0104999742682481 0.0258661845837691 0.00066345454786128 -0.00834689424780123

TCGA-02-0004 -0.0403854757577944 -0.00638711681221112 0.0635959923255598 -0.0120605341627906 -0.0088520773167311 0.0555052494105925 0.0302975090007186 0.0917656725670912 0.222321691812248 0.0457524238243225 0.074283015967963 -0.000505185662740229

TCGA-06-0179 -0.0167779619798639 0.0897632805153593 0.034658179347921 -0.024144764324645 -0.0360934348671291 -0.00852261883253618 0.0219002539718003 -0.014376864957144 -0.000645870414597219 -0.00710766492242289 -0.016962163496479 0.017771884726996

TCGA-06-1801 0.030312437511553 0.051574179602091 -0.0504448114632047 -0.0393570643050215 0.097937002501853 -0.0317648498627134 -0.0222756200238021 0.0986666457883489 -0.00455411660486317 -0.0660990730805076 -0.0515535560346901 0.00243320501370661

TCGA-14-0871 0.0324166262612716 -0.0254605295593414 0.0655346917228505 -0.0104413623280128 -0.0817148099296724 -0.0685867364287048 -0.105614567193746 -0.0149001936408112 0.0909086551747823 0.0023215306326302 -0.0402293822288095 -0.0109338290858692

TCGA-12-3649 -0.0903894709203225 0.0221849075957901 -0.027439704583304 0.0608223214001398 0.0441152913234627 -0.0397068057012005 -0.0359852587559358 0.0102836777577253 -0.0334954538556534 0.0388690932511062 -0.0551812309018238 -0.0154558565142919

TCGA-28-1749 -0.0124438506850051 -0.0656182222738488 -0.0776753933573728 -0.00623074444681448 0.0930601426458908 -0.012331793564309 -0.0449872375964008 0.0598676315084304 -0.0146620865622964 0.0424630189133027 -0.0351343928912966 0.00451158095909454

TCGA-08-0358 -0.0517879086580458 -0.0691759078625795 0.0663499379757292 0.0932800516091838 -0.0359939344115011 -0.0170728747774258 -0.0606742481587974 -0.00810619799900586 -0.00460345578998874 0.0358582527696791 0.00308620400785818 0.017631596358164

TCGA-14-0787 -0.00427201272528951 -0.0282097908757413 -0.00108771303856559 -0.0385060124255629 -0.0311983553946789 -0.0111953105198756 -0.00409232001993737 -0.0201536889337044 -0.00938298489810585 0.0467722492004568 0.0285996511408474 0.0140229637602578

TCGA-06-0162 -0.147574013333124 0.0419855394407033 0.0720911311536639 0.123122842785077 -0.0678294075867125 0.0404201638089118 0.0443493082825036 -0.133390916004938 -0.0653990738466261 0.00415810023977611 8.35753838573879e-05 -0.00848622436720831

TCGA-12-1092 0.00386078149809846 0.0252172355087001 0.0146012447612478 0.0213147970495366 0.0474049421744994 0.00459904288092817 0.0868340249369323 0.0753063405429464 0.0649500693447357 -0.0191872588783009 0.0114752252028891 0.000613736725965133

TCGA-12-1094 0.025212783218474 -0.0124255627206936 -0.0427242793425612 -0.0105481496038039 0.0699641459403081 0.034871714836543 0.0365330792570263 0.0965328222238191 0.0288109174576575 0.0623253643939468 0.0469178976060459 0.0276199822256096

TCGA-28-1747 0.0130371228215727 0.031458108969335 -0.021893704470829 0.0193141358304867 -0.00211453235371027 0.129147670507982 0.0384369762353907 -0.07869804983454 -0.0617109282870078 -0.0153800701703347 -0.0241002317888365 -0.00115045886766484

TCGA-08-0345 0.000610113105149913 0.015839263353207 0.0504869969123587 -0.0151902978833776 -0.0484045424978912 0.053782853370999 0.0322509125354719 -0.000561152312092262 0.00484078979572835 -0.0195135740247352 0.0476675461008726 -0.012125566447749

TCGA-02-0260 0.00581174304620431 -0.0501979854203052 0.0515896977248403 0.0340149084010601 -0.00529913418242017 -0.0576608178201993 -0.101992508940538 0.00141752833461151 -0.0124133023329787 0.0277251761965822 -0.00594440087789331 0.0335557414021935

TCGA-27-2519 0.0069664045886123 0.00162337982818836 0.00396299462999048 -0.0141298634553146 -0.0091092109725448 0.0369362897247818 0.0473689511324523 -0.000348605212538718 -0.0239430571237466 -0.0378619866594552 0.0266685623805843 0.0171318321514629

TCGA-06-0412 0.0113420297903255 -0.0483089550108716 0.0304662099486993 -0.0476597211934175 -0.0328076298056758 0.00296647995306559 0.00920075618277554 0.018603545694243 0.0182107717450162 0.0365579582085254 0.0404400165237428 0.0294615264246666

TCGA-27-1835 0.0470048818108034 -0.0591471637682789 -0.0189901120131039 0.047178813430078 0.0835449878384288 0.0107347445253458 -0.0707867562413903 0.0408337862170799 -0.00806059622126179 0.0448213607397369 -0.0361746209294687 0.00589889059670039

TCGA-02-0440 0.00950759157961364 0.0351006667110822 0.0314676344641769 -0.0350594665098967 -0.0719416006813613 -0.0181029146794538 -0.0337075425698767 -0.0393527672833303 -0.0258964157466635 -0.0672950720238355 -0.0143868256947665 -0.0146678178237995

TCGA-27-2521 -0.0113160733333791 -0.0173378538436319 -0.0370383532081866 0.048987423176588 0.00512069390684156 -0.0957980947924473 -0.047026692869206 -0.00367050186226554 -0.0443848139836213 -0.101991364279109 -0.0802634933158193 0.00313896160137153

TCGA-12-1599 0.00122280851601855 0.0114946859333568 -0.0341736862065383 0.0102140067362365 0.0448158082053488 -0.0030800408517378 0.0597132269327929 0.0161105394265855 0.00404884287511784 0.0216671200739522 0.0114575455209311 0.0304992859751971

TCGA-06-1087 0.0231122544173735 0.0530739530926754 -0.0315930145587661 -0.0550498862152626 0.0118252957068477 -0.065759358029882 -0.0663637695097043 0.060197951827385 0.129863446676816 -0.0541945804434152 -0.0678061226601097 -0.0578394972082893

TCGA-06-0397 -0.0273018670640774 -0.0365065142463483 0.0300795215345726 -0.0222852842778622 -0.0715942248054004 -0.046364257405695 -0.00389441032101142 -0.0366698458293806 -0.00935954134118681 0.0454265766928215 0.0611672275503083 -0.0267439997819534

TCGA-08-0356 0.00322843718583769 -0.033606992690606 0.0449691417603916 -0.00143316329732329 -0.0306302889910186 0.103119517253217 0.000377003814049239 0.0136285417378968 0.0339236348198733 0.0387821668199481 0.0208400082971913 -0.00132553161945691

TCGA-06-0152 -0.00231090343143186 -0.0386044956783618 0.0578751285116682 0.0310556941603611 -0.0136193137019328 -0.00318517609168156 0.00396383730280366 -0.0020133777216288 0.00189248389266762 0.0104572821046478 0.014405525499788 0.00132878477744937

TCGA-06-0645 -0.041384232193458 0.012102714544009 0.0606121188894136 0.0286812166586606 -0.00648834881614118 0.0178044020627792 0.0510445559185526 0.041941337689209 0.0394144859608523 0.0205062364151763 0.0628899017866869 -0.016038618396192

TCGA-19-0957 0.0618732604297596 0.0529610229880532 -0.0796716349760817 -0.0781687255800047 0.0559157164830041 0.00314030084816774 0.0636215845736637 0.0370503191890911 -0.00973914185468457 -0.0675793938237675 0.000129671032856718 -0.0101144492111778

TCGA-12-0776 -0.00945437784552025 0.012493685306392 0.0402295221065058 -0.0132102413748883 -0.0252237607769373 0.0338119123492476 0.0744188052262077 0.0240748421042579 0.0168271824355521 -0.000752638832446719 0.050045220271886 -0.0115933116482759

TCGA-15-1449 0.0426662417684458 0.0956902205587574 -0.0505334196266532 -0.0567141301996055 0.000425514656999389 -0.0320246059002427 -0.0565068252541125 0.00236341459699681 -0.00719722256606726 -0.0470032933196168 -0.105008462480321 0.0456089564899568

TCGA-28-1751 -0.00129479634959677 0.00970186949832 -0.0362269375322686 -0.0114665839901809 0.0569584523091271 0.00991176185138003 0.0837310270793942 0.0764827456085107 0.0729743286848199 0.0541397488040619 0.0907183895782245 -0.0560373063476188

TCGA-12-3651 -0.0701585670963341 0.0522292370994984 -0.0288941492716752 0.0254448424947459 -0.0370048967370874 -0.0194015635528264 0.00277769714491435 -0.0743677191524877 -0.0731085410875786 -0.0609033411565432 -0.0763658336927567 -0.07793745529658

TCGA-12-3650 -0.119941664377959 0.00769990309000181 -0.0403553071123916 0.0620075336451446 0.0392335872006289 -0.0463275777019189 -0.0159107453537585 -0.0150184516686117 -0.0609677460085539 -0.0107808149056938 -0.0728920472901985 0.00744122575581533

TCGA-19-0955 0.0242864298592444 0.0174688353877721 -0.0462935244384684 -0.017766971940499 0.0689610487581842 -0.0133058936468205 0.0251065763339461 0.0352033098629094 -0.0127096322462233 0.0502411394099634 0.021336308859296 -0.00545874802552178

TCGA-28-1745 -0.0447093381305816 -0.00881799083623856 0.120679898400946 -0.0122212032441531 -0.0954750377964574 -0.0403997900533929 -0.0275442290969699 -0.0716298446821488 0.0177756182496018 -0.00325831634567632 0.018240022323242 -0.00178820787066601

TCGA-06-0409 -0.00681636073372063 0.00200870877024961 0.0671624393043007 -0.0223617601674859 -0.0165772662020403 0.00118092818463124 0.059847027281874 0.0292765267948239 0.0486520203964624 0.0170987802750662 0.0609455695983214 0.00269858828412465

TCGA-19-1787 0.0117888684978399 0.00809988307677199 -0.0231225705607734 -0.0291738364327923 -0.0106835147375683 0.0883111700739097 0.026527611185515 -0.0130733888794468 -0.0300635905849864 -0.016271232027178 -0.000361467051324824 -0.0108823264089012

TCGA-15-1446 0.0505613995548121 -0.000260447411301416 -0.0458284868054641 -0.0264679696461501 -0.0223799185391321 0.0504520113729548 -0.00355643600879473 -0.0523521797896743 -0.0260944798853282 -0.0218492919137862 -0.00279856568113767 0.0393052496416138

TCGA-14-1453 -0.0160199013990127 -0.0440920042844459 -0.0541725125485296 0.0250190486596623 0.041548769623387 0.0239743589072974 -0.0037674803316433 0.0161212764677005 -0.0445621773745711 0.011003685626097 -0.0530223706603558 0.0239654206278138

TCGA-19-2620 0.0193077560140243 0.0611826542194361 -0.0445554338719437 -0.0244030919760031 0.0121183004777945 -0.0370988551486766 -0.00497267943498002 -0.0553826689474384 -0.023001538704199 0.0203976882332113 -0.000750700844770678 -0.0558274945319785

TCGA-06-0649 -0.0111948850665896 -0.0119032128407135 0.0403976231016539 -0.022825903621797 -0.0374781944067962 -0.0250183270239163 0.0275447266321466 -0.0273422643108506 0.00860169487624643 -0.0308322154890866 -0.00729100816599678 0.00120168012637123

TCGA-12-1597 -0.0825695222528313 0.0765042586214678 0.00970634151645234 0.0386735782038509 -0.0313750490306496 -0.0720585588470564 -0.0219390459648683 -0.0343045163964493 -0.0443355430441393 -0.0520117675371824 -0.0559856232035558 -0.0322782364453027

TCGA-02-0337 -0.000139051211530376 0.0351549288642694 0.0673445079939515 0.0609846215537065 0.0141015007207493 -0.00488505433722134 0.024734550253654 0.0651172165069238 0.073241912480471 0.00424506377776074 0.0555358058307911 0.00404060541971235

TCGA-02-0285 0.0138467685399847 -0.0390130247632666 0.0382775507354253 0.0140204384133452 -0.0212404054105359 0.00174418457880322 -0.0142411233575802 0.0142970720324588 0.0027010255466284 0.0290762138440899 0.0254638086826842 -0.0359192489972331

TCGA-27-2527 -0.00142683189626774 0.00815912728334353 -0.0131183754770536 0.0643927550211575 0.0154256089275257 -0.00476891819704301 0.0601939071420961 0.0455584926253989 0.00976264573326593 0.00794933750217565 0.0190255074934781 0.0166451016759251

TCGA-02-0332 -0.0311676423761341 -0.000292272777464492 0.0842970432646414 0.0396773598116563 -0.0324223886984839 0.0422236998317399 0.00954025808443256 0.0106310563269305 0.0213723263916665 -0.0249705431458179 0.011689884619262 -0.0331410272780602

TCGA-14-0865 -0.0223642273468566 0.00466909047385801 -0.0480703983829519 0.0107168167709651 0.0092785462217048 -0.0683261556796379 -0.0523061756025544 0.00375300527623706 -0.0588147400753603 -0.0936203791936421 -0.0998875626793615 -0.0325234110685614

TCGA-12-1088 0.0417531233690725 0.00990340510548215 -0.0376144575704131 -0.0529873383183654 0.0293712664578094 -0.00217857159172488 0.0392747134229193 0.0531193130222301 0.0191242502805348 -0.0397154553659502 0.0112154325375362 0.0131993066048641

TCGA-12-3646 -0.103946622045319 -0.000487069213785343 0.039530461103304 0.0181674763031374 -0.0511849548602939 -0.00110846189063285 -0.0607806487131562 -0.0614248588308759 -0.0459806452985586 -0.0618817993868783 -0.0630924518537045 -0.0222857749801145

TCGA-27-1836 0.110168143853708 0.0861849065713802 -0.0380658545370726 0.0537671320682214 0.0405394852288086 0.089361311647237 -0.000384232592621301 0.0416708283021664 -0.0235613114451841 -0.0518615929102194 -0.0496992933315884 0.0110860662845628

TCGA-08-0524 0.0169627773385873 0.00699350429622269 0.0486999644283423 -0.0200714823799915 -0.0478225040644656 -0.106273114518749 -0.107447470061032 0.00746384047713727 -0.0136119110790343 -0.0953948003615103 -0.0635892722209257 -0.0041622883050805

TCGA-08-0244 -0.0838365434548023 0.00417398427505909 0.0552186215656349 0.0244280320766433 -0.0621560885960275 -0.0175754766224624 -0.0358093707071379 -0.0480181047741626 -0.0242912992951799 0.0246645897543914 0.0120157659701752 -0.0276671606157108

TCGA-12-0691 0.0518985128192741 0.00144121951341049 0.021511566561116 -0.0387351329936659 -0.0758364076801365 -0.0289553209056624 -0.0454698730510673 -0.118723809962001 -0.0664570572548338 -0.014249991947557 -0.0455105166681934 0.00438517409002861

TCGA-14-0790 -0.00937631757179223 -0.0368137333033395 -0.0412550017407184 0.040800189056063 0.0390888993861402 0.084173639656248 -0.0467731583857456 -0.0518174607355903 -0.0378656171568999 0.049208826774416 -0.037348102982845 0.051348899062641

TCGA-32-2616 -0.0371891191612117 -0.0383280790727308 -0.032404854277707 -0.0279388017026105 -0.0253139279082 0.0569835440800462 0.00393178192920296 -0.0256238421554547 -0.0496153674306742 0.0200135764573244 -0.00227494623381819 0.0173678289419916

TCGA-41-2573 -0.0233082347218502 0.136070204767388 0.00653124480108384 -0.0175836463226953 -0.0224847365941219 -0.0338427019496429 -0.0060599357958995 -0.0296094421998167 -0.0398683387220198 -0.0827893265607853 -0.0403355411361014 0.00397875488333853

TCGA-14-3477 -0.0851843454896545 0.0184031130938006 0.0261503486739155 0.0192168471489861 -0.0443739877576382 -0.0695702344728354 -0.0772730046238707 -0.0415022570005048 -0.0487886030866649 -0.0911637483747748 -0.0788308786798599 -0.0738264507582135

TCGA-12-1095 0.0322153737423202 0.0286920214250456 -0.0359611232847261 -0.0276759370894792 0.0179305982401775 0.061634716711863 0.0868403824926053 0.018770020158959 0.039020806728495 -0.00353904932648444 0.0286124830887589 0.00956305937450533

TCGA-08-0517 0.0333654016962326 -0.0261021339854356 0.021051071277505 -0.0510631983694591 -0.0624758611857112 -0.0293988392079545 -0.0717390605421534 -0.0294197668884713 -0.015607616758228 -0.083252357642759 -0.0335245352208581 -0.0144242561578285

TCGA-08-0522 0.0136025270698019 -0.0338172697331611 0.0638527806258194 -0.0483991179483283 -0.0621799186655007 -0.0195728887792804 0.0256981061254173 0.0145428818498065 0.0849031903744129 0.0513625125668027 0.0908582499357317 -0.00879179256694861

TCGA-19-2621 0.0115241163714828 -0.0740151138909338 -0.0453164662156053 -0.0408063870064755 -0.0313186548662964 0.106049237566136 -0.0503374098792481 -0.0744878650433612 -0.0563389461040169 0.0395128978845423 -0.0338347578990694 -0.0327867021904687

TCGA-14-0812 -0.0417161875942593 0.069302599594906 -0.0247875921933511 0.0156726737280299 0.0103171378130611 0.0501643247037846 0.0704399521934417 -0.0157800937446883 -0.0342296571186259 -0.00565535585469992 -0.00440437307436087 0.0523823586538147

TCGA-14-1458 0.016231266164199 0.15758964103726 -0.0509063562989959 -0.044211060343302 -0.0155254075097319 -0.0562262711146135 -0.0345637687522499 -0.101682324449603 -0.095760021585343 -0.114928925032635 -0.110673094047427 -0.00382507397589269

TCGA-08-0360 -0.0253162376285895 -0.020077377263064 0.0661466071590072 -0.0231758867114195 -0.0570584999351051 -0.00143500604901568 0.0311866783373889 0.00609019118976324 0.0281842825483795 0.00542178947137785 0.0797980771689686 -0.0197392809961708

TCGA-02-0451 0.000742686162518926 0.0172545499936792 0.0325029531534463 -0.0220027686916106 -0.0546560164420318 0.0270820930628258 0.0201078620155026 -0.0491480115611863 -0.0395821997051038 0.00225956473184709 0.00513493166433756 -0.0121458861022914

TCGA-02-2470 -0.0275278385641837 0.126674538677143 0.00363714314903037 0.0894430692327645 -0.00690969439949117 0.0412778487264149 0.0596672733819411 -0.0103401500245689 0.0423709672283088 0.0136356879463122 0.0542501424201915 -0.0137123922482383

TCGA-02-0317 -0.0264861901366772 -0.0795310544368453 0.0601733508270224 0.0613599826283994 -0.0418693345813707 -0.0463062340616063 -0.093417578676145 -0.0204165359073957 -0.00389807560943531 0.0557575639556714 0.0162199013589396 0.0200586844787675

TCGA-08-0510 -0.00435864653546072 -0.0524200100905772 -0.00707989626832131 -0.00952569406254367 0.0223700729341753 -0.012733765958355 0.0431379405972946 -0.0259653988842027 0.0113353284261728 0.0689339064007228 0.0621890336876963 0.00365508200989422

TCGA-02-0048 -0.0109354471313471 -0.0150068978590385 0.0116326534670462 -0.0654103224185553 -0.0508946899089325 0.0234153534086446 -0.0817660110196984 -0.0448517663409641 -0.0337640305277958 -0.0292470329600389 -0.0783689458792466 0.0331839599956825

TCGA-14-0817 -0.126568714753431 -0.0117631317594082 0.0955964162673327 0.139082664345603 -0.0279815337287166 0.0433660725169503 0.0260576506033381 -0.0285472207911551 0.00641293157070815 0.0142051300996786 0.0178728709517308 -0.0158207592541602

TCGA-19-2619 0.0209966250397275 -0.0169181183006826 -0.0525583737900067 -0.0218324050836767 0.0552277630219727 -0.0301135898906573 -0.0292190308843237 0.0449069210038776 -0.00702409220957903 0.0283693080347367 -0.0136599805501255 0.012194693831641

TCGA-28-1753 0.00863904535357624 0.0194723816235959 0.019518938545916 -0.0201895733748939 0.0268543163193738 0.00852772883469239 0.0511263721650221 0.0765937256670089 0.0959648935500064 -0.00947952911594857 0.0146632207900648 -0.014020822543026

TCGA-08-0349 -0.0378806327643549 0.0142328444669902 0.0549579695337559 0.0272244600602306 -0.0318121042886666 0.0416967891617743 -0.00245238970604323 -0.023375860296638 -0.0167929594992577 0.00519458270043439 0.0143759462206401 -0.000861225813489829

TCGA-32-1982 -0.0555375029460334 -0.0198532169906725 0.0632283702203083 0.158093762169933 0.0931654031328546 -0.00101610622506277 0.033365199389415 0.0358054565019765 0.0307957179336217 0.0535213675068352 0.0270222395065798 -0.00244390082501608

TCGA-12-0615 -0.0710845539418912 -0.0157679655340396 0.0718171431434806 0.0848257127719163 -0.0212123294250313 -0.0301202689613383 -0.0357603661154026 -0.0751377483813854 0.0201498107264309 0.0332907965461049 0.0323624913020479 0.0149661798675034

TCGA-06-0875 -0.0306519907381967 0.008718317084207 0.0507942658498997 0.0211286569203865 -0.0423680146842403 -0.0677462639533158 -0.0307390608729799 -0.0167990364522667 -0.0194749568507502 -0.0697638037119063 -0.0361945917170185 -0.00886256923008557

TCGA-12-0656 0.000403758272237746 -0.0105414432404588 0.0357689772440918 0.0188602725914697 -0.000765647641227751 -0.0468702173562062 -0.0458370980041431 -0.00933614467816903 0.0146859283069828 0.0468189825185862 0.036531604376462 -0.0102832808156509

TCGA-08-0516 0.0805723014311952 -0.0110955201616649 -0.0137503720009296 -0.0623281727179767 -0.0508362526178658 0.0254128909054643 -0.0522899167642048 -0.0752658824783369 -0.0497539826605568 -0.00671257108096336 -0.0372506821459924 -0.0427101358300165

TCGA-08-0390 -0.0452764770745288 0.0172029603803333 0.0597502533626192 0.0604193473957528 -0.0572013099621029 -0.0124367562195152 0.0231703236259653 -0.0672679262799128 -0.0193365618799878 0.0229107987133911 0.041781206126024 -0.0152196388953565

TCGA-14-1454 0.044670092109085 0.0233606675327316 -0.0639444630234855 -0.0453711272123656 0.00117278644479865 -0.075297930021349 -0.0641401555358467 0.0239198164286653 -0.0307581083842343 -0.0907707820943521 -0.106157076938729 0.0314986766147696

TCGA-12-0820 0.0213392816758538 0.0115799953334516 0.0490591971835464 -0.00538288276259551 0.0158562383960649 -0.0690178326861825 -0.0661727698298069 0.0467997360381988 0.0332746362374899 -0.00690995804660987 -0.0153872747359469 0.00881001917845604

TCGA-02-0111 0.00326038583801653 0.00285832753448539 0.0548266997031616 0.0082120325344095 -0.0594985181938989 0.00961312742536987 0.0434427792934889 -0.016879131363787 0.0267978424151548 0.0267919083441717 0.0639738554458703 -0.0016464334154908

TCGA-06-0745 -0.01673176753242 -0.0329020386217034 0.0236383832988163 -0.030717791545216 0.00576508966289077 -0.0396500825686936 -0.00676237670210276 0.0379416276486698 0.00362182444632643 -0.0102076933625202 -0.0144965488264274 -0.0249033895395041

TCGA-06-0877 -0.0239396656822721 -0.0398158878208057 0.0552576886266879 0.0287803332925421 -0.0208164083928496 -0.0331190693858905 -0.0174714590855301 -0.00182104314417459 0.029564013026551 0.062579740258868 0.0519219563705717 0.00454953759836933

TCGA-02-0333 0.00656045244122029 -0.0355286722999961 0.0490567668160449 0.0381706065031948 -0.0838636188652038 -0.0318500459044746 -0.0766182125088585 -0.0766249747151427 -0.0189893299956418 0.0527759285964246 0.0292208770650964 0.0121673599483418

TCGA-14-1827 -0.106081272527954 0.0375117113059921 -0.0551171915380356 0.025342367649712 0.0145135555259336 0.068042343535474 0.0404203526484692 -0.068456159703387 -0.0896017887611699 0.0212687361546762 -0.0653027535265578 0.105433331235223

TCGA-06-2570 0.0688951373184074 0.0388562943412426 -0.059391026872483 -0.0273394236774922 0.0503973992111181 -0.0320053546334886 0.0154649757001994 0.0351062196254609 -0.0321637373466528 -0.108518960411935 -0.0778894102757288 0.0531115117118765

TCGA-14-1825 -0.0195829315822873 0.0180639970267277 -0.0707537374881499 -0.028524526547224 0.0403432113370178 -0.0665847461178622 -0.0297629559794582 0.0270884661686951 -0.0561255885307904 -0.0755690742718765 -0.0873636217052597 -0.00539166205263152

TCGA-12-0616 -0.0547978196123843 -0.0236419794939747 0.0641869688140708 0.0316820192918628 -0.0598364139662525 0.00857368003714424 -0.0293756322128639 -0.0488267495585149 -0.0158771003234004 -0.0347286304008141 -0.0111505133512306 -0.00773461689062518

TCGA-02-0432 0.0484265347031547 -0.000901228187121048 0.0292437764363022 0.0319529448022321 -0.0541397141032714 -0.0899500418189075 -0.0615406127005563 -0.108685796174616 -0.0796159167731432 -0.111648908266347 -0.0798327851000967 -0.0277923563160518

TCGA-02-0290 -0.066820551429627 -0.0666918585747697 0.0712068301393601 0.127792330202084 -0.0100302325420339 -0.0371038533913355 -0.0334130830008006 0.00652566612336856 0.0187753879784287 0.0659443780350251 0.0584605713845284 0.000628127314416249

TCGA-15-1447 0.0486206590801166 0.117868515473952 -0.0683614840830585 -0.0548675552536636 0.022287754052196 -0.022542583365596 -0.0461191034913387 0.00484449962188487 -0.0112145466512161 -0.0515127276666784 -0.106491758924944 0.0751502472104341

TCGA-12-0818 0.0649640300157292 -0.00161469025145165 0.0388076600527372 -0.0214031318564009 -0.0332590443497113 -0.0933306643217659 -0.0868311845637487 -0.0670749280265387 -0.0443786548048089 -0.0890252395719824 -0.0685595649029532 0.0239680171103019

TCGA-27-1832 0.0396205282499653 -0.032011126996847 -0.0127240929646527 0.0375204144968963 -0.00738949102856827 0.0616883528894608 0.0840579823992078 -0.00951811670333285 0.0212780294084895 0.0329411506572225 0.0928912781310277 -0.0197323603310686

TCGA-12-0775 0.0413113910026813 0.0375192572108823 0.0418195493244244 -0.0129511921180469 -0.00929786255322615 -0.0182034090144723 0.0390205897589013 0.0429104269912152 0.0717920018847186 0.031373923813653 0.0654984837081771 -0.0253459097142408

TCGA-08-0380 0.0130696782224354 0.000405530955912544 0.0458423210505959 -0.025469838756605 -0.0623623269690321 -0.00549484435509471 0.0414367295382912 -0.0760139627564685 -0.031928996792093 -0.0277698335545665 -0.0218466380284038 -0.000970756509582128

TCGA-06-0876 -0.0420103340224293 -0.0307117247276169 0.0723270269838902 0.0725711891142336 -0.0457928033911415 -0.00215507303823402 -0.0122214477431518 -0.0348740954610676 -0.00770406848004836 0.0230863898746406 0.0168547480917639 -0.00882801792184887

TCGA-19-1385 0.0189156255488465 0.0316377294442767 -0.0309850576519054 -0.0577682372651811 0.0864335207464058 0.00581434258982414 0.0969343249349463 0.0848014674088204 0.160251267761213 0.0707417234599616 0.0951740378032324 0.0403367727034598

TCGA-02-0023 0.0267545464494858 0.0132903398811 0.0481969383297096 -0.00222193601338297 -0.078069790281755 0.0542412574136861 -0.0312224238455462 -0.0632953937865953 -0.0173267049608691 0.0309344351172676 0.0040636867036062 -0.0097011909548497

TCGA-06-0182 -0.0927462667360412 -0.037251792226297 0.039032967529031 0.0282867289059411 -0.0344221226088552 0.0820324380812285 -0.0109854503442931 -0.0414599303523247 -0.0274200579939697 0.0118515434080743 -0.0254936246933334 -0.00551084951215258

TCGA-19-1788 0.0655993418162943 0.163957077466763 -0.102551162852518 -0.0256309727356077 0.00285530444550835 -0.0600885583508411 -0.0161830582531193 -0.122027649797403 -0.126455515257025 -0.0959961572512557 -0.114781317186614 0.126177182181239

TCGA-28-2506 0.00646362502526816 0.0461415495712266 -0.0448132582710795 0.0777326186825785 0.0272530199403629 -0.00645895765353368 -0.00218189324587628 -0.0689100914182552 -0.0737040137626973 0.00562931553272393 -0.0765938390545032 0.0424522397341517

TCGA-12-0828 -0.0387754417339083 -0.0295578625796784 0.0549282120122937 0.0478293724832515 -0.0314960919032648 -0.000221731975098859 -0.0195018028042969 -0.0118490914242536 0.00616843445134151 0.0115050873378789 0.0137839684926626 -0.0325214022245317

TCGA-06-0747 0.0355191155547702 -0.0395415383438313 0.00377156463763801 -0.0616836310861966 -0.050475087064459 0.0117895084867764 -0.0597602621742377 -0.0532506795229408 -0.0359094422527771 0.0244836114811063 -0.00240890817032107 -0.0736875651971086

TCGA-02-0456 2.73212174640215e-05 -0.0449782218120794 0.0474880273903163 -0.0277590374174371 -0.0803641615396664 -0.0294825708213049 -0.00174851107602109 -0.00228771245955001 0.130403169184808 0.017299705794713 0.0492725905524158 -0.0107648614789494

TCGA-12-0821 -0.0135547561543215 -0.00873703501001907 0.0508138543900842 0.00142386400102875 -0.0244405249413744 -0.0423746415143112 -0.0520724000634403 0.0107130529858745 0.00939773703413446 0.00202107011665111 -0.0188221736792671 0.0040966355970693

TCGA-08-0347 0.0314257011904836 0.0388336216931865 0.0423357056792426 0.0108526673409374 -0.0479688008601243 -0.021751113652728 -0.019573879136983 -0.0313700338740952 -0.0119116332821495 -0.0382977234407594 0.00765790188497302 -0.019410495676005

TCGA-16-1055 0.0796233556148125 -0.000748639853631214 -0.0462545291098047 -0.0927412799096607 0.0428179068539718 0.0491667851975422 0.0392829004607858 0.0830672483250194 0.052130012052032 0.0239606983771294 -0.0158802537800142 -0.0262122792113074

TCGA-02-0015 -0.00572351684180814 -0.0277536147267027 0.0521041796968514 -0.0615783582460798 -0.0974536299795905 -0.0663107200998248 -0.0482829390690301 -0.0268870704586271 0.0388158505701214 0.0143581327501021 0.0311831035066335 0.00979642073177808

TCGA-08-0359 0.022243807279282 0.0598489173074578 0.0233173142173221 -0.0119505285495524 -0.0641507557337042 -0.00491086706806469 -0.000906588699331491 -0.0513242515527491 -0.0083891967322607 -0.0451935630285515 -0.00500091045918945 0.000374419553311686

TCGA-02-2486 -0.0405128808028595 -0.00640922988561815 0.0235039116986977 0.0678428357864864 -0.074785683826446 0.14093193772628 0.0909790586621089 -0.0540139249832453 -0.0148296835661303 0.0376220247547206 0.0859508751099293 -0.0111201662306008

TCGA-12-0819 -0.0355578985292546 0.0509716043643768 0.0564088383224902 0.0450117582762938 0.00442369427481104 -0.0153486173122696 -0.0551472207686637 -0.0480246670301928 -0.0180751355308878 0.027787014476707 -0.0164245674223165 0.00878212593680008

TCGA-06-1802 0.0361882254231416 0.0416992653173659 -0.0100235734100664 0.0172429679098074 0.0327541047782027 -0.00270498965382426 0.0461850112918917 0.0639372479839012 0.00546421318471222 0.00874314079899377 0.0354236943194988 -0.0102153304140394

TCGA-16-1063 0.00532645183614589 -0.033109051862887 -0.0660465774353458 0.00300534341782762 0.073553289403979 0.0419848337003582 0.0122556516820939 0.0612304530479427 -0.00356758165636057 0.0228513041106346 -0.029976393634222 0.00189407761295709

TCGA-27-1830 0.0397763991620115 -0.00757534280051084 -0.057912792020611 -0.0590832848221269 0.0595765704165312 0.0286477589771519 0.0518720757523985 0.0554679248140676 0.0369330672588966 -0.017239587380596 0.0331050924526381 0.0208328418869002

TCGA-08-0511 -0.0574136143360453 0.0201980966630607 0.030009030250309 -0.00673219286174426 -0.000746918041247695 0.0372966182688918 -0.0216791127501493 0.0185754572693143 0.0322003999616728 0.0501296619484998 0.0352651466077668 -0.0446473994034273

TCGA-08-0512 0.000312574741690015 -0.0166547827163897 0.0693491713920569 -0.00902386624201154 -0.0493782540117854 -0.000856114294726727 0.0155487312470165 0.00625087811946906 0.0748936445796542 0.032381246650003 0.0986287185889049 0.00432540320118565

TCGA-06-0413 0.0781105480379131 0.0517096271510474 0.0143496059733011 -0.0477607755198187 -0.0320028669826706 -0.0948741148460622 -0.0670385781890436 0.0255902046735121 -0.000420437975947148 -0.0666400081252931 -0.0347165935704899 0.00999277951764111

TCGA-06-0744 0.0262768550278567 -0.0168440202868579 0.0263133371905481 -0.0220065133417278 -0.00392062825362311 -0.0425926515034413 -0.0512890524744971 -0.0137382871927399 -0.0223641689025967 -0.00105848933940618 -0.0532644905814963 -0.0354516185618227

TCGA-14-2555 -0.122813082719404 -0.0768602436039999 0.05830752861932 0.101129911597867 -0.0571623746648558 -0.0289543468100632 -0.0621264667125713 -0.0425856673524532 -0.0168360091126786 0.0502166594506888 -0.00244727563812189 -0.0891740575074904

TCGA-19-0960 0.0969555051514006 0.0283152696272506 -0.0752385019216899 -0.0458562150150561 0.0154611591014592 0.0510081131519125 -0.0130757679737609 -0.000386522959823449 -0.0685218206700975 -0.0872508376352598 -0.102065442128517 0.0722609567352373

TCGA-06-2559 0.0390154558707408 -0.00154519127508349 -0.0576029297425109 0.0144880091543216 0.0280347115430085 -0.0266875426718948 -0.0122734083226193 0.026139618966626 -0.00247713886087852 -0.0150309127636197 -0.0403398189936418 0.0302348526045262

TCGA-08-0350 -0.0146654010706227 -0.012341331926281 0.0683934444226279 0.0338236835457485 -0.0333990190418237 0.0178959603763444 -0.0247191604279109 -0.0137451078923058 -0.00523250765590897 -0.0535721284274813 0.0084944636261666 0.0030517741176337

TCGA-02-0325 0.111725461139096 0.010707159403374 0.00547564324996754 -0.0495379750710478 -0.0710552107112285 -0.049369271608639 -0.0453966104719672 -0.0381959881243437 -0.0150068541763934 -0.0506856191073942 -0.0137620465745923 0.0610186084453975

TCGA-19-2625 -0.0105766649102049 -0.0330560986813146 0.0368958454546235 0.0240477565943554 -0.0673573803247345 -0.0238424571136708 -0.0331448875717641 -0.0448300244747567 -0.0109537341590655 0.024594217281192 0.0350708783783372 0.00643994224959309

TCGA-02-0266 -0.0797429717204765 -0.0270205120890425 0.0796060416624337 0.0962790571302629 -0.0646433899353772 -0.0807147423821001 -0.0413301509906726 -0.0256972382918104 0.0335033668806919 0.0279467345451736 0.0559309874322406 0.0013475329668126

TCGA-27-1837 0.0715268920673201 0.00909139599401406 -0.0601602625256191 0.0371094005461726 0.0393439635826012 0.0936458384558523 0.00452431688263139 0.0358668650583931 -0.0268038002191691 -0.0147272030650847 -0.0369362239115973 0.0116832237772546

TCGA-16-0861 -0.00953165431609316 -0.000115485743802713 0.0744307049937044 0.0885935313738524 -0.0845583049629141 -0.0241167720510991 -0.0336063637171359 -0.0605690504297774 -0.0033682213124184 0.0134615098977537 0.0394831992232106 0.0415690248210447

TCGA-12-0827 -0.00916083556681433 -0.00103445025792278 0.0670984635494157 0.0763902901240433 -0.025096077004481 0.0086262825620477 0.0138919589257881 -0.0253280865270487 0.00893733436051565 0.00304873342820796 0.0205333530700492 0.0162306719745978

TCGA-12-1097 0.00346820269163276 0.00403904602864235 -0.0234992041903073 0.0673682222216037 0.0292443774088359 -0.00595844061982481 0.0213117497591011 0.00889303670384036 -0.0124542013262305 0.0136607251892566 0.00361112509409532 0.0109455376780683

TCGA-02-0070 0.0369547763199444 -0.0400283998972842 0.0376363118894179 -0.0413430619104129 -0.0494971826290801 -0.0117333157535607 -0.0341336502535773 0.00498985676932685 0.0143799187077034 0.00805212891782944 0.034801157707359 0.00672062038896367

TCGA-28-1760 0.00350275766105976 -0.0272591230733642 -0.04571716584298 0.0403677333214234 0.0473667858682836 -0.00174055717076632 0.069488890500015 0.0518742191734688 0.0232688261167177 0.0589321105016694 0.0704284672449444 -0.0890413863145832

TCGA-06-1800 0.0230640872796717 0.0292540127983083 -0.016497431803728 -0.0214943113857363 0.0526703985150731 -0.0279459979693116 0.0481934174659856 0.101792802008924 0.174253018681431 -0.00736410266513455 0.00854810949839 -0.0121828318803852

TCGA-12-1093 -0.0114069373925683 -0.0241518801446129 -0.00280880952722307 0.031375188085501 0.00868358148212959 0.0314070000335338 0.0682371207549217 0.014167421042504 0.0282918063733083 0.0190385809325851 0.060173992780427 -0.0175982506377751

TCGA-12-1091 -0.025412558211073 -0.069765357142596 -0.0385024860726767 -0.0353900879578632 -0.00969188979391971 -0.00601967983894012 -0.00906566076750785 -0.0166408282040202 -0.0264943193561032 0.0347580479476663 -0.0178693465599028 -0.0101608460602007

TCGA-28-1752 -0.039939554059899 0.0734226082266593 -0.0324906813110252 0.0245048545689817 0.0814383160475387 0.114951553678988 0.0972690716895903 0.0247896869835339 -0.0120933871714191 0.0107985018524682 -0.00130344325419018 0.102678265024794

TCGA-16-1060 0.032101671131714 -0.0304597057227009 -0.0112013501586581 0.0127696483223097 0.0235711505685807 0.01949342980106 0.049876236186699 0.0558800735253683 0.0724557078704008 0.0373375658585739 0.0559242131142254 -0.0176474008452432

TCGA-14-0786 0.00976820342490014 -0.0726171738926789 -0.0519829207695007 0.0167952353075597 0.0412678788703062 -0.0389498888198653 -0.00460742579604177 0.0219566388697881 -0.0269941414517084 0.0837396861318361 -0.00354527793236446 -0.0165703463062891

TCGA-27-2528 0.00761693849698607 -0.0239705957196638 -0.0322092787737836 0.0230519652258651 0.0538843261818546 -0.0241528469428537 -0.0377842068879155 0.0545294689708207 0.068484508345663 0.0485810591663318 -0.00679888356128687 0.00825152293830563

TCGA-27-1834 0.0315854548059115 0.0165412208799718 -0.0511675838536312 -0.0156295775653971 0.0233847648480944 0.117937457969604 0.064882948774272 -0.0432500475914174 -0.0605577455024764 -0.0373379018424448 0.00213415078044426 -0.0467271360089865

TCGA-06-2565 -0.00514574173323175 -0.0470971066574497 -0.0232822267908353 -0.0341526730350856 0.0505728024050641 0.0272901459926984 -0.0255543208879734 0.00974981368194098 -0.006130308671114 0.025584550210848 -0.0260128226426024 -0.0392410256781022

TCGA-12-1090 0.0238004422186714 -0.0843982433044653 -0.0326783824273907 -0.0165953338361528 -0.0538058877443023 0.0440993342751121 -0.0647731404963113 -0.0772824873336201 -0.0308641741262381 0.0149141925617789 0.0232275548744532 0.061020697137675

TCGA-02-0068 -0.0267219025252143 -0.0422603481872687 0.0669638754058437 -0.0121417080211321 -0.0377594595776315 0.0362409695327164 -0.0228848101886776 0.0117328736462182 0.0457017100440821 0.0284835480227636 0.0281655920802819 -0.0126110122638934

TCGA-32-2638 -0.0069269916994044 -0.0219752950923157 0.0143038209881929 -0.0265344793371685 -0.000796973943909733 0.0887444113142607 0.0200166686569872 0.0513864046426052 0.0325724196673139 0.0468491420249645 0.0257451683206878 -0.121965449766993

TCGA-27-1838 0.0619171526898129 -0.0260370824594662 -0.0246255917808855 0.0168933928056748 0.00459980925991539 0.0566838203391871 0.0213289268012838 0.0398746039149317 0.00893769627013266 -0.000708829691572581 0.0150684777735672 0.0434198965024727

TCGA-02-0324 -0.092098999036295 -0.0122434506008381 0.1050840562507 0.172493919968345 -0.0339534452837775 -0.0127711182295022 -0.0346917716990873 -0.00059045645956115 0.00958220137579897 0.0200798473072915 0.036277667944846 -0.0378624949488358

TCGA-14-1795 -0.00445854958227099 0.0394081300499281 -0.0227130538230121 -0.050623899219582 -0.00648941450164294 -0.0159771538385826 -0.00728204344889239 0.0279554397379344 0.0135828928097642 -0.0232947161755382 0.0385094660142296 -0.0103150238136172

TCGA-02-0016 -0.00216036823961995 -0.0128370314158296 0.0482329443222791 -0.0552240126052881 -0.0299221486081281 -0.0278660891279641 -0.0568579220713224 -0.0508867992268546 -0.0267464395342727 0.0274966504157202 -0.0245191179634682 0.0122489790201979

TCGA-28-1755 0.0706271979657701 -0.0729862966283288 -0.0874014053125276 -0.0102903377409992 0.00419588290169034 -0.0316883513889887 -0.0479733432366812 0.0213155364274986 0.0220332145093368 0.0469532946003316 0.00725170941484946 -0.0416443428720399

TCGA-27-2524 -0.107440876006289 -0.0314128261837729 0.0676485626161169 0.144301793053908 -0.0304120716355477 -0.0189314229622057 0.012118997066226 -0.0108401918685671 0.0466548754492074 0.0593942370242067 0.0906027411964077 0.0568855637625111

TCGA-02-0446 0.00654869738604262 0.0581285138714075 0.030888432479129 -0.0520578650367168 -0.039630172402091 -0.00116951897938139 0.0132581987947163 -0.00119745287251195 0.00524215226865928 -0.0488207739421464 0.00637292818763635 0.00252843745732406

TCGA-02-0087 -0.0396179654825083 -0.00647204732583324 0.0758099098270382 0.00533704561114188 -0.0812904243980039 -0.0317185150221628 -0.0183587204973008 -0.0777195404233798 -0.0177633291141445 -0.0612216087884439 0.00684190624350944 -0.0140522990583726

TCGA-14-1401 0.037381254519601 0.0642775935046416 -0.0490436642418919 -0.0311906257093231 0.0291973257331297 0.0222302070064848 0.0486674450620162 0.018139914220093 -0.000867319301415712 -0.024893928189373 -0.0162696996100348 0.0182088185405157

TCGA-12-3648 -0.0450173815649481 -0.0585039130223435 -0.0173476548858533 0.0711258195610542 -0.00799347413951558 0.0940561610137979 -0.0344502965282951 0.0176675788577289 -0.00949456126587061 0.0509294396098037 -0.00308707408582963 -0.0295627582769093

TCGA-08-0357 0.0331775322469822 -0.0158757765298107 0.0344194308288994 0.00291136169614108 -0.00913485445124386 -0.0387428086168438 -0.0277155041611661 0.0270123690959006 -0.0138860524518992 0.0125929370928701 -0.00563376348953558 -0.00717306912793416

TCGA-12-3653 -0.0324305316426415 -0.0869247575976055 0.0222541517258478 0.0333171913370025 -0.0105509679785261 0.167037777461202 -0.0122215536179513 -0.0464265749381029 -0.0401549147802226 0.0599509374049289 -0.0235058499012009 0.0238469784182913

TCGA-14-0867 -0.00625670926720592 -0.00445204593010816 0.0518577608966054 0.00601296742393674 -0.0481043645749867 -0.0312220511300494 -0.0143874128928935 0.0176329062609022 0.0723695107449656 0.0102704869223437 0.0502598934312784 0.000392495224581087

TCGA-06-2561 0.0583501055744013 0.0433947788555386 -0.0351824999950223 -0.0122794797061098 -0.00403990479472986 0.0180518663886147 0.0448264102399692 -0.04932785282373 -0.0413706006886882 -0.0050154133322854 0.0167311918262221 0.0223889241976965

TCGA-08-0348 0.0181552118673245 0.0286938194774385 0.0396200113605694 0.00981632115588854 -0.0343589405252188 -0.0463341796773195 -0.020848289179576 -0.00319863512076422 -0.0106391747250265 -0.0606510764504736 -0.0209706809489833 -0.0175021429223481

TCGA-02-0106 -0.0206373405269006 -0.0534915568430529 0.0405204182248055 -0.0266469076037102 -0.0710426157236534 0.0383495938838721 -0.00915727784018007 -0.0112585837418187 0.0755137798450876 0.0490818939082225 0.0637730586883709 -0.017910113013818

TCGA-12-0822 -0.0631274241229072 -0.00546568131848075 0.105460720273402 0.129267861062378 -0.0459700719684012 -0.014382948009368 0.0316610987604148 -0.0379374132699552 0.0162011200781563 0.0253573659146049 0.0480674182118119 -0.0142749111128007

TCGA-14-1456 0.0324317533860126 -0.00531373138281352 0.00926181656842046 0.016884346421733 0.0161431027971784 -0.0535158133891315 -0.00876783858708438 0.00930983585575109 -0.0239247423165528 -0.0840962626540312 -0.0758022998996641 0.00818806747105861

TCGA-12-0662 -0.0375671277229847 -0.0405120590508513 0.0412360310372038 0.0138562911300959 -0.0241027278647689 0.0122207060264737 0.0242408549044652 -0.0184239617138933 0.0108690025623615 0.0468750093027977 0.0280865897050433 -0.043867959595534

TCGA-14-1451 -0.0055791801683485 0.0483232709324267 -0.0418433408548424 0.00838723030154702 0.0338594085940259 0.0276903249490392 0.041133465993344 0.0461999428099659 0.00379921555636422 -0.0451349120642356 0.000717639484363118 -0.000823332696421921

TCGA-06-0644 -0.0273958287232676 -0.0218716853807398 0.059153560037841 -0.0286890459081336 -0.0308893589307796 -0.00797073993083938 0.0339321780063345 0.0391274285226297 0.137109938863761 0.0527640807696512 0.103331997697642 -0.012581919337015

TCGA-02-0258 0.0607694111728652 -0.000374054760406585 0.0374666511203226 0.0464679928114641 -0.0294820988917166 -0.0783788152183069 -0.108469270788915 -0.0594906460193718 -0.0569086589172987 -0.105217795348676 -0.0675749417314811 -0.0015898016312872

TCGA-06-0192 -0.0192967797183611 -0.0449688861619824 0.0462458162371421 -0.0011285277547977 0.0165858422018974 -0.00981874569253097 0.0523696945402067 0.0542166071568715 0.0515486291919396 0.0521498922691881 0.0844768715575306 0.00427488605791027

TCGA-12-1089 0.0804153447586981 0.0335583436170787 -0.0683902502993206 -0.0382364099325816 0.00999146937625261 -0.0277657882721774 -0.0211441021948595 -0.0691730226926865 -0.0755127014158486 -0.0359060442100615 -0.0784772481902058 -0.0713594029122191

TCGA-27-1833 -0.0337192585062264 -0.0789959568442612 -0.0630586719200055 0.0715974480304168 -0.0213687243859489 0.0628776894566885 -0.0217390295248681 -0.0729095441241843 -0.0641057947810734 0.0609927058433388 -0.0396447380216598 -0.0286591013647744

TCGA-12-0772 0.0606596547431944 -0.022929141566407 0.0240351116935985 -0.0339683840109134 -0.033737708153383 -0.00637408996180088 0.0116675627837561 0.0271960509724085 0.0368750285580476 0.0201180272795345 0.0462163872893141 0.0069224413232565

TCGA-08-0514 -0.0112457472180789 -0.0621249782730201 0.00516958493315266 -0.0154774938835278 0.0176095193070889 -0.0482805227816938 -0.0116082241667515 0.0132412602784133 0.0180086127551591 0.0426933709957889 0.0146067132950554 -0.00239639355610122

TCGA-06-0879 -0.047580822071093 -0.0576376770960957 0.0592880018695558 0.0174884124406745 -0.0114211411497592 -0.0362159512708953 -0.025440233454273 -0.00347914360747489 0.0107651450383152 0.0340721877593355 0.0299462069280934 0.0096940940847239

TCGA-12-1602 -0.0491144737443846 0.0438675019413441 -0.0184867134510172 0.0426007866407895 -0.00474935215684694 -0.0394405747820715 0.0274713079039953 -0.000432243083900073 -0.0163003071200453 -0.0413183959585582 -0.0245931987147221 -0.00627058020418854

TCGA-08-0389 0.0274069277871036 0.0114750438575311 0.0441230573536582 -0.0225972535284375 -0.0476201383467243 0.100768175752021 -0.014198181347595 -0.0558293579155183 -0.0410275211403272 -0.0235430481409274 -0.0255581954662179 0.00378366780808603

TCGA-06-1084 -0.0472905689563065 -0.00218840276555557 0.0392408313526691 0.0427372406545187 0.000530987791039451 0.0235376224291342 0.0960191613208149 0.0193907381535108 0.0573518475723382 0.0382743485628305 0.0497688326515601 -0.00666382399539904

TCGA-06-2558 0.0381088802654148 0.066217709191364 -0.0277162016285824 0.0299980670172672 0.0671837377313349 -0.047772109947724 0.00574422627736743 0.0614939058062093 -0.0141455575097379 -0.063205309081351 -0.0654204574496148 0.0292409286095436

TCGA-02-0039 -0.0408098421125351 0.020902452649754 0.0640105997773851 0.00458293984996707 -0.0758849829637352 0.0542660684017447 0.000808311559347638 -0.0461328915705621 0.00736537961779137 0.0266427125724346 0.0769462693886066 -0.0186004357111089

TCGA-19-1789 0.0290334969216473 -0.00243872573067983 -0.0601030013121356 0.115677888109171 0.0717150980342837 0.0137094287962254 -0.0567303759492932 -0.0663368574192275 -0.0790261479570219 0.0817280267091513 -0.021719033056188 -0.112880563630412

TCGA-32-2632 -0.145671364562857 -0.0599334889858394 0.00702221017936899 0.126679451213274 -0.0198307994449634 0.0269838754873904 -0.0439259926757783 -0.0772874931061856 -0.0408633064520513 0.0562454272892609 -0.0355819327298958 -0.105218911269567

TCGA-02-0104 -0.0123079891204506 0.0240949080411493 0.0590420572459945 0.055174648464566 -0.0853145938022138 -0.0984474493618226 -0.0980741612875969 -0.0749518800268275 -0.0482144476690199 -0.106633362572712 -0.103283617970606 -0.018916034243542

TCGA-26-1799 -0.0518389679615308 0.0970810037338755 -0.0591439353030228 0.0327924183713565 0.10387820951478 0.0264678128738181 0.00355133766172978 0.0663048371144338 -0.00342058059473182 0.0205824960158291 -0.0264410945768454 0.0866851769435232

TCGA-02-0026 0.0057783482926556 0.0253921175645537 0.0467318467552086 -0.0309593548760478 -0.0650525129101306 -0.0738826216213844 -0.0729962874482108 -0.0316508820772171 -0.0146526838693464 -0.0764611354326321 -0.0282296615317254 0.0191245076996623

TCGA-32-2615 -0.00774479527170379 -0.00178799574428745 -0.0338917533612342 -0.0196775831350743 -0.00655541439325861 0.0451996435273125 0.03597743621227 -0.0153415563544677 0.0157364728986379 0.0202174630525 0.037937997212589 -0.0406191721754043

TCGA-28-2514 -0.000174590368649628 0.0657193305586077 -0.0181183444794887 -0.0428371955982452 0.0161612717682625 0.00385574374023594 -0.0332426645179839 0.0257003581441745 -0.00773223142028762 -0.0071313685590555 -0.033125121872685 0.00207761370791551

TCGA-06-0686 0.0314265538733578 0.0623796159649751 0.00451427657846109 -0.0409551945922817 -0.00504293109214777 -0.0659592557515288 -0.0353268195246659 0.0284301421995457 -0.0124873395086708 -0.0689648992955414 -0.0405957667215697 -0.0111338066779041

TCGA-12-0688 -0.0566507019485729 -0.0545379269264873 0.0422567924324124 0.0352018505355745 -0.0161654748835886 -0.0491586590177682 -0.0467308322687462 0.00975777104994755 0.0225043281923414 0.0480840351287397 -0.0107135534264467 -0.0192588165971955

TCGA-02-0084 0.0748732396085952 0.0212468202527186 0.0512876380670932 0.00964828855126296 -0.0162974134672276 0.0079938950536247 0.050826770977265 -0.0285369593867838 -0.0118721305516054 -0.047442454002249 0.0411126290965762 0.10241104495025

TCGA-08-0520 -0.0635168341250127 -0.00979102908706868 0.0412888535045995 0.0382806800302453 0.0136776352094002 -0.035049046846799 -0.0211767624576219 0.00143398272152292 -0.0210078146100696 0.000807977831770378 -0.0069999711713796 -0.00192761775983666

TCGA-12-0773 -0.0759052601096637 -0.0546257084958063 0.0431746927010444 0.0342828143009304 -0.0437074524119228 0.0384284811685397 -0.00997486228955069 -0.0293238723822331 -0.0285933889512338 -0.0100929113922072 -0.00948702923373161 -0.0270883482444015

TCGA-02-0338 0.0604002886848401 0.00663760936284131 -0.0281973896824877 -0.0548851312726188 -0.0595973703532659 -0.0292834621083523 -0.0951611408557746 -0.020259026448784 -0.040199826122461 -0.079709003421306 -0.0797179106642605 0.0313628775540413

TCGA-02-2483 0.00372594775032537 -0.00456751729306167 -0.0212733818262286 0.0448715215434387 0.0504737198616136 -0.0392855371509943 0.0113890044398835 0.0419445557039034 -0.00135537208811571 -0.0814434724875109 -0.0534728889459267 -0.00119395408539578

TCGA-12-0653 0.0402382773882973 0.00653025557587803 0.0489635287760698 0.00106245028257893 -0.0546734285874853 -0.0371322166857447 -0.00514441668054818 -0.0279405256679002 -0.00141194687431156 -0.00555256783857157 0.0202648092932897 0.00202222434190556

TCGA-27-2523 -0.0429663489323756 -0.0436509362310777 -0.0173507841649682 0.0695190583930473 0.00341110902110975 -0.0383286808868643 -0.0339077904050442 0.00905413918184689 -0.0203894098701043 0.0317757734711355 -0.0211751882299148 -0.0273936426836828

TCGA-06-0155 0.0461162116115872 -0.0281767316161033 -0.0320219315748823 -0.0128373183129009 0.0805117991048271 0.00171267132711992 0.0418904759523655 0.138690369594632 0.173857726413931 0.0508650120625292 0.0253370952275322 -0.0285914342320809

TCGA-12-0769 -0.00726202098218793 -0.0406548646134145 0.0276930065915663 -0.00727811568012675 -0.0312591674761993 0.0386208689542359 -0.0210163588805026 -0.0169079728072624 -0.0237380816898853 0.0467216146481504 0.00828647087069727 -0.0144327879391838

TCGA-19-1786 0.00535882698705965 0.0224825640001816 -0.0993026986478718 -0.0293730712611098 0.0890026121570152 0.0127247424804145 -0.00140664344341144 0.0387959540487464 -0.00542359086180881 0.0589254287754019 -0.0229414903260716 -0.130903768864721

TCGA-02-0271 0.0487637959591571 -0.02597850911438 0.0122164998012206 -0.00625047454968248 -0.0407892955938205 -0.0317913786508111 -0.0421923857562521 -0.0171752849797288 0.0120838796457878 0.0207882678729174 0.0496380433153951 0.0270077863289851

TCGA-06-0881 -0.0284775455924316 0.0195742191031938 0.102830460695664 0.0955951170123988 0.0137579976683196 0.03917291609525 0.103000172431268 0.0645611908406891 0.136965819515353 0.037434230874168 0.0961385793837276 0.0122783126446426

TCGA-06-0194 -0.0116978849637216 -0.0347501240492959 0.0491755784931402 -0.00194506553607058 -0.00990181248599288 -0.0132343563229511 0.0491164053789192 0.0167702315509696 0.0206263419426604 0.0175602104740002 0.0593725890812247 -0.0233739744822561

TCGA-02-0330 0.0814540739318172 0.0190158979140434 0.053423322897253 -0.0472626596011628 -0.0509461059476622 0.0948814992729817 -0.0087765945926889 -0.045442336590615 -0.0178802488032393 -0.0128761432312575 0.0406558653228858 0.037776439267972

TCGA-12-0670 -0.0660407277367772 0.00859686054608757 0.0663961510314314 0.0744993566933432 -0.0315894140763424 -0.018434452188113 -0.0329282437743612 -0.0818405289049978 -0.0324946734256003 0.00292728102180366 -0.0220569293474896 -0.0220209910318173

TCGA-06-0146 0.0707969999872786 0.0158257354723473 0.0383174064623316 0.000208697243065348 -0.0200157538494948 -0.0632965462853437 -0.0332684345126789 -0.00699813786936125 -0.0258132054525936 -0.0881796428575163 -0.0390127595342503 0.00930372344002457

TCGA-14-1459 -0.0160859726096171 -0.0355657929136624 -0.0364009851808658 0.0361759509635357 -0.0564975444518124 -0.00497790671307435 -0.0135107451100663 -0.0889330505544228 -0.0570600593762591 0.0018600376012511 -0.0135792250858115 -0.0160320030413083

TCGA-06-0743 0.0375842285758127 -0.0289393912480163 0.0259592371493562 -0.0417867232233091 -0.00989491952542229 -0.0195529345410174 -0.0251306233677238 -0.0112371908795123 -0.0120396856588897 0.0153967919761374 -0.00536751982138845 0.019679824103866

TCGA-12-0619 -0.0341746424009707 -0.0278936752402678 0.0616383019810472 -0.0249402517727984 -0.0560436816668054 0.0135124697776246 0.00170246010176168 -0.021426017629266 0.0328391195727791 0.0228112726578397 0.0459303408302177 0.00494252481392551

TCGA-28-2513 -0.0501960986309297 -0.0404012322323396 -0.0126424693118015 0.0466670972257557 0.0670556418669535 0.0587429560355071 0.0933177299018993 0.052697145007939 0.0463958339849648 0.0345560882683483 0.0608025706835152 -0.0354177839478294

TCGA-27-1831 -0.117573750240466 -0.0442952259244358 0.0447005087257224 0.20952880135028 0.00847514232562226 0.0731209864337434 0.037801585700456 -0.0177460725795189 -0.000819582719471538 0.0519452418811464 0.0144783028758652 -0.0554101977170237

TCGA-14-2554 -0.074631276413274 -0.00541560528333144 0.0135850839590099 0.119345737244633 0.00993451819121595 -0.0141380994645058 0.0359076500397026 -0.0282743254146542 -0.0325845761375392 0.0282210058900269 0.0209163026606848 -0.0531018244747819

TCGA-12-0780 0.0211311892105878 0.00168457592162213 0.0204163530014317 3.54836733888273e-05 0.00353920769648672 -0.0194570917255663 -0.0516666809183197 0.0349830005042108 0.00547467424478352 0.0370753228225436 0.0248551789750337 0.0099012989679453

TCGA-06-0878 -0.000134681792332554 -0.0477008181476073 0.0497277486143791 -0.0165763165245187 -0.0382709005386673 0.000784889452759638 0.000662168169967936 0.0142841735580565 0.0299776767387947 0.0464874692485192 0.060364347361461 0.000834526177404252

TCGA-08-0352 -0.011435435808452 0.024694077676611 0.0410235540405203 -0.00312658702787061 -0.0311799360351072 0.0349432028624565 -0.00559456612324415 0.0225927254834756 0.0278035707793159 0.0449131239256113 0.0705317887111305 -0.0515333884159209

TCGA-12-1099 0.0300002471537044 0.0509042612908335 -0.0508344529690349 -0.00799074967047621 0.015272559472756 -0.0290477476868508 -0.00857541013648102 0.0100768974910887 -0.0316869053346109 -0.0659083692416282 -0.0549960524807143 0.0425347952002143

TCGA-06-0648 -0.01253746384654 0.0525580959439572 0.0361398221598283 -0.0100976950994993 -0.0666847977965607 -0.0361820683404346 -0.0587545696208767 -0.0354940923677877 -0.0213517827041463 -0.0683417971837434 -0.028216865252518 0.00712953733768642

TCGA-02-0289 0.0402315803247213 -0.0353518769910475 0.0274640415858289 0.00580205117271721 0.0162809856855687 0.00551602985807515 -0.0641084990543916 0.0274189307188827 0.0062434306338244 0.0360100714191641 0.0166253552488263 0.00686653934802953

TCGA-14-1037 0.01628912545891 0.0123883210870581 -0.0261970890979536 0.00122442109223486 0.0279542948450401 0.0315414999455896 0.0847629532387544 0.0275248082354954 0.0378188877595759 0.0173902853906254 0.047272142598658 0.0373696284858859

TCGA-14-0866 -0.0292883763893411 0.015599087825579 -0.0174088713164287 0.0337389624657988 0.0701075967139596 0.00917686129373754 0.0164915866676842 0.0594363453124133 -0.00480878250104517 0.035446400649823 -0.0267826523402206 -0.0515119484742309

TCGA-08-0518 0.0534536560620529 -0.0863935409032537 0.0118955305389726 -0.05151676543728 -0.0571547708734885 -0.0483494644278055 -0.0587408276167901 -0.0223005880010516 -0.00185563192424939 0.0514041729545926 0.0178369158192682 0.0136183689612968

TCGA-14-1396 0.0293606910166768 -0.0565990335067731 -0.024259367431744 -0.049578041550407 -0.0346047002688686 -0.00968704690375043 -0.0192615968793212 0.00588242707467383 0.0432805079064761 0.0312349259455832 0.0792501223280771 0.0274557281424373

TCGA-06-0177 0.00449108130320709 0.0467411929911527 0.0588199669663314 -0.00289303096274063 -0.0658518394613272 -0.0327838832649784 -0.0325678496968399 -0.0221317002781478 0.0120036001947948 -0.0588660436312055 -0.0323568155474825 0.00958050097676179

TCGA-06-2557 0.0135347614399948 0.0109183104126692 -0.0018093482358076 -0.0145435094281299 0.0126127305248677 0.0756949243361001 0.0758856949930768 -0.0541002506214747 -0.024297085192032 0.00269252159510644 -0.0295047842708607 0.0190163059907033

TCGA-02-0269 0.025641528895487 -0.0500359366274231 0.042966986534577 0.0103093831366408 0.00231653819844362 -0.0214497251006562 -0.0948639864855993 -0.00107530796332052 -0.00836916420885516 0.0447189227916036 -0.014539215716474 0.0238693086194425

TCGA-08-0344 0.0149819178751824 0.0257982826979544 0.0533908467238313 -0.0248643473820301 -0.0264412568571222 -0.0903397886944373 -0.103995557282651 -0.0152034351490364 -0.0176714320709809 -0.0816860027354027 -0.0672188046841686 -0.0379814934828345

TCGA-27-2526 -0.0203282107710117 -0.0627173139154092 0.0143266680751155 0.194990830066739 0.000412492923081175 0.0618446195082608 0.0241105805583261 -0.0477109014357723 -0.0507336060509702 0.0557739983721658 -0.00118376501188331 0.0056226784374206

TCGA-08-0351 -0.0261460180090866 0.0584659145575164 0.0619514425298865 0.0193573446281218 -0.0674734694413115 -0.0301884666487143 -0.0178096620746873 -0.0953816816414855 -0.0454897814604882 -0.0602523843933102 -0.0137133560112614 -0.0393411073242741

TCGA-02-0430 0.0133872405468103 -0.0663283556229766 0.0201095886292145 -0.0265508689797853 -0.0111332922951363 0.0216982475558379 -0.0322005760161874 0.0175160109605766 -0.00820029652598052 0.0130208602449737 0.00193046831095744 -0.00122282534182385

TCGA-12-1096 0.0644403695714036 0.0505165700304228 -0.0299461431335034 -0.0350281378234689 0.0424054489768249 0.0404954582274174 0.098037373318468 0.055469224194133 0.0120319747034503 -0.0354420694868379 0.0156710846163794 0.0134858183326938

TCGA-06-0646 -0.00210431568861307 -0.00820735558781395 0.0366505616454792 -0.0312525942251435 -0.0453395871216688 0.00426551711496844 0.00317457999918714 0.0160197653525284 -0.00150423776172736 -0.029860317907908 0.0140665823839298 -0.00129519407409402

TCGA-19-2629 0.030871965413639 0.0224844783707833 -0.0278112112368901 -0.011970688025443 0.000526475984479749 0.0619093077818258 0.0292859029261825 -0.0446850818739228 -0.0547166984354288 -0.0864699633218128 -0.049785456752396 0.00499917104555574

TCGA-28-2509 -0.0192188195329039 -0.0593592484892687 -0.0172821924117425 0.0733009431291256 -0.0620805028967493 -0.0359630774752215 0.0118018252454833 -0.0481563780976552 -0.0242260623685827 0.0359150725851476 0.0221011880656847 -0.00750642755285981

TCGA-02-2466 -0.00293849206632373 -0.0252550449853487 -0.0426028427529086 0.0119338798186394 -0.0144980409699195 -0.0882775216977492 -0.0551676911302198 -0.00657851917584868 -0.00953361241748992 -0.0601833039741946 -0.0862881229208829 -0.0187670166830936

TCGA-12-0654 0.0589137982838093 -0.0431089781501043 0.0467419335769513 -0.0189721142175604 -0.0446821619429301 -0.0393447709575552 0.00339524998992554 0.0164850332957545 0.0474507641026321 0.0358401467802594 0.0591945595252698 -0.0206481972130995

TCGA-12-1600 -0.056015362861957 -0.040018359013859 -0.0509055795412909 0.00317862863409715 0.104684839676066 0.00580937648187437 -0.000489412636682103 0.052669728596383 -0.0272611263886635 0.046054157775662 -0.0366011636737041 -0.088151991796646

TCGA-02-0321 -0.0300133537430384 0.0134380087269127 0.0696867515936245 0.0176788238931564 -0.0805021607381497 -0.031279299613963 -0.0463299825121297 -0.0564837874990808 0.00523191532217056 -0.0151508828650453 0.0448635606626935 0.00779219793944631

TCGA-16-0849 0.0466882896788452 0.0328310213334987 0.0413585061054489 -0.00926791207471644 -0.0117690134951523 -0.0256163183590995 -0.0308075339959036 -0.0172209243009843 -0.0174727214622088 -0.0779129478730717 -0.0321887655130214 0.0223495749958478

TCGA-32-1986 0.0832344736777123 -0.0238219457844968 -0.031706149306843 -0.0167298090181606 0.0652521478211551 -0.0327856961311051 0.0294183863476063 0.120752558520375 0.101896743785934 0.0559804287956493 0.0525618492705495 0.0266958236272405

TCGA-28-1756 0.0593181987831331 0.114564725587256 -0.0417980468683889 -0.0258450893519661 0.00386354474332384 -0.0487216600926904 -0.0240033708552971 -0.0909024103673382 -0.0768911846021268 -0.126597102389838 -0.108575879901922 0.0324733398187144

TCGA-16-1460 0.0511859656289538 0.0393493051803783 -0.0405188249338784 -0.0511260297182713 0.0395476381638407 0.000452044781059583 0.01966145154662 0.0244151452886864 -0.0166068810071096 -0.0856421693833579 -0.0380393128176767 0.00998549076162384

TCGA-08-0353 0.0259334308835588 -0.0426269855024992 0.0162732556881551 -0.0212621635818959 -0.0147001126413504 0.00451451369986778 -0.0428344813020941 0.00953754488795853 0.00410221716335106 -0.00286169090336706 -0.0275306408047111 0.00578411373736216

TCGA-14-1034 0.0236222551439669 -0.0340090958613909 -0.0175717669639623 -0.00186753394545378 0.0205616760311696 0.0172680897260337 0.0444825246351937 0.00934515673334229 0.00619817482709382 0.0497067712005637 0.0425219669782033 -0.0274831406234878

TCGA-14-0789 -0.00366575290425432 -0.0438159280518578 0.0645686628759453 -0.0174054560794711 -0.053162508238921 0.017423605721748 0.0228373941488436 -0.00935754549015222 0.0407271901517279 0.0437125815329022 0.0769111261969281 0.0141095949348819

TCGA-27-2518 -0.024251105440555 0.0992041720460861 -0.0257194712272246 0.0774703163856399 0.0441213981654586 0.0297449526648575 0.00093333675088677 -0.0277188983849877 -0.0333038315331333 0.00771008336662778 -0.0200893229941318 0.0395184650592274

TCGA-02-0059 -0.0330472528991648 -0.020853429035186 0.0775308685278356 -0.0170988681380128 -0.047867494349568 -0.00895361946963042 0.00886246892373895 0.0154658312205386 0.0634315809416243 0.0243406169482798 0.0522258851416805 -0.0289795140883073

TCGA-06-0414 -0.00869427211519535 0.0506010617576373 0.0211680894583231 -0.0133975553878815 -0.0408250623561252 -0.0925658852168422 -0.0424056732325567 -0.0191950930613938 -0.0372246461080395 -0.0739814311349957 -0.0459421235796105 -0.0189272792085744

TCGA-14-0783 -0.106181647282347 -0.0647832220191173 -0.0193773439159778 0.108823958199759 0.0567833744813447 0.0156662363575006 0.0561376631566271 0.0371295899018632 0.0139005562147861 0.0716626512900477 0.0239724948298744 -0.0180980807964884

TCGA-02-2485 -0.0126050034098541 0.00395569617099612 -0.0359836842807752 -0.00623913736757891 0.0480752074844497 0.0732183899685529 -0.0142879415057783 -0.0036712522359026 -0.040301856418579 -0.0132446930396433 -0.0492384514725533 0.0416475000626712

TCGA-14-1829 -0.142915203614058 -0.0266272245707882 -0.00819520176216613 0.114651272714852 0.0293545219673903 0.0206591521078291 0.0612053590426802 -0.0203198401864955 -0.016715647647153 0.0138316379665263 -0.0158202353220226 -0.0351526324923401

TCGA-08-0355 -0.0026854316121168 -0.0224807409607028 0.0633326119071132 -0.0245386640424141 -0.0329873957820954 0.0332652529457549 -0.0227999461002504 -0.0386712973218796 0.00993195381387778 0.0330041284911431 0.00903264419367565 0.0406858597926615

TCGA-14-0813 0.00419789505650825 0.0149743222595263 0.0621196636374709 0.00877704827189552 -0.0953357922045352 -0.0557963307843139 -0.0679772321287723 -0.0650446090537977 0.0113275426892676 -0.0101233012409956 0.0136282194142871 0.0185681967391784

TCGA-06-0127 0.0549577890657835 0.0601160304864123 0.0196697349726736 -0.0356929347800435 -0.0164560330044272 0.0429566127000078 0.000224073669194325 -0.0412606471399508 -0.0315931348615984 0.0149769139641625 0.00374699946191685 0.0315182291902879

TCGA-15-0742 0.0353842589375448 -0.0537878172093429 -0.0021307749524408 -0.0603865359895468 -0.0213038290291509 -0.0238422999624247 -0.0661046823179835 0.0102366991234256 -0.0252102546488715 0.0164608913022614 -0.0278064026000371 -0.0267115681530679

TCGA-12-0707 -0.0650535396284716 -0.00963081425764876 0.0551225653474662 0.0529225775118765 0.00495176844020704 0.0469556658946167 -0.0347796287051948 0.00376321123776263 -0.02167593733827 0.0397423509132755 0.0144825470742948 0.0752428052427206

TCGA-06-0216 0.00361144727474743 -0.0317554395538701 0.0175408918897286 -0.0329945866577329 -0.0483468066043653 -0.0170875967863264 -0.0292669995981169 -0.0216771783507332 -0.0276046886742313 0.00386108450727401 -0.0181969513609821 -0.00722378882110178

TCGA-08-0509 0.0372284360277135 0.0142420109542207 0.045240382691082 -0.0463942270885635 0.0182569321570313 0.0297596357180083 0.059888346036425 0.0333305731065051 0.0194367192821583 0.0167692311608162 0.045526980651375 -0.0119821058320416

TCGA-08-0529 0.0111582408974172 -0.0437664867252149 0.022030572652011 -0.0210526053803208 -0.0349455087052628 0.000605065498324489 -0.0306632100162222 9.32506686010519e-05 -0.0147906213372516 0.0431886435599375 0.00822124250472327 0.0035769150382715

TCGA-12-0826 -0.031238430249568 0.00516017290002491 0.0654687146319702 0.0327474818114308 0.00302452309983361 -0.0455722337454203 -0.0455150078649391 0.00434144802995385 0.00997630777334536 0.0382376087835293 -0.0165558028361097 0.012745689095396

TCGA-02-0326 0.0584388583846912 0.0380524820779687 0.087257231041079 0.0928258865806273 -0.0259541646270406 -0.0517812533218047 -0.0215221076906928 -0.0102043696156818 0.0193559565323507 0.0209936300219018 0.0715876714348593 0.0610597745578309

TCGA-02-0051 -0.0199585202770424 -0.0313851723743213 0.0657294821391909 -0.0120053795022243 -0.10612723443888 0.0141059650785662 -0.00629122270037797 -0.031607580825432 0.0848087485547297 0.0302246984409296 0.0591281127839174 -0.00820634844184949

TCGA-19-0964 0.0182888651450058 -0.0675559874070882 -0.0453787496455585 0.0448942737268351 0.0108235254119847 -0.0283180262523003 -0.0391300090272577 -0.0331217775340762 -0.03917751278633 0.01990420181912 0.00310035244946554 -0.0881988850822513

TCGA-16-1056 0.0631711301047415 -0.0139755898294822 -0.0590755991806802 -0.0959185037216562 0.0747755531280075 -0.0242142312335882 0.0111119358665198 0.0918110497335714 0.0269913793989556 0.012426399829252 -0.0589198586741972 0.0208979207482653

TCGA-19-2623 0.0157514606340727 -0.014721803973741 0.0273259961200722 -0.0346013687070374 -0.0268883176256942 0.0543349531086427 0.00704315943491276 0.0393095301833532 0.0295357092311078 -0.00503018985414433 0.0136531427047023 -0.00154022185908975

TCGA-02-0281 0.0868656982777105 -0.0161561955320362 0.00787121550892585 -0.0533954258558482 -0.0618439364568177 -0.103687032244107 -0.11872264555615 -0.00908436282687284 -0.0176195237443544 -0.0773103559153903 -0.0664630206583362 0.0039612388170297

TCGA-19-1386 0.0397715489063715 -0.0597656874556378 -0.112629303526206 -0.0768682991999826 0.063737031663226 0.0587161508780635 -0.0233067121239164 -0.0143371773010282 -0.0427558921666894 0.0795854360918528 -0.0182246435066245 0.0617367766210889

TCGA-08-0245 0.025124652570347 -0.0203636227874029 0.022511036938477 -0.0365696235696699 -0.0194952694549016 0.0213510497835026 -0.0521207217831027 0.016334907105638 -0.0178877582889974 -0.0580314291711172 -0.0549320182977819 -0.000853335214614837

TCGA-08-0521 -0.0298655101940832 0.0118374840353174 0.0419439138629087 -0.0199956665130384 -0.0506098376157515 -0.0271777206822569 -0.0207022928864519 -0.0336753187796356 0.0164480679774192 0.0269382573301685 0.0623473793750644 -0.000272576842745206

TCGA-08-0531 0.000167204970507265 -0.0674507462570808 0.0254863022038808 0.00541413432975773 -0.0253856962508551 -0.0478714795384414 -0.0487988146862271 -0.0487467326664815 -0.0414080315888263 0.0409350435118147 -0.00810257783423648 -0.00637272988815298

TCGA-06-0164 -0.0573253051068462 0.0104401635899394 0.0497578148744005 0.0114483448734116 0.0392261405215735 0.000654023466761985 0.0446092422767504 0.0908120497686879 0.0950216264782811 0.0387674860944687 0.0585130848362687 -0.00672983388588866

TCGA-06-0238 -0.00290945340710345 0.0630229451174228 0.060951384574188 0.0163503382738627 -0.0567862816358495 0.0123726380521798 -0.00358345228295083 -0.0412936434170724 -0.0183358928968115 -0.0629068817706187 -0.0322467381694994 0.0177833783774484

TCGA-08-0346 0.00402505687941262 -0.0276052125751882 0.0670263471594653 -0.0186646198100571 -0.0544467535997036 -0.0230801379073438 0.00554539917839339 -0.0030754271801868 0.0201233165570646 0.0129382186842968 0.072591647410646 0.0025668319318584

TCGA-28-1750 0.0554715386178245 -0.0113401336948134 -0.059231577681349 -0.026424153641267 0.00640136925777789 -0.0221882791689642 0.0366208808627034 0.00830931112231581 0.00294615589063427 -0.00878530673049879 0.0297170309880368 -0.0414420623586142

TCGA-06-0175 -0.0268646588928616 0.0120137990332933 0.0334586183456351 -0.036288962440856 -0.0619407888592481 -0.0569046956045889 -5.51713496981702e-05 -0.0208917671465902 -0.00230189654532381 0.0230129530577936 0.0210534848816497 -0.0114031207361856

TCGA-26-1443 0.0627677681916526 -0.0712381638596213 -0.0437679692000475 0.0415006075341063 0.028879238866282 -0.0319724097500197 -0.0640096308212176 0.0351639202757812 0.017685033215895 0.068857399695963 0.0285561988808664 -0.0188947485662948

TCGA-06-0410 -0.0439720252251561 0.013375161971717 0.00609554453805584 -0.00918487075540999 -0.0337374749650221 -0.0107988554660875 -0.0387357796753899 -0.022623335189328 -0.0299189878397287 -0.0639251390878637 -0.0513733811258637 0.0161456569346256

TCGA-02-0025 -0.00466151138680282 -0.011564994428535 0.103608723156248 0.0317364603402245 -0.0798307853385336 0.0311899717551092 0.045309271788642 -0.0177204791311114 0.089511678220182 0.0164984835611088 0.0800394653451334 -0.0142494948774907

TCGA-26-1440 0.0606964963941487 -0.0261772019633733 -0.0483653998137383 -0.00250794082352181 0.0372296818222388 -0.0155606754910007 0.00913194208299255 0.0155859398438196 -0.00337332533399953 0.0311536330767776 0.0082231689637337 -0.00762664271809927

TCGA-32-1970 0.0424528366591616 0.012099270509596 -0.0416568856440212 -0.0161977638070725 0.0231199086873501 0.053876416783447 -0.0208319497062647 -0.0391324890965984 -0.0471371180317482 0.00796626624766444 -0.062463482183393 -0.0392685888407195

TCGA-08-0375 -0.0227632289931855 -0.0563991284266756 0.0596858566851074 0.00713295196343444 -0.0599583302701258 -0.0300863111935868 -0.0591617334433195 -0.0206524613142124 -0.00929916554955525 0.00931327959240392 0.00269581987297222 -0.0127005538140708

TCGA-16-0848 -0.00656291097462477 -0.00544125589332817 0.088224481291814 0.0833566531084084 -0.023207439688958 -0.00916582603000661 -0.0176400812453886 0.0109112615922325 0.0204322737748875 -0.0120445941532797 0.029376217564208 0.0178575553639376

TCGA-19-1389 0.0387492904684259 0.0263678704098428 -0.0483404193564094 -0.0290628874713681 0.0465355984818542 0.0230291013842049 0.0888279396277766 0.0812652465140575 0.0826219392838898 0.0615181306394258 0.102048911514228 0.0207594538202425

TCGA-06-2566 -0.0231325083067227 0.0184061602301391 -0.0326236801581674 -0.0202597320947317 0.0387158738147214 0.0253105118824515 0.021368105285215 -0.0318009441439022 -0.0231451175626022 0.0245841958395949 0.0149384002013346 0.0101403970807801

TCGA-12-0620 -0.0353367642004307 -0.0318935935304032 0.0646604666556874 0.00784324227189528 -0.0572225210970644 -0.027780609109042 0.00299645178217064 0.00151585212181143 0.0133450018464053 0.041144706940702 0.0354913754903842 0.0196848539500924

TCGA-06-0394 0.0262562907915699 -0.0496039783030329 0.0237099481496351 -0.049263851658535 -0.0178406198167447 0.0174317847202725 -0.00716208836509301 0.035402529322067 0.0335436956287998 0.0395019730091776 0.0470791159218533 -0.00774576960129893

TCGA-08-0246 -0.0165029566487679 -0.030557071037229 0.0636669350908961 -0.00911804481057324 -0.0647719843108444 0.0732835233916968 -0.0176224655810123 -0.0486694744423158 -0.00321827512039148 0.0366229763803699 0.0285636081003963 -0.0671290224940961

TCGA-19-1392 -0.0229883770240375 -0.0387797583153049 -0.0384603135901896 0.0414557035897575 -0.0667187323992399 -0.0490499457501861 -0.0585352392518673 -0.0705613516353982 -0.0354913050842274 -0.0334743410199921 -0.0468016520797804 -0.0112125895507256

TCGA-06-0149 0.0216904259776152 -0.027450231857366 0.0469204126124367 -0.0082433036854819 -0.0351337515839522 0.0730908173676952 0.0164220306546287 -0.0295295346889152 0.00206286845428757 0.0192200063251652 0.0274017011117591 -0.0162922512111147

TCGA-08-0354 -0.0602402032741417 -0.047071306672327 0.0731760276440488 0.0306750546877787 -0.0674774245976549 -0.0137806066627475 -0.025674113629953 -0.0397100199134925 -0.0119033219002666 0.045709010626864 0.0254746465668548 -0.00134629571610322

TCGA-02-0339 0.0623704435135107 0.0524500161424092 0.00140971121071236 -0.0239751366997142 0.000979135122719983 -0.00816145919894741 -0.105256011149386 0.0295464111721037 -0.0267689268957841 -0.0886207924078442 -0.0794976770678123 0.0295848379492235

TCGA-06-2563 -0.0303846085132137 -0.00685105933476754 -0.0230479263458584 -0.0275819634753512 0.052481407893459 0.0634449431316976 -0.0142456304232976 0.0380204750184303 -0.00395757900790225 0.0535552929828528 -0.0318615973328387 -0.0243912680769904

TCGA-02-0079 0.0368726947888556 0.049911447534388 0.0522682859685844 -0.0261464858815263 -0.0565731773111851 0.0845701614263049 0.0373672870405287 -0.0202731743213031 0.0161967826020242 0.011531950858718 0.0830504857449463 0.034303886061927

TCGA-32-2634 -0.0167346797863049 0.0589855652349008 -0.0347652631077579 -0.0434573623287293 -0.00191280638950899 -0.0546976043189914 -0.034957295556111 0.0156362181475007 -0.035211055169332 -0.0443458616739669 -0.0601349584353842 -0.0226296593977759

TCGA-16-1062 0.0306823973298969 -0.0828726994380651 -0.0492591332372395 -0.0164080785415776 0.0209098027238285 -0.00275421628893541 -0.0407527380372364 0.0315309369679956 0.0305076435804716 0.0565309720691008 0.0085382999205523 0.0111680716196951

TCGA-16-0846 0.0350716547865794 0.0590122821609607 0.0560593103359936 -0.00680187011488668 -0.0486230063729346 0.118377811932878 0.00250929520630858 -0.0838197374555849 -0.0350288587831124 -0.0462156171017887 0.00707177870751956 0.0193296857370593

TCGA-12-1098 -0.0768202008825775 -0.0399026290542093 0.00123698190810252 0.123201322479022 -0.0331221096804183 -0.0258594826925743 -0.0140399489212064 -0.078547957745764 -0.0481134212875149 0.0192873695198889 0.00766217399687727 -0.00629395889226106

TCGA-12-0829 0.0277894150460276 0.0182965648806435 0.0725018172901866 -0.0238131685135883 -0.0392862772853276 0.00525136601535604 0.0047336196023878 0.000355083280656364 0.0245137946457798 0.00918781530318367 0.042980177782948 0.0291214007393127

TCGA-06-2562 0.00890310839294094 0.0206653371914089 -0.0424404306361426 -0.0306376216658433 0.0422907453536977 -0.032208703052195 0.0194677541538773 0.0614882933102381 0.0290753720091134 0.0360307876246754 0.057576142171969 -0.0147958182505196

TCGA-12-3644 -0.185548474034173 0.00197609695203322 0.0675158392020262 0.0955671784346679 -0.0502539997715155 -0.0319395567008079 0.00577885424710466 -0.0289082270035014 -0.00658833332682909 -0.0439744768162228 -0.0358830639624042 -0.0819028557371538

TCGA-16-1047 0.0404888653163856 -0.0529364653818783 -0.0564851031253619 -0.0307338085116659 0.0273569456887498 0.0246684825334634 -0.0237958609856819 0.033058167531162 -0.00861809365005708 0.0310956319083948 -0.0019759446295624 0.0131672353967521

TCGA-19-1390 0.0616126946405479 0.0122292769602954 -0.0575278259255205 -0.0628947643769667 -0.0530503344776443 -0.0597194941318779 -0.0651512291745523 -0.0449877453641903 -0.0608200087919544 -0.10173846104242 -0.103720560101857 0.0555271689570565

TCGA-06-1805 0.101208326733625 0.0243998933018026 -0.0218196667320142 -0.0538400584068481 0.053438876662927 0.0481777975491061 0.0673526820851715 0.0421063947271149 0.0252962299911257 -0.0795266133122315 -0.0435711169009133 0.0212552227629683

TCGA-08-0386 -0.0258253642654816 0.00374541624928582 0.0257959351677592 -0.0459247995445973 -0.0218243769087842 -0.0297246939695452 -0.0558244825814351 -0.0101313736729955 -0.0111630892943803 -0.00714180033176363 -0.0137358032523552 0.0192567209371061

TCGA-41-2575 -0.0422259348218825 0.00289820064260397 -0.048698771412024 -0.0185822221719807 -0.051332758521847 -0.049725041487769 -0.0267749490343681 -0.069499530136853 -0.0694950089112171 -0.0609253513473053 -0.0926509734437984 0.0228262156419853

TCGA-12-0778 0.0398225224264623 -0.0247327637964356 0.03465562558709 -0.0348643989169942 0.0319885407253166 0.0120525605419044 0.00909180624670654 0.0760180397840668 0.0399794463550479 0.0271413029082121 0.05203905327157 -0.00893283733757844

TCGA-12-3652 -0.0339250537008018 -0.00567883606608994 0.00489981643821422 0.0410743834358203 0.0174351550007869 0.0694809084405623 0.0636229146562784 -0.0438749983566287 -0.024951331221667 0.045612259534107 0.000987628718707313 -0.0743605000302733

TCGA-06-0882 -0.0322885369635343 0.0415574388957944 0.0503647986239291 0.0116583189455912 -0.022277346626062 0.0116160495595756 0.0172638542914476 -0.0327247163725516 -0.0112260577316918 0.0216813086306924 0.0500580825667123 -0.00937231474470977

TCGA-16-0850 0.0226478159588235 -0.0120262552034831 0.0521010466848574 0.00634693178772401 -0.0534865551184524 -0.121366325701348 -0.122474332517153 -0.0309065786274965 -0.0265334205750004 -0.0871793549842011 -0.0633465254427537 0.00562142237340362

TCGA-08-0385 0.0300892756924498 0.00114889094735201 0.0576593523617841 -0.00115283389265929 -0.0438850481745339 -0.0702865242720717 -0.0649062444181358 -0.042330047036521 -0.0173534637398536 -0.0631653650000338 -0.0296076286255739 -0.00329732947506154

TCGA-26-5136 0.0588936920123386 -0.0118554463630936 -0.0147571327410043 -0.0218596800891968 0.0310334980027507 0.0975236119208437 0.0385234466650284 0.0212684091451489 -0.0251451872730578 -0.0109476122344817 0.0187051658291194 -0.0405830807350265

TCGA-28-5207 0.00785614504340927 0.103673345164831 -0.0749893645572216 -0.053822334966931 0.127814093840958 0.00396870290572672 0.0348842014765204 -0.00383977550191914 -0.0283677925283623 -0.0331689553347972 -0.0798258332100154 0.0860987247847732

TCGA-06-5418 -0.00179465328313498 -0.064225187490752 -0.0334228491056069 0.00245218266266762 -0.021141449538523 -0.0256522596717732 0.0240725755842051 -0.0140086216795951 -0.00564134145069555 0.0421150527291625 0.0287830720628117 0.0354984867038838

TCGA-76-4932 -0.0197828509118196 -0.0685144216100135 -0.0222118497996089 0.0310217464295799 0.00855418770031807 -0.0125098760633772 -0.0153351851541272 -0.0055749815501556 -0.0280881904873832 0.0489173433889518 -0.0112351664068083 0.0129841711102087

TCGA-12-5295 -0.0892102534292822 -0.0611907297533156 0.00421985498411878 0.0777955939338143 0.0310208650401312 0.0157560343763233 0.0482649155001629 -0.0547107879253734 -0.0550338758148405 0.0355624505936337 -0.019567974154072 -0.0311923529758089

TCGA-76-4934 -0.0279874094959248 0.0327009967400427 -0.0125231741771388 0.0252541934547352 -0.014941120239489 -0.00876342143591049 0.0111369318655836 -0.0246258599312667 -0.043622513985475 -0.0532906524015064 -0.0438807961076639 -0.00792462775091053

TCGA-28-5216 0.0257721258472521 0.0432976072444536 -0.0571011568774412 -0.0171077115476612 0.000898399463680969 -0.0305192679169487 0.0152403506391509 -0.00182569200677033 0.0148594454583732 -0.0553096632895228 -0.010082657169934 -0.061798548947661

TCGA-76-4929 0.0348917048680812 0.00592057262099001 -0.0592827294022828 -0.035759039896083 -0.00318673918319634 0.00861343082256259 0.0141956451391245 -0.0301156523690547 -0.0546129750092704 -0.062147950819603 -0.0335193068977313 0.153844324927057

TCGA-12-5299 -0.0266196486776924 -0.0290606465585396 -0.0551552564292923 0.0394014364268739 -0.0069027592443303 0.0597521338691459 0.0292175305162805 -0.0100678101665859 -0.0137940568393811 0.0421570126819066 0.0216573140479428 0.0131686730114507

TCGA-26-5133 -0.0118093818809727 -0.0241705686516488 -0.0288351276014422 -0.0229152392913929 -0.00122759932693184 -0.0532375323023934 -0.0536284639930789 -0.0201370558654642 -0.0302001098846345 -0.0628174664202202 -0.0465384360280934 -0.00626429275844108

TCGA-26-5139 0.0479401501887299 0.0161417330279879 -0.0661920020448855 -0.0376327395866202 0.0294006864931655 0.00863381731345119 0.0193297590733805 0.039795180763659 0.00759719776612052 0.0439175165991147 0.0304100667925148 -0.00896531732121875

TCGA-76-4935 0.0117853245092433 0.038460943758279 -0.0331349276184796 -0.00720926001104049 0.018089191434401 -0.0630533415281733 -0.0405205695169009 0.00340344694485255 -0.0389900594563594 -0.0626878228796156 -0.0713502437187332 0.0253428062158169

TCGA-06-5414 -0.0454244458343884 -0.0628044832529673 -0.00904724573064398 0.0305685321394968 0.0266136324234126 0.0606879198319776 0.0343796532723566 0.0189638041162278 -0.0097128414290788 0.063529826966669 0.0314016208158164 0.0196129153588063

TCGA-06-5416 -0.0831684870060303 0.0102250389093119 -0.0100395928046779 0.016234708355269 -0.0282883493082956 -0.0498557078282774 -0.0703984765618335 -0.0291540905067192 -0.0197657939370601 -0.087849352852928 -0.0691706668641102 0.0318295731958766

TCGA-28-5204 -0.00399834395430509 -0.0551616588460089 -0.0667885734370025 0.00237656976030521 0.0801654479253327 0.119379269633431 0.0212252655253573 0.0458793495408959 -0.0176432580794158 0.04702513805033 -0.0237788161916502 0.0845512113081732

TCGA-28-5220 -0.0685841746222827 -0.0331806198072533 -0.00962586626729678 0.0328396334158914 -0.0323552827878225 0.0565665446950436 0.0142369255736527 -0.104225152508921 -0.0746232892011487 -0.00908977972378178 -0.0772442437771747 0.0118048012854939

TCGA-76-4926 -0.00697982480200734 0.0597129538885464 -0.0410156727228632 -0.00738176697893421 -0.0162211674048518 0.00356847302799582 -0.00779714838994587 -0.0639184655710404 -0.0626294861834108 0.0110733240292076 -0.00238342337094233 -0.0521101774985734

TCGA-26-5132 -0.0093385609358641 -0.0265913607473939 -0.0127818696546981 -0.0162569562162517 0.0254605231852576 0.0406227206408517 -0.00874264011148837 0.0321046598989052 -0.0119018770232629 0.0333209155451912 0.00789445676503627 -0.0554272529518834

TCGA-76-4925 0.0232089082888516 -0.0694644368400031 -0.0339840504856822 -0.027348342837166 -0.0429807330337609 -0.0550406557539435 -0.0611580493725306 -0.00831663976106629 -0.0181518396660619 0.046701662377231 -0.0198757331165481 -0.00434133591423186

TCGA-28-5208 -0.064993625558941 -0.0285546932797405 -0.0249523963790456 -0.00620830211089967 0.036761904852455 0.00650982514976696 0.017041856319685 0.0118490851857037 -0.00789950605983262 0.0310113303017044 -0.00924284427403559 0.000392352098587166

TCGA-32-5222 -0.130854686629446 -0.0241808373238173 -0.00383871429354836 0.0592371466749472 0.0258694133065406 0.0278769464156184 0.0352139136323558 -0.0156874171212399 -0.039407200162738 0.0320098654090253 -0.0329903119394575 0.0213087205927162

TCGA-06-5415 -0.0319306850658139 0.0374789526694439 -0.0248291997476331 -0.00381335477399198 0.0208500348085397 0.00819102283583559 -0.0190338447183526 -0.0189346850233417 -0.0272408246982941 0.0114118029067457 -0.0381508164233116 -0.0366509473017088

TCGA-28-5215 0.0408985636706446 0.0384226324565873 -0.0509599409466794 -0.0253257854476597 0.0330116488074416 -0.0113308153247931 0.0472691630162187 0.0315311651480078 0.0495862077876285 -0.0840006321678073 -0.021177814217268 -0.0335298823592965

TCGA-28-5213 -0.106168250996608 -0.0363079244442658 0.0352942467426897 0.0895964010929798 1.16400377322121e-06 0.0271881211940373 0.0998322327922277 -0.00295037843029435 0.0308425085551519 0.026111951608981 0.0413311861559973 -0.0431546060814734

TCGA-28-5209 -0.017701370480343 -0.0602415129476551 -0.0462015284835576 -0.0111030163821732 0.0289766582163965 0.0244122802496602 -0.0289447205150671 0.0481595931253361 0.0341419318811939 0.0610731265970609 -0.022444037861811 0.0288046558341225

TCGA-76-4931 0.0391126648758441 0.00901158613018021 -0.0796282336015903 -0.039150742127127 0.0133963425847305 -0.031704104056673 -0.0411473590966878 -0.0151600328012347 -0.0555430834139313 0.00566218456679682 -0.0214341077040508 -0.00957773567704291

TCGA-28-5214 -0.032133178676386 0.0265034726284749 -0.00864278730527455 0.00910128970865404 0.100192261684943 2.78623566771875e-05 0.0464038592915019 0.109001208065225 0.057259847015742 0.024973510921521 0.0313056166933828 -0.00109352454292199

TCGA-26-5135 0.0325321530705375 0.0222594597935694 -0.0484379331488974 -0.0115622892091563 0.0935613259613464 -0.00144158894304686 0.0162403960906992 0.11170562086203 0.0230354286547777 -0.0380384875083261 0.00480151933873506 0.0297007633239098

TCGA-28-5219 0.0321625439428748 0.0557415048643725 -0.0669861789403224 -0.0520516610668281 0.0871595117711389 -0.0135969104465921 0.0166717007203419 0.103894867780072 0.00667129151083263 -0.0441134757438524 -0.0368724168826015 -0.0134498488393879

TCGA-76-4927 0.0075135696537865 0.014897669982221 -0.0453927849598946 -0.0224076183575689 0.0515157457919203 -0.000330362493904501 0.0397653978382304 0.07309379333412 0.0331029164585599 0.0128831214860769 0.00756814842226262 0.00834777628457661

TCGA-06-5417 0.0151491311061486 -0.043327077540724 -0.0300258581930895 0.00996656416128336 0.00124629147712823 -0.0857431009460524 -0.0595963841819187 -0.0384793787038982 -0.0607821239119822 -0.103505393194861 -0.110438378194782 -0.0167027690194026

TCGA-28-5218 0.0558233594557459 -0.00755559815091939 0.0337302516820536 0.0457958920327749 0.109277528504391 -0.0262192057294377 0.106926223545568 0.121047184763409 0.229858002570873 0.0229723153010978 0.0491470203451362 -0.131737803520532

TCGA-76-4928 0.0127444072709277 -0.0249111156885369 -0.0506465184102133 -0.0180317134705369 0.0534891571715959 -0.0128335266649968 0.0527488523422926 0.046753072504473 0.0359163557036016 0.0390009336725517 0.0783315684280545 -0.11452832740801

TCGA-12-5301 -0.121762905924997 -0.032122996342258 -0.0141344247912482 0.0833362969987153 -0.0136478600989426 -0.0370157750766774 0.0180937827009966 -0.0373144938748198 -0.0460071967061736 -0.00722530748351271 -0.00465691241916256 -0.350313482502712

TCGA-26-5134 0.0465076870657717 0.0304699562038171 -0.0243573091918987 -0.0168119592557812 -0.0381350404125473 -0.0203630980224116 -0.0286445263335577 -0.00497796246027352 -0.0233332196328234 -0.0738323929357878 -0.0353046572564841 0.0425960041923438

TCGA-32-4208 -0.0476131197705154 -0.0225456872404034 0.0289785710548257 0.10957827552314 -0.0529003286203942 0.0239601465900212 0.0220057090107677 -0.0319175307385139 -0.0171386202780127 -0.0711743001383671 0.00876195679970892 -0.0131108010539647

TCGA-32-1978 0.00604586268736338 -0.06554951304937 -0.0210707942808035 -0.00271798588475266 0.0226792310738025 0.0180084451954757 -0.0294996637239608 0.0316327496571019 -0.00241398168033006 0.0378037232824313 -0.0265810655057489 0.0288685189151405

TCGA-32-2494 -0.00320687283274521 0.0207258708184645 0.00863277549202105 -0.0186834382922852 0.0549381164856029 0.00823753063535804 0.0775686723915139 0.109979714421866 0.0582555163298776 0.0048661702970912 0.0450577657837958 0.0335627880754289

TCGA-41-2572 -0.0244669334821405 -0.0504313985770735 0.000546490569821735 0.0156833455079206 0.0307714625160058 0.0789010041016615 -0.00602465184971903 0.0234196379504313 0.00116867718192173 0.0647290954979434 0.0138702388989246 0.0366631459111982

TCGA-26-1439 0.0373269154733423 -0.0307717865727394 -0.007026668305414 -0.0412537598248873 0.0335373360930336 0.064141857222917 0.0213928190719667 0.0581493020603995 0.0237266525038238 0.0443691672821712 0.031630924310642 -0.0279515023815163

TCGA-32-1991 -0.00517251092225016 -0.00678704959338941 0.00566793463402324 -0.0150032310262004 -0.0374130136396481 0.139268483789186 0.0262914305469534 -0.0290907907219566 -0.00847718048139242 0.0346154791233629 0.0449465057968629 0.0352975485247335

TCGA-32-4213 -0.0584763337284756 -0.0231859229476556 0.0248315984302549 0.0713042318289019 0.0306503479553979 0.0658773906001612 0.0554330578669568 0.0509502432262666 0.0355867414654654 0.0193136751754163 0.0287463124483116 0.00836842841477967

TCGA-32-4210 -0.0130301857976769 -0.000922656350809626 -0.00917895486631071 0.0186174515727678 -0.0562635110110227 0.00625555145791076 -0.0242075032369574 -0.0868372484226128 -0.0725485022185235 -0.0450021813165943 -0.047828413701205 0.0726654819891208

TCGA-32-2495 -0.0327012271783554 0.00417599496704243 -0.0118661693787534 0.0232903577414246 -0.0499273899922959 0.0293880502968357 -0.0122368188497707 -0.0640511846441037 -0.0274943682702172 0.0384375682550286 0.00948460828970376 -0.0434084898796462

TCGA-19-1790 -0.0153931912263613 -0.0291279488398535 0.0184620568856904 -0.00169280763144784 -0.0553235028478989 0.0112676640643224 0.0624309719934596 0.0132446399489029 0.0780641965837012 0.0511322396589511 0.109097376733145 0.0262570337312349

TCGA-41-3393 0.0103694740138507 0.0107803239368962 -0.0175187999466922 -0.0348303950801075 0.0186804245489857 -0.0220949637958343 0.0382142499440044 0.0561391623385184 0.0285175863950408 0.0103266009360317 0.0386384124351416 0.02205723102092

TCGA-32-4719 -0.048133851394858 -0.0362315378391358 0.0905302179853861 -0.0612835721957692 -0.0983793092091176 -0.00448187130975701 -0.0574261710869097 -0.0608663791226898 -0.00558918727561307 0.00340702865198523 0.00798948340396017 -0.0016291684581607

TCGA-32-1973 0.0290824014653526 0.0362326828209588 0.0033227327936752 -0.0492402928550311 0.0188195306080668 -0.0269032393189285 0.0129781919074895 0.0266857684722921 0.00648697947122228 -0.0378441206748246 -0.0271709173392667 -0.00446803194501484

TCGA-19-4068 0.00644932988407587 -0.0283439859067834 -0.00985384663995538 -0.00356179674144544 0.0298384635909404 0.0744002890776136 -0.000326266405280825 -0.0116230729993379 -0.0395145707803772 0.0438843438855504 0.00875729544924287 0.00338267944573194

TCGA-32-2491 0.0252584050686343 0.0047627552358229 0.00631728305045299 -0.00760730266767658 -0.0076796596139486 0.0203930487970144 0.0386781311775228 0.0514627352883851 0.0381621276317784 0.0412986750704033 0.0828383453233875 0.000247669042925244

TCGA-19-2631 0.0392621904825296 -0.0505021252244339 -0.022867556947079 -0.0392577572104779 -0.0159865842271567 -0.0616979068199796 -0.0417103996372318 -0.0267040372251956 -0.0295265884795364 0.0382122920982622 0.0146579362952482 0.0157930032769539

TCGA-32-1987 -0.000268590321735427 -0.0176423724971397 0.0159206414321409 0.021283230491163 0.0256270949810184 0.0314385800601508 0.0858208587729246 0.0103922737048585 0.00883732107179775 0.0317119426269038 0.0547915511671214 0.0123815540294883

TCGA-32-4209 -0.0764537581506849 -0.0216524690622754 0.0150267472484202 0.057583314994948 0.00574411023178248 0.0061870248471884 0.0520721691789794 -0.00107206012864555 6.62608744223437e-05 0.046535837565111 0.0624503196733901 -0.0456238351554793

TCGA-32-4211 -0.0152078631831399 0.0313746238936207 0.00940602209949868 0.0190527480981085 -0.00134796146717399 -0.00480559260400839 0.0305340175174461 -0.00439381509392459 -0.0102974987975223 -0.0212640649402148 0.00120582789375659 0.0341740330633292

TCGA-06-0939 -0.103057827053032 0.0136616255186745 0.0329653340247045 0.0387134946275986 -0.0494619014670052 0.0335713064796357 0.0738773900216295 -0.0238980343106447 -0.0182466788757368 -0.00667523519045184 0.00407960442548638 -0.0305864012024736

TCGA-41-3915 -0.0211112266667787 -0.00950461615179012 0.00626446076254558 0.00196192087965742 0.0024942961793621 -0.0222695285639826 0.0435449976665535 0.0276569632956636 0.0535222240999626 -0.0151219863385025 0.0177859960725064 -0.0455529471838742

TCGA-14-4157 0.0536086539956539 0.0146131718108889 -0.0239690071753084 -0.0299074671948582 -0.0397094919119197 -0.112766150558954 -0.10437482453526 -0.0601662000608399 -0.0801731653780094 -0.136152185725622 -0.120806655869875 -0.0101891733544669

TCGA-06-5410 -0.121013616492533 0.0759338931303586 0.0462073502003311 0.0324623154245179 0.0454001034184851 0.0340325857353933 0.14717181199288 0.0312444450061019 0.0527641443312782 0.0322795255325657 0.108259502644114 0.0644741120871816

TCGA-81-5911 0.0166684501390232 -0.0325418497737308 -0.0670561214317801 -0.029589512399914 0.0757570127150538 0.035115651118387 0.0427217445620798 0.058543297443394 0.028373096260748 0.0592762867305529 0.0353431381114534 0.0322889790953933

TCGA-19-5953 0.0195770799030827 0.0592665087972474 -0.0577317017852456 0.0144385010059131 0.058215680998059 0.0106533953855054 0.0393311357724471 -0.0688967296548457 -0.0702865607634998 0.0183333482568234 -0.00942296003666205 0.00736233227297168

TCGA-76-6286 0.015519747731801 -0.0096024518294268 -0.0700938841681681 -0.00879360976843034 0.0825756189481243 -0.0113556906205864 -0.0240916401386437 0.023661522470324 -0.00961390043593947 -0.0074978495566536 -0.0422908854973383 0.0925622872334785

TCGA-06-6389 0.0256079273487484 0.0292685835345846 -0.0283336756258028 -0.0183389550750254 -0.0116566745980365 0.00234047824054537 0.0354527909535044 -0.0831161647632732 -0.0681559876686317 -0.119005451668849 -0.0376135750728162 0.0386969703334304

TCGA-41-6646 0.00915661489962339 0.0553460454858261 -0.0592635547625793 -0.0215838599031041 0.0672843976419693 -0.0203816420241217 0.0289028032301802 0.0537203407039745 0.0171770696214959 0.0403400457266637 0.0444311088186102 -0.0168025016752865

TCGA-87-5896 0.0545231458286678 0.00930974624487141 -0.0847901507644788 -0.0444269849767156 0.0957262414666064 -0.00684383124931559 -0.0150461893428302 0.047874886430716 -0.036005807103577 0.039398324760881 -0.00924337873587255 -0.123881176635135

TCGA-06-5408 -0.0242051775856887 -0.0386640811093801 -0.0426146547588763 -0.0102307461351603 0.00489268088438949 0.0626687498476604 -0.00326773605666266 -0.0153748103012345 -0.0107142705755151 0.0173145280964752 0.0167346796526822 -0.0269605738930655

TCGA-76-6664 0.00886125932166767 -0.0289052193635738 0.0377575912196629 -0.0758556777478588 -0.0158170464728043 -0.021284568880629 -0.0239842456142525 0.020364384503829 0.0507366867392463 0.00190231679972693 0.011433585067476 -0.0306252866578783

TCGA-15-1444 0.0662631944448643 0.0512573406893561 -0.0952997637164699 -0.00234586076828576 0.0799653734434939 0.0536321649045292 0.049291560180792 -0.0349440220419639 -0.0805437753689969 -0.0970582769074237 -0.0962958549836704 0.134890094138146

TCGA-76-6656 0.00372243684366218 -0.0146027015843623 -0.0881235783898892 -0.0252695081698234 0.0975854474391467 -0.00111536474795588 0.000171221766368912 0.0689534943757402 0.00110562452481831 0.0339244833938707 -0.00629906388161162 0.0284634532214982

TCGA-19-5955 0.0525403432369778 0.0412815675504098 -0.020180584802732 -0.0387944236736099 0.0283347941793349 0.00699637868349611 0.0827225724954405 0.0180429911834615 0.0397889603694882 0.0156623543496694 0.0408384784621355 0.019539364228074

TCGA-06-5859 -0.0105951179529847 -0.0203558116364292 0.0499263900412881 -0.0552393911642397 -0.0606578282974347 -0.00223175600680832 -0.0200068586193061 -0.0739352158046911 -0.0260744216074948 -0.0224618478023711 -0.0182390608860934 0.00721170980448712

TCGA-76-6282 0.020398935902899 -0.0158507904052223 0.0466815297944485 -0.0467869373400349 0.0141931096856043 0.00766684671281376 0.0493158925938606 0.0468840467549583 0.0794266080623039 0.0378496586456673 0.0714760711236712 -0.00849500588601063

TCGA-06-6695 0.0377421823972449 -0.0144555023001984 -0.00638335929916025 -0.0904212921668684 0.0145081069765847 -0.00808501473792729 -0.000222797349578142 0.0293404933243829 0.00377571178646161 -0.0140810977239387 -0.00420401945957107 0.0210225050960332

TCGA-06-1804 -0.00581803601957825 0.028535509535581 -0.041940533409315 -0.0835747474045358 0.0242991903174674 0.0423156386569973 -0.00377225831173552 -0.0363751906472237 -0.0524427378763592 -0.00834890876196065 -0.0371356730921358 0.0555992866638854

TCGA-28-6450 0.0820844049768539 -0.0255415947787721 -0.068607315692676 -0.0329608897468375 0.0523465275068722 -0.0349834090666989 -0.00404321526696448 0.00537232949224554 -0.0290575418904393 0.0358546815106868 0.00821092609620888 0.0533607340609974

TCGA-06-6694 0.0413467344281409 0.0384104084967174 -0.00456217216655134 -0.0364206695353309 0.0208328210610491 0.065378982623802 0.0371005731098112 0.0665887862768962 0.121242952576773 -0.0472129717222762 -0.00770759518467082 0.00657506124339225

TCGA-41-5651 0.0153486143249233 0.0981637787103311 -0.0669893009364254 -0.0118669957365911 0.0464852333246557 -0.0784934490999001 -0.0477603226095812 0.0278530365129128 -0.0457374775288728 -0.0958532412188673 -0.101263642750276 -0.137729881427478

TCGA-06-6698 -0.00771468190307643 -0.0184826230281342 -0.0463531494937633 -0.0450354863283481 0.0134189411955982 0.0157651870316075 0.0632157330663279 -0.0253588961388496 0.00784271691133625 0.00917406429216759 0.013174987237792 0.0480599509317581

TCGA-74-6584 0.0187681454658609 0.198263674987634 -0.0726737812585469 -0.0965046514438348 0.00430325056913788 -0.0348969333360335 -0.0163208050348037 -0.0613756751352925 -0.0752746518098922 -0.0918306309434732 -0.109326352006816 -0.0121005706196475

TCGA-74-6573 0.0252193160872075 -0.0739240350368319 -0.0457958564378523 -0.0450073490791696 0.0228337893193569 0.0430313296800404 -0.0210074597684528 0.0211650976438095 -6.0559345152454e-05 0.0469102419921982 0.0122936020817345 0.0279438825577815

TCGA-06-6700 -0.0616406320107466 0.202132105758883 0.0166186725827945 0.0182176249007127 0.0134432476428703 0.0733660650647854 0.029743145032469 -0.0541708494988469 -0.0413776051760487 -0.0337644084366948 -0.0404329194869164 -0.0477320134154963

TCGA-32-1980 -0.0373365490175133 0.211523562305516 -0.0239673879383153 0.0406641997904569 0.0724196994742992 -0.00784132737902773 0.0809875261328181 0.0342845887485147 -0.0130193454067007 -0.0267667340973294 0.0126218498202152 0.0999809619917557

TCGA-76-6660 0.0754496052278468 -0.00738533885484809 0.00219080745433178 -0.0977546646567414 -0.0638186987281024 -0.0533153302732197 -0.0343098196484326 -0.032342641423011 -0.00175492394812083 -0.0390433512020176 -0.0062641645531058 -0.0140306029461102

TCGA-76-6191 0.00597883344192459 0.0721506923782324 -0.0504296869438889 -0.0704650982775681 0.069305643197334 -0.0296548262419746 0.00203556143518413 0.0852006579686188 0.0387066005382521 -0.0641403944779284 -0.0186697678510282 -0.0107495358728037

TCGA-06-5856 -0.0186337217855088 -0.0177230261693657 -0.0402832944913372 -0.00677184733198479 0.055727541995502 -0.022327739523182 0.0198477323347093 0.031593724491185 -0.0246614187739444 -0.0138191685843711 -0.038488560522407 -0.0681694610609457

TCGA-26-1442 0.0570966344291182 0.0614569196887862 -0.00176547139415414 0.0296366326244962 -0.0142095750951947 -0.0472212414995285 -0.0474199730940329 -0.0702755087905698 -0.0571444293142412 -0.0983627029964952 -0.0592793979339489 0.0169737649778011

TCGA-26-6173 0.0500576916513313 -0.0312539271585575 0.010255373110372 -0.0749747881458978 0.0357667149528613 -0.0132432974075916 0.00898140946262272 0.0766883800025833 0.101842250290596 0.0423222918683418 0.02745896764222 -0.0373622768822203

TCGA-19-5958 0.0141952636215113 0.0709300229578141 -0.0844438800680331 -0.0280198211842499 0.0917060272091741 -0.0351139742324983 0.00967809428561646 0.0179425477982626 0.000965389420557036 0.0236820747369689 -0.0206983622919697 0.0968209213083319

TCGA-19-5951 0.0520499481367663 -0.0630080487357622 0.0347268610418461 -0.0169192389852291 -0.0133658601965249 0.0223120117413913 -0.0650343051997017 0.0133435629600349 0.014260909950276 0.018461202094584 0.00913821454561857 0.089690620573542

TCGA-76-6285 0.0223650326172356 -0.0188943732238377 -0.0162536506452658 -0.0455308166527418 -0.0119264841119393 -0.000360612330584396 -0.0194511419574079 -0.0183181119025845 -0.0138396815572732 -0.0404225485425048 -0.0105824241906396 0.0021514970684812

TCGA-32-1979 0.044589847721857 -0.0286100007343053 -0.0617582079808464 -0.00797321216026907 0.0679146957595387 -0.0164336779113255 0.0704206380744817 0.0337469632969799 -0.0341065343927815 -4.78989319694625e-07 -0.0200140343522562 -0.0510313236594317

TCGA-14-0740 0.0361185065954395 -0.00523883051329698 -0.0828071034075479 0.00822472959024667 -0.00244445047695132 -0.0979287738173129 -0.0624917349517112 -0.0145930675796097 -0.0548010509826324 -0.0885559142094184 -0.0922579882338355 0.227279408053586

TCGA-19-5947 0.0637172711452072 -0.014540761256479 -0.0099388039117178 0.0106685672656155 0.00294412562780383 0.011257136562158 0.103500715430455 0.0607100590173727 0.193381847002201 0.0404123000814618 0.102454340660556 -0.0659280383174177

TCGA-14-1395 0.0102148894653041 -0.0693826233931318 -0.116110212906247 -0.0448803180434567 0.0669770127877995 -0.0514911207368968 -0.100831222609466 -0.00277534593025167 -0.0290905021884536 0.048181180546986 -0.102071843040199 -0.0324651162725657

TCGA-19-5954 0.022854691694175 -0.0498758458940036 -0.0287027843383334 -0.0564775031117763 0.0236366090831994 0.0936793183555468 0.00752861125272667 0.0290492100707832 0.0245811558765144 0.0207710359472872 0.0323057552719666 0.0144067572262064

TCGA-28-5211 0.0716975741227117 -0.0199519145175428 -0.0217970662774538 -0.058060363734809 0.054109861850343 0.0401523046246268 0.0607545299462956 0.0898070169678782 0.0459666419425303 0.0127870166771824 0.0599105475851435 -0.116127481729107

TCGA-76-6662 0.0260664564480707 -0.0413505181312946 -0.0310830747237228 -0.0267967418638732 -0.0111763512335075 0.0481235747293827 0.0189080351707577 0.0260747101119008 0.0451032207188566 0.0149091093703153 0.049212435859875 -0.0719756723177781

TCGA-19-5952 -0.0357368761250945 -0.0224555383264388 -0.00734813441306215 -0.0430022210103354 0.0329520221128027 0.00999703538418935 0.0239871150141246 0.0366477789984291 0.0617708898780277 0.042118961607785 0.0303641385576128 -0.0730842189644444

TCGA-76-6280 0.0154080076893997 -0.0705378481229709 -0.0785390144342983 -0.00923691155384479 0.0875650677472068 -0.0303641512406438 0.0123210383966226 0.0844554834625814 0.0356818062757784 0.0578632981774458 0.0535462189055052 -0.0299838527345899

TCGA-06-5411 -0.0238203111933639 0.172983422320231 -0.0384361620187416 -0.0104376677330039 0.0297368369499711 -0.044528055103996 0.0219867300085013 -0.0142425165839342 -0.0453001754906457 -0.0790945927875005 -0.0561123919898463 0.0487127942053097

TCGA-76-6661 0.074738728335638 -0.0123148388792557 -0.0455539368299629 -0.00279050614864825 0.114422237010155 0.0177067441183226 -0.0178656879295087 0.0906280697381365 0.0478563696897856 0.0732947666596362 0.0232631463805002 0.0846400981121166

TCGA-06-0650 -0.0478601737843126 0.0544579080094452 0.0370942602372201 0.0215152579831757 0.0861562852195963 0.0223667903877038 0.0549470649323451 0.119774090024192 0.123657947222126 0.0237563586301766 0.0918794410246213 -0.0390097171158046

TCGA-81-5910 0.0602482641553231 -0.0765184394620134 -0.0577567626108734 -0.0220292498628157 0.0201162604072467 0.147392257716978 -0.0192731261681997 -0.02831054178803 -0.026663163962974 0.0542081121930614 0.0250659090113835 0.0666750662112663

TCGA-74-6575 0.0146279907672254 0.0932096101622318 -0.0683267167970123 -0.0519312535717501 0.0524842620194957 0.0105578746281915 0.0802512370045036 0.0525721505815795 0.0196008997839957 0.0172672165182452 0.041950670122668 -0.114106477561275

TCGA-26-6174 0.0355259114072695 0.171964186922032 -0.056242423237955 -0.0459583165296809 0.0114094523275971 -0.0450894666238648 0.0030994528805939 -0.0605268638549179 -0.0620141777108657 -0.079182381639799 -0.0629277703439177 -6.29850951011095e-05

TCGA-06-6391 0.0812798201392424 0.0793593594890877 -0.0827018198332552 -0.0319666016392959 0.0781132865595908 -0.0536411604166093 -0.0181693703735252 0.0436143127139746 -0.0423337317520521 -0.0688479390892254 -0.0512368999144936 0.0775800028818496

TCGA-06-6697 0.00525131641399228 -0.0297640832218309 -0.0645595796210059 0.00892530100334566 0.0426525891301903 -0.0148779052878583 0.0962618137333411 0.00939526079602465 0.0735877983878567 0.0389106509691466 0.0349924812145367 -0.101107932573959

TCGA-06-6701 0.122489897579992 0.00573873824343638 -0.0523303463927714 -0.0469386957367952 0.0291361937673845 -0.0410064975192646 0.00956046378387877 -0.0912047625546353 -0.107835524781668 -0.116234270580162 -0.102367798148626 0.00165433770717905

TCGA-06-6693 0.0278570309372586 0.0145500748026706 -0.0826983956191174 -0.042923602712514 0.0120798451361342 0.0860861761107928 0.0136749576843808 -0.0171636985057594 -0.043993512299673 0.0215346880704617 -0.00535416419178008 0.0286148298404455

TCGA-06-5412 -0.0573102554515353 0.00799600698573842 -0.0100075858667781 0.0716716917682491 0.127547965252776 0.0396147182310407 0.121948835418896 0.0593572330396557 0.0315708647280615 0.0529214701385793 0.0702917647314276 0.0940940433238006

TCGA-19-5959 0.0436593898401745 -0.0252587066928578 -0.0509401584198937 -0.0142789535561684 0.0161784720565192 -0.0541550634209072 -0.0287290312679615 -0.00593626448493137 -0.0223304676837818 -0.00155442351956882 -0.0335790173551443 -0.00489902372476228

TCGA-74-6581 -0.0750964926483032 -0.0231791845195534 0.0102476224448781 0.0643295061623342 -0.000275388184223801 -0.0610846250058903 -0.0124571356987828 0.0270140066106489 -0.0102558574657631 0.0075604898694877 0.0168250611984848 0.127122263201449

TCGA-74-6577 0.0155126996013054 0.0242630018662544 -0.0510636951323468 -0.0531416012129669 0.048160776102928 0.00405816963582346 0.04736529616423 0.0525130489645631 0.106872012573392 -0.0212500316969207 0.00773836203191929 0.0235029848144548

TCGA-14-0862 -0.120265284400329 -0.0537311498937502 0.0183568080160103 0.108006496752943 -0.0174773071132015 -0.0125074950661638 0.0737518222975724 -0.0530533947951099 -0.0420309949864923 0.0215201155115832 0.00724506020441898 -0.184515011933295

TCGA-76-6193 -0.00822853604504335 0.0553470637995416 0.0104049369434039 -0.00335413641513175 0.0529884689157562 0.0510512329199051 0.094929443703723 0.0619098133644836 0.0520625805057362 -0.0172822880700175 0.0727602613787573 -0.00688645656840277

TCGA-06-6388 0.0460901924830906 0.0784800364481464 -0.0494903920641681 -0.0907730768159107 0.0330284896465184 -0.0523264819427375 -0.0489448575801645 -0.00571806903854639 -0.0220931513359063 -0.00276770681705824 -0.0386207404672547 0.0437788900361534

TCGA-06-6390 0.00465458602266362 0.0185592019215186 -0.0379413625751362 -0.0222813114487701 0.0724839931397504 -0.0195052044129339 0.058068765742507 0.0164054844903655 -0.0094797150982841 0.0195957303204517 -0.00223287318116321 0.0589078934265552

TCGA-76-6283 0.00687436424450407 0.0600862629440272 -0.0372372019984216 -0.0583579672319946 -0.0129070167842889 0.090919703379795 0.0105790672802254 -0.00140612100410015 -0.0245992017819763 -0.0488403636935665 -0.0165875231881525 0.0666561276072627

TCGA-76-6657 0.00171764932550059 -0.0134308996797946 -0.0741854672705539 -0.0305413715119463 0.0842648773534399 -0.0282229360755942 -0.021159117190275 0.0724382703644103 0.032286101589921 0.0455020838806709 0.0397267524251831 0.0032982582663523

TCGA-06-5413 -0.0545367232104867 0.00835706116834468 -0.0538015909412821 0.024880759721169 0.111339981878948 0.0186559240023746 0.0936754632785329 0.0797696290627603 0.0507395286041276 -0.0165947900535976 -0.0275259001663604 0.000301955496393736

TCGA-14-1450 -0.00568381499215979 -0.012658567199245 -0.0387971423072505 0.00198569990382619 0.116807639586691 0.0275311008244357 0.0422163155817072 0.0812907004090465 0.0255208951900128 0.0487546744041988 0.0470586302643165 0.0246785296331065

TCGA-74-6578 -0.0525130759037252 -0.0195224407060168 -0.0269063357985858 -0.00930076847053836 0.119489323970438 -0.0327570009992972 0.0248520243649331 0.114742218610789 0.0493534247713666 0.05120624570606 0.0256854557926344 0.0389179874532644

TCGA-19-5956 0.0627125216598367 0.0193649997880204 -0.0571897820579872 -0.0780471285256997 -0.012885483126112 -0.0769028367602298 -0.0952156978401445 -0.0361511038519215 -0.0479704691587516 -0.0937253000981149 -0.0646647542770875 0.0228811375869208

TCGA-06-6699 0.000619965853092659 -0.0692337501745764 -0.0995067164146438 -0.0386057348344484 0.0666436579064435 -0.0360956594238839 -0.022094599354195 0.0442196496072541 -0.00472120048637273 0.0495426194253842 -0.0487497978486872 0.0154805204327446
