# Supplementary material for: Identification of glioblastoma immune subtypes and immune landscape based on a large cohort
Source: Hereditas. 2021 Aug 19;158:30. doi: 10.1186/s41065-021-00193-x (PMC8377979; doi:10.1186/s41065-021-00193-x)
Supplement: Supplementary file 7 — Additional file 7. [file 41065_2021_193_MOESM7_ESM.docx]

p.value HR Low 95%CI High 95%CI pearson_cor

ABCD2 0.160345837125232 1.0690996352167 0.97388258308743 1.17362611250013 -0.516745024166993

ACHE 0.0786278041606919 1.25949689333008 0.973943557118079 1.62877244036821 -0.235431528324321

ACVR2A 0.463160145632524 1.07112957101789 0.891494552495357 1.28696081731239 -0.282260168813786

ADAMTS12 0.686709258330553 0.97290985853424 0.851356040043141 1.11181873189641 0.24153486980272

ADAMTS14 0.698642057731092 1.05445748623943 0.806203409652387 1.37915639772075 0.581836183338142

ADAMTS16 0.29305922928133 0.844655008424122 0.6165997825088 1.15705860348041 0.436698121681781

ADAT1 0.934820627907786 0.991452751597804 0.807100494024426 1.21791346421987 -0.317757652151385

ADCYAP1 0.348841186611785 0.953776530675653 0.863866989379729 1.05304367645864 -0.284214206220585

ADORA2A 0.00182914547199298 0.676857513024861 0.5295445302359 0.865151213504387 0.631096337613326

AIM2 0.277025313330426 0.954845839422978 0.878520485082917 1.0378022966389 0.0803390114611092

ALDH3B1 0.63411095743883 1.05436351924808 0.847843440900588 1.31118833630457 0.680038804748706

ALOXE3 0.343392159709717 0.814994994954881 0.533789718409674 1.24434176772909 0.662274416231482

ALPK2 0.624057074060589 1.01607708159538 0.953293605249611 1.08299544868242 0.317419638891959

AMHR2 0.283053308722293 0.934565105389874 0.825940309495563 1.05747585651294 0.891606441981877

AMIGO3 3.7518028666554e-06 2.70711361849627 1.77503886576595 4.12862179233785 -0.322327963093502

AMPD1 0.361019637138442 0.949773393437822 0.850351513635664 1.06081953688259 0.777836844095728

ANK2 0.0506829115061116 1.11636729634728 0.999672815754591 1.24668383566378 -0.692168162963166

ANKRD44 0.155129405329767 0.863162300442365 0.704669922275664 1.05730233880125 -0.295083911030594

APBB2 0.96811500509096 0.997304260757532 0.873665813094534 1.13843963403145 -0.433880567337534

APOBEC3D 0.926087902307158 1.00733824057811 0.863154274146168 1.17560714385013 0.396607025313873

AQP10 0.136613793415815 0.861618697680453 0.708179319056689 1.04830338900808 0.865084835677006

ARHGEF6 0.105944767714507 1.12221556924893 0.975814598237217 1.29058100395272 -0.688310830593628

ARSB 0.685287037178414 1.06529481692982 0.784543319531835 1.44651419332046 0.482045031755714

ART4 0.0421036694403765 0.886927561927312 0.790016070578569 0.995727212903693 0.857538871854639

ASGR2 0.194569513703116 0.912940982391235 0.795561725158134 1.04763868216989 0.899451873194362

ATE1 0.00519556659965633 0.745424345839879 0.606620083081229 0.915989217746368 -0.394284039074714

ATP2A3 0.0547897014295478 0.792425764929666 0.624946806256667 1.00478726611249 0.805555198720495

ATP8A1 0.669244720862344 0.968678097982177 0.837085155401262 1.12095794729578 -0.503368515504164

AZGP1 0.3732332686971 1.05596000453774 0.936690670931101 1.19041596739183 0.241406621224411

B3GAT1 0.490616442483275 1.04053915326685 0.92936786195156 1.16500879125271 -0.278002641391714

BCL2L14 0.0510962394098401 0.799259189025334 0.638133672874082 1.0010680808682 0.386894894571486

BFSP2 0.0291039156581469 0.857121802679952 0.746278004505361 0.984429100407787 0.579328320199005

BLK 0.0148459844509947 0.877921139846983 0.790611107683368 0.97487313332676 0.758055037365562

BMPR1A 0.743646320728061 0.972603415748884 0.823439988011568 1.14878730459846 -0.492878922537973

BMPR1B 0.240481189117584 1.08733003834913 0.945465807000423 1.25048056052628 -0.601832539635252

BMPR2 0.860076577339631 0.977746382111085 0.761297955212777 1.255734343151 -0.596037735193995

C16orf54 0.536540498823811 0.91771785171324 0.698960842364146 1.20494025459926 0.268717542623407

C1orf116 0.00848803971137905 0.744838276843802 0.598124030093026 0.927540160132606 0.708952939828504

C1orf127 0.465238181761453 0.856441354173903 0.565015334010526 1.29818033066969 0.403074019313091

CARD11 0.547488826591072 0.952600644872651 0.813197244159716 1.11590145580174 0.768290626204707

CCDC69 0.202985873053921 0.841160941432802 0.644507619702408 1.09781747765654 0.720652431111185

CCL1 0.0933333294495879 0.835158207675019 0.676699261043322 1.03072261490514 0.570852765909636

CCL16 0.0334482707674557 0.854924108229629 0.739930518777291 0.987789004891979 0.847558433884982

CCL25 0.590321108499986 1.08056136320744 0.814980261760883 1.43268851338059 -0.269788330920681

CCL28 0.14947567700416 0.914644413554826 0.810147361774787 1.03262004262332 0.589391226714855

CCR10 0.718094958756104 0.959311944810187 0.765626173013445 1.20199575183455 0.447430388772806

CCR3 0.599947025768258 0.910555609900922 0.641556914096479 1.2923428935213 0.178517204366257

CCR8 0.0873855733226775 0.736432434901604 0.518544416693545 1.04587517233961 0.210105069504196

CD19 0.00152243924390321 0.73502019845771 0.607636419028861 0.889108478725253 0.649056108074307

CD1A 0.00124971202119798 0.820155547406332 0.727117325215985 0.925098465700254 0.824555743242549

CD1B 0.0766446335060031 0.894583449935542 0.790795983286004 1.01199243017544 0.806733173441087

CD1C 0.00360737744286818 0.805821332511863 0.696788535596761 0.931915476156713 0.792520404978142

CD1E 0.0405510003751609 0.898948836362391 0.811821780158379 0.995426619669705 0.864407233598139

CD3D 0.159269122699055 0.910624020809564 0.799317980165882 1.03742956852201 0.720048331667781

CD3E 0.0541980187275093 0.774747257653993 0.597477540758901 1.00461234489247 0.843828778473347

CD3G 0.0497884209337647 0.906408864898197 0.821651655101277 0.999909177161909 0.656608636108428

CD5 0.149644506205092 0.870176783915669 0.720199908646643 1.05138535311497 0.775959099601395

CD6 0.281210863744103 0.874651030654939 0.685555990259918 1.11590364068689 0.591628872210367

CD70 0.809124347494233 0.986518785050166 0.883642783477654 1.10137187951297 0.585984998851182

CD7 0.216640880160517 0.854691511974616 0.666245806675138 1.0964385416352 0.715176422163307

CD79A 0.84304523640967 0.958821811055423 0.632360491204789 1.45382148021938 0.410797577624738

CD8B 0.178515697758858 0.903810555838196 0.779989168297319 1.04728828815373 0.837541451031094

CD96 0.0497758843510067 0.835443438145846 0.698088618724325 0.999823975953659 0.735356395136412

CDH20 0.665284644405853 1.01821601663282 0.938257897508343 1.10498814801437 -0.577462428253221

CDSN 0.184949405910086 0.880002502983343 0.728458695682772 1.06307249792812 0.783200092390906

CHRDL1 0.169070703149322 1.08525419751143 0.965808715573865 1.21947198676538 -0.268289939936761

CHST13 0.294955020147575 0.897240471641234 0.732455961187399 1.09909742921051 0.739268762226683

CISH 0.0982231612385942 0.860725356156296 0.720540179872017 1.02818435310849 0.507979121585616

CLEC4M 0.664224696008789 1.10009352963143 0.715104347863163 1.69234850487654 0.359849527251959

CLIC3 0.877728954540115 0.988206107359504 0.849586195687954 1.14944347681151 0.380778395132843

CLIP3 0.618422045765635 1.04490357968735 0.879046336028906 1.2420545380756 -0.626596124755179

CLOCK 0.00982385795018563 1.30432962582713 1.06609813209527 1.59579659844888 -0.292907872175871

CMA1 0.106481806137843 1.18325796907183 0.964599089340194 1.45148325023787 0.304627065908177

CNTFR 0.192595405591951 1.12967315034415 0.94036801421702 1.35708723320523 -0.507482804740027

COL14A1 0.809970898962582 0.988728129994953 0.901466809045246 1.08443628232823 0.37743428864429

COL6A6 0.298191676704748 0.903765596022507 0.746904063764843 1.09357050279898 0.36743530131812

CPNE5 0.511180081075848 1.04468188386042 0.916961014048872 1.19019262732581 -0.509077849939811

CPZ 0.973898414862447 1.00165941416335 0.9069550690371 1.10625279711737 0.407080276177755

CR1L 0.0518860227025905 0.910237326667298 0.827895494403648 1.00076881256038 0.795926861680521

CR2 0.0152758268592263 0.801648014690531 0.670509542483284 0.958434591515588 0.751355441358619

CRCT1 0.03278240487414 0.770136233083022 0.605929357202882 0.978843177767861 0.525623394481318

CREB1 0.84594403404746 1.02086443657538 0.828900741248865 1.2572846735475 -0.642649463922262

CSF2 0.0346025447513394 0.860910434752734 0.749248842946231 0.98921310809329 0.482694371918091

CTLA4 0.086267962508766 0.795439544296797 0.612414384915924 1.03316330284757 0.526204434081228

CTSE 0.11344012812575 0.725803364351963 0.488073798991851 1.07932555444023 0.686563048692807

CTSG 0.0706223699527511 0.881045180050715 0.768015607155084 1.0107094205624 0.818750297998665

CTSO 0.0129249625758145 1.13650648456304 1.0274388640077 1.25715216223723 -0.693374116217634

CXCR3 0.182942424362166 0.908294163622498 0.788401833148027 1.04641853048025 0.533644763359294

DACT1 0.191911005590425 1.09717455722733 0.954516893609916 1.26115317296726 -0.353488603747114

DACT3 0.801302151723942 0.976507746513727 0.811463946829088 1.17511983462422 -0.314437893877825

DDI2 0.19100540837366 0.771473112278897 0.522910189517844 1.13818926251575 0.36280397463063

DERL3 0.461050947310796 0.940547084172247 0.799098892592849 1.10703296643867 0.896148634875597

DMKN 0.351856462878889 0.949955438026164 0.85263518736517 1.05838387578648 0.394173808338675

DMXL2 0.254532156080523 0.89714952857076 0.744363305458057 1.08129628464079 -0.383801464648816

DNAH8 0.00857826511510133 0.811371435960297 0.694267009804253 0.948228272113762 0.821397971286729

DSP 0.0198179721825708 0.834802308391876 0.717157504037911 0.971745941683075 0.535351398112755

E2F5 0.222195114374232 0.910746199312123 0.783801605990082 1.05825075277018 -0.370784367985246

EDA 0.888109135855842 0.987054178787883 0.823206757577816 1.1835130638739 0.352430474006415

EDNRB 0.270618667503768 0.955865961317142 0.882105671906404 1.03579397016018 -0.410855765256109

ENG 0.773956952475386 0.96734699705135 0.771243004410728 1.21331436052277 0.594236425744057

ENO3 0.689657710741097 0.962589337232656 0.79829869734632 1.16069114885707 0.585987462263031

ENOX1 0.9989786242466 0.99992189678865 0.887216442864312 1.12694462294848 -0.293760155185348

ENPP3 0.0039734319304941 0.800474661826312 0.687982633726142 0.931360259423364 0.643931892198009

ENTPD1 0.42361563971975 1.10228453703441 0.868354954901114 1.39923333623808 -0.633181063388925

EPOR 0.124926479569406 0.835167528207269 0.663517641417741 1.05122269044946 0.571721178965508

EPS8L1 0.181654777173052 0.895018773753441 0.76059060117602 1.05320602717483 0.770350085027911

ERN1 0.0691600774267368 0.76342322834955 0.570600950446454 1.02140563405588 0.750911048722194

ETV3 0.27292136039772 0.827880494653072 0.590633802369878 1.16042480243588 0.51896938776353

EVPL 0.526486420849066 0.927517075758589 0.734853769183052 1.17069267642208 0.766017405486369

F2R 0.847495952014972 0.987724522348 0.87089778378902 1.12022300459078 -0.422733949322425

F5 0.174930686814091 0.948976848746135 0.879819732103744 1.02356997302483 0.364394407216626

FAM129C 0.58180996569191 1.11409882006119 0.758461037821142 1.6364930022344 0.212821051552307

FAM46C 0.000175225330055071 0.669392577097136 0.542784486731 0.825532846326196 0.665339091258537

FAM83A 0.0982342648162338 0.924994563478894 0.843338441846479 1.01455702717897 0.899415519459399

FCER2 0.0104140775086062 0.787659919777383 0.656189595594391 0.945470872121547 0.540705291428264

FCRL1 0.548594771169257 0.900422187403591 0.639155969761166 1.26848555583643 0.289540254560751

FCRL2 0.0673538962321898 0.832594578483235 0.68420161985383 1.01317172015432 0.661209659542984

FCRL4 0.0881536970736158 0.65964488110891 0.408909147894866 1.06412725519426 0.186558362543445

FCRL5 0.882236258383602 1.03123689785778 0.686457648748242 1.5491844856599 0.258318463093644

FGF14 0.0071451375298033 1.09485619690012 1.02489908424641 1.16958841149904 -0.669766453420672

FGF7 0.944879306535076 0.991986647245449 0.789681224362953 1.2461199252991 -0.390517007838865

FIBIN 0.237773968558739 1.06838526234832 0.957269573480078 1.19239877713177 -0.640076129220134

FMNL3 0.275414839285029 1.18294011807527 0.874676720715583 1.59984516543107 -0.50305790379449

FOXP3 0.00083559684036253 0.469076572596737 0.300861330553413 0.731343009599694 0.320014279579412

FUT7 0.739798981137733 0.913375245916216 0.535088235554043 1.55909677025267 0.131910791239983

FYN 0.758732373549075 1.02648899736338 0.868760493271433 1.21285402578597 -0.503447039431852

GATA1 0.122135113566415 0.887963165128009 0.763776947771742 1.03234142497292 0.82027150806075

GATA2 0.153518873749682 0.90160574020766 0.782063242453563 1.03942094021082 0.822917395364371

GATA3 0.233203934573623 0.930038028607457 0.825493902289201 1.04782207628351 0.751508283562454

GBGT1 0.741105867334596 1.0455091119955 0.80291509748567 1.3614008588064 0.583181941505765

GFI1 0.0104941368021755 0.839628214394023 0.734424140757398 0.959902458652119 0.819853019488204

GFRA3 0.652360749297956 0.900377571765765 0.570343043353587 1.42138977793445 0.42196984711937

GGT1 0.642381373714578 0.953543308795293 0.780086924801711 1.16556862170122 0.781535756062638

GJB3 0.320647401749892 0.869461937345394 0.65972059920642 1.14588518442771 0.715298782769314

GNA15 0.453119104944478 0.945025438551865 0.815247764323929 1.09546216327346 0.66570109514826

GNG7 0.0998894100812215 1.12343272493535 0.977993921202286 1.29049993061727 -0.361723968652434

GPR157 0.828989214886502 1.0170772467538 0.872216701847449 1.18599669517131 0.769742791556459

GPR174 0.0356938869933027 0.863486123584852 0.752961413886956 0.990234389003544 0.696566101222599

GPR35 0.412575715267189 0.912178242760953 0.732130990627472 1.1365031083486 0.402861015018495

GPR55 0.190425250156959 0.751688373354405 0.490314921459987 1.15239285183023 0.383763687602992

GRAP2 0.0112671800813757 0.800019501049937 0.673226230635684 0.950692609608884 0.791035050136767

GYPC 0.242446365153995 1.09167790470341 0.942364346610247 1.26464955078625 0.511615381767795

GZMM 0.112528942753561 0.845399572236332 0.687034689783309 1.04026834069003 0.815776869058858

HAVCR1 0.00365177771472348 0.716911446444327 0.572808629987478 0.897266547911704 0.311895985107465

HSH2D 0.139410666047269 0.919983516098607 0.823661380997468 1.02756993276556 0.871598113702076

HSPA1L 0.441766045168185 0.930114707708881 0.773334367222589 1.11867958565376 -0.376561401103004

ICOS 0.00438218078734874 0.730358176916456 0.588388354254065 0.906583318198031 0.439635965288147

IFNB1 0.50491171953202 0.913627680618669 0.700572074606065 1.19147703576682 0.12874404912637

IFNK 0.00739306140363935 1.29954920634018 1.07282582564108 1.57418669399592 0.181678074084134

IFNW1 0.0441143657664097 1.24518263431788 1.00579436299557 1.54154750697647 0.243114506464517

IGLL1 0.994159926114831 0.999136707221281 0.792843404397626 1.25910634329542 0.737141505841401

IGSF10 0.0552856955013416 0.876951930715492 0.766764703210271 1.00297351399434 0.554460747201939

IL11 0.835597789422272 0.988378828805207 0.88507193105658 1.10374385962524 0.685024394512064

IL11RA 0.328847086672852 1.05588517868873 0.946698978542538 1.1776642162337 -0.18244157300861

IL12A 0.836551896531192 0.983590555161298 0.840521228015983 1.15101242890217 -0.416010139681745

IL12B 0.741572857880715 0.951724304098205 0.709241704453032 1.27710926377313 0.312252243562712

IL17A 0.34457550135637 0.797315526188523 0.498474581127883 1.27531487536009 0.391119290879962

IL17B 0.641535687335021 0.971916539135772 0.862079449780588 1.09574791428572 0.354512631196614

IL19 0.280572503276565 0.897789932480784 0.738109911170603 1.09201455049633 0.652648321008526

IL20 0.000824727703419622 0.688813822428166 0.553632204859903 0.857003038846276 0.507365189713865

IL21 0.828095843945202 1.03364304008232 0.76676173354419 1.39341582602471 0.345776747353507

IL22 0.515251177273863 1.07268223704464 0.868334706558644 1.32511941879105 0.210885999293675

IL22RA1 0.253161089556379 0.915336517783855 0.78647383929018 1.06531317245702 0.742095422549175

IL2 0.110711905300976 0.802401326310092 0.612218591517358 1.05166340484441 0.357368803871573

IL23R 0.00323101819492303 0.840863836190771 0.749249590342557 0.943680183649078 0.82635067924527

IL24 0.0915717532505155 0.754663469611954 0.54417732919758 1.04656501072277 0.578989405692591

IL27 0.766645129762772 0.979983547912156 0.857480116837082 1.1199883651191 0.191849139094367

IL2RG 0.0212949027549436 0.826761390708063 0.703166273184621 0.972080748511758 0.906416725858327

IL32 0.114689680647281 0.86897033167242 0.729826735667752 1.03464206012677 0.568672926098313

IL5RA 0.219224553389015 0.84363657690681 0.643208403726771 1.10651955069507 0.463647048696684

INHBC 0.088529101533812 0.653574821119989 0.400632992038541 1.06621285638136 0.590529834094956

INHBE 0.0278226355853607 0.804791871009052 0.663207299701426 0.976602573484699 0.817819434589795

IRF4 0.0136151014774519 0.803791380135014 0.675756613581285 0.956084734939311 0.877221902294439

ITGA2B 0.0869556342144636 0.723706611088155 0.499751658074439 1.04802305399192 0.606411452296197

ITGA9 0.336071941290809 0.869861222170322 0.65475763444158 1.15563149787627 0.390487313735113

ITGB7 0.805979132431008 0.982706793948175 0.855005404725715 1.12948133138609 0.642466487113728

JUP 0.884694661108527 0.982454983236922 0.77342400630632 1.24798013278215 0.297939995102987

KCND2 0.778584005126621 0.993041300207219 0.945859918395835 1.04257618357453 -0.564071956499446

KCNJ10 0.775921475179941 0.971720896788669 0.797544533481194 1.1839357698739 -0.518376365870002

KCNK6 0.111307979529552 0.837333781426875 0.672971851777679 1.04183831711026 0.767425417466988

KCNT2 0.986021066815298 0.998870696450451 0.880266837484269 1.13345479545597 -0.477804797513086

FAM30A 0.98141465075233 1.0020079140983 0.846401761400673 1.1862213734694 0.35112473430622

KLRK1 0.112795466853426 0.901332788788365 0.792727606918034 1.02481708603976 0.535089255501176

KRT14 0.117994053473348 0.844522595734487 0.68327927989612 1.04381683403973 0.799017625956531

KRT16 0.111059573343272 0.919673315089998 0.829664276289725 1.01944730014297 0.850200674914891

KRT6A 0.651171163321796 0.977641605391337 0.886358755012954 1.07832534308095 0.845931638221211

KRT6B 0.291849407817585 0.918823761282995 0.78499247898504 1.07547158335809 0.708543275606168

KRT6C 0.127812254725952 0.907742371308533 0.801413516985213 1.028178580976 0.889854078903207

LAD1 0.166494066647543 0.887687693025273 0.749833723405934 1.05088557069597 0.858349970797975

LAG3 0.423792848475904 0.946827605602216 0.82818130188545 1.08247132927232 0.334146592582669

LAIR2 0.0361322220526546 0.88128143606167 0.78302863857342 0.991862789286854 0.594855424956108

LAMC2 0.00162109648629238 0.70463133030146 0.566793391255376 0.875989945018076 0.493571132648891

LAT 0.457197249205415 0.934943500121651 0.783012370339415 1.11635445560179 0.587893645824427

LATS1 0.00101689269632874 0.632340219837834 0.481082219293894 0.831155543872406 -0.225321446346276

LAX1 0.0718538293022728 0.815358852590294 0.652862254619143 1.01830065039552 0.411821559071312

LCE3D 0.721293028941631 0.926036795884051 0.60712215681569 1.41247381223732 0.289290200757674

LCK 0.0618172654709341 0.88581081942424 0.779969788495662 1.00601436027725 0.868228720221987

LGALS2 0.414547368513874 1.04103883490018 0.945167049414609 1.14663524975986 0.447693409428016

LIMD2 0.78079235000882 0.963917264066429 0.744101662833745 1.24866874833595 0.363281968320477

LIME1 0.939811599801085 1.00836918253639 0.812203255287735 1.25191373177752 0.649766545239537

LRRC15 0.445123219180413 0.943747768227035 0.813420535353043 1.0949561897241 0.615221674840009

LSAMP 0.846157823386337 0.987276511976269 0.86748354653886 1.12361198663537 -0.209750802085659

LY9 0.000458008736799752 0.535204100306642 0.377289421176146 0.759214048705887 0.619074045981416

LYPD3 0.0397844623441696 0.85190501924777 0.731194875753645 0.992542735028767 0.737788838218428

MAN1A2 0.366393591570879 1.10137801688912 0.893178885267089 1.35810816410407 -0.485747685774016

MAP4K1 0.295639892410608 0.866021905642427 0.66138328122522 1.1339777740725 0.831733945180932

MET 0.156609511965613 1.09051717547882 0.9673065430972 1.22942175725033 0.451700685890965

MLPH 0.817748956877987 0.983855622855347 0.856664558004143 1.12993105361948 0.706645250237803

MMP12 0.384898122088097 0.967855259395477 0.899090565066778 1.0418792494724 0.365541777250736

MMP1 0.615873077675312 1.01986142097828 0.944440026336571 1.10130584155183 0.560841611978054

MMP16 0.578847648938082 1.03746245484427 0.911115458375386 1.18133035206176 -0.562196680908061

MMP3 0.934030078446527 1.00363178861321 0.921075942886175 1.09358709767044 0.463732096693698

MS4A1 0.0622806145724516 0.886991121774979 0.781926406365579 1.00617301539219 0.794891985471097

MS4A2 0.126169271421698 0.900914369467771 0.78814968750632 1.02981288196857 0.610267433068273

MYO1G 0.783056545469079 1.02354700831141 0.867274468939872 1.20797799974885 0.753512988331342

NCOA2 0.345148193752119 0.898579038724129 0.719668665202357 1.12196671590161 -0.497511619177182

NCR1 0.00126143480696439 0.784510308670706 0.676915179969453 0.909207597380783 0.493959286540129

NFKB2 0.992055535708213 0.998815841508765 0.79103398564596 1.26117600931418 0.687015769956286

NHLRC2 0.0740371592243744 0.801183365153977 0.628216902228907 1.02177254754212 -0.185189506920024

NOVA2 0.0558941089733679 1.17383182556707 0.995975536391959 1.38344879403932 -0.613075871525319

NTNG2 0.691899739332294 0.978593132173039 0.879267739382431 1.08913869512472 -0.385855437490257

NTRK1 0.408124650139123 0.938546547732855 0.807593139080395 1.09073440527777 0.448752979263329

OVOL1 0.0739519162106735 0.85281887828851 0.716172174309739 1.01553797432337 0.685733470884842

P2RX1 0.674128192490861 1.06429712291148 0.796010191241798 1.42300736636369 0.492269067162461

P2RX5 0.0536267767274947 0.827271114154546 0.682354719644383 1.00296440635917 0.58314615315334

P2RY10 6.02274639141499e-05 0.689600008702336 0.575103884826062 0.826890905364149 0.668066864696732

PCDH17 0.989953124692613 0.999245910923271 0.888543494510879 1.12374058970126 -0.593865644755633

PCYOX1L 0.203206576406994 1.15753557270749 0.924003002361751 1.45009117790582 -0.235690041502277

PDCD1 0.275635841246028 0.915434785235863 0.780979321751129 1.07303845656349 0.676823706849052

PDE3A 0.184579040020405 0.880295931077867 0.729176719288297 1.06273404755517 0.329241938404649

PDE3B 0.0300567253103781 0.797875021406642 0.650630885970691 0.978441945366435 0.221405249770132

PDE4B 0.157104750386523 1.09943190442014 0.964139130275834 1.25370963017659 -0.439519220996575

PDZRN3 0.2472471101611 0.88721359570212 0.724423238271905 1.0865857454772 -0.283524266542483

PGLYRP4 0.015665545609895 0.81283827624872 0.687090918044939 0.961599179938189 0.804490459079829

PIM2 0.0916501280862094 1.19979244108511 0.970928012726275 1.48260415068567 0.558316560832007

PKHD1L1 0.0880066597740366 0.788301644672816 0.599800049117173 1.03604440164437 0.363462343739271

PKP3 0.297000053630352 0.913223170554105 0.769992878216384 1.08309645820195 0.738577604974577

PLA1A 0.326302628752017 0.92545921938558 0.792833475317141 1.08027069165208 0.515029816903663

PLEKHN1 0.963400722464195 0.997474070263526 0.895335792271185 1.11126409715421 0.757123002341728

PNMA2 0.00187777713464387 1.31258579213897 1.10574751593701 1.55811470240123 -0.644272452169925

POU2AF1 0.786464759489484 0.984573029202247 0.879827288883139 1.10178902391518 0.540737242343834

PPL 0.373480043777528 1.04898426229916 0.944126192940369 1.16548824805331 0.343956027062547

PRG2 0.195201809807397 0.885084943092713 0.735815115180236 1.06463612982121 0.597572099443606

PRLR 0.0357994226388836 0.796731178506641 0.644420510520936 0.98504091729085 0.46405859590766

PSMC5 0.0476721337961519 1.22078502946057 1.0020486860514 1.48726914061198 -0.370714924603179

PSMD4 0.213566604714827 1.17722873577946 0.910334156694734 1.52237229170523 -0.151318138313607

PSMD6 0.650431328273836 1.06140198139229 0.820252240844482 1.37344844671638 -0.500732419039454

PSMD7 0.412796079611556 0.889938846942098 0.673240967078326 1.17638585591968 -0.21712240265883

PSME3 0.0999370026796526 1.30360218645003 0.950531977246018 1.78781850710685 -0.467437125845967

PTGER2 0.0298821983821259 0.85821808425435 0.747597741699693 0.985206668049395 0.701397380527113

PTGFR 0.0132767650563889 0.871034813273611 0.780860544239042 0.9716224638728 0.71283704752353

PTPN7 0.96876231887735 1.0050130320985 0.782491936035258 1.29081355113455 0.589837602424722

PTPRCAP 0.440126681487131 0.9159434265727 0.732921854674363 1.14466822803979 0.730406332295058

PVR 0.996074978350955 0.999485113916879 0.814068893882027 1.2271326179504 0.367045551052891

PVRIG 0.082676600131183 0.842331452340643 0.693939461893933 1.02245558087421 0.793398712999026

QPRT 0.797327931851143 0.981108561890854 0.848205804863855 1.13483544287939 0.390350188799219

RAB39B 0.776705810348058 0.979096476186975 0.846103585673004 1.13299355529766 -0.471312782628298

RAD23B 0.955982428329866 1.00782681099853 0.764108164764263 1.32928154390398 -0.172040461671927

RAET1E 0.0253916153310705 0.829017846724439 0.703334149288714 0.977160842940252 0.773392012575695

RASSF2 0.789113636017477 1.02434303281678 0.858818196617592 1.22177039682298 -0.526373167477316

RASSF3 0.0283455931647337 0.627890479653446 0.414191995315163 0.951844697383512 0.338414399197254

RASSF6 4.08310130600509e-05 0.596502179403768 0.46603323863208 0.763496721988859 0.378000634799788

RBP5 0.0611155733522086 0.83082363982698 0.684329237708582 1.00867810764107 0.693600399720568

RC3H2 0.943501655411755 1.00918390203467 0.783731662686606 1.29949088012433 -0.346278617444648

RFXAP 0.282188187633738 0.921998004886627 0.795151377768957 1.06907985671871 -0.601800943448539

RNASE7 0.283435765482159 0.854155044989287 0.640379578952841 1.13929435737734 0.415780059861269

RNF180 0.349560205040574 1.0595276688276 0.938634147245923 1.195991946708 -0.53899751616651

ROCK2 0.0283806952910816 1.2685154180735 1.02549551529332 1.56912569766817 -0.403103977429214

S100A7 0.00806731401990237 0.81609316818749 0.702171046456318 0.948498321774261 0.474154360007856

S100A7A 0.368630433415013 0.910597580935069 0.742430571872813 1.1168558863533 0.302130298004944

SAMD14 0.628878342202283 1.06022860241555 0.836365688322766 1.3440110050835 -0.497536220898761

SARDH 0.175210688696687 1.25498256568145 0.903711488868421 1.7427920963321 0.513260739864145

SBSN 0.542321618901517 0.939013968158772 0.766947835889691 1.14968344799411 0.514341558003326

SCUBE3 0.00666879999604731 0.673689067937645 0.506442685841494 0.896166482303076 0.581678331343086

SDCBP2 0.110248559794157 1.10272724050512 0.978026039897715 1.2433282114648 -0.286912945535456

SERPINA1 0.936693878946176 1.00608117309801 0.866281191519588 1.16844199870797 0.706192732895776

SFN 0.345238261665603 0.924011501623031 0.784161429773596 1.08880292081971 0.804777928415197

SFRP2 0.239925602419304 0.959938564128708 0.896662244059687 1.02768021404517 0.305221894835581

SGCD 0.198758422077402 0.937677449430053 0.850023353898049 1.03437040304554 0.421651534183182

SGIP1 0.977663837101696 1.00157782357798 0.896918452282686 1.1184496585282 -0.563261478306993

SH2D1A 0.0137203103091999 0.844138022714002 0.737720341682498 0.965906673748985 0.542924995022156

SH2D2A 0.363932424304607 0.940096080149153 0.822728655255463 1.07420670748047 0.765063265310225

SIT1 0.329052566136683 0.941419288214553 0.833943534762017 1.0627461444081 0.746253176580905

SKAP1 0.118262148764538 0.91321933411154 0.814908883755003 1.02338993821284 0.747127715505568

SLA2 0.140817520023267 0.817014807157874 0.624344234420841 1.06914288354792 0.573884680320977

SLAMF6 0.22407605478696 0.791465441622802 0.542864080573344 1.15391231009719 0.350727817666995

SLC10A2 0.253133832083288 1.18138738729076 0.887630808669569 1.57236110465973 0.177169756460977

SLC12A3 0.0563954175844915 0.906879789546306 0.820243088271188 1.00266733660746 0.882507324431381

SLC18A2 0.841816384095065 0.981707993615397 0.818920120212471 1.17685542330834 0.409009751508089

SLC24A4 0.136087748467479 1.06054907227834 0.981655236929281 1.14578346082971 -0.574209505630461

SLC25A45 0.759313431618698 1.05383879230419 0.753503255004848 1.47388374607355 0.534448187426125

SLC39A2 0.734775964699784 0.962772169806044 0.773038339705663 1.19907409935964 0.423766549892736

SLURP1 0.40236696488981 0.856641161598143 0.596365857012657 1.2305098809985 0.495461324981884

SMPDL3B 0.0459686488938762 0.878122709302012 0.772895218502133 0.997676624376447 0.850304628561814

SON 0.25792701946621 1.13720276732747 0.910122305138787 1.42094103914974 -0.639608804871608

SOX5 0.279274358576956 1.07583458747097 0.942406411014972 1.22815384750227 -0.479731295205854

SPN 0.20079852066008 0.864094488914874 0.690824498001136 1.08082340440079 0.767013332543609

SPON1 0.298835558087209 0.969673610898415 0.914944029675726 1.02767697386475 -0.250120696399796

SPRR1B 0.0578713879076576 0.911468014473139 0.828207982710581 1.00309820570508 0.845344489570511

SPRR2D 0.258685403051 0.916393717988512 0.787563027135101 1.06629871824182 0.780138603427138

ST3GAL5 0.15134852629162 1.12098185903922 0.959058822379729 1.31024322906181 -0.286649071255207

STAT5A 0.658069480212429 1.05198088822455 0.840516388677215 1.31664748492455 0.686787541104718

STK33 0.556344163349293 1.03759639057941 0.917543613539633 1.1733570523042 -0.201086680123317

SULT1C2 0.0139145205224215 0.816634965064412 0.694900153295514 0.959695667072546 0.41105470089715

SULT1C4 0.187447447169071 0.902365978091913 0.774536593214767 1.05129230245677 -0.550662089865912

SULT2B1 0.697623724225148 1.03206526398368 0.88015783078877 1.2101905724876 0.455615842767622

SYT11 0.0687568075830403 1.17404771744296 0.987737475973617 1.39550040001707 -0.62810538422632

TAP2 0.19863371093364 0.790118416796442 0.551697327827482 1.13157537851286 0.368616898301335

TBX21 0.414914511929666 1.03771010673788 0.949354511246355 1.13428888035959 -0.541690912680826

TCEAL7 0.0356140757115594 1.12909070566923 1.00820014702911 1.2644769249293 -0.531656065158746

TGM2 0.660835396502433 0.96111563671994 0.80506982107345 1.14740764461384 0.616313404908289

THPO 0.929336450145045 1.01131369668416 0.788678326452119 1.29679662645461 0.503044178786273

THSD7A 0.622303525938139 1.02367864616929 0.932660167176459 1.12357963543728 -0.544245209138666

TLR9 0.670588383063602 0.957815307489939 0.785287044963459 1.16824818280905 0.71352381719757

TMC8 0.0580688257078607 0.762167256913866 0.575530713328745 1.00932741565053 0.81877805324893

TMIGD2 0.348483422804171 0.881761206736201 0.677804560238356 1.14708998923151 0.597486144589244

TNFRSF10A 0.103153439838014 0.85077538513273 0.700502048027463 1.03328570985044 0.664528907892048

TNFRSF13B 0.0635201665777459 0.840793692557403 0.700068187781348 1.00980739559774 0.841569639481902

TNFRSF17 0.0394222229197529 0.88102664909929 0.780985900447105 0.993882163530421 0.646578418329161

TNFRSF18 0.369682752379539 0.931508327468008 0.797745213501703 1.08770037031428 0.834531131946096

TNFRSF8 0.779212871242043 0.974822085981151 0.815642205568909 1.16506734549596 0.676686051561526

TNFRSF9 0.210653611829026 0.906817602830869 0.778048425632979 1.05689843679708 0.577939218739247

TNFSF15 0.903710111277542 0.985335640223962 0.775603792197546 1.25178130079112 0.659114484462016

TNFSF18 0.907559129442856 1.01232181724035 0.823276124225521 1.24477733715994 0.155247815341928

TNFSF9 0.322733535301457 0.922480642671956 0.786141535261751 1.08246479537699 0.841062780483395

TNIK 0.847629453100716 1.01160781290209 0.899260450972964 1.13799107479632 -0.546081309996787

TPSD1 0.369456927754901 0.837560231710939 0.568717413666668 1.23348982268871 0.533910647192531

TRAT1 0.00999861418896133 0.813246813763313 0.69488238702924 0.951773123684527 0.652589849851818

TTBK2 0.882364292672627 1.02742992789597 0.717941375077647 1.47033210980793 -0.224891518759658

TTC21B 0.637680455470594 0.941894469999685 0.734184953482424 1.20836743985005 -0.342266409167681

TXK 0.0421579526595828 0.795521186325465 0.638004105658644 0.991927720025167 0.617623548503209

UBASH3A 0.0144635850304042 0.805453840806345 0.677231962260557 0.957952261296392 0.640523617180941

UBR1 0.518743123595955 0.919120013779437 0.711410466858755 1.18747423475516 -0.233929964444458

ULBP1 0.31312263576763 0.891433693972655 0.713030774964162 1.11447367862863 0.249023731327474

ULBP3 0.325119941955148 0.884565085457645 0.692824839310392 1.12936971369201 0.54613721112405

UTS2 0.442937344165374 0.972300415017736 0.904973976752303 1.04463567055963 0.716918564772088

VGLL3 0.226676503961627 0.873625587860883 0.701782350493734 1.08754753839038 0.533859507607523

ZAP70 0.0662195860031787 0.820410988142245 0.664209603020063 1.01334606787401 0.741449284276301

ZBTB10 0.614104409890357 0.921439444793487 0.670418343013619 1.2664490154084 -0.498793635499531

ZNF215 0.142940743573015 0.905060896975799 0.791983105938122 1.03428371273697 0.478013565900892

ZNF620 0.900052444948077 1.01090744653759 0.853469679140196 1.19738742973817 -0.582327257475625

ZNF671 0.414346517461089 0.925379322420424 0.76815587787157 1.11478270886375 -0.220447108028794

ZNF80 0.0425762205529351 1.32801058952802 1.00954113218085 1.74694429942517 -0.313626291563612

EIF2AK3 0.668429442544386 0.957579407139302 0.785287711392547 1.1676718070008 -0.52202574464657

EIF2AK4 0.173275754776768 1.17065232448723 0.933125350696895 1.46864176801527 -0.348158042068102

EIF2AK1 0.426027733484783 1.14708211675144 0.818197826281616 1.60816533643306 -0.247002200815161

IFNA1 0.589339640026556 0.884718216456241 0.5670956275362 1.38023692041171 0.156102871168892

VTCN1 0.0666810191007845 0.887958819898738 0.782052049638841 1.00820765855685 0.526756753786936
